# Mecp2 protects kidney from ischemia-reperfusion injury through transcriptional repressing IL-6/STAT3 signaling

**DOI:** 10.7150/thno.72515

**Published:** 2022-05-09

**Authors:** Jiao Wang, Mingrui Xiong, Yu Fan, Chengyu Liu, Qing Wang, Dong Yang, Yangmian Yuan, Yixue Huang, Shun Wang, Yu Zhang, Shuxuan Niu, Junqiu Yue, Hua Su, Chun Zhang, Hong Chen, Ling Zheng, Kun Huang

**Affiliations:** 1 Tongji School of Pharmacy, Tongji Medical College, Huazhong University of Science and Technology, Wuhan, China, 430030.; 2 Hubei Key Laboratory of Cell Homeostasis, Frontier Science Center for Immunology and Metabolism, College of Life Sciences, Wuhan University, Wuhan, China, 430072.; 3 Department of Transfusion Medicine, Wuhan Hospital of Traditional Chinese and Western Medicine, Tongji Medical College, Huazhong University of Science and Technology, Wuhan, China, 430030.; 4 Department of Pathology, Hubei Cancer Hospital, Tongji Medical College, Huazhong University of Science and Technology, Wuhan, China, 430030.; 5 Department of Nephrology, Union Hospital, Tongji Medical College, Huazhong University of Science and Technology, Wuhan, China, 430030.

**Keywords:** renal ischemia-reperfusion, Mecp2, inflammation, IL-6/STAT3 signaling, fibrosis

## Abstract

**Rationale:** Ischemia-reperfusion (IR) induced acute kidney injury (AKI) causes serious clinical problems associated with high morbidity and mortality. Mecp2 is a methyl-CpG binding protein, its mutation or deletion causes a neurodevelopment disease called Rett syndrome. Notably, some Rett syndrome patients present urological dysfunctions. It remains unclear whether and how Mecp2 affects AKI.

**Methods:** Renal tubular cell specific Mecp2 deletion mice challenged with IR injury were used to investigate the effects of Mecp2 on renal tubular damage, function, cell death, fibrosis and inflammation. Cultured renal epithelial cell lines were transfected with wildtype or different domain-deletion mutants of Mecp2 to study the effects of Mecp2 on Il-6/STAT3 signaling.

**Results:** Our results indicated rapidly upregulated Mecp2 upon acute *in vivo* and *in vitro* renal injury. Notably, increased tubular MeCP2 staining was also found in the renal sections of AKI patients. Furthermore, ablation of Mecp2 aggravated renal injury, and promoted renal cell death, inflammation, and fibrosis. Mechanistically, through its transcriptional repression domain, Mecp2 bound to the promoter of proinflammatory cytokine *Il-6* to negatively regulate its expression, thus inhibiting STAT3 activation.

**Conclusions:** A novel protective role of Mecp2 against AKI *via* repressing the Il-6/STAT3 axis was suggested.

## Introduction

Acute kidney injury (AKI), a disease with high morbidity and mortality, is defined by a rapid increase in serum creatinine, decrease in urine output, or both 1. Annually, about 13.3 million patients are diagnosed with AKI, which cause approximately 1.7 million deaths 1. Furthermore, patients incomplete recovery from AKI may subsequently develop chronic kidney disease, and eventually end-stage renal disease 2.

Renal ischemia/reperfusion (IR) injury, a major challenge during kidney transplantation, cardiac, thoracic, and vascular surgery, is a common cause of AKI 3, 4. Among multiple types of renal cells affected by IR injury, proximal tubular epithelial cell (TEC) is the major type contributing to the development of AKI 5. Upon renal IR injury, TECs undergo apoptosis and/or necrosis to release proinflammatory cytokines and chemokines, which aid in recruiting immune cells 6. TECs and recruited immune cells sustained produce the profibrotic cytokines such as TGFβ1, thus cause progressive renal dysfunction 6. Together, inflammatory cell infiltration followed by acute TEC injury induces interstitial fibrosis 7.

IL-6 (Interleukin-6) is a proinflammatory cytokine involved in the pathogenesis of many inflammatory diseases 8. Systemic or local insults such as hypoxia, proinflammatory cytokines and chemokines, initiate synthesis and secretion of IL-6 by TECs 9. Upon renal IR injury, rapidly increased Il-6 level has been reported in the kidney and serum which is harmful to the kidney, since Il-6 knockout mice show less renal injury caused by AKI 10. Upregulated renal Il-6 subsequently leads to the initiation of STAT3 signaling, a pathway often constitutively active in inflammatory response 8. Moreover, Il-6 can trigger TECs to generate collagen and accelerate interstitial fibrosis, which is also associated with enhanced STAT3 activation 11; while blocking Il-6 protects against IR-induced renal fibrosis by suppressing STAT3 activation 12. All these indicate that Il-6/STAT3 signaling plays a critical role in renal IR injury.

Mecp2 (methyl-CpG binding protein 2), a member of methyl-CpG binding protein family, is a multi-function protein with four domains 13, 14. The N-terminal domain (NTD) interacts with a repressive chromatin regulator heterochromatin protein 1 15; the methyl-binding domain (MBD) specifically binds to methylated-CpGs 16; the transcription repression domain (TRD) recruits gene co-repressors to suppress transcription 17; and the C-terminal domain (CTD) facilitates Mecp2 binding to DNA and the nucleosome core 18, 19. Mutation of Mecp2 is the main cause of Rett syndrome (RTT), a neurodegenerative disease characterized with loss of acquired speech and motor skills, breathing irregularities and seizures 20. A recent study identified additional urological dysfunction from over 1000 RTT patients and Mecp2 whole body knockout mice, and Mecp2 whole body knockout mice may die of kidney failure due to obstructive nephropathy as they develop hydronephrosis from urethra obstruction 21. Furthermore, increased phosphorylated Mecp2 has been found in the kidneys of type 1 diabetic mice 22, suggesting Mecp2 may have unknown function in the kidney. However, the role of renal Mecp2 in kidney diseases, such as IR-induced AKI, remains unclear.

Here, we found that renal IR injury dramatically upregulated Mecp2 level, renal tubular cell specific knockout of Mecp2 aggravated renal tubular injury by increasing inflammation, cell death and fibrosis. Mechanistical studies demonstrated that the Il-6/STAT3 pathway was significantly regulated by Mecp2. Further mapping study indicated the TRD domain of Mecp2, which directly binds to the promoter of *Il-6*, is responsible for the repression of Il-6/STAT3 signaling.

## Methods

### Animals and renal IR model

LoxP-flanked *Mecp2^flox/y^* (male) and *Mecp2^flox/flox^* (female) mice (JAX, No. 007177) in B6.129P2 background were backcrossing to the C57BL/6 background for more than 10 generations in our lab to generate *Mecp2^flox/+^* female mice, then crossing *Ksp*-Cre*^ +/-^* mice in the C57BL/6 background (a kind gift of Dr. CY Wang, Tongji Medical College) to further generate renal tubular cell specific Mecp2 KO mice. Genotyping was performed (primers listed in Table S1), and male offspring with indicated genotypes were used. Mice were housed and handled as we previously reported 23, 24, all animal experiments were approved by the Committee on Ethics in the Care and Use of Laboratory Animals of College of Life Sciences, Wuhan University.

Renal unilateral IR injury was performed as we previously described to reduce surgery caused mortality 25. Briefly, mice (10-12 weeks, male) were anesthetized and left renal pedicles were bluntly clamped for 45 minutes. Reperfusion was achieved by removing the clamps. Kidneys were harvested at 1, 3, 7 or 16 days after the injury.

### Assessment of renal function

Serum levels of nitrogen (BUN) and creatinine were analyzed with a Siemens ADVIA 2400 automatic biochemical analyzer using a BUN or creatinine reagent kit (all from Fuxing Changzheng Medical, Shanghai, China) as we previously described 26.

### Human renal tissue specimens

Human renal biopsy samples, including acute kidney injury samples and the control para-carcinoma renal tissues, were collected by the affiliated Hubei Cancer Hospital and Union Hospital of Tongji Medical College. Five AKI biopsy samples were obtained from kidney donors presenting mild acute tubular injury (determined by an experienced renal pathologist) during organ procurement. Collection and use of patient samples were approved by the institutional review boards of these hospitals. All subjects included in the study provided written informed consent.

### Mouse primary tubular epithelial cells isolation

Mouse primary tubular epithelial cells (mPTEC) were isolated from renal cortex of C57BL/6 mice. Minced renal cortex was digested in 1 mg/ml type II collagenase (Sigma-Aldrich, Saint Louis, MO), and then sequentially passed through 200-μm and 70-μm cell strainers. Cells were collected for qPCR examination or cultured in RPMI-1640 medium with 5 mg/L human epidermal growth factor (PeproTech, Rocky Hill, NJ) for purity examination by immunofluorescence staining.

### Renal histology, immunohistochemical and immunofluorescent staining, and quantification of renal tubules

H&E staining and the degree of renal damage assessment were performed as we previously described 26, 27. Sirius Red (SenBeiJia Biotech., Nanjing, China) staining was performed to examine fibrosis. For immunohistochemical staining for Mecp2 or p-STAT3, sections were incubated overnight with respective primary antibodies (Table S2) and visualized by DAB substrate followed with the ABC kit (both from Vector laboratories, Burlingame, CA), quantification of positive stained cells were performed as we previously reported 23, 28.

For immunofluorescent staining, sections were incubated overnight with anti-Mecp2 primary antibody (Table S2). After washing, sections were incubated with Alexa Fluor secondary antibody (Thermo Fisher Scientific, Eugene, OR). For renal tubular staining, sections were incubated with 1 μg/mL PNA (peanut agglutinin, detecting distal tubules and collecting ducts), or 1 μg/mL LTL (lotus tetragonolobus lectin, detecting proximal tubules), or 5 μg/mL DBA (dolichos biflorus agglutinin, detecting collecting ducts) (all from Vector Laboratories), respectively. Sections were covered with 4,6-diamidino-2-phenylindole (DAPI) dye and antifading medium. Images were taken by a confocal microscope (Leica SP8, Germany) with 3-12 fields per renal sample. Positively stained cells or tubules were manually counted, and quantified using Image-Pro Plus 6.0 software.

For mPTEC purity examination, cells were fixed and blocked, primary antibody against pan-keratin (Table S2), a marker for epithelia cells was applied overnight at 4 °C 29. After washing, cells were incubated with an Alexa Fluor 488-conjugated mouse secondary antibody (Thermo Fisher, Eugene, OR). Imaging was taken with an Olympus BX60 Microscope.

### TUNEL assay

For TUNEL assay, positive cells were detected by an *In situ* Cell Death Detection Kit (Roche, Mannheim, Germany) on renal sections and quantitated as we previously described 26, 30.

### *In vitro* HR injury and treatments

A rat tubular epithelia cell line NRK52E (from the Shanghai Institute of Cell Resource Center, Shanghai, China) was cultured in DMEM media (Hyclone, Palo Alto, CA) plus 5% FBS (Lonsera, Shanghai, China). A mouse tubular epithelia cell line TCMK-1 (from Otwo Biotech., Guangzhou, China) was cultured in DMEM media plus 10% FBS. Cells were maintained in a 37 °C incubator with 21% O_2_, which was considered as normal conditions. *In vitro* hypoxia and reperfusion (HR) experiments were performed as previously described 25, 31. For IL-6 treatment, cells were starved overnight in medium without FBS, then treated with 50 ng/mL recombinant human IL-6 (Peprotech, Rocky Hill, NJ) for 30 minutes.

### Plasmids, transfection and generation of Mecp2 knockdown cell lines

Flag-tagged mouse Mecp2 (full-length Mecp2) and backbone vector (pCMV6) were purchased from Origene (Rockville, MD). Four different Flag-tagged domain-deletion mutants of mouse Mecp2 (Mecp2-ΔN, Mecp2-ΔMBD, Mecp2-ΔTRD and Mecp2-ΔC) were cloned with SgfI and MluI restriction sites into pCMV6 vectors by standard procedures. To knockdown Mecp2, NRK52E cells were transfected with pLKO.1 vector containing either scrambled shRNA or shRNAs targeting Mecp2 (Table S1). Stable knockdown cell lines were established as we previously described 32.

### MTT assay

NRK52E cells were cultured at a density of 1000 cells/well in 96-well plates for 24 hours, and transfected with the empty vector or pCMV6-*Mecp2*. 24 hours later, cells were treated with HR stimulation, then MTT assay was performed as we previously reported 33, 34.

### RNA-sequencing, quantitative real-time PCR (qPCR), Western blots, and reporter assays

Total RNA of kidney samples from different groups were prepared for RNA-sequencing as we previously reported 35, 36. Sequencing and analysis were performed by Novogene (Beijing, China). qPCR and Western blots were performed as we previously described 37. Primers and antibodies used are provided in Tables S1 and S2. For reporter assay, promoter region of *Il-6* from -2000 to +500 of TSS (transcription start site) was cloned into pGL3-enhancer (Promega, Madison, WI). Five pGL3-*Il-6* luciferase reporter plasmids were constructed by inserting different regions of the *Il-6* promoter into pGL3-enhancer vector. NRK52E cells were transfected with indicated plasmids, luciferase assays were performed and analyzed as we previously described 38.

### Chromatin immunoprecipitation (ChIP)

ChIP assay was performed as we previously described 39, 40. Briefly, NRK52E cells or minced kidney tissues were crosslinked with 1% formaldehyde, then quenched with glycine. After washing, samples were resuspended with digestion buffer (50 mM Tris-HCl, pH7.6, 1 mM CaCl_2_, 0.2% Triton X-100) plus 1 mM PMSF. Crosslinked chromatin was sheared with Micrococcal Nuclease (New England Biolabs, Beverly, MA) and sonication. Chromatin was immunoprecipitated using anti-Mecp2 antibody or rabbit IgG (Table S2). The purified DNA was detected by qPCR with primer sequences provided (Table S1). Input samples were used as the internal control for comparison between samples. Agarose gel electrophoresis was used to confirm the expected size and a single product for each primer set. Formation of a single product was confirmed by the melting curve for each qPCR reaction.

### Statistical analyses

Data were expressed as average ± standard deviation (SD). Statistical significance was determined by analyzing the data with the nonparametric Kruskal-Wallis test followed by the Mann-Whitney test for comparison of three or more than three groups, or with the Mann-Whitney test only for comparison of two groups. Differences were considered statistically significant at p < 0.05.

## Results

### Mecp2 senses renal IR injury

**Figure 1:**
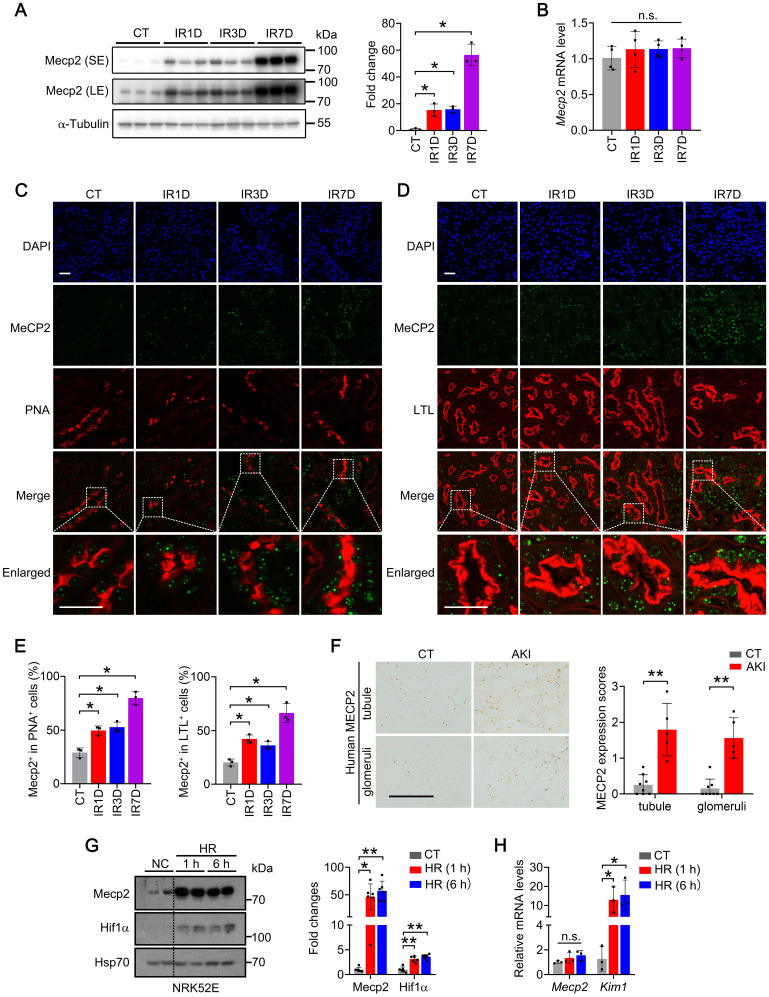
** Mecp2 senses renal IR injury. (A)** Western blots of Mecp2 (left) with quantitative results (right) of non-injured mice (CT) or mice at 1, 3, 7 days after IR injury (IR1D, IR3D, IR7D); SE, short exposure; LE, long exposure. **(B)** mRNA levels of *Mecp2* in the kidney after IR injury. **(C-E)** Representative co-immunofluorescent staining for Mecp2 (green) with PNA (peanut agglutinin; red; **C**) or LTL (lotus tetragonolobus lectin; red; **D**) in the kidney, and quantitative results of the percentage of Mecp2^+^ cells in PNA^+^ (**E**, left) or in LTL^+^ (**E**, right) tubular cells. DAPI (blue) stains nuclei; scale bar = 25 µm; n = 3 per group. **(F)** Representative immunochemical images (left) and quantitative results (right) of MECP2 in the kidney of AKI patients and control subjects (CT); n = 5-8 per group. Brown color indicates positive staining; scale bar = 100 µm. **(G)** Western blots of Mecp2 and Hif1α (left) with quantitative results (right) in NRK52E cells with 1 h or 6 h of hypoxia and reperfusion for 1 h (HR 1 h, HR 6 h). At least three biological replicates per group were used for these experiments. **(H)** mRNA levels of *Mecp2* and *Kim1* in NRK52E cells under HR 1 h or HR 6 h injury. At least three biological replicates per group were used for these experiments. NC, non-injured cells. *P < 0.05; **P < 0.01; n.s., not significant.

To investigate the role of Mecp2 in the IR-induced AKI injury, its protein level was first examined in wildtype mouse. Under physiological conditions, Mecp2 protein level was relatively low compared with the other tissues we examined (Figure S1). However, upon renal IR injury, dramatically increased protein level, but not transcriptional level of Mecp2 was observed in the kidney (Figure 1A-B). Immunohistochemical staining demonstrated weak Mecp2 staining in renal glomeruli and tubular cells under non-injured conditions; upon IR injury, significantly increased nuclear Mecp2 levels were found in glomeruli and renal tubular cells (Figure S2A). Immunofluorescent analysis was further performed to determine the type(s) of tubular cells expressing Mecp2 and its up-regulation after the injury. We co-stained with PNA (peanut agglutinin, detecting distal tubules and collecting ducts), LTL (lotus tetragonolobus lectin, detecting proximal tubules), DBA (dolichos biflorus agglutinin, detecting collecting ducts). While weak Mecp2 staining was found in the proximal tubular cells, distal tubular cells, and collecting tubular cells under non-injured conditions, significantly increased nuclear Mecp2 staining was found in these tubular cells at day one or day three after the injury, which were further increased at day seven after the injury (Figure 1C-E; Figure S2B). We noticed that Mecp2 distributed in the nucleus as punctuate, which may due to its capacity in reorganizing and clustering global chromatin architecture 41-43. Notably, significantly increased Mecp2 levels were also found in the renal tubules and glomeruli of AKI patients compared with those of the control individuals (Figure 1F).

Next, we examined whether hypoxia and reperfusion (HR) injury affects Mecp2 expression in TECs *in vitro*. HR injury was set up in NRK52E cells (Figure 1G-H) and TCMK-1 cells (Figure S3) as demonstrated by drastically upregulated Hif1α (hypoxia-inducible factor 1 subunit alpha) protein and *Kim1* (kidney injury molecule 1) transcription after injury*.* Consistent with *in vivo* IR injury, upregulated Mecp2 protein level, but not transcriptional level, was also observed upon HR injury (Figure 1G-H; Figure S3). These results indicate that Mecp2 may sense renal IR injury *in vivo* and *in vitro* which may contribute to the pathogenesis of AKI.

### Renal tubular cells specific Mecp2 knockout mice show normal renal morphology

**Figure 2:**
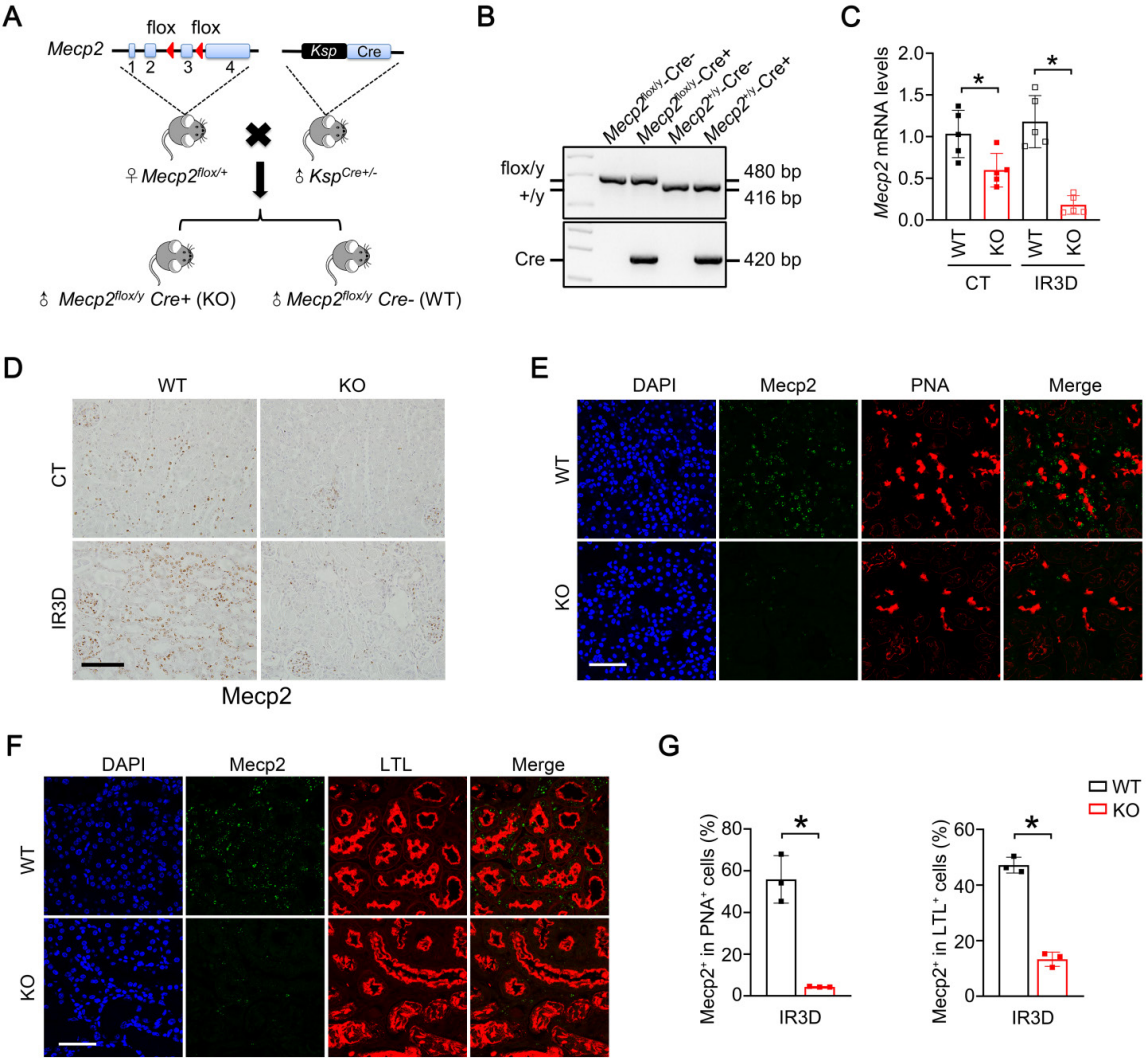
** Generation and verification of *Mecp2^Ksp^* KO mice. (A)** Experimental strategy to generate *Mecp2^Ksp^* KO mice. Blue boxes indicate exons of *Mecp2*, red triangles indicate LoxP site. **(B)** Representative genotyping results. **(C)** The mRNA levels of *Mecp2* in WT and *Mecp2^ksp^* KO mice with or without IR injury.** (D)** Representative immunohistochemical staining of Mecp2 in the kidney of WT and *Mecp2^ksp^* KO mice with or without IR injury; Scale bar = 100 µm. Brown color indicates positive staining. **(E-G)** Representative co-immunofluorescent staining for Mecp2 (green) with PNA (red; **E**) or LTL (red; **F**) in the kidney of WT and *Mecp2^ksp^* KO mice at day 3 after IR injury (IR3D), and quantitative results of the percentage of Mecp2^+^ cells in PNA^+^ (**G**, left) or LTL^+^ (**G**, right) tubular cells. DAPI (blue) stained nuclei; Scale bar = 50 µm. n = 3-6 per group. *P < 0.05.

Using female *lox*P-flanked *Mecp2^flox/+^* mice crossing with male *Ksp*-Cre mice (expressing Cre under the control of *Ksp-cadherin* promoter which is expressed exclusively in renal tubular epithelial cells of mouse and human 44), the tubule-specific ablation of Mecp2 mice were generated (Figure 2A). Since *Mecp2* is a X-linked gene 45, males genotyped as *Mecp2^flox/y^* (WT) and *Mecp2^flox/y^ Ksp*-Cre (*Mecp2^Ksp^* KO) were used in this study (Figure 2B). In non-injured conditions, significantly reduced mRNA and protein levels of Mecp2 in the kidney, but not in other examined tissues, were found in *Mecp2^Ksp^* KO mice (Figure 2C-D; Figure S4A-B).

*Mecp2^Ksp^* KO mice were born at the expected Mendelian frequency, with normal size and no physical or behavioral abnormalities. Under non-injured conditions, *Mecp2^Ksp^* KO mice showed normal body weight and normal kidney weight (Figure S4C-D); and exhibited similar renal morphology, including similar number of proximal tubules and distal tubules, as well as glomeruli, compared with those of the WT mice (Figure S4E-F).

At day three after IR, significantly reduced mRNA and protein levels of Mecp2 were also found in the injured kidneys of *Mecp2^Ksp^* KO mice (Figure 2C-D), further immunostaining suggested reduction of Mecp2 in the proximal, distal, and collecting tubules (Figure 2E-G; Figure S4G), but not in glomeruli (Figure 2D). While the body weights were similar between WT and *Mecp2^Ksp^* KO mice before or after IR injury (Figure S4C); the kidney weights were significantly increased in injured WT mice, but not in injured *Mecp2^Ksp^* KO mice, with a trend of decreased kidney weights observed in injured *Mecp2^Ksp^* KO mice compared to that of injured WT mice (Figure S4D).

### Renal tubular specific knockout of Mecp2 exacerbates renal IR injury

**Figure 3:**
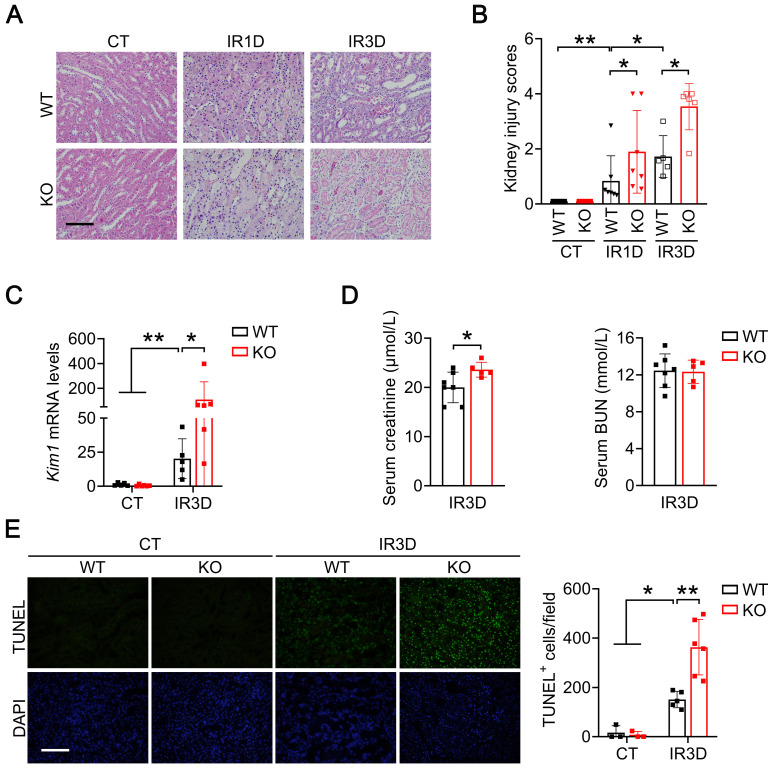
** Renal tubular specific knockout of Mecp2 exacerbates renal IR injury. (A-B)** Representative H&E images **(A)** with injury scores **(B)** of the kidney of WT and *Mecp2^ksp^* KO mice at 1 day (IR1D) or 3 days (IR3D) after the renal IR injury. **(C)** mRNA level of *Kim1* in the kidney of WT and *Mecp2^ksp^* KO mice with or without injury. **(D)** Serum creatinine and BUN (blood urea nitrogen) levels of WT and *Mecp2^ksp^* KO mice at IR3D. **(E)** Representative TUNEL images (left) with quantitative results (right) of WT and *Mecp2^ksp^* KO mice with or without injury. DAPI stained nuclei. Scale bar = 100 µm; n = 3-7 per group. *P < 0.05; **P < 0.01.

We next examined the pathological effects of *Mecp2^Ksp^* KO at different time points after the IR injury. *Mecp2^Ksp^* KO mice exhibited more severe renal morphological injury than WT mice at day one and day three after the renal IR injury (Figure 3A-B). Consistently, at day three after injury, transcriptional level of *Kim1* was significantly higher in *Mecp2^Ksp^* KO mice than that of the WT mice (Figure 3C). While a mildly increased serum creatinine level was found in the injured *Mecp2^Ksp^* KO mice (Figure 3D).

To explore whether more severe renal injury observed in *Mecp2^Ksp^* KO mice is due to increased cell death, we performed terminal deoxynucleotidyl transferase dUTP nick-end labeling (TUNEL) assay. While there was no difference in the number of renal TUNEL^+^ cells between non-injured WT and* Mecp2^Ksp^* KO mice, more cell death was observed in *Mecp2^Ksp^* KO mice than that of WT mice at day one or day three after the IR injury (Figure 3E; Figure S5).

### Renal tubular specific knockout of Mecp2 enhances activation of Il-6/STAT3 signaling upon IR injury

**Figure 4:**
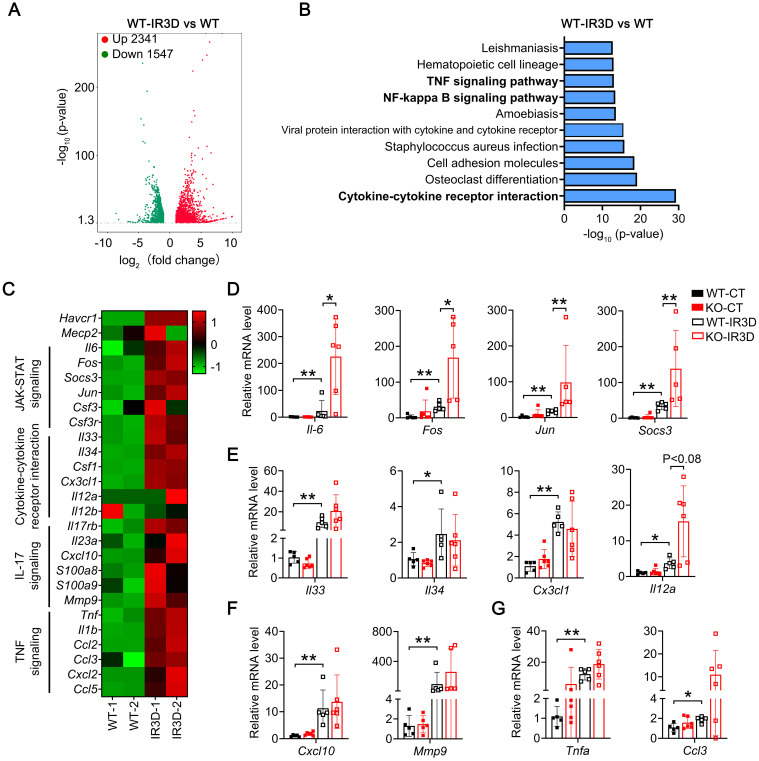
** Renal tubular specific knockout of Mecp2 enhances activation of Il-6/STAT3 signaling after IR injury. (A)** Volcanic map showing altered genes in the WT mice of injured kidney at day three after injury (IR3D) *vs.* non-injured kidney. **(B)** Top 10 KEGG pathways for differentially up-regulated genes in the WT mice of injured kidney at day three after injury (IR3D) *vs.* non-injured kidney. **(C)** Heatmap of inflammation-related pathways identified by RNA-seq. **(D-G)** qPCR validations of related genes of Il-6/STAT3 signaling **(D)**, cytokine-cytokine receptor interaction signaling **(E)**, Il-17 signaling **(F)**, and TNF signaling **(G)** in the WT and *Mecp2^ksp^* KO mice at IR3D. n = 5-6 per group. *P < 0.05; **P < 0.01.

To unbiased study signaling pathways altered by IR injury in the kidney, global gene expression profiles were analyzed by RNA sequencing (RNA-seq). Compared to non-injured kidney, 1637/1433 and 2341/1547 significantly upregulated/downregulated genes were found at day one or day three in the injured kidney of WT mice, respectively (Figure 4A; Figure S6A and Tables S3-4). Mecp2 usually acts as a transcriptional repressor 46, knockout of Mecp2 may upregulate certain genes that contribute to the aggravated phenotypes observed. Therefore, the upregulated genes identified by RNA-seq were further analyzed by KEGG, and among the top 10 significantly altered pathways, most are related to inflammation (Figure 4B; Figure S6B).

Since exacerbated inflammation contributes to the development of IR injury, we further analyzed enriched inflammation-related pathways in injured kidney of WT mice, including Il-6/STAT3 signaling, cytokine-cytokine receptor interaction signaling, Il-17 signaling, and TNF signaling related genes (Figure 4C), and examined identified genes in injured kidney of WT and *Mecp2^Ksp^* KO mice (Figure 4D-G). For Il-6 signaling pathway related genes, *Il-6*, *Fos*,* Jun* and* Socs3* (suppressor of cytokine signaling 3) were upregulated in IR-injured WT mice, and were further upregulated in injured *Mecp2^Ksp^* KO mice (Figure 4D). Increased Il-6 signaling as well as increased tubular injury, as demonstrated by significantly up-regulated *Il-6* and *Kim1* level, were also confirmed in isolated mPTECs at day one or day three after IR injury (Figure S7). On the other hand, cytokine-cytokine receptor interaction signaling related genes such as *Il33*,* Il34*, *Csf1* (colony-stimulating factor 1), and* Il12a*, were upregulated to similar extent in injured WT and *Mecp2^Ksp^* KO mice (Figure 4E). Furthermore, Il-17 signaling or TNF signaling related genes, *Cxcl10* (C-X-C motif chemokine ligand 10) and *Mmp9* (matrix metalloproteinase-9) or *Tnfa* (tumor necrosis factor *alpha*) and *Ccl3* (C-C motif chemokine ligand 3) were also upregulated to similar extent in injured WT and *Mecp2^Ksp^* KO mice (Figures 4F-G). Together, these results indicate specific enhanced activation of Il-6/STAT3 signaling in *Mecp2^Ksp^* KO mice after IR injury.

**Figure 5:**
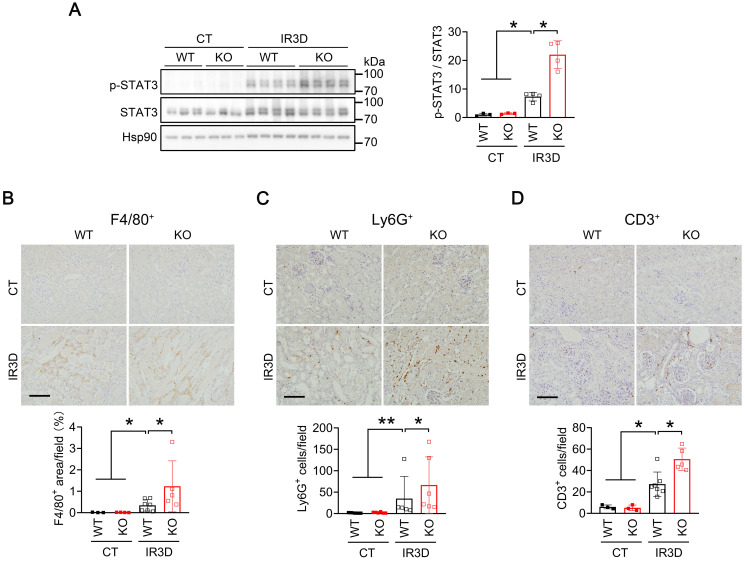
** Renal tubular specific knockout of Mecp2 increases STAT3 activation and inmmue cell infiltration after IR injury. (A)** Western blots of p-STAT3 and STAT3 (left) with quantitative results (right) in the kidney of WT and *Mecp2^ksp^* KO mice with or without injury. **(B-D)** Representative immunostaining for F4/80 (**B**, top), Ly6G (**C**, top), and CD3 (**D**, top) with quantitative results (**B-D**, bottom) of the kidney of WT and *Mecp2^ksp^* KO mice at IR3D. Scale bar = 100 µm. Brown color indicates positive staining; n = 3-7 per group. *P < 0.05; **P < 0.01.

To investigate whether activation of Il-6/STAT3 inflammatory pathway is responsible for aggravating AKI in IR-injured *Mecp2^Ksp^* KO mice, we examined the level of phosphorylated STAT3 (p-STAT3), the active form. Compared to the non-injured WT mice, increased p-STAT3 levels were observed in the kidney of injured WT mice, and were further increased in injured *Mecp2^Ksp^* KO mice either at day one or day three after the injury (Figure 5A; Figure S8A-B). Immunohistochemical staining demonstrated significantly increased nuclear p-STAT3 levels in tubular cells of *Mecp2^Ksp^* KO mice compared with WT mice after IR injury (Figure S8B). Activation of Il-6/STAT3 signaling after IR injury predicts increasing inflammation, which may increase inflammatory cell infiltration in the kidney. Consistently, the numbers of infiltrated macrophages (F4/80^+^ cells), neutrophils (Ly6G^+^ cells) and lymphocytes (CD3^+^ cells) were increased in injured kidneys of WT mice, and were further increased in injured kidneys of *Mecp2^Ksp^* KO mice either at day one or day three after the injury (Figure 5B-D; Figure S8C-E). Meanwhile, in HR stressed NRK52E cells, overexpressing Mecp2 ameliorated HR-induced cell death in a dosage-dependent manner (Figure S9).

### TRD domain of Mecp2 contributes to the suppression of Il-6 signaling

**Figure 6:**
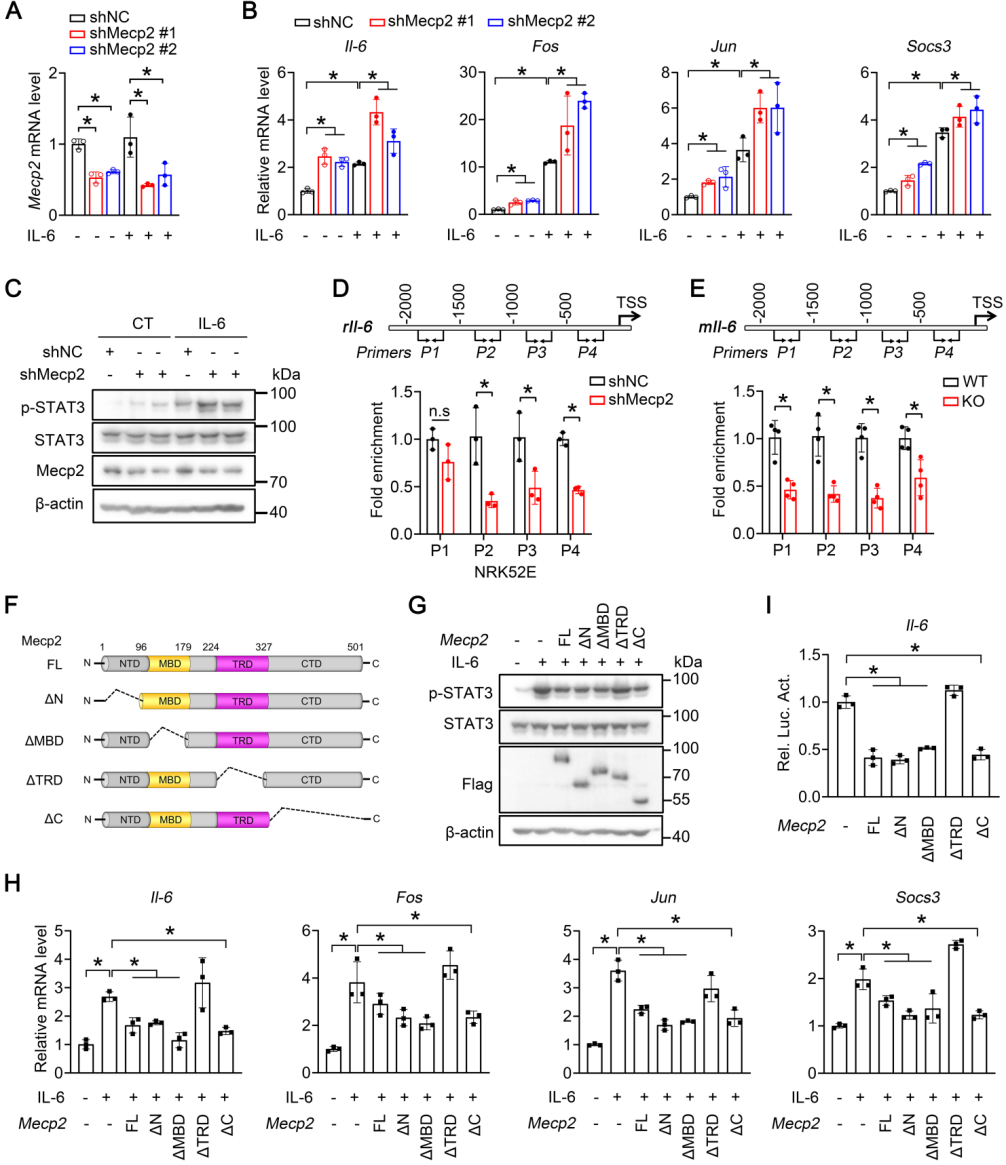
** Mecp2 TRD-dependently suppresses Il-6 signaling. (A)** Knockdown efficiency of* Mecp2* in NRK52E cells. **(B)** mRNA levels of *Il6*,* Fos*,* Jun* and* Socs3* in the indicated groups. **(C)** Representative p-STAT3 and STAT3 levels in the indicated groups. **(D)** Mecp2 binding affinity on the promoter of *Il-6* (four different regions) in NRK52E cells. **(E)** Mecp2 binding affinity on the promoter of *Il-6* (four different regions) in the kidney of WT and *Mecp2^ksp^* KO mice. **(F)** Illustration of domain-truncated Mecp2 constructs. **(G)** Representative p-STAT3 and STAT3 levels in NRK52E cells transfected with different Mecp2 constructs under IL-6 treatment. **(H)** mRNA levels of *Mecp2*,* Il-6*,* Fos*,* Jun* and* Socs3* in NRK52E cells transfected with different Mecp2 constructs under IL-6 treatment. **(I)** Luciferase reporter assays. At least three biological replicates per group were used for these experiments. TSS, transcription start site. *P < 0.05.

We next investigated whether Mecp2 directly regulates Il-6/STAT3 signaling. qPCR analysis demonstrated that knockdown of Mecp2 in NRK52E cells significantly potentiated the Il-6-triggered transcriptions of *Il-6*, *Fos*,* Jun* and* Socs3* (Figure 6A-B). Consistently, knockdown of Mecp2 enhanced Il-6-induced phosphorylation of STAT3 (Figure 6C). Furthermore, ChIP assays demonstrated enrichment of Mecp2 on the promoter of *Il-6* (Figure S10A-B); downregulated Mecp2 led to decreased Mecp2 binding on the promoter regions of *Il-6* both* in vitro* and *in vivo* (Figure 6D-E). Further luciferase report assay suggested that the binding region is located between -2000 to TSS of* Il-6* promoter (Figure S10C-D).

To pinpoint the domain of Mecp2 responsible for the activation of Il-6 signaling, wildtype and four domain-deletion mutants of Mecp2 (Mecp2-ΔN, Mecp2-ΔMBD, Mecp2-ΔTRD and Mecp2-ΔC) were constructed (Figure 6F) and transfected to NRK52E cells. Overexpression of wildtype Mecp2, Mecp2-ΔN, Mecp2-ΔMBD, or Mecp2-ΔC, but not Mecp2-ΔTRD, ameliorated the activation of Il-6 signaling as demonstrated by decreased Il-6-induced p-STAT3 level (Figure 6G), and decreased Il-6-triggered transcriptions of *Il-6*, *Fos*,* Jun* and* Socs3* (Figure 6H). Luciferase reporter assays further confirmed the binding and negative regulation of wildtype Mecp2 on *Il-6*; while among four different domain-deletion mutants, only Mecp2-ΔTRD lost the ability to bind and negatively regulate *Il-6* (Figure 6I; Figure S11A-E). Together, these results suggested that the TRD domain is involved in the suppression of Il-6 signaling.

### Renal tubular specific Mecp2 knockout mice show increased fibrosis upon IR injury

**Figure 7:**
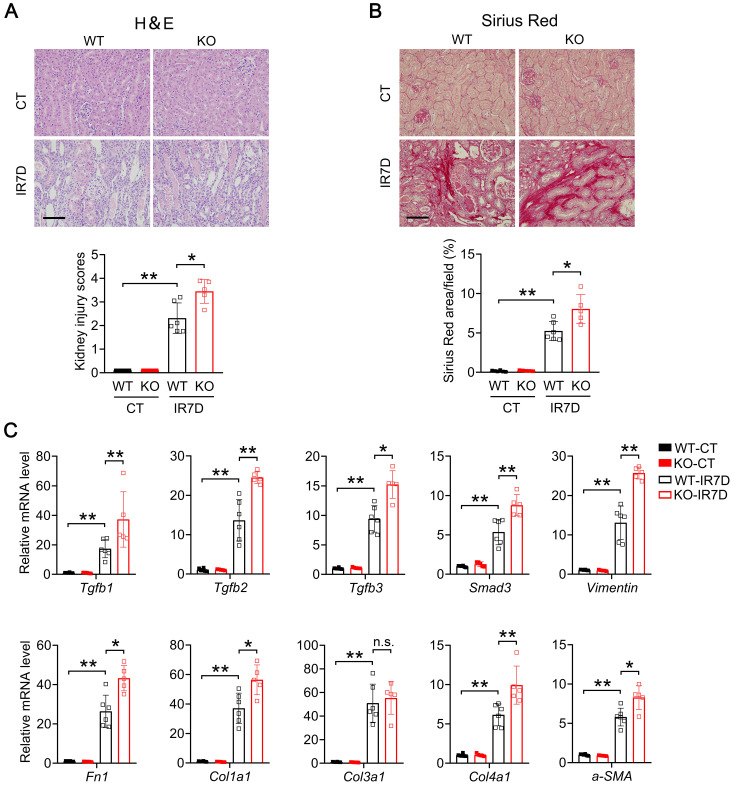
***Mecp2^Ksp^* KO mice show aggravated kidney injury and fibrosis at day 7 after IR injury. (A)** Representative H&E images (top) with injury scores (bottom) of the kidney of WT and *Mecp2^ksp^* KO mice at day 7 (IR7D) after the renal IR injury. **(B)** Representative Sirius Red staining images (top) with quantitative results (bottom) of the kidney of WT and mice under IR7D injury. **(C)** mRNA levels of indicated genes in the kidney of WT and *Mecp2^ksp^* KO mice at IR7D. Scale bar = 100 µm. n = 3-7 per group. *P < 0.05; **P < 0.01.

We next examined whether renal fibrosis is also elevated in IR-injured kidney of *Mecp2^Ksp^* KO mice. At day seven after renal IR injury, compared with the WT mice, *Mecp2^Ksp^* KO mice exhibited more severe renal morphological injury (Figure 7A), and aggravated renal fibrosis as indicated by Sirius Red staining (Figure 7B). Furthermore, compared to non-injured WT mice, significantly up-regulated fibrinogenic genes, including *Tgfb1*, *Tgfb2*, *Tgfb3*, *Smad3*,* Vimentin*,* Fn1*,* Col1a1*, *Col3a1*, *Col4a1* and *a-SMA*, were found in injured kidney of WT mice (Figure 7C); while all fibrinogenic genes, except for *Col3a1*, were further elevated in injured kidney of *Mecp2^Ksp^* KO mice compared to those of WT mice at day seven after IR injury (Figure 7C).

**Figure 8:**
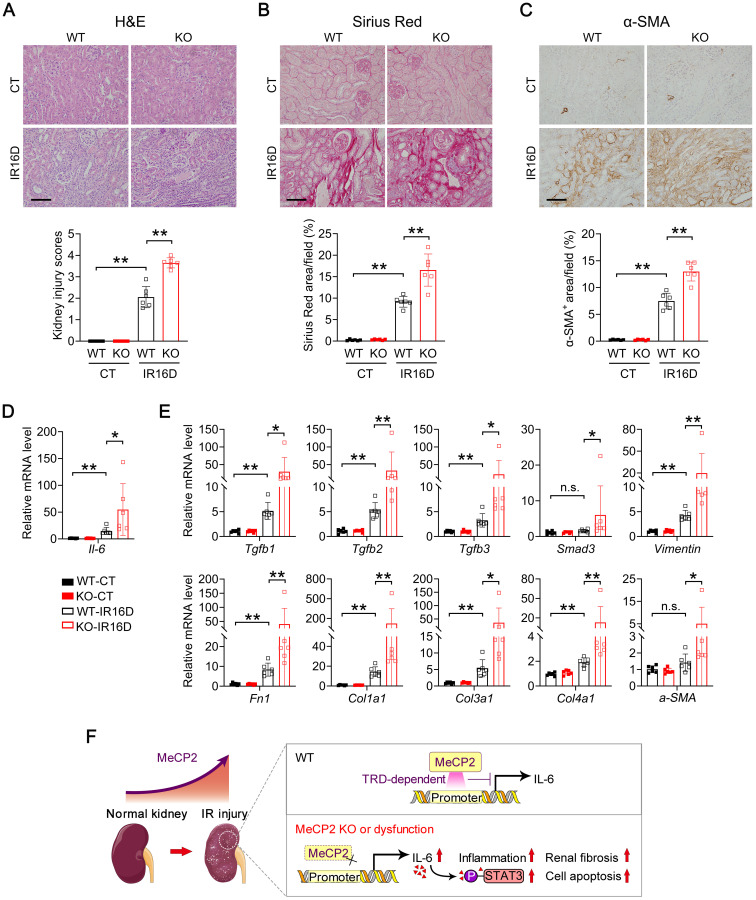
***Mecp2^Ksp^* KO mice show aggravated kidney injury and fibrosis at day 16 after IR injury, and a possible mechanism. (A)** Representative H&E images (top) with injury scores (bottom) of the kidney of WT and *Mecp2^ksp^* KO mice at day 16 (IR16D) after the renal IR injury. **(B)** Representative Sirius Red staining images (top) with quantitative results (bottom) of the kidney of WT and mice under IR16D injury. **(C)** Representative immunostaining for α-SMA (top) with respectively quantitative results (bottom) of the kidney of WT and *Mecp2^ksp^* KO mice at day 16 after the injury (IR16D). **(D-E)** mRNA levels of *Il-6* gene **(D)** and indicated fibrosis-related genes **(E)** in the kidney of WT and *Mecp2^ksp^* KO mice at IR16D. **(F)** Upon IR injury, upregulated Mecp2 plays a defensive role against increased* Il-6* through its TRD domain; in IR-injured *Mecp2^ksp^* KO mice, elevated Il-6/p-STAT3 signaling exacerbates inflammatory cell infiltration, extracellular matrix deposition, cell death, and the progression of AKI. Scale bar = 100 µm. n = 6 per group. *P < 0.05; **P < 0.01.

Consistently, at day 16 after renal IR injury, *Mecp2^Ksp^* KO mice exhibited more severe renal pathological injury and more aggravated renal fibrosis than those of WT mice, as indicated by H&E and Sirius Red staining (Figure 8A-B). The fibroblast activation was examined by α-smooth muscle actin (α-SMA) staining, significantly up-regulated staining was found in *Mecp2^Ksp^* KO mice (Figure 8C). Furthermore, upon renal IR injury, significantly elevated *Il-6* expression was detected in *Mecp2^Ksp^* KO mice compared to that of WT mice (Figure 8D). The transcription levels of all examined fibrinogenic genes were elevated in injured kidneys of *Mecp2^Ksp^* KO mice compared to those of WT mice at day 16 after IR injury (Figure 8E). These results indicated a highly fibrinogenic environment in injured kidney of *Mecp2^Ksp^* KO mice.

## Discussion

Mecp2 is well-known for its critical roles in neurological disorders, since mutation or deletion of Mecp2 is the predominant cause of RTT syndrome 47, 48. Besides neurological changes in RTT patients, abnormalities have been found in peripheral tissues, including metabolic and urological disorders, indicating non-central nervous system function of Mecp2 21, 49, 50. Recently, we reported a specific role of Mecp2 in adipose tissues that regulates browning and affects obesity 32. Here, we presented another novel non-neurological role of Mecp2 in the kidney, especially in renal tubular epithelial cells, which protects mouse from IR-induced AKI by reducing renal cell death, inflammation and fibrosis (Figure 8F). Low protein level of renal Mecp2 was found in adult mouse under physiological conditions (Figure 1A; Figure S1 and S2A), and the dramatic and rapid upregulation of Mecp2 after the injury, both* in vivo* and *in vitro*, make it a new sensitive marker for renal injury (Figure 1; Figure S2-3). Meanwhile, we found dramatically increased Mecp2 level in the glomeruli and renal tubules of AKI patients (Figure 1F). Together, these data clearly demonstrated an important role of Mecp2 in ischemia-induced AKI. Since dramatically up-regulated Mecp2 was found in proximal tubular cells, distal tubular cells, and collecting duct cells upon IR injury (Figure 1C-E; Figure S2B), the *Ksp*-Cre driven Mecp2 knockout was used in this study because *Ksp*-Cre is expressed in multiple tubules in the kidney 44, and successful Mecp2 knockout in proximal tubules, distal tubules and collecting tubules was observed (Figure 2E-G; Figure S4G).

Mecp2 is expressed in several immunological cells, such as peripheral macrophages and monocytes 51. A RTT mouse model with whole body Mecp2 knockout exhibits gradual loss of total meningeal macrophages and certain types of monocytes, suggesting impacts on development of immunological system 51. While overexpression of Mecp2 restrains the inflammatory response in macrophages and human peripheral blood mononuclear cells 51, 52. Consistently, our results confirmed that Mecp2 regulates inflammation in renal tubular epithelial cells. Mecp2 directly bound to the promoter of *Il-6* and repressed its transcription, therefore, ablation of Mecp2 enhanced inflammation *via* upregulating Il-6/STAT3 signaling in injured kidney (Figure 5-6; Figure S8).

As a chromatin-associated protein, Mecp2 can selectively bind to methyl-CpG containing DNA region and suppress gene transcription mostly through its MBD domain 14. By using DNA methylase inhibitors which downregulate DNA methylation level, the transcriptional suppression effects of Mecp2 were reversed in pancreatic cancer cell lines and hepatic stellate cells 53-55, which indirectly suggest MBD as a key domain for gene suppression. However, overexpression TRD but not MBD of Mecp2 suppresses gene transcription in *Xenopus* oocyte 56, while overexpression of TRD inhibits *β-galactosidase* transcription in mouse fibroblasts 17, indicating Mecp2 may also suppress transcription through TRD domain. Here, we demonstrated that Mecp2 TRD-dependently repressed *Il-6* transcription in tubular epithelial cells (Figure 6; Figure S11), suggesting Mecp2 may protect against AKI through its TRD domain.

Mecp2 has been shown to be an essential regulator of fibrosis in several human or animal tissues/organs, including liver, lung, muscle and skin 57-60. However, whether Mecp2 positively or negatively regulates fibrosis remains controversial. In a CCl_4_-induced liver injury model, Mecp2 null mice show ameliorated liver fibrosis through upregulating the anti-fibrinogenic gene *Pparγ* 61; while, in a bleomycin-induced lung injury model, Mecp2-null mice also show ameliorated lung fibrosis by transcriptionally inactivating the myofibroblast marker *a-SMA* 62. These results suggest a positive regulation on fibrosis by Mecp2. In addition, disorganized architecture and fibrosis have been found in the muscle of Mecp2 null mice 59; whereas in scleroderma fibroblasts, upregulated Mecp2 inhibits myofibroblast differentiation and fibroblast migration through direct activating the plasminogen activator urokinase, indicating Mecp2 as a negative regulator on fibrosis 60. Here, renal tubular specific knockout Mecp2 accelerated extracellular matrix components accumulation, and aggravated renal fibrosis at day 7 and day 16 after IR injury (Figure 7 and Figure 8A-E). Therefore, increased Mecp2 in injured kidney may be a defensive countermeasure to the pro-fibrotic nature of AKI.

As an intracellular mediator of Il-6/STAT3 inflammatory signaling, activation of STAT3 is not only pivotal to inflammation 63, but also activates fibroblasts 64. Suppressing STAT3 activation protects against IR-induced renal fibrosis 12, and ameliorates renal interstitial fibrosis in diabetic mice 65. Moreover, in dermal fibroblasts, STAT3 acts as a non-canonical downstream mediator that transmits the profibrotic effects of TGFβ 64. Therefore, enhanced STAT3 activation in injured kidney of *Mecp2^Ksp^* KO mice may also contribute to the aggravated fibrosis.

In summary, our results reveal a novel non-CNS (central nervous system) anti-inflammation and anti-fibrosis function of Mecp2 through regulating Il -6/STAT3 signaling in the kidney. Which addresses in part the mechanism behind the long-observed urological disorders in patients with Rett syndrome, and provides new angles for diagnosis and prognosis for AKI and other kidney disorders.

## Supplementary Material

Supplementary figures and tables.

## Abbreviations

IR: ischemia-reperfusion
AKI: acute kidney injury
Mecp2: methyl-CpG binding protein 2
TEC: tubular epithelial cell
IL-6: interleukin-6
NTD: N-terminal domain
MBD: methyl-binding domain
TRD: transcription repression domain
CTD: C-terminal domain
RTT: Rett syndrome
BUN: blood urea nitrogen
PNA: peanut agglutinin
LTL: lotus tetragonolobus lectin
DBA: dolichos biflorus agglutinin
HR: hypoxia and reperfusion
qPCR: quantitative real-time polymerase chain reaction
ChIP: Chromatin immunoprecipitation
Hif1α: hypoxia-inducible factor 1 subunit alpha
Kim1: kidney injury molecule 1
TUNEL: terminal deoxynucleotidyl transferase dUTP nick-end labeling
WT: wildtype
*Socs3*: suppressor of cytokine signaling 3
*Cxcl10*: C-X-C motif chemokine ligand 10
*Mmp9*: matrix metalloproteinase-9
*Tnfa*: tumor necrosis factor *alpha*
*Ccl3*: C-C motif chemokine ligand 3
p-STAT3: phosphorylated STAT3
α-SMA: α-smooth muscle actin
CNS: central nervous system
