# Supplementary material for: Mecp2 protects kidney from ischemia-reperfusion injury through transcriptional repressing IL-6/STAT3 signaling
Source: Theranostics. 2022 May 9;12(8):3896–910. doi: 10.7150/thno.72515 (PMC9131276; doi:10.7150/thno.72515)
Supplement: Supplementary file 1 — Supplementary figures and tables. [file thnov12p3896s1.pdf]

Supplemental Information

for

Mecp2 protects kidney from ischemia-reperfusion injury through transcriptional repressing IL-6/STAT3 signaling

Jiao Wang[1], Mingrui Xiong[1], Yu Fan[2], Chengyu Liu[3], Qing Wang[2], Dong Yang[1], Yangmian Yuan[2], Yixue Huang[1], Shun Wang[3], Yu Zhang[1], Shuxuan Niu[1], Junqiu Yue[4], Hua Su[5], Chun Zhang[5], Hong Chen[1], Ling Zheng[2*] & Kun Huang[1*]

[1]Tongji School of Pharmacy, Tongji Medical College, Huazhong University of Science and Technology, Wuhan, China, 430030

[2]Hubei Key Laboratory of Cell Homeostasis, Frontier Science Center for Immunology and Metabolism, College of Life Sciences, Wuhan University, Wuhan, China, 430072

[3]Department of Transfusion Medicine, Wuhan Hospital of Traditional Chinese and Western Medicine, Tongji Medical College, Huazhong University of Science and Technology, Wuhan, China, 430030

[4]Department of Pathology, Hubei Cancer Hospital, Tongji Medical College, Huazhong University of Science and Technology, Wuhan, China, 430030

[5]Department of Nephrology, Union Hospital, Tongji Medical College, Huazhong University of Science and Technology, Wuhan, China, 430030

Corresponding authors

Ling Zheng, Ph.D.
College of Life Sciences
Wuhan University
Wuhan, China 430072
lzheng@whu.edu.cn

Kun Huang, Ph.D.
Tongji School of Pharmacy
Huazhong Univ. of Sci. & Tech.
Wuhan, China, 430030
kunhuang@hust.edu.cn

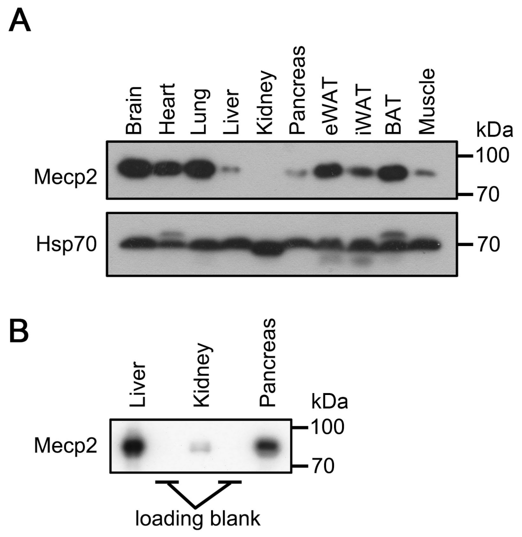

Figure S1. Mecp2 expression in several tissues from C57 male mice. Related to Figure 1. **(A)** Mecp2 protein level in indicated tissues from C57 male mice. **(B)** Western blots detecting Mecp2 protein in the liver, kidney and pancreas by longer exposure time.

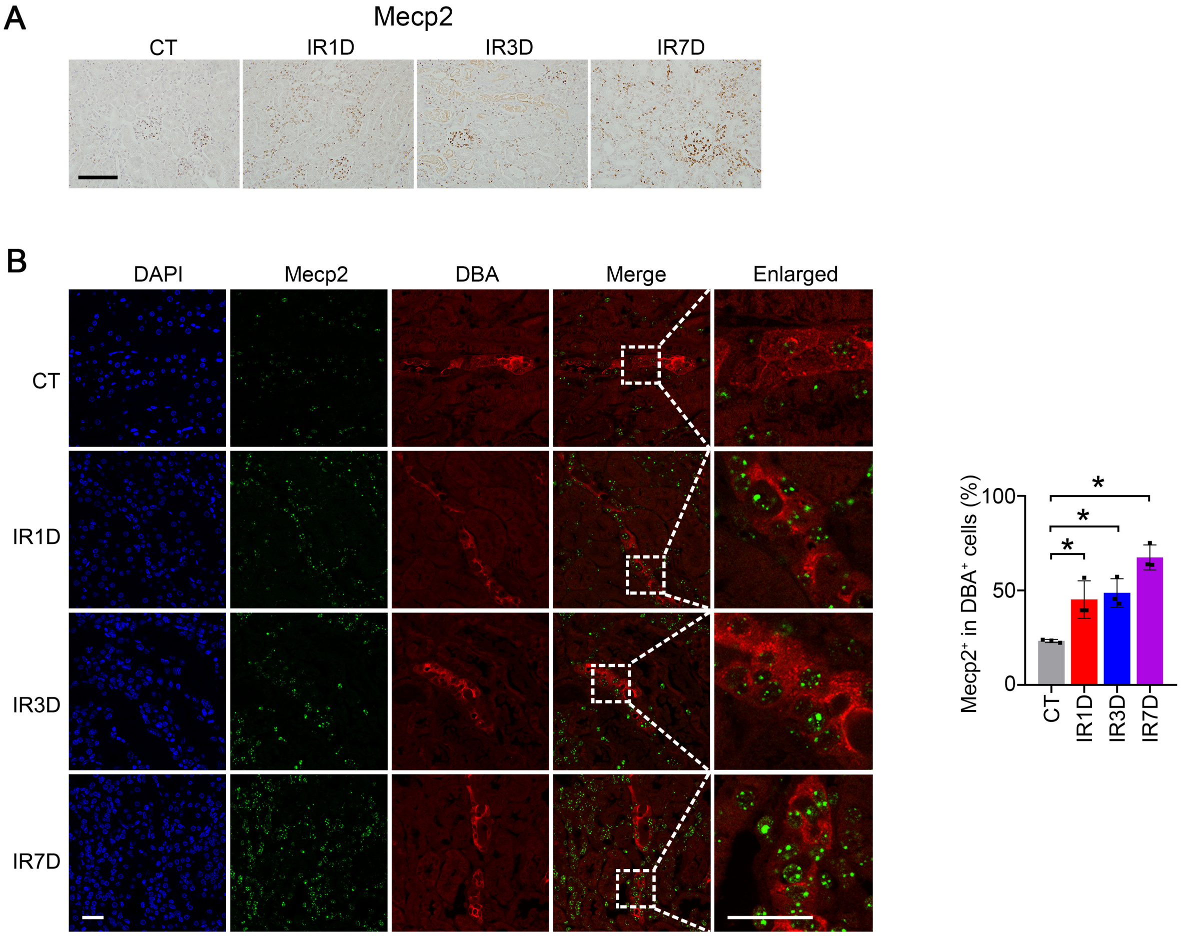

Figure S2. Mecp2 protein was up-regulated in the glomeruli and collecting tubules after IR injury. Related to Figure 1. **(A)** Representative immunochemical images of Mecp2 in the kidney before and at day 1, 3, 7 after the IR injury (CT, IR1D, IR3D, IR7D). n = 3 per group. Brown color indicates positive staining; scale bar = 100 μm. **(B)** Representative co-immunostaining for Mecp2 (green; left) with DBA (dolichos biflorus agglutinin; red; left) with quantitative results (right) in the kidney before and at IR1D, IR3D, IR7D. n = 3 per group. Scale bar = 25 μm. DAPI (blue) stained nuclei. *$P < 0.05$.

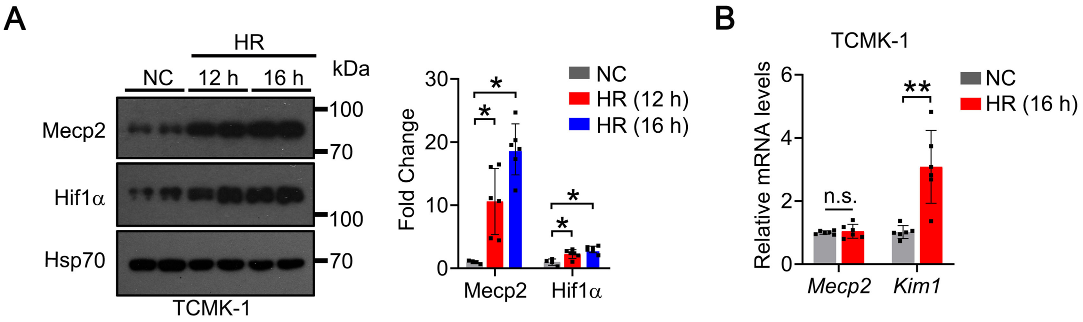

Figure S3. Mecp2 expression in TCMK-1 cells. Related to Figure 1. **(A)** Levels of Mecp2 and Hif1α in TCMK-1 cells after hypoxia for 12 h or 16 h and reperfusion for 1 h (HR 12 h, HR 16 h) injury (left) with quantitative results (right). **(B)** mRNA levels of *Mecp2* and *Kim1* in TCMK-1 cells after hypoxia for 16 h and reperfusion for 1 h (HR 16 h) injury. At least three biological replicates per group were used for these experiments. NC, non-injured cells; HR, HR injured cells; **P* < 0.05; ***P* < 0.01; n.s., not significant.

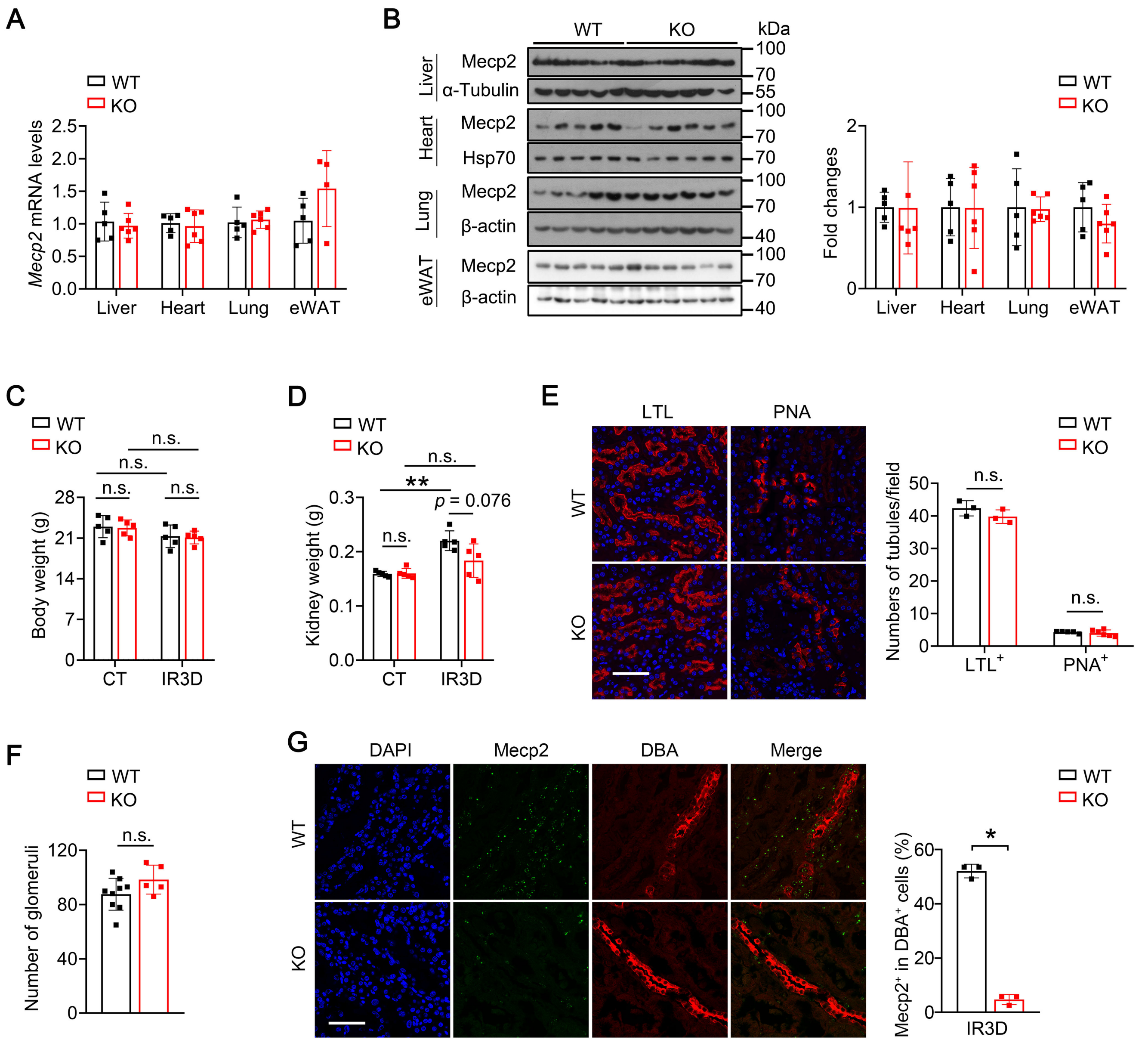

Figure S4. *Mecp2^{ksp}* KO mice had similar renal morphology compared to the WT mice. Related to Figure 2. **(A)** mRNA levels of *Mecp2* in the liver, heart, lung, and eWAT of the WT and *Mecp2^{ksp}* KO mice. **(B)** Protein levels of Mecp2 in the liver, heart, lung, and eWAT of the WT and *Mecp2^{ksp}* KO mice (left) with quantitative results (right). **(C-D)** Body weights **(C)** and kidney weights **(D)** of WT and *Mecp2^{ksp}* KO mice before and after IR3D injury. **(E)** Representative images of LTL (red; lotus

tetragonolobus lectin) and PNA (red; peanut agglutinin) staining (left) with quantitative results (right) in the kidney of WT and *Mecp2^ksp^* KO mice. **(F)** Number of glomeruli in the kidneys of WT and *Mecp2^ksp^* KO mice. **(G)** Co-immunofluorescent staining for Mecp2 (green; left) and DBA (red; left) with quantitative results (right) in the kidney of WT and *Mecp2^ksp^* KO mice at IR3D. DAPI (blue) stained nuclei. Scale bar = 50 μm. n = 3-9 per group. $^{*}P < 0.05$; $^{**}P < 0.01$; n.s., not significant.

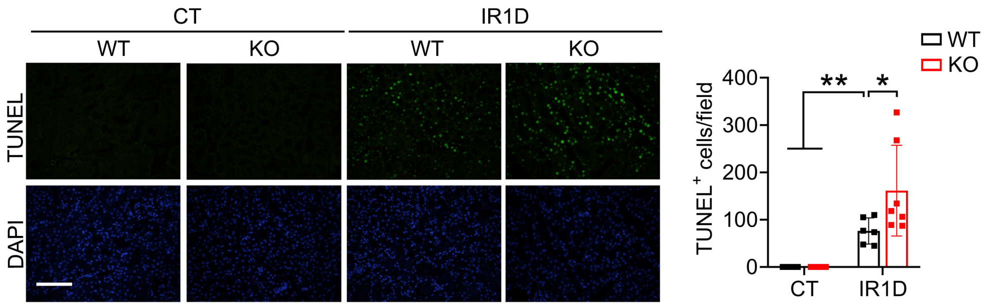

Figure S5. Renal tubular specific knockout of Mecp2 exacerbates cell death at IR1D. Related to Figure 3. Representative TUNEL images of WT and *Mecp2*^{ksp} KO mice at 1 day after IR injury (IR1D, left), with quantification results of TUNEL^{+} cells (right). DAPI (blue) stained nuclei. Scale bar = 100 µm. n = 5-7 per group. **P* < 0.05; ***P* < 0.01.

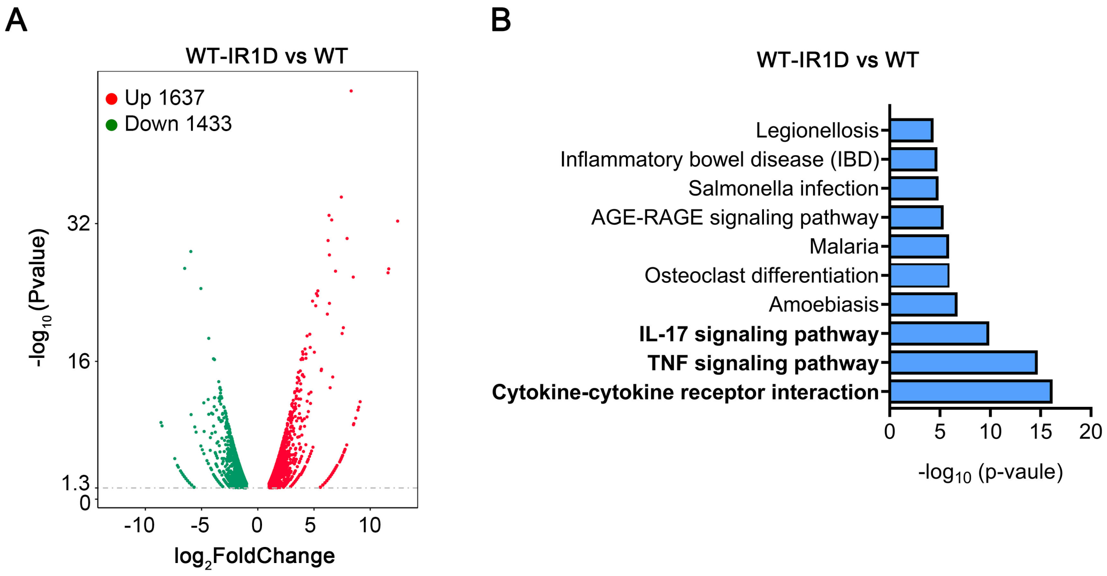

Figure S6. RNA-sequencing analysis in the kidney of WT mice at one day after IR injury. Related to Figure 4. **(A)** Volcanic map of altered genes in the WT mice of injured kidney at day one after injury (IR1D) *vs.* non-injured kidney. **(B)** Top 10 KEGG pathways for the differentially upregulated genes enriched in the WT mice of injured kidney at day one after injury (IR1D) *vs.* non-injured kidney.

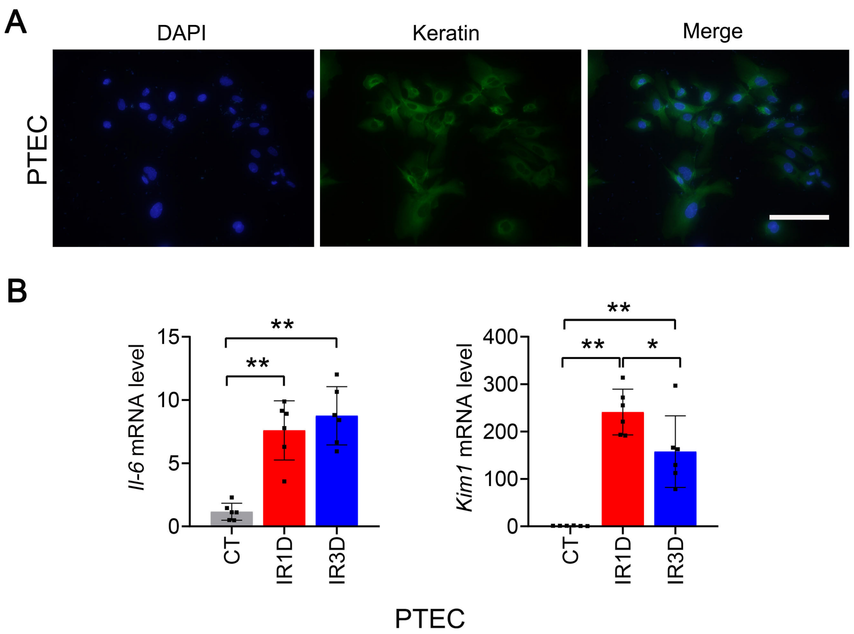

Figure S7. *Il-6* and *Kim1* gene levels in mouse primary tubular epithelial cells (mPTEC) at day one or day three after IR injury. Related to Figure 4. **(A)** Representative immunochemical staining for pan-keratin (green) in isolated mPTEC of WT mice. DAPI (blue) stained nuclei. Scale bar = 100 μm. **(B)** *Il-6* or *Kim1* gene level in isolated PTEC of WT mice at day 1 (IR1D) and day 3 (IR3D) after the IR injury. n = 6 per group. *$P < 0.05$; **$P < 0.01$.

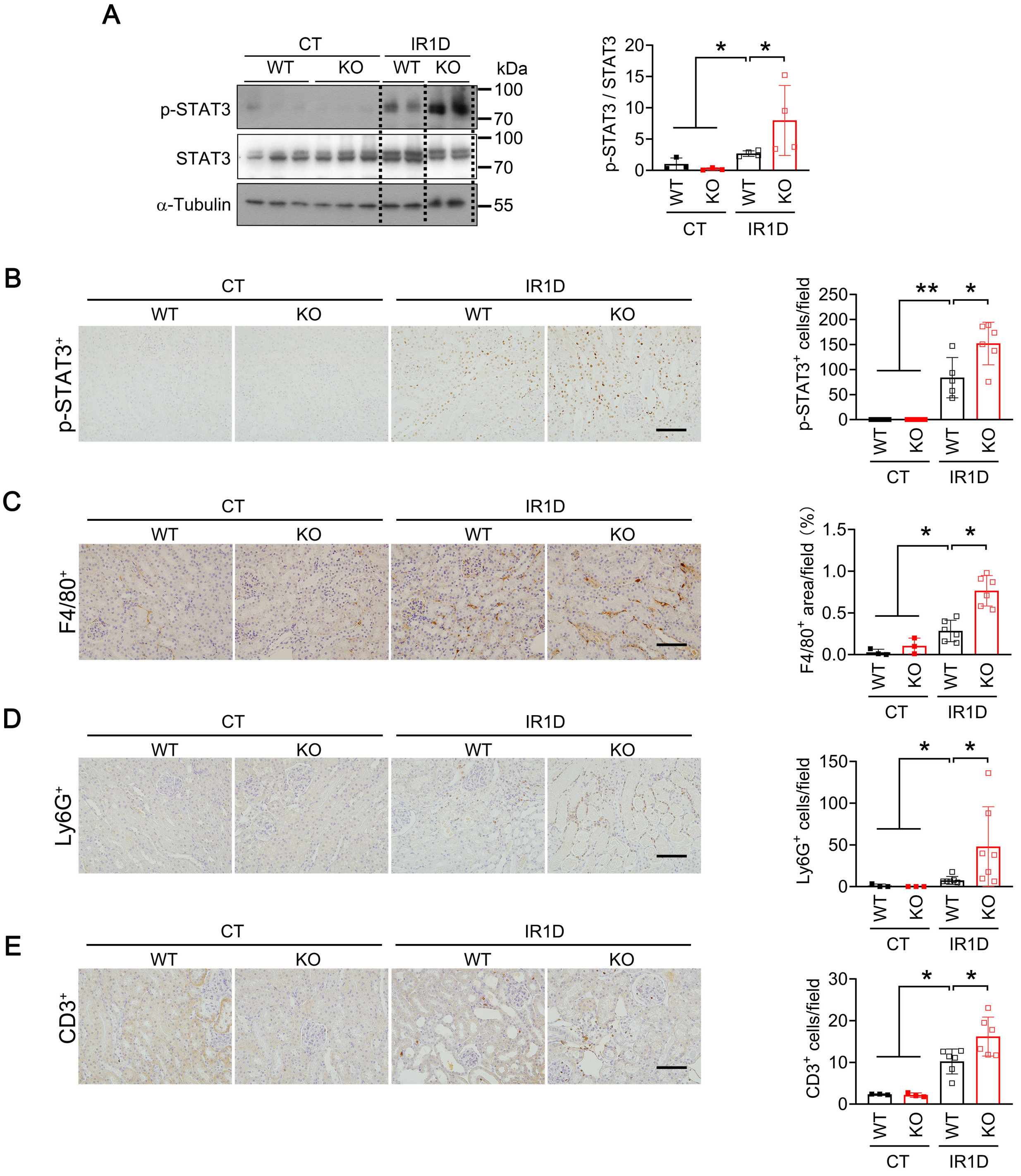

Figure S8. Renal tubular specific knockout of Mecp2 increases STAT3 activation and immune cell infiltration. Related to Figure 5. **(A)** Levels of p-STAT3 and STAT3 in the kidney of WT and *Mecp2*^{ksp}

KO mice (left) with quantitative results (right) at one day after IR injury (IR1D). **(B)** Representative immunochemical staining for p-STAT3 in the kidneys of WT and *Mecp2*^{ksp} KO mice (left) and quantitative results (right) at IR1D. Brown color indicates positive staining; scale bar = 100 μm. **(C-E)** Representative immunostaining for F4/80 **(C)**, Ly6G **(D)**, and CD3 **(E)** of the kidney of WT and *Mecp2*^{ksp} KO mice before and at day 1 after the IR injury (left) and quantitative results (right). Brown color indicates positive staining; scale bar = 100 μm. n = 3-7 per group. *$P < 0.05$; **$P < 0.01$.

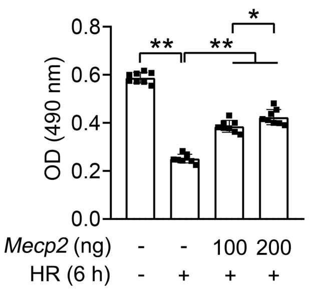

Figure S9. Overexpression of Mecp2 protected NRK52E cells from HR injury. MTT assay for indicated *Mecp2* dosage on cells after hypoxia for 6 h and reperfusion for 1 h (HR 6h) injury. The experiments were repeated for three times, and at least three biological replicates per group were used. *P < 0.05; **P < 0.01.

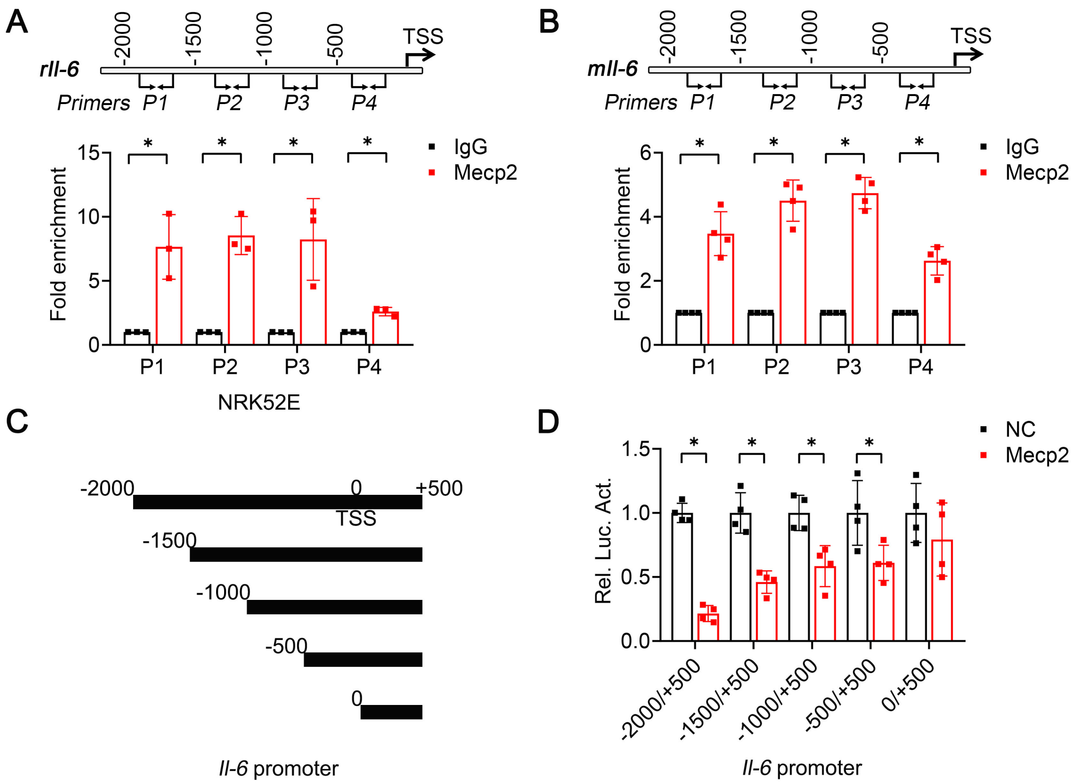

Figure S10. Mecp2 binds to the *Il-6* promoter and transcriptionally regulates *Il-6* level. Related to Figure 6. **(A-B)** Enrichment of Mecp2 on the promoter of *Il-6* in NRK52E cells **(A)** and in mouse kidney **(B)**. **(C)** Illustration of different constructions of *Il-6* promoter regions used for luciferase reporter assays. **(D)** Luciferase reporter assay results for NRK52E cells with different constructions of *Il-6,* together with *Mecp2* or the control vector. The experiments were repeated for three times, and at least three biological replicates per group were used. *$P < 0.05$.

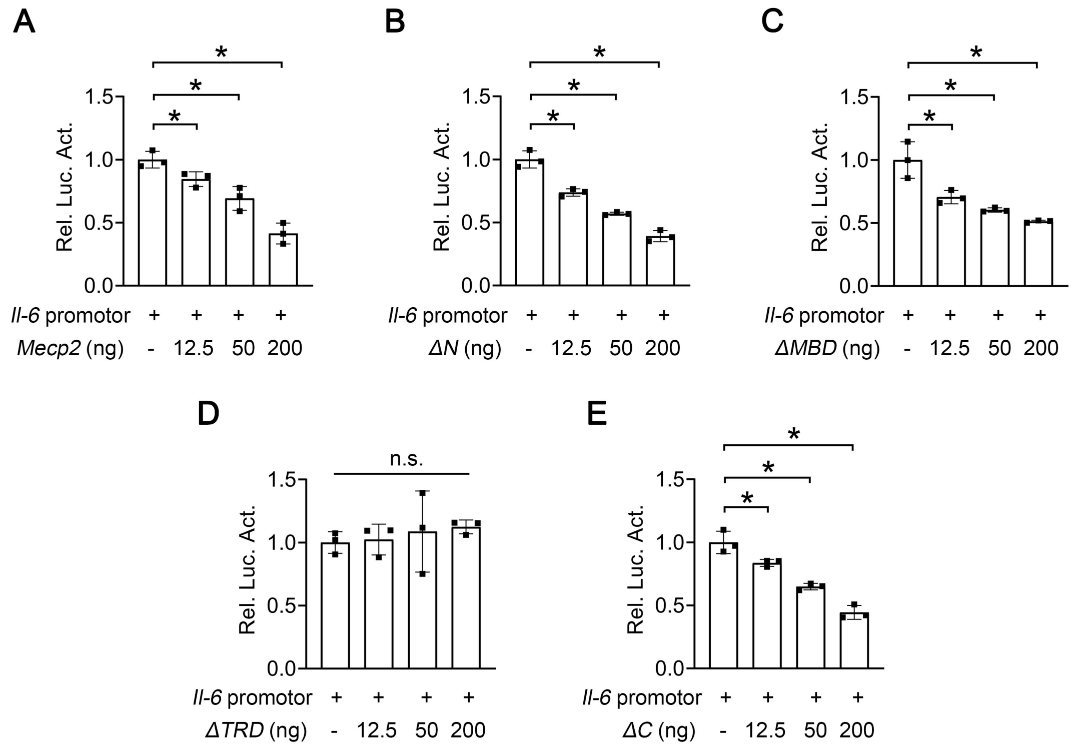

Figure S11. Transcriptional regulation of *Il-6* by full-length Mecp2 and its four domain-deletion mutants. Related to Figure 6. **(A-E)** Luciferase assays indicate dosage-dependent effects of full-length Mecp2 **(A)**, Mecp2-△N **(B)**, Mecp2-△MBD **(C)**, Mecp2-△TRD **(D)**, and Mecp2-△C **(E)** on *Il-6* promoter in NRK52E cells (2×10^4) (pGL3-enhancer-*Il-6* (50 ng), pRL-TK (renilla luciferase reporter plasmid, 20 ng)). At least three biological replicates per group were used for these experiments. *$P < 0.05$; n.s., not significant.

Table S1. Primers used in the present study.

Gene	Forward	Reverse
**(1) qPCR primer sequences**		
M/R *Mecp2*	GGAGGCGAGGAGGAGAGAC	ATGGTGGGCTGAAGGTTGTA
M *Kim1*	CAGGGTCTCCTTCACAGCAG	CGCCTGGGAGAAGCAGTATG
M *Il-6*	CACTTCACAAGTCGGAGGCT	CTGCAAGTGCATCATCGTTGT
M *Fos*	TACTACCATTCCCCAGCCGA	GCTGTCACCGTGGGGATAAA
M *Socs3*	GCCTTTCAGTGCAGAGTAGTG	AAGAGCAGGCGAGTGTAGAG
M *Jun*	GCACATCACCACTACACCGA	GGGAAGCGTGTTCTGGCTAT
M *Il33*	GGGCTCACTGCAGGAAAGTA	TTTGCCGGGGAAATCTTGGA
M *Il34*	TTGCTGTAAACAAAGCCCCAT	CCGAGACAAAGGGTACACATTT
M *Cx3cl1*	CTCACGAATCCCAGTGGCTT	TTTCTCCTTCGGGTCAGCAC
M *Il12a*	TGGATCTGAGCTGGACCCTT	TATCCTTGGATGTGCACGGT
M *Cxcl10*	ATGACGGGCCAGTGAGAATG	TCAACACGTGGGCAGGATAG
M *Mmp9*	AAAGGCAGCGTTAGCCAGAA	GGTCTTTGGGGAAGACCACA
M *Tnfα*	GACGTGGAACTGGCAGAAGAG	ACCGCCTGGAGTTCTGGAA
M *Ccl3*	GCCACATCGAGGGACTCTTC	GATGGGGGTTGAGGAACGTG
M *Tgfb1*	TGGCCAGATCCTGTCCAAAC	GTTGTACAAAGCGAGCACCG
M *Tgfb2*	AACACCCTCTGGCTCATTGG	TGTAGAAAGTGGGCGGGATG
M *Tgfb3*	GGACTTCGGCCACATCAAGA	ATAGGGGACGTGGGTCATCA

M *Smad3*	CCGTCAGTCCGTCGGTCC	GCCCGAACTTCGCTTTTAACT
M *Fn1*	GCCTGAACCAGCCTACAGAT	AGCTTAAAGCCAGCGTCAGA
M *Vimentin*	AGCAGTATGAAAGCGTGGCT	ACCTGTCTCCGGTACTCGTT
M *Col1a1*	CGATGGATTCCCGTTCGAGT	CATTAGGCGCAGGAAGGTCA
M *Col3a1*	ACGTAAGCACTGGTGGACAG	CAGGAGGGCCATAGCTGAAC
M *Col4a1*	TCATTAGCAGGTGTGCGGTT	GTTAGGGCACTGCGGAATCT
M *a-SMA*	TCCAGCCATCTTTCATTGGGA	CCCCTGACAGGACGTTGTTA
R *Kim1*	CTCCAGGAAGCCGAGCAAAC	AAGCACTGGGTACAGATCCAAA
R *Il-6*	AGCCAGAGTCATTCAGAGCAA	AGAGCATTGGAAGTTGGGGT
R *Fos*	TACTACCATTCCCCAGCCGA	GCTGTCACCGTGGGGATAAA
R *Jun*	GCACATCACCACTACACCGA	TATGCAGTTCAGCTAGGGCG
R *Socs3*	CCCCGCTTTGACTGTGTACT	AAAGGAAGGTTCCGTCGGTG
H/R/M *Rn18s*	CTCAACACGGGAAACCTCAC	CGCTCCACCAACTAAGAACG
**(2) ChIP primers used in the present study**		
R *Il-6-P1*	GGCACCACTTGTAGGACCAT	ACTCCCTGCCATGTGTTCTC
R *Il-6-P2*	TGCCATTTTAAGGTCCAAGG	TGGGTGGACTCTTGTTTGTG
R *Il-6-P3*	TGGAAATGTTTTGGGGATGT	CAAGCACTCCTGGTTTCCTC
R *Il-6-P4*	AAACACACTTTCCCCCTCCT	TCATGGGAAAATCCCACATT
M *Il-6-P1*	TCCCCATTTTCATTTTCACC	AAAGCCGGTTGATTCTTGTG

M *Il-6-P2*	GCCTACTTTCAGCCTGGAATC	TAACCCCTCCAATGCTCAAG
M *Il-6-P3*	GACTTGGAAGCCAAGATTGC	ACCCAACCTGGACAACAGAC
M *Il-6-P4*	AAGCACACTTTCCCCTTCCT	TCATGGGAAAATCCCACATT
**(3) primer sequences used for genotyping**		
Mecp2-flox	TGGTAAAGACCCATGTGACCCAA	GGCTTGCCACATGACAAGAC
Ksp-cre	GCAGATCTGGCTCTCCAAAG	AGGCAAATTTTGGTGTACGG
**(4) sequences of shRNA used in the present study**		
Scramble (NC)	5'-CCTAAGGTTAAGTCGCCCTCG-3'	
Mecp2#1	5'-GCTGGAAAGTATGATGTATAT-3'	
Mecp2#2	5'-GCATCTGCAAAGAAGAGAAGA-3'	

M: *Mus musculus*; H: *Homo sapiens*; R: *Rattus norvegicus*.

Table S2. Antibodies used in the present study.

Antigen	Vendor	Catalog number	Dilution
MECP2 (WB)	Cell Signaling Technology	#3456s	1:1000
MECP2 (IHC, IF)	Cell Signaling Technology	#3456s	1:500
MECP2 (ChIP assay)	Abcam	ab2828	1:100
Hif1α (WB)	Abcam	ab113642	1:1000
p-STAT3 (WB)	Cell Signaling Technology	#9131s	1:1000
p-STAT3 (IHC)	Cell Signaling Technology	#9145s	1:100
STAT3 (WB)	Cell Signaling Technology	#4904s	1:1000
CD3 (IHC)	Gene Tech	130914	1:100
Ly6G (IHC)	BD Biosciences	BD551459	1:100
F4/80 (IHC)	Santa Cruz	sc-52664	1:200
Pan-keratin (IHC)	Cell Signaling Technology	#4545	1:200
α-SMA (IHC)	Sigma	A2547	1:5000
Flag (WB)	Sigma	F7425	1:10000
β-actin (WB)	Sigma	AC-40	1:10000
α-tubulin (WB)	Beyotime	AF0001	1:10000
Hsp70 (WB)	BD Biosciences	BD610607	1:10000
IgG (ChIP)	Cell Signaling Technology	#2729s	1:100

Table S3: Differential expressed genes of WT_IR1D vs WT

gene_name	WT_IR1D readcount	WT readcount	log2FoldChange
Gm23332	0.00	48.75	-8.61
Gm9780	0.00	45.63	-8.52
Gm31107	0.00	20.74	-7.38
Gm25386	0.00	17.63	-7.15
Gm24515	0.00	16.60	-7.06
BC048644	0.00	14.52	-6.87
Gm13259	0.00	13.48	-6.77
Gm32817	0.00	12.45	-6.65
Cntnap5a	0.00	12.45	-6.65
9130214F15Rik	0.00	12.45	-6.65
Gm20485	0.00	11.41	-6.53
Gm44939	0.00	11.41	-6.53
Adam7	0.00	11.41	-6.53
Klk1b21	0.00	11.41	-6.53
Pvalb	8.68	797.51	-6.50
Kank4os	0.00	10.37	-6.39
Gm33050	0.00	10.37	-6.39
Rtl4	0.00	10.37	-6.39
4933429O19Rik	0.00	10.37	-6.39
Trim31	0.00	10.37	-6.39
Gm26894	0.00	10.37	-6.39
1700125G22Rik	0.00	10.37	-6.39
Gm45650	0.00	10.37	-6.39
Pdxk-ps	0.00	9.34	-6.24
Gm6878	0.00	9.34	-6.24
Il11ra2	0.00	9.34	-6.24
Gm5300	0.00	9.34	-6.24
Gm28154	0.00	9.34	-6.24
Gm10388	0.00	9.34	-6.24
Gm9871	0.00	9.34	-6.24
Cib3	0.00	9.34	-6.24
Gm2814	0.00	9.34	-6.24
Cyp4b1-ps2	0.00	9.34	-6.24
Tekt5	0.00	8.30	-6.08
Cyp2j15-ps	0.00	8.30	-6.08
Ctcfl	0.00	8.30	-6.08
Gm19439	0.00	8.30	-6.08
Gm27415	0.00	8.30	-6.08
Gm10075	0.00	8.30	-6.08
Duox1	0.00	8.30	-6.08
Gm43134	0.00	8.30	-6.08
Fam196b	0.00	8.30	-6.08
Gm42109	0.00	8.30	-6.08
Gm27457	0.00	8.30	-6.08
Gm43409	0.00	8.30	-6.08
Nccrp1	49.17	3058.32	-5.96
Gm29012	0.96	66.38	-5.93
Actn2	0.00	7.26	-5.89
Gm31126	0.00	7.26	-5.89
Rpl36-ps3	0.00	7.26	-5.89
Gm45540	0.00	7.26	-5.89
Gm28802	0.00	7.26	-5.89
Gm15473	0.00	7.26	-5.89
Hrh3	0.00	7.26	-5.89
Gm37268	0.00	7.26	-5.89
Gm2602	0.00	7.26	-5.89
Gm42567	0.00	7.26	-5.89
Kbtbd12	0.00	7.26	-5.89

Gm29629	0.00	7.26	-5.89
Gm14142	0.00	7.26	-5.89
Gm13267	0.00	7.26	-5.89
Vamp9	0.00	7.26	-5.89
Gm44167	0.00	7.26	-5.89
Gm9682	0.00	7.26	-5.89
1700001C19Rik	0.00	7.26	-5.89
Gm15912	0.00	6.23	-5.67
2010203P06Rik	0.00	6.23	-5.67
Gm32401	0.00	6.23	-5.67
Vstm2l	0.00	6.23	-5.67
Gm37817	0.00	6.23	-5.67
AC158982.1	0.00	6.23	-5.67
Gm11716	0.00	6.23	-5.67
Gm20900	0.00	6.23	-5.67
Gm11629	0.00	6.23	-5.67
CT025867.1	0.00	6.23	-5.67
Gm23622	0.00	6.23	-5.67
Igkv2-112	0.00	6.23	-5.67
Gm15494	0.00	6.23	-5.67
Gm27463	0.00	6.23	-5.67
Gm24613	0.00	6.23	-5.67
Slain1os	0.00	6.23	-5.67
4930444A19Rik	0.00	6.23	-5.67
Gm27027	0.00	6.23	-5.67
Ifi44l	0.00	6.23	-5.67
Myh1	0.00	6.23	-5.67
Gm28905	0.00	6.23	-5.67
Odf4	0.96	52.89	-5.61
Adamtsl3	0.96	48.75	-5.49
Gm12326	0.96	36.30	-5.06
Egf	877.47	29289.91	-5.06
Sycn	0.96	33.19	-4.93
1110019B22Rik	3.85	110.97	-4.80
Ccdc170	0.96	30.08	-4.79
Gm33543	1.93	56.00	-4.77
AC130815.1	0.96	24.89	-4.52
Gm4208	6.75	146.23	-4.41
Sost	0.96	22.82	-4.40
Gm48287	0.96	22.82	-4.40
Pappa2	4.82	101.64	-4.36
Slc4a1	107.99	2222.44	-4.36
5830473C10Rik	1.93	38.37	-4.23
Kcns1	2.89	56.00	-4.22
Gm47078	0.96	19.71	-4.19
Car5a	6.75	119.27	-4.12
Kcnq3	0.96	18.67	-4.11
Rph3a	3.85	67.41	-4.08
D630023O14Rik	7.71	125.49	-4.00
Atp4a	194.78	2996.09	-3.94
Lhfpl1	0.96	16.60	-3.94
Lrrc52	5.78	90.23	-3.93
Wfdc15b	236.24	3583.07	-3.92
Mfrp	10.60	157.64	-3.88
Grem1	15.43	225.05	-3.86
Calb1	978.72	14094.79	-3.85
Gm3953	0.96	15.56	-3.85
Paqr6	0.96	15.56	-3.85
Gm47708	16.39	235.42	-3.83
4930434B07Rik	2.89	41.49	-3.79
-	5.78	80.89	-3.78
Rln1	0.96	14.52	-3.75

Uncx	2.89	40.45	-3.75
2410003L11Rik	1.93	26.97	-3.72
Rp1	1.93	26.97	-3.72
C330021F23Rik	2.89	38.37	-3.67
-	13.50	173.19	-3.67
Gm45909	1.93	25.93	-3.67
Gm29201	0.96	13.48	-3.64
Grin2c	0.96	13.48	-3.64
Klkb1	4.82	61.19	-3.63
Gm15241	1.93	24.89	-3.61
Pdzrn4	2.89	36.30	-3.59
4930533I22Rik	5.78	68.45	-3.54
Gm11754	0.96	12.45	-3.53
AC140300.3	0.96	12.45	-3.53
Tcerg1l	0.96	12.45	-3.53
Slc16a7	571.80	6351.01	-3.47
Htr3a	14.46	161.79	-3.47
Hhatl	41.46	457.35	-3.46
Gm9973	14.46	157.64	-3.43
Cox6a2	5.78	63.26	-3.42
Gm44850	1.93	21.78	-3.42
1700003D09Rik	1.93	21.78	-3.42
Bfsp2	0.96	11.41	-3.40
Cyp2c38	0.96	11.41	-3.40
4930404H11Rik	0.96	11.41	-3.40
Gm37529	0.96	11.41	-3.40
Tmem207	41.46	438.68	-3.40
Car14	264.20	2757.57	-3.38
Dnah11	4.82	50.82	-3.36
Abca13	634.48	6520.05	-3.36
Kcnt1	82.92	847.29	-3.35
Wnt11	1.93	20.74	-3.35
C030005K06Rik	62.67	636.76	-3.34
Insrr	169.71	1694.57	-3.32
Dusp15	52.07	511.28	-3.29
Slc12a1	4437.51	43452.11	-3.29
Alms1-ps2	18.32	178.38	-3.27
Gm47585	0.96	10.37	-3.27
Gm16053	0.96	10.37	-3.27
Aox3	0.96	10.37	-3.27
Gm38197	0.96	10.37	-3.27
Gm12138	0.96	10.37	-3.27
Gm49024	0.96	10.37	-3.27
Bend6	0.96	10.37	-3.27
Kirrel3os	0.96	10.37	-3.27
Gm11945	0.96	10.37	-3.27
Gm28512	0.96	10.37	-3.27
Crygn	0.96	10.37	-3.27
Gm45234	0.96	10.37	-3.27
Mir874	0.96	10.37	-3.27
Ovol3	0.96	10.37	-3.27
Ighv14-1	0.96	10.37	-3.27
Gm7701	0.96	10.37	-3.27
Slc22a22	188.03	1789.98	-3.25
Fmod	21.21	201.19	-3.24
Scn4b	241.06	2219.33	-3.20
Nptx1	1.93	18.67	-3.19
Grin3a	9.64	89.19	-3.19
Dok7	4.82	44.60	-3.18
Phyhip	26.03	233.34	-3.16
6330410L21Rik	3.85	35.26	-3.15
Gm45170	40.50	357.79	-3.14

Shd	47.25	412.76	-3.12
Gm19531	0.96	9.34	-3.12
Gm49495	0.96	9.34	-3.12
B230322F03Rik	0.96	9.34	-3.12
Tlx2	0.96	9.34	-3.12
Gm46378	0.96	9.34	-3.12
Ces3a	0.96	9.34	-3.12
AC140346.1	0.96	9.34	-3.12
2200002J24Rik	0.96	9.34	-3.12
Gm40438	0.96	9.34	-3.12
Ighv1-78	0.96	9.34	-3.12
Neurog2	4.82	41.49	-3.07
Ltf	2.89	24.89	-3.05
AC161607.1	1.93	16.60	-3.03
4933417D19Rik	1.93	16.60	-3.03
Colec11	1.93	16.60	-3.03
Trpm1	1.93	16.60	-3.03
Ppp1r1b	268.06	2180.96	-3.02
Tmem61	49.17	399.27	-3.02
-	5.78	47.71	-3.02
Fam107a	380.88	3034.46	-2.99
Mapk8ip2	2.89	23.86	-2.99
Gm29674	2.89	23.86	-2.99
Spr-ps1	8.68	69.49	-2.98
Mymx	13.50	105.78	-2.96
Slc22a27	1.93	15.56	-2.93
Fmo3	1.93	15.56	-2.93
Cyp4f15	1.93	15.56	-2.93
Rhcg	666.30	4989.34	-2.90
Pgam2	265.17	1981.84	-2.90
Gm11734	4.82	36.30	-2.88
Vstm2a	40.50	297.64	-2.87
Alms1-ps1	2.89	21.78	-2.86
Gm27202	13.50	98.52	-2.86
Gm29216	972.93	7031.32	-2.85
Slc2a4	171.63	1240.34	-2.85
G6pc	1732.77	12372.22	-2.84
Lmod3	1.93	14.52	-2.83
Gm11775	1.93	14.52	-2.83
Cldnd2	1.93	14.52	-2.83
Glns-ps1	1.93	14.52	-2.83
Kctd8	1.93	14.52	-2.83
Hsd17b14	41.46	295.57	-2.83
Rnf212b	7.71	54.97	-2.81
Egfl6	85.82	599.43	-2.80
Sfrp1	642.19	4458.36	-2.80
Rhbg	66.53	462.54	-2.80
C8a	101.24	702.10	-2.79
Gm4956	25.07	174.23	-2.79
Gm44065	2.89	20.74	-2.79
4930556J24Rik	7.71	53.93	-2.79
Gm26685	7.71	52.89	-2.76
Gm42872	7.71	52.89	-2.76
Gm47951	4.82	33.19	-2.75
Acot3	184.17	1239.30	-2.75
BC024386	593.98	3973.01	-2.74
Gm17025	6.75	45.63	-2.73
Adgrg4	13.50	90.23	-2.73
Gm26588	7.71	51.86	-2.73
A930017M01Rik	1.93	13.48	-2.73
Col8a2	1.93	13.48	-2.73
Mir681	1.93	13.48	-2.73

Gm7842	1.93	13.48	-2.73
1700025N23Rik	1.93	13.48	-2.73
E330021D16Rik	1.93	13.48	-2.73
2900009J06Rik	2.89	19.71	-2.72
Etnppl	74.24	488.46	-2.72
Gm49272	3.85	25.93	-2.71
Gm44120	201.53	1309.82	-2.70
Bhmt	20.25	130.67	-2.68
Shisal1	11.57	74.67	-2.68
Prss12	9.64	62.23	-2.67
1700028J19Rik	59.78	380.61	-2.67
Itih2	27.00	172.16	-2.67
Rasd2	27.96	177.34	-2.66
Cyp2c68	3.85	24.89	-2.65
-	3.85	24.89	-2.65
Mep1b	528.41	3297.88	-2.64
R3hdml	17.35	108.90	-2.64
Matn4	12.53	78.82	-2.64
Lrrc34	2.89	18.67	-2.64
Chrne	2.89	18.67	-2.64
Igkv9-124	2.89	18.67	-2.64
Mylk3	9.64	60.15	-2.63
Tmem52	33.75	207.42	-2.62
Nr0b2	14.46	89.19	-2.61
Rnf224	1.93	12.45	-2.61
Lrrc17	1.93	12.45	-2.61
Pianp	1.93	12.45	-2.61
Gm7488	1.93	12.45	-2.61
Hspe1-ps6	1.93	12.45	-2.61
Gm25193	1.93	12.45	-2.61
Gucy2f	1.93	12.45	-2.61
Mroh4	1.93	12.45	-2.61
Mir5620	1.93	12.45	-2.61
Pax6	1.93	12.45	-2.61
Klhl3	459.95	2813.57	-2.61
Ell3	4.82	30.08	-2.61
10-Mar	4.82	30.08	-2.61
Nepn	73.28	448.02	-2.61
4930512B01Rik	13.50	82.97	-2.61
Hpd	450.31	2740.97	-2.61
Slc8a1	299.88	1824.21	-2.60
Ryr2	6.75	41.49	-2.60
Gm13262	16.39	99.56	-2.59
Nkx3-1	3.85	23.86	-2.59
Vsig8	3.85	23.86	-2.59
Clnk	3.85	23.86	-2.59
D630024D03Rik	51.10	308.01	-2.59
Myadml2os	5.78	35.26	-2.58
Cyp2d26	1354.78	8060.09	-2.57
Unc13c	2.89	17.63	-2.56
Gm12927	2.89	17.63	-2.56
Gm15462	2.89	17.63	-2.56
Vmn1r19	2.89	17.63	-2.56
Xpnpep2	64.60	380.61	-2.56
Gm28023	12.53	73.63	-2.54
Gm13110	12.53	73.63	-2.54
Cyp7b1	836.01	4847.26	-2.54
Cwh43	158.14	914.70	-2.53
Corin	22.17	128.60	-2.53
Slc6a12	41.46	239.57	-2.53
Elmod1	3.85	22.82	-2.53
Gm13111	171.63	989.37	-2.53

Cacnb4	107.99	621.21	-2.52
Hcar1	9.64	56.00	-2.52
Vpreb1	9.64	56.00	-2.52
Pex5l	15.43	89.19	-2.52
Uroc1	390.52	2241.11	-2.52
Tmem52b	1553.42	8901.16	-2.52
Baiap3	13.50	77.78	-2.52
Cr2	10.60	61.19	-2.51
Cox7a1	76.17	432.46	-2.50
Kcnj1	1618.98	9149.02	-2.50
Mecomos	5.78	33.19	-2.49
Gm11752	5.78	33.19	-2.49
2210418O10Rik	5.78	33.19	-2.49
Sag	1.93	11.41	-2.49
C030010L15Rik	1.93	11.41	-2.49
Slc6a7	1.93	11.41	-2.49
Gm9797	1.93	11.41	-2.49
Sapcd1	1.93	11.41	-2.49
Gm26550	1.93	11.41	-2.49
Gm11627	1.93	11.41	-2.49
Bsnd	491.77	2728.53	-2.47
Ralyl	2.89	16.60	-2.47
Gm9003	2.89	16.60	-2.47
Pnliprp1	2.89	16.60	-2.47
Gm10109	23.14	128.60	-2.47
Acacb	388.59	2131.18	-2.45
Lgr6	4.82	26.97	-2.45
Gm5096	4.82	26.97	-2.45
Gm44421	39.53	216.75	-2.45
1-Mar	6.75	37.34	-2.45
-	9.64	52.89	-2.44
Zfp648	15.43	84.01	-2.44
Wdr86	16.39	89.19	-2.43
Fndc9	20.25	109.93	-2.43
-	66.53	359.87	-2.43
-	117.64	634.69	-2.43
Asb11	31.82	170.08	-2.41
Gm45727	7.71	41.49	-2.41
AC162305.2	6.75	36.30	-2.41
Camk1g	18.32	97.49	-2.40
Anxa13	16.39	87.12	-2.40
Pde6a	14.46	76.75	-2.40
9430037G07Rik	13.50	71.56	-2.40
Fads6	291.20	1528.64	-2.39
Gm4609	3.85	20.74	-2.39
Cpxm2	3.85	20.74	-2.39
Gm19434	2.89	15.56	-2.38
Gm16157	2.89	15.56	-2.38
Klk1b22	2.89	15.56	-2.38
Gm12735	2.89	15.56	-2.38
Ccl19-ps3	2.89	15.56	-2.38
Fabp1	86.78	450.09	-2.37
1810041H14Rik	7.71	40.45	-2.37
Gm45051	7.71	40.45	-2.37
Wscd2	69.42	358.83	-2.37
Scara5	21.21	109.93	-2.37
Snx31	48.21	248.90	-2.37
Sv2a	266.13	1368.93	-2.36
Ppp1r1a	979.68	5006.97	-2.35
1110018N20Rik	4.82	24.89	-2.34
Flg	4.82	24.89	-2.34
Gpx6	274.81	1388.64	-2.34

Gm13412	7.71	39.41	-2.33
Ranbp3l	337.49	1701.83	-2.33
Cyp2d12	719.33	3618.33	-2.33
Ces2h	17.35	87.12	-2.32
C1qtnf3	378.95	1888.50	-2.32
Trim61	3.85	19.71	-2.32
Ipcef1	103.17	513.35	-2.31
Acmsd	1316.21	6533.53	-2.31
Ces1d	1334.53	6611.31	-2.31
Cyp2d9	2315.18	11460.64	-2.31
Lrrc15	10.60	52.89	-2.30
Perm1	48.21	238.53	-2.30
Ano4	5.78	29.04	-2.30
Chrna4	615.19	3035.50	-2.30
4933417A18Rik	12.53	62.23	-2.30
Tdg-ps	7.71	38.37	-2.30
Tmem86a	156.21	765.36	-2.29
Afm	201.53	986.25	-2.29
Akr1c21	5873.29	28594.04	-2.28
Gm9725	2.89	14.52	-2.28
Luzp2	2.89	14.52	-2.28
Gm44249	2.89	14.52	-2.28
Gm20456	2.89	14.52	-2.28
Gm48702	4.82	23.86	-2.28
Fancd2os	6.75	33.19	-2.28
Cdkl1	606.51	2927.65	-2.27
Spag5	40.50	194.97	-2.26
Akr1c14	779.12	3736.56	-2.26
Atp6v1c2	179.35	858.70	-2.26
-	69.42	331.86	-2.25
Cyp27b1	269.99	1287.00	-2.25
Fam69b	24.10	115.12	-2.25
Cited4	38.57	183.56	-2.25
Nat8f1	1877.41	8895.97	-2.24
Aqp3	1049.11	4964.45	-2.24
Ms4a2	3.85	18.67	-2.24
1700112J16Rik	3.85	18.67	-2.24
Cadm2	3.85	18.67	-2.24
Abca8a	143.67	678.25	-2.24
Pcsk6	273.85	1291.15	-2.24
Gys2	60.75	286.23	-2.23
Gm8765	6.75	32.15	-2.23
AI197445	9.64	45.63	-2.23
Hsd3b2	747.30	3489.74	-2.22
Gm44165	4.82	22.82	-2.21
D630029K05Rik	687.51	3179.65	-2.21
Ntrk1	8.68	40.45	-2.20
Gm906	23.14	106.82	-2.20
Wnt9b	24.10	110.97	-2.20
Vwa3a	5.78	26.97	-2.20
Kcne1	171.63	786.10	-2.19
4930461G14Rik	28.92	132.75	-2.19
Cyp2c69	6.75	31.12	-2.18
Art4	32.78	149.34	-2.18
Lrrc66	77.14	350.53	-2.18
AI314278	721.26	3261.58	-2.18
A930038B10Rik	7.71	35.26	-2.17
Gcgr	287.35	1297.37	-2.17
Gm44702	2.89	13.48	-2.17
Actl9	2.89	13.48	-2.17
Gm49328	2.89	13.48	-2.17
Gm18666	2.89	13.48	-2.17

Mir1192	2.89	13.48	-2.17
Clec2h	456.09	2054.43	-2.17
Gm45847	8.68	39.41	-2.17
Inmt	1528.34	6832.21	-2.16
Casr	449.34	2005.69	-2.16
Stpg1	3.85	17.63	-2.16
Pnck	3.85	17.63	-2.16
1700119H24Rik	3.85	17.63	-2.16
Gm5743	3.85	17.63	-2.16
Gm30191	3.85	17.63	-2.16
Kcng4	3.85	17.63	-2.16
Oxgr1	240.10	1068.18	-2.15
Olfr1396	4.82	21.78	-2.15
Gm36372	4.82	21.78	-2.15
H2-Bl	4.82	21.78	-2.15
A830008E24Rik	4.82	21.78	-2.15
C1qtnf2	23.14	102.67	-2.14
Slc7a13	1744.34	7694.01	-2.14
Gm17597	5.78	25.93	-2.14
Pld6	5.78	25.93	-2.14
9030619P08Rik	58.82	259.27	-2.14
Rimkla	17.35	76.75	-2.14
Kcng1	7.71	34.23	-2.13
Frmpd3	8.68	38.37	-2.13
Rufy4	27.00	118.23	-2.13
Aldoc	132.10	568.32	-2.10
Syn2	50.14	215.71	-2.10
Hsd11b2	1261.25	5390.68	-2.10
Slc34a3	379.91	1624.05	-2.10
Malrd1	114.74	489.50	-2.09
Itm2a	9.64	41.49	-2.09
Prom2	263.24	1120.04	-2.09
Gm20646	7.71	33.19	-2.09
Col19a1	6.75	29.04	-2.08
Rapsn	32.78	138.97	-2.08
Slc22a29	98.35	415.87	-2.08
Slc26a10	30.85	130.67	-2.08
Erich6b	4.82	20.74	-2.08
-	4.82	20.74	-2.08
Cacna1e	4.82	20.74	-2.08
Npb	4.82	20.74	-2.08
Sec14l4	4.82	20.74	-2.08
-	28.92	122.38	-2.08
Nlrp6	1497.49	6305.38	-2.07
AC166995.1	15.43	65.34	-2.07
Acss3	78.10	328.75	-2.07
Lypd2	3.85	16.60	-2.07
Fam243	13.50	57.04	-2.07
Gm8947	11.57	48.75	-2.06
B230206L02Rik	17.35	72.60	-2.06
-	9.64	40.45	-2.05
Phf24	9.64	40.45	-2.05
9630013D21Rik	43.39	180.45	-2.05
Gatm	6192.46	25636.32	-2.05
Ccdc151	39.53	163.86	-2.05
Gm37336	14.46	60.15	-2.05
Ankrd65	13.50	56.00	-2.04
Espnl	7.71	32.15	-2.04
Gm10658	24.10	99.56	-2.04
Slc2a5	656.66	2701.56	-2.04
Gm10705	52.07	213.64	-2.03
Nat8f6	496.59	2031.62	-2.03

-	16.39	67.41	-2.03
Tnn	26.03	106.82	-2.03
Klk1	13357.85	54579.85	-2.03
Batf3	24.10	98.52	-2.03
Htra4	10.60	43.56	-2.03
Gm15743	5.78	23.86	-2.02
Gm28793	5.78	23.86	-2.02
Gm24694	8.68	35.26	-2.01
Fgf11	45.32	182.53	-2.01
Gm22806	4.82	19.71	-2.00
Hapln1	4.82	19.71	-2.00
Plcd4	28.92	116.15	-2.00
A4gnt	107.99	432.46	-2.00
-	7.71	31.12	-1.99
Gjb6	20.25	80.89	-1.99
-	23.14	92.30	-1.99
Rec8	101.24	402.39	-1.99
Gm15889	132.10	524.76	-1.99
Chst9	13.50	53.93	-1.99
Lrrc31	31.82	126.53	-1.99
Cyp2j11	1259.32	4987.26	-1.99
Adssl1	59.78	236.45	-1.98
Kcnn3	9.64	38.37	-1.98
Efcc1	9.64	38.37	-1.98
Ntrk2	6.75	26.97	-1.98
Ism1	3.85	15.56	-1.98
Prss50	3.85	15.56	-1.98
Gm14002	3.85	15.56	-1.98
Tarm1	37.60	148.30	-1.98
Dpep1	991.25	3896.27	-1.97
Gm17546	17.35	68.45	-1.97
Slc22a28	756.94	2963.94	-1.97
AC140300.1	8.68	34.23	-1.96
Ttc36	631.59	2452.67	-1.96
Gpc5	5.78	22.82	-1.96
Akr1d1	335.56	1302.56	-1.96
Gm5475	25.07	97.49	-1.95
Gm17029	39.53	153.49	-1.95
Trim63	144.64	560.02	-1.95
Aldh3b3	22.17	86.08	-1.95
Gdf10	50.14	193.93	-1.95
Galnt11	5220.48	20113.93	-1.95
Gm11127	16.39	63.26	-1.94
Atp6v1b1	1168.68	4483.25	-1.94
Apoh	127.28	487.42	-1.94
Ogn	118.60	452.16	-1.93
Gm12678	164.88	627.43	-1.93
Gm42868	4.82	18.67	-1.93
Mir8116	4.82	18.67	-1.93
Gm27239	4.82	18.67	-1.93
Gm41349	4.82	18.67	-1.93
Gm31600	23.14	88.15	-1.92
Esrrb	429.09	1624.05	-1.92
Comp	8.68	33.19	-1.92
Ctnna2	8.68	33.19	-1.92
Slc16a4	823.47	3113.28	-1.92
Cyp2b10	12.53	47.71	-1.92
Gm4952	20.25	76.75	-1.92
Capn6	43.39	163.86	-1.91
Sall3	77.14	290.38	-1.91
Fam151a	242.03	909.51	-1.91
Dio1	155.24	581.80	-1.91

Miox	9536.50	35641.95	-1.90
Cntfr	70.39	263.42	-1.90
Aspa	995.11	3716.85	-1.90
Abca9	98.35	367.13	-1.90
E130215H24Rik	9.64	36.30	-1.90
Car12	3087.55	11501.09	-1.90
Pm20d1	818.65	3047.95	-1.90
Arhgef19	292.17	1087.89	-1.90
Gm48851	7.71	29.04	-1.90
Tyw3	211.17	782.99	-1.89
Gm11634	5.78	21.78	-1.89
Gm36864	14.46	53.93	-1.89
Angptl3	97.39	360.90	-1.89
Mpped1	41.46	153.49	-1.89
-	10.60	39.41	-1.88
Gm9108	22.17	81.93	-1.88
Gm6300	84.85	312.16	-1.88
Fn3k	233.35	855.58	-1.87
9330159M07Rik	26.03	95.41	-1.87
Sectm1b	236.24	861.81	-1.87
Gm47795	11.57	42.52	-1.87
Kcnj10	986.43	3593.44	-1.86
Pah	5358.37	19503.10	-1.86
Slc26a7	27.00	98.52	-1.86
Gm10681	27.00	98.52	-1.86
Unc5c	108.96	396.16	-1.86
Iqcn	22.17	80.89	-1.86
Pantr1	68.46	248.90	-1.86
Aqp4	280.60	1017.37	-1.86
Vegfd	80.99	293.49	-1.86
-	79.07	286.23	-1.85
Cyp2j7	12.53	45.63	-1.85
Mreg	28.92	104.75	-1.85
Gm13855	86.78	313.20	-1.85
L3mbtl1	4.82	17.63	-1.84
Gm42670	4.82	17.63	-1.84
Igkv3-10	4.82	17.63	-1.84
Gm30784	13.50	48.75	-1.84
Gm28417	27.96	100.60	-1.84
Zmynd10	205.38	736.32	-1.84
Efhd1	874.58	3124.69	-1.84
Tmem117	113.78	406.53	-1.84
Prox1	107.99	384.76	-1.83
Kcnk4	21.21	75.71	-1.83
Slco4c1	234.31	832.77	-1.83
Arhgef39	110.89	394.09	-1.83
Mfsd4a	760.80	2700.53	-1.83
Flvcr2	203.46	719.73	-1.82
Atp2b2	46.28	163.86	-1.82
Cyp2d37-ps	12.53	44.60	-1.82
Tox	12.53	44.60	-1.82
Gm29430	5.78	20.74	-1.82
Gm32899	5.78	20.74	-1.82
Angptl1	5.78	20.74	-1.82
Gm15526	5.78	20.74	-1.82
Grem2	214.06	756.03	-1.82
Ak4	3573.53	12567.19	-1.81
Inpp5j	580.48	2039.92	-1.81
Gm44386	151.39	532.02	-1.81
Tdrd12	17.35	61.19	-1.81
Gm39078	39.53	138.97	-1.81
Gm43158	21.21	74.67	-1.81

Gm15823	29.89	104.75	-1.80
5330417C22Rik	10.60	37.34	-1.80
Spink8	156.21	545.50	-1.80
Usp2	1186.03	4126.50	-1.80
Klk1b5	27.96	97.49	-1.80
Gm26641	11.57	40.45	-1.79
Igfn1	11.57	40.45	-1.79
Ces1e	431.98	1497.53	-1.79
Gm18890	7.71	26.97	-1.79
Rhox6	7.71	26.97	-1.79
Lamc3	99.32	343.27	-1.79
Ifit1bl2	12.53	43.56	-1.79
Slc12a3	18008.46	61687.91	-1.78
Nap1l5	35.67	122.38	-1.77
Tmem25	706.80	2416.37	-1.77
Cpeb3	709.69	2421.56	-1.77
Clcnka	1151.32	3927.38	-1.77
Hsd3b4	357.74	1219.59	-1.77
Slc28a1	206.35	702.10	-1.77
Defb42	16.39	56.00	-1.76
Gm11827	16.39	56.00	-1.76
Aadat	1470.49	4994.52	-1.76
Car3	169.71	576.61	-1.76
Slco1a4	41.46	141.04	-1.76
Bco2	109.92	373.35	-1.76
Rbfox1	18.32	62.23	-1.76
Slc29a4	4.82	16.60	-1.76
Fndc1	4.82	16.60	-1.76
Mamdc2	4.82	16.60	-1.76
Fam71d	4.82	16.60	-1.76
Gm37652	4.82	16.60	-1.76
Gm42572	4.82	16.60	-1.76
Mroh7	156.21	527.87	-1.76
Csad	3089.48	10433.94	-1.76
Acaa2	4043.13	13606.33	-1.75
Slc22a30	1222.67	4105.75	-1.75
Hopx	37.60	126.53	-1.75
C6	5.78	19.71	-1.75
Klk1b3	5.78	19.71	-1.75
Gm36738	5.78	19.71	-1.75
Mst1	5.78	19.71	-1.75
Gpr37	5.78	19.71	-1.75
D630023F18Rik	265.17	889.81	-1.75
Sectm1a	30.85	103.71	-1.74
Gm15990	14.46	48.75	-1.74
Bdh1	1168.68	3910.79	-1.74
Slc37a4	2969.91	9920.59	-1.74
Aqp11	629.66	2102.14	-1.74
Trim43c	6.75	22.82	-1.74
Gm36723	6.75	22.82	-1.74
Mir1968	6.75	22.82	-1.74
Magix	110.89	370.24	-1.74
Cend1	16.39	54.97	-1.74
Halr1	96.42	321.49	-1.74
Nupr1l	18.32	61.19	-1.73
Gm40787	7.71	25.93	-1.73
Dscaml1	7.71	25.93	-1.73
Edar	7.71	25.93	-1.73
Lgr5	7.71	25.93	-1.73
BC049987	94.49	314.23	-1.73
Slc16a5	348.09	1155.30	-1.73
Gm33280	8.68	29.04	-1.73

Zkscan4	8.68	29.04	-1.73
Slc25a29	75.21	248.90	-1.72
Vipr1	9.64	32.15	-1.72
-	77.14	255.12	-1.72
Ciart	91.60	302.83	-1.72
Rflna	24.10	79.86	-1.72
Dpys	334.59	1102.41	-1.72
Akr1c18	107.99	355.72	-1.72
Fgf12	13.50	44.60	-1.71
Gm49291	13.50	44.60	-1.71
Apela	167.78	550.69	-1.71
Slc22a3	14.46	47.71	-1.71
Akap5	17.35	57.04	-1.71
Gm5069	18.32	60.15	-1.71
-	127.28	413.79	-1.70
Scnn1a	2007.58	6509.68	-1.70
Aspdh	640.26	2075.18	-1.70
Eci3	1338.39	4335.98	-1.70
Fbp1	9619.43	31034.26	-1.69
Slc2a12	112.82	364.01	-1.69
Spp2	2299.75	7412.96	-1.69
1500026H17Rik	57.85	186.68	-1.69
Notumos	27.96	90.23	-1.69
Ano5	22.17	71.56	-1.68
Cyp2u1	90.64	291.42	-1.68
Cpt1b	13.50	43.56	-1.68
Bbox1	13.50	43.56	-1.68
5430402O13Rik	12.53	40.45	-1.68
Frmpd4	11.57	37.34	-1.68
Frs3os	10.60	34.23	-1.68
Adra2c	10.60	34.23	-1.68
Shh	9.64	31.12	-1.68
-	8.68	28.00	-1.68
Slc22a19	558.30	1783.76	-1.68
9230102O04Rik	7.71	24.89	-1.67
Gm48931	7.71	24.89	-1.67
Hoxd4	7.71	24.89	-1.67
Pbld2	361.59	1153.22	-1.67
Gm14634	6.75	21.78	-1.67
Lingo4	6.75	21.78	-1.67
Ank2	151.39	482.24	-1.67
Ascl4	5.78	18.67	-1.67
4930524J08Rik	5.78	18.67	-1.67
Slc38a11	5.78	18.67	-1.67
Slc25a35	250.70	795.43	-1.67
B3galnt1	162.96	515.43	-1.66
Pecr	3586.07	11334.12	-1.66
Gm15318	154.28	487.42	-1.66
Ass1	11511.30	36344.05	-1.66
Gm11788	18.32	58.08	-1.66
Ak3l2-ps	89.67	283.12	-1.66
Gm15848	107.99	340.16	-1.65
Emx1	581.44	1829.39	-1.65
Vmn1r18	15.43	48.75	-1.65
Spock3	14.46	45.63	-1.65
8430422M14Rik	82.92	260.31	-1.65
Cyp2e1	13352.06	41763.76	-1.65
-	12.53	39.41	-1.64
4930426D05Rik	100.28	313.20	-1.64
-	456.09	1421.82	-1.64
Gm40663	11.57	36.30	-1.64
Slc5a8	2150.29	6695.31	-1.64

Ckb	1706.73	5306.68	-1.64
Mansc4	95.46	296.60	-1.63
Omd	10.60	33.19	-1.63
Negr1	10.60	33.19	-1.63
Tppp	41.46	128.60	-1.63
Tspan1	395.34	1223.74	-1.63
Sema4g	652.80	2020.21	-1.63
Slc2a13	79.07	244.75	-1.63
Scn7a	48.21	149.34	-1.63
BC049352	9.64	30.08	-1.63
Gm28437	2040.36	6307.45	-1.63
Shisa2	15.43	47.71	-1.62
Slc22a8	4531.04	13933.01	-1.62
Tnfaip8	2742.34	8432.40	-1.62
Gm36210	33.75	103.71	-1.62
Slc22a26	126.31	386.83	-1.61
Gm28551	7.71	23.86	-1.61
Slc34a1	51193.37	156411.61	-1.61
Ttr	551.55	1685.24	-1.61
Slc26a4	917.01	2799.05	-1.61
-	24.10	73.63	-1.61
Acsm5	963.29	2925.57	-1.60
C330013E15Rik	23.14	70.52	-1.60
Rab11fip4	1225.57	3721.00	-1.60
Tmem72	1461.81	4437.62	-1.60
Rxrg	6.75	20.74	-1.60
Nrxn3	6.75	20.74	-1.60
4930451E10Rik	6.75	20.74	-1.60
Gm44008	6.75	20.74	-1.60
Amdhd1	17.35	52.89	-1.60
Gm13528	27.96	85.04	-1.60
Slc5a11	632.55	1914.43	-1.60
BC064078	47.25	143.12	-1.60
Cxcl12	1220.75	3688.85	-1.60
Sucnr1	304.70	920.92	-1.60
Gm6135	26.03	78.82	-1.59
Gm4450	558.30	1684.20	-1.59
Npy1r	15.43	46.67	-1.59
Cmbl	1503.27	4521.62	-1.59
Prelp	176.46	530.98	-1.59
3110070M22Rik	10.60	32.15	-1.59
Angpt1	177.42	533.06	-1.59
Fcamr	185.13	555.87	-1.59
Col23a1	42.42	127.56	-1.59
Tmem221	63.64	190.82	-1.58
Gm15651	14.46	43.56	-1.58
Gm26808	14.46	43.56	-1.58
Tmie	170.67	511.28	-1.58
Clca3a1	441.63	1322.26	-1.58
Crispld1	23.14	69.49	-1.58
Plekhd1	202.49	605.65	-1.58
Dnase1	4273.58	12772.53	-1.58
Sostdc1	949.79	2837.42	-1.58
Mkln1os	27.00	80.89	-1.58
Smcp	9.64	29.04	-1.58
Aqp2	4019.98	11970.88	-1.57
Pacsin1	13.50	40.45	-1.57
Irs1	163.92	487.42	-1.57
Acy3	8991.69	26692.06	-1.57
Acot11	474.41	1408.34	-1.57
Gm11789	28.92	86.08	-1.57
Hmgcs2	3534.00	10480.61	-1.57

Hsd17b11	2825.27	8361.88	-1.57
Gm16332	12.53	37.34	-1.57
Klhl14	81.96	242.68	-1.56
Trmt9b	46.28	136.90	-1.56
Lypd6	56.89	168.01	-1.56
Gm11615	55.92	164.90	-1.56
Arl4d	172.60	508.17	-1.56
Gm27343	11.57	34.23	-1.55
Slc15a1	11.57	34.23	-1.55
Gm48558	131.14	384.76	-1.55
Elavl3	18.32	53.93	-1.55
Pik3c2g	74.24	217.79	-1.55
Gm45606	53.03	155.56	-1.55
Rap1gap	1510.02	4421.02	-1.55
Gm13844	7.71	22.82	-1.55
2610318M16Rik	7.71	22.82	-1.55
Gm14023	14.46	42.52	-1.55
Scube3	178.38	521.65	-1.55
Rapgefl1	27.96	81.93	-1.55
Lhx1	418.48	1222.71	-1.55
Gm19541	37.60	109.93	-1.54
Slit2	485.98	1414.56	-1.54
Dnajc6	344.24	1001.81	-1.54
Pdk2	2862.88	8322.47	-1.54
Gm6614	130.17	378.53	-1.54
Pfkm	829.26	2407.04	-1.54
Slc15a2	1061.64	3081.13	-1.54
Gm6652	45.32	131.71	-1.54
Cyp4a14	21661.07	62799.65	-1.54
Fabp3	1336.46	3868.27	-1.53
Syt7	102.21	295.57	-1.53
Sult1a1	212.13	612.91	-1.53
Nol3	137.89	398.24	-1.53
Pmel	9.64	28.00	-1.53
Gm37296	9.64	28.00	-1.53
Igkv8-30	12.53	36.30	-1.52
Eaf2	101.24	291.42	-1.52
Mapk10	15.43	44.60	-1.52
Gm42397	85.82	246.83	-1.52
Spag8	27.00	77.78	-1.52
Cldn8	744.40	2137.40	-1.52
Zim1	38.57	110.97	-1.52
Mcrip2	1592.95	4572.44	-1.52
Fam13a	930.51	2670.45	-1.52
Cyfip2	3256.29	9342.95	-1.52
Bhmt2	1816.66	5209.19	-1.52
Lrrn1	75.21	215.71	-1.52
Kl	7925.23	22659.94	-1.52
Gm6665	20.25	58.08	-1.51
Catsperz	17.35	49.78	-1.51
Kank4	64.60	184.60	-1.51
Fgf9	206.35	589.06	-1.51
Ybx2	305.67	872.18	-1.51
Csmd1	14.46	41.49	-1.51
1810010D01Rik	11.57	33.19	-1.51
Slc13a4	68.46	194.97	-1.51
Clcnkb	3025.84	8604.56	-1.51
Serpina1d	1715.41	4876.30	-1.51
Hpgd	143.67	408.61	-1.51
Fgfbp1	73.28	208.45	-1.51
Ptger3	835.04	2369.70	-1.50
Gm10766	16.39	46.67	-1.50

Kcnab3	24.10	68.45	-1.50
Mcoln3	224.67	634.69	-1.50
Htr1b	59.78	169.05	-1.50
Fbxo40	356.77	1007.00	-1.50
Rab27a	358.70	1011.14	-1.49
Rrh	38.57	108.90	-1.49
Gm5424	2269.86	6394.56	-1.49
Vwce	262.27	738.40	-1.49
Gm38309	10.60	30.08	-1.49
Tox3	107.99	303.86	-1.49
Rgs11	148.49	416.90	-1.49
Mettl7a3	25.07	70.52	-1.49
Gm18159	25.07	70.52	-1.49
Slc7a8	3595.71	10080.30	-1.49
Cbr2	29.89	84.01	-1.49
Sptbn2	876.51	2455.78	-1.49
-	44.35	124.45	-1.49
Hnf4aos	99.32	277.94	-1.48
Zfp750	60.75	170.08	-1.48
Panct2	54.96	153.49	-1.48
Acy1	1270.89	3543.66	-1.48
Pde4c	1498.45	4167.98	-1.48
Gm15883	134.99	375.42	-1.47
Gm24119	21.21	59.12	-1.47
Slc43a2	3456.86	9592.88	-1.47
1700019D03Rik	9.64	26.97	-1.47
Dcxr	1989.26	5509.94	-1.47
-	242.03	669.95	-1.47
Plppr4	63.64	176.30	-1.47
Gm42937	107.99	298.68	-1.47
-	62.67	173.19	-1.46
Tbc1d22bos	11.57	32.15	-1.46
Gm37320	11.57	32.15	-1.46
-	11.57	32.15	-1.46
Gm19265	27.00	74.67	-1.46
Enpp1	627.73	1730.87	-1.46
0610006L08Rik	174.53	481.20	-1.46
Smim5	211.17	581.80	-1.46
2310016G11Rik	13.50	37.34	-1.46
Gldc	3595.71	9874.96	-1.46
Mettl7a2	851.44	2337.55	-1.46
Gm47889	24.10	66.38	-1.46
Gm39033	15.43	42.52	-1.46
Impa2	250.70	686.54	-1.45
Acsl1	5235.91	14316.72	-1.45
Syp	63.64	174.23	-1.45
Dhtkd1	681.73	1860.50	-1.45
Gm45645	48.21	131.71	-1.45
D430019H16Rik	35.67	97.49	-1.45
Slc25a21	211.17	575.58	-1.45
Lpl	7381.38	20108.75	-1.45
Gm45203	10.60	29.04	-1.44
Hykk	3034.51	8244.69	-1.44
Gm1840	12.53	34.23	-1.44
-	12.53	34.23	-1.44
Tff1	16.39	44.60	-1.44
BC049715	16.39	44.60	-1.44
-	44.35	120.30	-1.44
Ido2	107.99	292.46	-1.44
Irx1	681.73	1844.95	-1.44
Slc22a14	31.82	86.08	-1.43
Tmem151a	111.85	301.79	-1.43

Slc13a2	744.40	2005.69	-1.43
D230017M19Rik	257.45	693.80	-1.43
Ripor3	185.13	498.83	-1.43
Ndrg2	1532.20	4124.42	-1.43
Acss1	3659.35	9840.74	-1.43
Cckar	1269.92	3409.88	-1.42
Adrb1	27.00	72.60	-1.42
-	27.00	72.60	-1.42
Bcl2l14	23.14	62.23	-1.42
Hsbp1l1	21.21	57.04	-1.42
Rps18-ps3	13.50	36.30	-1.42
Gm26684	45.32	121.34	-1.42
Trnp1	11.57	31.12	-1.42
3110083C13Rik	11.57	31.12	-1.42
Amacr	2136.79	5704.91	-1.42
Pck1	39734.14	106053.67	-1.42
Gpr162	139.81	373.35	-1.42
Dusp9	106.07	283.12	-1.42
Gm16010	578.55	1542.12	-1.41
Tmem26	189.96	505.06	-1.41
Gm3336	51.10	135.86	-1.41
AC114585.1	20.25	53.93	-1.41
Fam78a	63.64	169.05	-1.41
Pdzd7	43.39	115.12	-1.41
Tle2	338.45	896.03	-1.40
Slc22a13	955.58	2529.41	-1.40
Gm43031	39.53	104.75	-1.40
Colgalt2	228.53	603.58	-1.40
Gm20743	44.35	117.19	-1.40
Bckdhb	610.37	1609.53	-1.40
Slc25a42	2395.21	6312.64	-1.40
Ckmt1	767.55	2020.21	-1.40
Bmx	12.53	33.19	-1.40
Gp2	55.92	147.27	-1.39
Slc7a12	466.70	1225.82	-1.39
Lnx1	91.60	240.60	-1.39
Lipg	58.82	154.53	-1.39
Pla2g3	39.53	103.71	-1.39
Ano3	146.56	383.72	-1.39
Sgk2	2510.92	6562.57	-1.39
Pbld1	2488.74	6500.34	-1.39
Osr1	28.92	75.71	-1.38
1700055D18Rik	22.17	58.08	-1.38
Upb1	2084.72	5437.35	-1.38
Cyp1a1	240.10	626.39	-1.38
Ugt3a1	1951.65	5086.82	-1.38
Lin7a	33.75	88.15	-1.38
Pygm	113.78	296.60	-1.38
Gm12962	20.25	52.89	-1.38
Gpr137b	2307.46	6000.48	-1.38
Hlf	1322.96	3438.92	-1.38
Scube1	13.50	35.26	-1.38
Fbln5	1433.85	3719.97	-1.38
Hmx2	108.96	282.09	-1.37
Cela1	885.18	2288.81	-1.37
Gm25047	11.57	30.08	-1.37
Gm34777	16.39	42.52	-1.37
Tenm2	30.85	79.86	-1.37
Itih5	477.30	1232.04	-1.37
Wscd1	91.60	236.45	-1.37
Tmem64	2253.47	5803.43	-1.36
Fut9	4296.73	11060.33	-1.36

Gm43429	24.10	62.23	-1.36
Cabyr	24.10	62.23	-1.36
Dnajc12	1097.32	2822.90	-1.36
Gm15952	14.46	37.34	-1.36
Slc10a5	554.44	1422.86	-1.36
Efcab12	47.25	121.34	-1.36
Gm128	34.71	89.19	-1.36
Srp54a	100.28	257.20	-1.36
Dixdc1	195.74	500.91	-1.36
D630039A03Rik	350.99	896.03	-1.35
Klf15	1636.34	4176.28	-1.35
Hrg	20.25	51.86	-1.35
Sowaha	41.46	105.78	-1.35
Prodh	4810.68	12248.81	-1.35
A230060F14Rik	15.43	39.41	-1.35
Rps26-ps1	28.92	73.63	-1.34
Adamtsl2	453.20	1150.11	-1.34
Gm43190	75.21	190.82	-1.34
Tldc2	48.21	122.38	-1.34
Gm14257	43.39	109.93	-1.34
0610005C13Rik	2033.62	5142.82	-1.34
Gm44832	27.00	68.45	-1.34
1810034E14Rik	139.81	353.64	-1.34
Gm45792	497.55	1257.97	-1.34
Frem2	271.92	687.58	-1.34
Gm8798	32.78	82.97	-1.34
Fitm1	538.05	1357.52	-1.33
Slc22a7	971.97	2450.59	-1.33
Hao2	3040.30	7654.60	-1.33
Nos1	33.75	85.04	-1.33
Fam222a	108.96	273.79	-1.33
Slc39a8	803.22	2016.06	-1.33
Ptpn18	215.03	539.28	-1.33
Gstk1	784.90	1967.32	-1.33
Srcin1	727.05	1821.09	-1.32
-	158.14	396.16	-1.32
Gamt	199.60	499.87	-1.32
Khk	7251.21	18149.73	-1.32
Prlr	591.09	1478.86	-1.32
Slc35f1	64.60	161.79	-1.32
E530011L22Rik	20.25	50.82	-1.32
5033417F24Rik	20.25	50.82	-1.32
Gm15890	17.35	43.56	-1.32
Sord	17710.51	44244.43	-1.32
Tesc	277.70	693.80	-1.32
Trim67	14.46	36.30	-1.32
Qpct	11.57	29.04	-1.32
Atp5l2-ps	11.57	29.04	-1.32
Gm16365	11.57	29.04	-1.32
AC163032.1	32.78	81.93	-1.32
Insl6	36.64	91.27	-1.31
Slc6a19	4616.86	11467.90	-1.31
Proz	47.25	117.19	-1.31
1810010H24Rik	25.07	62.23	-1.31
Slco1a1	1623.81	4016.57	-1.31
4932422M17Rik	137.89	341.20	-1.31
Gnmt	474.41	1172.93	-1.31
Hddc3	31.82	78.82	-1.31
Scd2	2921.70	7211.77	-1.30
Cit	120.53	297.64	-1.30
Smim24	985.47	2431.93	-1.30
Wnk4	3643.92	8989.31	-1.30

Ovol1	118.60	292.46	-1.30
-	55.92	137.93	-1.30
Hnf1aos1	112.82	277.94	-1.30
Gm13075	112.82	277.94	-1.30
Hoxd8	1841.73	4534.06	-1.30
Adcy5	103.17	254.08	-1.30
Cyp4a10	17669.04	43443.81	-1.30
Pdk1	596.87	1466.42	-1.30
Gstt3	566.02	1389.67	-1.30
Nrtn	197.67	485.35	-1.30
Tef	4490.54	10992.92	-1.29
Ceacam2	395.34	967.59	-1.29
Tbxa2r	80.03	196.01	-1.29
Kcnn1	62.67	153.49	-1.29
Adhfe1	2990.16	7308.22	-1.29
Cyp2j13	1298.85	3172.39	-1.29
Nxpe3	250.70	611.87	-1.29
Gm15879	50.14	122.38	-1.29
Thnsl2	2991.12	7271.92	-1.28
Adam1a	55.92	135.86	-1.28
Gm33472	14.46	35.26	-1.28
Zfp286	14.46	35.26	-1.28
AW822252	139.81	339.12	-1.28
2610203C22Rik	26.03	63.26	-1.28
AI838599	201.53	488.46	-1.28
-	33.75	81.93	-1.28
Ccrl2	53.03	128.60	-1.28
Kcnq1	498.52	1207.15	-1.28
Hnmt	180.31	436.61	-1.28
Bcat2	1546.67	3740.71	-1.27
3110045C21Rik	88.71	214.68	-1.27
Ak1	458.02	1107.59	-1.27
Aifm3	298.92	722.84	-1.27
Gnaz	81.96	198.08	-1.27
Gm42941	27.00	65.34	-1.27
Etfb	1298.85	3132.99	-1.27
Macrod2	1234.25	2976.39	-1.27
Gm5436	47.25	114.08	-1.27
Bcar3	427.16	1029.81	-1.27
Aldh4a1	3985.27	9599.10	-1.27
Nsg2	27.96	67.41	-1.27
Ppp2r2b	60.75	146.23	-1.27
N4bp2os	74.24	178.38	-1.26
Acadm	15466.68	37108.36	-1.26
Pygl	321.09	770.54	-1.26
Gm13652	95.46	229.20	-1.26
Nr3c2	267.10	640.91	-1.26
Fa2h	184.17	441.79	-1.26
Slc6a20b	2892.77	6933.84	-1.26
Aqp1	14565.10	34885.93	-1.26
Fam217a	58.82	141.04	-1.26
Tsc22d3	702.94	1683.16	-1.26
Sctr	16.39	39.41	-1.26
AC110166.2	25.07	60.15	-1.26
AC107792.3	33.75	80.89	-1.26
A230107N01Rik	26.03	62.23	-1.25
2810414N06Rik	26.03	62.23	-1.25
Nrap	26.03	62.23	-1.25
Bmp7	899.65	2142.59	-1.25
Gm6710	17.35	41.49	-1.25
Tln2	3210.01	7629.71	-1.25
Gm42067	78.10	185.64	-1.25

Idh2	6988.93	16559.90	-1.24
2810425M01Rik	13.50	32.15	-1.24
Ptprn2	13.50	32.15	-1.24
Gm3716	185.13	438.68	-1.24
Rnaset2a	23.14	54.97	-1.24
Acox2	1934.30	4579.69	-1.24
Gm3219	27.96	66.38	-1.24
Ptger1	49.17	116.15	-1.24
D630044L22Rik	39.53	93.34	-1.24
Gna14	161.99	381.64	-1.24
Mapk15	380.88	897.07	-1.24
Gk	8515.35	20047.56	-1.24
Cavin2	465.73	1096.18	-1.23
Ppargc1a	1308.49	3076.98	-1.23
Shmt2	4105.80	9649.92	-1.23
Mdh1	17182.09	40373.05	-1.23
Gm11695	88.71	208.45	-1.23
Gm671	20.25	47.71	-1.23
1700018L02Rik	20.25	47.71	-1.23
Spag6l	20.25	47.71	-1.23
Sgpp2	508.16	1192.63	-1.23
Ptpdc1	590.12	1383.45	-1.23
Dhrs11	487.91	1142.85	-1.23
Atp2a3	234.31	548.61	-1.23
Retsat	7159.61	16757.98	-1.23
Chrm3	37.60	88.15	-1.23
Fmo2	4423.04	10344.75	-1.23
Gm44117	21.21	49.78	-1.23
Papss2	4261.05	9956.89	-1.22
Cyp51	2726.92	6371.75	-1.22
Gm13594	32.78	76.75	-1.22
Gm35549	32.78	76.75	-1.22
Large2	522.62	1219.59	-1.22
Vegfb	952.68	2221.40	-1.22
Gm37691	16.39	38.37	-1.22
Pxmp2	414.63	965.51	-1.22
Tmem88b	69.42	161.79	-1.22
6430571L13Rik	63.64	148.30	-1.22
Ankrd9	175.49	408.61	-1.22
C230035I16Rik	28.92	67.41	-1.22
Bmp8a	17.35	40.45	-1.21
Ctsf	986.43	2288.81	-1.21
Smad9	29.89	69.49	-1.21
Esrra	3282.33	7605.86	-1.21
Gm19705	24.10	56.00	-1.21
Hyal3	24.10	56.00	-1.21
-	339.42	786.10	-1.21
Trpm6	397.27	918.85	-1.21
Nxph4	37.60	87.12	-1.21
D930015M05Rik	18.32	42.52	-1.21
Ces1f	6212.71	14362.36	-1.21
Hoxd9	813.83	1881.24	-1.21
Tlr12	634.48	1466.42	-1.21
Daam2	375.09	866.99	-1.21
Fxyd1	58.82	135.86	-1.21
Tmem213	538.05	1241.37	-1.21
Map2k6	206.35	476.02	-1.21
Slco2b1	509.12	1173.96	-1.21
Gm15638	215.03	495.72	-1.20
Cux2	53.03	122.38	-1.20
Gm15860	53.03	122.38	-1.20
Gm31992	19.28	44.60	-1.20

Rap1gap2	1031.75	2374.89	-1.20
3110099E03Rik	33.75	77.78	-1.20
Qdpr	3448.18	7926.31	-1.20
Gm19950	3227.37	7412.96	-1.20
Gm4524	170.67	392.01	-1.20
-	49.17	113.04	-1.20
Kynu	423.31	971.74	-1.20
Alb	79.07	181.49	-1.20
2810030D12Rik	64.60	148.30	-1.20
Aldob	80046.83	183198.04	-1.19
Rilp	351.95	804.77	-1.19
Stard8	1680.70	3841.30	-1.19
Acox3	3657.42	8351.51	-1.19
Atp6v0d2	453.20	1033.96	-1.19
Tmem206	419.45	955.14	-1.19
Rac3	133.06	302.83	-1.19
Fat2	24.10	54.97	-1.18
Adamts15	331.70	753.95	-1.18
Hmcn2	169.71	384.76	-1.18
Gper1	26.03	59.12	-1.18
Gm43667	91.60	207.42	-1.18
Map3k7cl	615.19	1391.75	-1.18
Gm2415	74.24	168.01	-1.18
Hrasls	18.32	41.49	-1.17
Pcbd1	2849.38	6420.49	-1.17
Sfxn2	1074.18	2420.52	-1.17
Abcc6	303.74	684.47	-1.17
-	100.28	226.08	-1.17
Lyplal1	817.69	1841.84	-1.17
Tril	121.49	273.79	-1.17
Cd300lg	575.66	1296.34	-1.17
Adra1a	426.20	959.29	-1.17
Glul	10338.76	23251.06	-1.17
Pdzrn3	577.59	1297.37	-1.17
Galnt14	1064.54	2389.41	-1.17
Spc25	499.48	1121.07	-1.17
Isoc2a	3845.45	8627.37	-1.17
4930481A15Rik	122.46	274.83	-1.17
Kcnj11	22.17	49.78	-1.16
Cabcoco1	146.56	327.72	-1.16
Gal3st1	435.84	973.81	-1.16
Ggt5	99.32	221.94	-1.16
Rab3a	164.88	368.16	-1.16
Cbs	2121.36	4729.03	-1.16
Ecm2	38.57	86.08	-1.16
Gm6277	66.53	148.30	-1.15
Lpar3	527.45	1173.96	-1.15
Mtfp1	541.91	1206.11	-1.15
Igfbp1	125.35	278.97	-1.15
Hoxb3os	82.92	184.60	-1.15
Mogat2	155.24	345.35	-1.15
Gm20518	100.28	222.97	-1.15
Hsd3b3	837.94	1860.50	-1.15
Slc29a1	582.41	1292.19	-1.15
Hist1h4i	90.64	201.19	-1.15
C1qtnf7	46.28	102.67	-1.15
P4htm	33.75	74.67	-1.14
Cdo1	2131.01	4704.14	-1.14
Gstm7	145.60	321.49	-1.14
Gpr137b-ps	1377.92	3038.61	-1.14
Gfra1	77.14	170.08	-1.14
Scel	97.39	214.68	-1.14

Gm14794	37.60	82.97	-1.14
Gm44949	60.75	133.78	-1.14
Pla2g7	188.99	415.87	-1.14
Cox6b2	368.34	809.95	-1.14
Cyp4a32	62.67	137.93	-1.14
Ascc1	1020.18	2242.14	-1.14
Acpp	66.53	146.23	-1.13
Apc2	43.39	95.41	-1.13
Scpep1os	21.21	46.67	-1.13
D030055H07Rik	25.07	54.97	-1.13
Haao	2589.99	5660.32	-1.13
Atp5g1	4654.47	10167.42	-1.13
Gm11992	528.41	1154.26	-1.13
Heph	185.13	404.46	-1.13
Notum	251.67	549.65	-1.13
Cnnm4	386.66	844.18	-1.13
-	62.67	136.90	-1.13
Car4	1554.38	3391.21	-1.13
Gm17586	133.06	290.38	-1.13
Prelid2	65.57	143.12	-1.12
Hoxd3	36.64	79.86	-1.12
Ccl28	331.70	720.77	-1.12
AC154691.1	242.03	525.80	-1.12
Cldn10	2376.89	5161.49	-1.12
Isoc2b	266.13	577.65	-1.12
Raver2	48.21	104.75	-1.12
Tlcd1	611.34	1326.41	-1.12
Lhx1os	279.63	606.69	-1.12
Csrp2	2070.26	4489.47	-1.12
Rdh16	3442.39	7463.78	-1.12
Aadac	2048.08	4436.58	-1.12
Lpin1	1106.96	2397.70	-1.11
Arhgef9	62.67	135.86	-1.11
Gm16124	64.60	140.01	-1.11
Aqp6	977.75	2112.51	-1.11
Extl1	102.21	220.90	-1.11
Tmed6	227.56	491.57	-1.11
Aldh9a1	10232.69	22081.25	-1.11
4833439L19Rik	10674.32	23027.06	-1.11
Susd2	2517.67	5422.83	-1.11
Tjp3	329.77	710.39	-1.11
Gm12121	343.27	738.40	-1.10
Il15	212.13	456.31	-1.10
Chrdl1	77.14	165.93	-1.10
Insc	62.67	134.82	-1.10
Inca1	57.85	124.45	-1.10
Clec11a	52.07	112.01	-1.10
9130230N09Rik	46.28	99.56	-1.10
Reln	40.50	87.12	-1.10
D730001G18Rik	27.96	60.15	-1.10
Rab40b	27.96	60.15	-1.10
Gm26631	27.00	58.08	-1.10
Cdh4	24.10	51.86	-1.10
Osbpl1a	1430.95	3068.69	-1.10
Adcy1	475.38	1019.44	-1.10
Atp2b4	851.44	1825.24	-1.10
Fabp7	142.71	305.94	-1.10
Gstm2	593.02	1270.41	-1.10
Ninl	556.37	1191.59	-1.10
Gm5113	221.78	474.98	-1.10
Gm26767	101.24	216.75	-1.10
Phf11c	98.35	210.53	-1.10

Wnk1	7539.52	16125.37	-1.10
A330023F24Rik	178.38	381.64	-1.10
Pdp2	1234.25	2631.04	-1.09
Ppp1r16b	1471.45	3135.06	-1.09
Susd4	106.07	226.08	-1.09
Mgll	1638.27	3488.70	-1.09
Gm37254	54.00	115.12	-1.09
Dock8	1210.14	2575.04	-1.09
Abat	4987.13	10611.28	-1.09
C730036E19Rik	50.14	106.82	-1.09
Ptgds	137.89	293.49	-1.09
Cyp27a1	1067.43	2270.14	-1.09
Fam81a	340.38	723.88	-1.09
Fam120aos	501.41	1065.07	-1.09
Arhgap24	3437.57	7300.96	-1.09
Neu2	119.56	254.08	-1.09
Oxct1	16110.80	34214.95	-1.09
Gas1	430.06	911.59	-1.08
Osbp2	353.88	749.80	-1.08
Tspan13	226.60	480.17	-1.08
Gm45819	35.67	75.71	-1.08
Gm17750	84.85	179.42	-1.08
Sypl2	1457.95	3079.06	-1.08
Hist2h2be	107.03	226.08	-1.08
Nacad	54.00	114.08	-1.08
Ankrd24	645.08	1360.64	-1.08
Agmat	27.96	59.12	-1.08
-	52.07	109.93	-1.08
Gm43775	27.00	57.04	-1.08
Prdm16	448.38	944.77	-1.08
Snhg11	2619.88	5519.28	-1.07
Lancl3	49.17	103.71	-1.07
Upp2	139.81	294.53	-1.07
Cd164l2	48.21	101.64	-1.07
Esr1	198.63	417.94	-1.07
Rhov	172.60	362.98	-1.07
Bcl11b	166.81	350.53	-1.07
Acaa1b	12088.89	25372.91	-1.07
Hspb7	99.32	208.45	-1.07
Kbtbd11	637.37	1336.78	-1.07
Syt9	39.53	82.97	-1.07
Slc2a9	456.09	955.14	-1.07
Slc25a11	3083.69	6456.79	-1.07
1700113A16Rik	38.57	80.89	-1.07
Zc2hc1c	163.92	343.27	-1.07
Tmem218	135.96	284.16	-1.06
Myo5c	245.88	513.35	-1.06
Cytip	245.88	513.35	-1.06
-	179.35	374.38	-1.06
Gm38102	97.39	203.27	-1.06
Lactb2	9864.35	20574.39	-1.06
Kcnc2	49.17	102.67	-1.06
Sirt3	1177.35	2454.74	-1.06
Fam214a	810.94	1689.39	-1.06
Gm12251	122.46	255.12	-1.06
Akr1c19	627.73	1306.71	-1.06
Kifc2	297.95	620.17	-1.06
Prps2	1198.57	2488.97	-1.05
Tmem178	664.37	1379.30	-1.05
Mecom	840.83	1744.35	-1.05
Abhd18	325.92	676.17	-1.05
Rtn4rl1	229.49	476.02	-1.05

Dmtn	4068.20	8430.33	-1.05
Dlec1	1263.17	2617.56	-1.05
Nit2	1419.38	2941.13	-1.05
Zfr2	80.03	165.93	-1.05
Mgat3	2272.75	4703.11	-1.05
Sh3d21	139.81	289.35	-1.05
Acss2	3149.26	6508.64	-1.05
Itga2b	124.39	257.20	-1.05
G0s2	808.04	1668.65	-1.05
Tet2	1075.14	2218.29	-1.04
Slc5a2	6655.30	13729.74	-1.04
Gm17753	48.21	99.56	-1.04
Gcdh	7337.03	15111.12	-1.04
Igfbp5	9735.14	20004.00	-1.04
Cyp4a31	10548.97	21670.57	-1.04
Lgals4	397.27	816.18	-1.04
Slc16a9	2187.90	4491.54	-1.04
Acot1	3793.38	7784.23	-1.04
Shank2	949.79	1945.54	-1.03
Dpf3	51.10	104.75	-1.03
Cyp2c23	518.77	1061.96	-1.03
Smim4	813.83	1665.53	-1.03
Slc30a2	1160.00	2373.85	-1.03
Klf1	49.17	100.60	-1.03
Peg3	644.12	1315.01	-1.03
Abhd14b	2187.90	4465.62	-1.03
Stk32b	273.85	558.98	-1.03
Gm11423	94.49	192.90	-1.03
Antxr1	103.17	210.53	-1.03
Chst7	434.88	886.70	-1.03
Aspg	758.87	1546.27	-1.03
Fmo1	10772.68	21940.21	-1.03
Slc38a3	3625.60	7382.89	-1.03
Slc35g1	447.41	910.55	-1.02
Scnn1g	993.18	2020.21	-1.02
C920021L13Rik	45.32	92.30	-1.02
Spata2l	174.53	354.68	-1.02
Ppil6	86.78	176.30	-1.02
-	85.82	174.23	-1.02
Gm42375	43.39	88.15	-1.02
Acat1	11426.44	23177.43	-1.02
Rnf208	167.78	340.16	-1.02
Gm11611	200.56	406.53	-1.02
Hes6	1055.86	2139.47	-1.02
Fras1	744.40	1507.90	-1.02
Sod2	5549.30	11237.67	-1.02
Pde1a	907.36	1835.61	-1.02
A430035B10Rik	49.17	99.56	-1.02
Gpt	855.29	1728.80	-1.02
Fbxo44	453.20	915.73	-1.01
Acsm2	46910.14	94772.45	-1.01
Cmc2	281.56	568.32	-1.01
S100g	8370.71	16891.76	-1.01
Col27a1	2042.29	4121.31	-1.01
Sp5	301.81	608.76	-1.01
Hgd	6664.94	13440.40	-1.01
Ndufb10	4509.83	9090.94	-1.01
B3gat2	882.29	1778.57	-1.01
Dpp4	1408.78	2838.46	-1.01
Rdh16f2	2907.23	5854.25	-1.01
Gm44250	195.74	394.09	-1.01
Afmid	322.06	648.17	-1.01

Gstz1	4346.87	8735.23	-1.01
Slc12a6	997.04	2003.62	-1.01
Pantr2	66.53	133.78	-1.01
Apobec2	58.82	118.23	-1.01
Plcb1	512.02	1027.74	-1.01
L3hypdh	525.52	1054.70	-1.00
Gcnt1	8639.74	17332.52	-1.00
Tm2d2	1549.56	3107.06	-1.00
Slc6a13	3277.51	6569.83	-1.00
Ddt	578.55	1159.44	-1.00
Tmem204	369.31	739.43	-1.00
Rdh11	1177.35	588.02	1.00
Ptpn22	145.60	72.60	1.00
Shtn1	1279.57	637.80	1.00
Hivep2	1107.93	551.72	1.01
Sdc4	20181.90	10038.82	1.01
Parp9	1225.57	608.76	1.01
Sh3bgrl3	2000.83	993.51	1.01
Themis2	96.42	47.71	1.01
Smc2	303.74	150.38	1.01
Spry2	691.37	342.24	1.01
Apaf1	435.84	215.71	1.01
Map1b	435.84	215.71	1.01
Gm14681	178.38	88.15	1.02
Mkl2	1559.20	770.54	1.02
Nfam1	117.64	58.08	1.02
Isg15	260.35	128.60	1.02
Rnh1	8039.01	3969.90	1.02
Wasf1	128.24	63.26	1.02
St3gal1	1340.31	661.65	1.02
Susd1	756.94	373.35	1.02
Adgrg1	23250.16	11468.94	1.02
Cd93	1893.80	932.33	1.02
Casp4	78.10	38.37	1.02
Fbxl2	303.74	149.34	1.02
Ampd3	2111.72	1037.07	1.03
Ankrd6	409.81	201.19	1.03
Mchr1	209.24	102.67	1.03
Fam43a	1637.31	803.73	1.03
Il1r1	636.41	312.16	1.03
Dtx4	1883.19	922.99	1.03
Ctsb	50636.99	24819.11	1.03
Gm3756	233.35	114.08	1.03
Pafah1b3	772.37	377.50	1.03
Srgap1	422.34	206.38	1.03
Gfpt2	104.14	50.82	1.03
Bst2	1125.29	549.65	1.03
Gmpr	7091.14	3463.81	1.03
Fcer1g	319.17	155.56	1.04
D830044D21Rik	70.39	34.23	1.04
Acta2	1832.09	891.88	1.04
Lmna	5816.40	2830.16	1.04
Pou2f2	89.67	43.56	1.04
Psd	432.95	210.53	1.04
5430416N02Rik	119.56	58.08	1.04
Tmem98	539.98	262.38	1.04
Sema6a	1131.07	549.65	1.04
Oasl2	609.41	295.57	1.04
Trim30d	126.31	61.19	1.04
Ppp1r14b	3428.90	1661.39	1.05
Ly6a	21964.81	10638.25	1.05
Gmds	978.72	473.94	1.05

Gpr39	66.53	32.15	1.05
Aif1	105.10	50.82	1.05
Syk	143.67	69.49	1.05
Tpm3	10383.12	5025.63	1.05
Lcp1	1868.73	904.33	1.05
Arid5b	2220.68	1074.41	1.05
Mrpl33	1861.98	899.14	1.05
Ammecr1	348.09	168.01	1.05
Serpinb9	1810.87	874.25	1.05
Zc3hav1	1495.56	721.80	1.05
Vmp1	9659.92	4660.59	1.05
Xdh	3904.27	1883.32	1.05
Mex3d	456.09	219.86	1.05
Cd151	8403.50	4051.83	1.05
Mast4	1661.41	799.58	1.05
Fgf2	301.81	145.19	1.06
Mxra7	659.55	317.35	1.06
Gm13375	135.96	65.34	1.06
Sult2b1	62.67	30.08	1.06
H2-T10	62.67	30.08	1.06
Rflnb	2066.40	993.51	1.06
Tap1	897.72	431.42	1.06
BC021767	161.99	77.78	1.06
Abca1	3341.15	1605.38	1.06
Phgdh	1209.18	579.72	1.06
Vopp1	674.98	323.57	1.06
Colec12	718.37	344.31	1.06
Heyl	270.95	129.64	1.06
Eif1a	3937.06	1883.32	1.06
S100a9	269.02	128.60	1.06
Gm9791	162.96	77.78	1.07
Timeless	412.70	197.05	1.07
Pi15	110.89	52.89	1.07
Cad	1690.34	806.84	1.07
Arhgef40	1431.92	683.43	1.07
Crem	1076.11	513.35	1.07
Xbp1	11936.54	5692.47	1.07
3110082I17Rik	356.77	170.08	1.07
Nras	2492.60	1188.48	1.07
Tmbim1	2156.08	1027.74	1.07
Mapk11	328.81	156.60	1.07
Ppp1r18	701.98	333.94	1.07
Ccne2	124.39	59.12	1.07
Fut10	65.57	31.12	1.07
Nefm	65.57	31.12	1.07
Lsm5	157.17	74.67	1.07
Rgs2	837.94	398.24	1.07
Ccdc88b	172.60	81.93	1.07
Tcaf1	1296.92	616.02	1.07
Apex1	585.30	277.94	1.07
Col4a2	16787.71	7962.61	1.08
Irak3	137.89	65.34	1.08
Kif11	63.64	30.08	1.08
Fat1	6696.76	3170.32	1.08
Gm16685	184.17	87.12	1.08
B3gnt3	377.02	178.38	1.08
Iffo2	464.77	219.86	1.08
Pcolce2	103.17	48.75	1.08
Snora31	79.07	37.34	1.08
Btg1	3089.48	1461.23	1.08
Rps27a	7202.03	3404.70	1.08
Smad3	2851.31	1347.15	1.08

Plk2	487.91	230.23	1.08
Smyd5	1217.85	574.54	1.08
Pla2g5	312.42	147.27	1.08
Steap4	77.14	36.30	1.09
Stat3	10935.64	5153.19	1.09
Nlgn2	564.09	265.49	1.09
Cxadr	2522.49	1187.45	1.09
Zfp9	99.32	46.67	1.09
Dhx58	143.67	67.41	1.09
Nipsnap3b	1382.74	649.21	1.09
Irf7	713.55	334.98	1.09
Rock2	3801.10	1782.72	1.09
Phldb3	323.02	151.42	1.09
Cdc7	119.56	56.00	1.09
Pirb	126.31	59.12	1.09
Hoxb2	126.31	59.12	1.09
Vcl	3033.55	1420.79	1.09
Itgb4	507.20	237.49	1.09
Midn	5695.86	2665.27	1.10
Tubb2a	4733.53	2213.11	1.10
Ppp4r4	448.38	209.49	1.10
Arhgap11a	270.95	126.53	1.10
Zyx	4625.54	2160.22	1.10
Il3ra	115.71	53.93	1.10
Plau	26950.98	12558.89	1.10
1810055G02Rik	416.56	193.93	1.10
Stk32a	430.06	200.16	1.10
Tjp2	3534.96	1644.79	1.10
Scimp	55.92	25.93	1.11
Rrad	600.73	278.97	1.11
Mb21d1	62.67	29.04	1.11
A230050P20Rik	596.87	276.90	1.11
Map4k4	3138.65	1455.01	1.11
Aebp1	5257.13	2437.11	1.11
Phldb1	3691.17	1710.13	1.11
Creb3l1	512.98	237.49	1.11
Zc3h12a	358.70	165.93	1.11
Ihh	2401.96	1110.70	1.11
Gm10575	54.00	24.89	1.11
Prrx1	128.24	59.12	1.12
Mob3a	175.49	80.89	1.12
Nr4a1	377.99	174.23	1.12
Ubash3b	587.23	270.68	1.12
Ifit3b	295.06	135.86	1.12
Ptpre	193.81	89.19	1.12
Itga3	4072.05	1875.02	1.12
Hr	85.82	39.41	1.12
Gata6	65.57	30.08	1.12
Nnmt	65.57	30.08	1.12
Cenpe	58.82	26.97	1.12
Rcc2	2715.34	1247.60	1.12
Tnfsfm13	97.39	44.60	1.12
Il4ra	1565.95	716.62	1.13
Ier5l	1246.78	570.39	1.13
Sirpa	3435.64	1571.16	1.13
Ptgfrn	883.26	403.42	1.13
Anxa9	145.60	66.38	1.13
Zfp948	329.77	150.38	1.13
Jpt1	1459.88	665.80	1.13
Ybx3	4026.73	1834.58	1.13
Dyrk3	150.42	68.45	1.13
C4b	1899.58	863.88	1.14

Pknox2	173.56	78.82	1.14
Rela	2775.13	1261.08	1.14
Lrp1	4594.68	2086.58	1.14
Ets2	3745.17	1697.68	1.14
Ncf1	146.56	66.38	1.14
Pros1	1963.22	888.77	1.14
Mapk7	753.08	340.16	1.15
Rel	215.99	97.49	1.15
Bub1b	71.35	32.15	1.15
Csrp1	3853.17	1738.13	1.15
Col16a1	1267.03	571.43	1.15
Kcne4	82.92	37.34	1.15
Galnt15	322.06	145.19	1.15
Ifit2	916.04	412.76	1.15
Rab7b	135.96	61.19	1.15
Fstl1	1875.48	844.18	1.15
Slit3	576.62	259.27	1.15
Hic1	380.88	171.12	1.15
Socs1	57.85	25.93	1.15
Ifit3	1034.64	464.61	1.15
Anxa5	7219.39	3239.80	1.16
Tmem184b	2360.50	1057.81	1.16
Tlnrd1	1513.88	678.25	1.16
Dock2	166.81	74.67	1.16
Gm5637	74.24	33.19	1.16
Gm10277	62.67	28.00	1.16
Gm13415	51.10	22.82	1.16
Cryab	7674.52	3435.81	1.16
Pax8	3902.35	1746.43	1.16
Serpinh1	7715.02	3451.36	1.16
Plscr1	3164.69	1415.60	1.16
Inava	1387.56	620.17	1.16
Jam2	598.80	267.57	1.16
Lrrc8e	67.49	30.08	1.16
Axl	2358.57	1052.63	1.16
Il33	912.18	406.53	1.17
Esyt1	1491.70	664.76	1.17
C3ar1	111.85	49.78	1.17
Trpv2	212.13	94.38	1.17
Gm44785	289.27	128.60	1.17
Serpina3g	77.14	34.23	1.17
Fn1	1716.37	762.25	1.17
Gins1	170.67	75.71	1.17
Smim3	490.80	217.79	1.17
D630003M21Rik	81.96	36.30	1.17
Oas1a	175.49	77.78	1.17
Foxm1	98.35	43.56	1.17
Pold1	863.01	382.68	1.17
Spi1	241.06	106.82	1.17
Gm12250	54.00	23.86	1.17
Gpx7	70.39	31.12	1.17
Il2rg	103.17	45.63	1.17
Eprn	953.65	422.09	1.18
Coro1a	696.19	308.01	1.18
Plpp2	3903.31	1726.72	1.18
Elf3	1341.28	593.21	1.18
Fen1	75.21	33.19	1.18
Fxyd5	685.58	302.83	1.18
Rpp25	42.42	18.67	1.18
Olfr1372-ps1	63.64	28.00	1.18
Kntc1	84.85	37.34	1.18
Cd53	164.88	72.60	1.18

Nop58	1916.94	844.18	1.18
Ddx60	68.46	30.08	1.18
Gm43759	68.46	30.08	1.18
Tax1bp3	2193.68	965.51	1.18
Gja1	349.06	153.49	1.18
Syne2	17714.36	7792.53	1.18
Kif21b	162.96	71.56	1.19
Pus7l	167.78	73.63	1.19
Lcp2	78.10	34.23	1.19
Snhg5	219.85	96.45	1.19
Serpinb6a	1705.77	748.77	1.19
Trim45	104.14	45.63	1.19
Dok1	224.67	98.52	1.19
Ifi204	217.92	95.41	1.19
Cyp24a1	2876.38	1260.04	1.19
Shisa4	963.29	421.05	1.19
Tlr9	40.50	17.63	1.19
Gm29787	40.50	17.63	1.19
Gar1	1092.50	477.05	1.20
Gclc	22667.75	9898.82	1.20
Ifrd1	1579.45	689.65	1.20
Aox1	76.17	33.19	1.20
Klhl23	80.99	35.26	1.20
Cdt1	478.27	208.45	1.20
Nt5c1a	90.64	39.41	1.20
AA414768	59.78	25.93	1.20
Spire2	401.13	174.23	1.20
Ttll7	210.21	91.27	1.20
Ercc1	651.83	283.12	1.20
Bmp6	5796.15	2518.00	1.20
Slc11a1	169.71	73.63	1.20
Was	74.24	32.15	1.20
Irf1	1192.78	517.50	1.20
Itpkc	428.13	185.64	1.21
Gm31774	158.14	68.45	1.21
S100a8	232.38	100.60	1.21
Atad5	162.96	70.52	1.21
Aplnr	167.78	72.60	1.21
Plch1	242.03	104.75	1.21
Dap	4045.06	1749.54	1.21
Gpc3	1259.32	544.46	1.21
Pask	156.21	67.41	1.21
Rasgrp4	53.03	22.82	1.21
Coro1c	6359.27	2743.05	1.21
Steap2	10243.30	4417.91	1.21
Ifit1	442.59	190.82	1.21
Selenon	442.59	190.82	1.21
Mybl1	77.14	33.19	1.21
Emp1	1946.83	838.99	1.21
Carhsp1	3554.25	1531.75	1.21
Kcnk6	125.35	53.93	1.22
Gm10156	134.99	58.08	1.22
Slc1a4	3247.61	1397.97	1.22
Parp14	1397.21	600.47	1.22
Zbp1	142.71	61.19	1.22
Rbpms	5482.76	2353.11	1.22
Runx2	113.78	48.75	1.22
Tpm3-rs7	983.54	421.05	1.22
Slc16a3	160.06	68.45	1.22
Il21r	60.75	25.93	1.22
Rps12-ps9	51.10	21.78	1.23
Pemt	126.31	53.93	1.23

Mmp9	121.49	51.86	1.23
Pdzd4	121.49	51.86	1.23
Lxn	191.88	81.93	1.23
Ddias	41.46	17.63	1.23
Ccno	41.46	17.63	1.23
Ablim3	647.98	275.86	1.23
Arntl2	124.39	52.89	1.23
Iigp1	641.23	272.75	1.23
Cpne7	239.13	101.64	1.23
Slc7a1	1400.10	595.28	1.23
Pprc1	1850.41	786.10	1.23
A730020M07Rik	100.28	42.52	1.24
Csf3r	227.56	96.45	1.24
Mamstr	80.99	34.23	1.24
Kcnn4	49.17	20.74	1.24
-	799.37	338.09	1.24
Sash1	4375.80	1849.10	1.24
H6pd	26503.57	11188.93	1.24
Gpr161	93.53	39.41	1.24
S1pr2	285.42	120.30	1.25
Mapk4	2970.87	1251.74	1.25
1700016C15Rik	1486.88	626.39	1.25
Kifc1	83.89	35.26	1.25
Rcan1	9810.35	4131.68	1.25
Gm13552	101.24	42.52	1.25
Gas7	441.63	185.64	1.25
Il20rb	74.24	31.12	1.25
Rcn1	896.76	376.46	1.25
Pgd	13465.85	5653.06	1.25
Slfn1	52.07	21.78	1.25
Cdc20	69.42	29.04	1.25
Ciita	429.09	179.42	1.26
Map1a	163.92	68.45	1.26
Atad2	404.98	169.05	1.26
Mt1	15662.42	6539.75	1.26
Relt	42.42	17.63	1.26
Ctsd	28478.37	11867.17	1.26
Col4a1	24867.22	10359.27	1.26
Slc12a4	2085.69	868.03	1.26
Wnt9a	97.39	40.45	1.27
Gm45515	37.60	15.56	1.27
Parvg	117.64	48.75	1.27
Zswim4	863.97	357.79	1.27
Cstb	5715.15	2365.55	1.27
Phlda3	426.20	176.30	1.27
Net1	8835.48	3655.67	1.27
Zbtb42	421.38	174.23	1.27
Map3k8	145.60	60.15	1.27
Bard1	57.85	23.86	1.27
Msl3l2	45.32	18.67	1.27
Elf4	402.09	165.93	1.28
Naip2	118.60	48.75	1.28
Klf8	40.50	16.60	1.28
Mex3a	237.20	97.49	1.28
Pim1	638.34	262.38	1.28
Pdlim3	134.03	54.97	1.28
Tert	80.99	33.19	1.28
Trp53	2420.28	993.51	1.28
Eda	88.71	36.30	1.29
Loxl4	283.49	116.15	1.29
Fgf18	149.46	61.19	1.29
Plec	9835.42	4026.94	1.29

Cp	6019.85	2462.00	1.29
Vav1	152.35	62.23	1.29
Tnfaip3	512.98	209.49	1.29
Elf5	119.56	48.75	1.29
Gm9333	43.39	17.63	1.29
Pklr	3603.43	1469.53	1.29
Adamtsl5	79.07	32.15	1.30
Slc39a6	544.80	221.94	1.30
Vasp	3238.94	1319.15	1.30
Pmaip1	1142.64	464.61	1.30
Rps27rt	3040.30	1236.19	1.30
Nuak2	13210.32	5367.87	1.30
Ccr2	266.13	107.86	1.30
Tacc2	11178.63	4533.03	1.30
Itgax	215.03	87.12	1.30
Sec14l2	238.17	96.45	1.30
Ccl4	38.57	15.56	1.30
Rasl12	782.01	316.31	1.31
Dysf	2712.45	1097.22	1.31
Chac1	444.52	179.42	1.31
Mthfd2	926.65	373.35	1.31
Rad18	141.74	57.04	1.31
Pole2	103.17	41.49	1.31
Tlr1	80.03	32.15	1.31
Gm10123	3960.20	1592.94	1.31
Kctd11	539.02	216.75	1.31
Thbs1	4434.62	1783.76	1.31
Uprt	263.24	105.78	1.31
Eno2	152.35	61.19	1.31
Layn	152.35	61.19	1.31
-	41.46	16.60	1.31
Nfkb1	2444.39	982.11	1.32
4921507P07Rik	33.75	13.48	1.32
Gm17383	33.75	13.48	1.32
Mospd2	1873.55	751.88	1.32
Usp43	443.56	177.34	1.32
Col1a2	4161.73	1664.50	1.32
Bcl6b	352.92	141.04	1.32
Insyn1	78.10	31.12	1.32
Soat2	62.67	24.89	1.33
Cenpt	482.13	191.86	1.33
Gstm5	10891.28	4333.91	1.33
Ptpn23	5673.69	2256.66	1.33
Gm31651	99.32	39.41	1.33
Emp2	937.25	372.31	1.33
Ifitm3	2812.73	1116.93	1.33
Cmtm3	569.87	226.08	1.33
Gm9910	65.57	25.93	1.33
Ckap4	1125.29	445.94	1.34
Col8a1	908.33	359.87	1.34
Dok4	1164.82	461.50	1.34
Plk3	1339.35	529.94	1.34
Rgs19	262.27	103.71	1.34
Exo1	39.53	15.56	1.34
Atp8b4	68.46	26.97	1.34
Cd2ap	9758.28	3852.71	1.34
Gm19221	126.31	49.78	1.34
Cdh24	147.53	58.08	1.34
Ikzf4	189.96	74.67	1.35
AI839979	31.82	12.45	1.35
Eno4	31.82	12.45	1.35
Scamp4	31.82	12.45	1.35

Bco1	153.31	60.15	1.35
Smox	768.51	301.79	1.35
Pik3ap1	103.17	40.45	1.35
Ifngr2	1685.52	661.65	1.35
Sh2d4b	113.78	44.60	1.35
Mab21l3	216.95	85.04	1.35
Skap1	74.24	29.04	1.35
Pnrc1	2643.03	1036.03	1.35
Adgrf1	95.46	37.34	1.35
Epb42	55.92	21.78	1.36
Lmnb1	496.59	193.93	1.36
Pde11a	34.71	13.48	1.36
Frzb	1190.85	464.61	1.36
Fjx1	526.48	205.34	1.36
Il1rl2	101.24	39.41	1.36
Mcm4	795.51	310.09	1.36
Adamtsl4	489.84	190.82	1.36
Chtf18	80.03	31.12	1.36
Mcm5	826.37	321.49	1.36
Angptl7	149.46	58.08	1.36
Socs2	1496.52	581.80	1.36
Slc38a2	7680.30	2982.61	1.36
1700007J10Rik	37.60	14.52	1.37
Gm15337	37.60	14.52	1.37
Gstp3	262.27	101.64	1.37
Cldn1	1591.99	617.06	1.37
Slc15a3	313.38	121.34	1.37
Rad54l	75.21	29.04	1.37
Gls2	27.00	10.37	1.37
Ncaph	88.71	34.23	1.37
Slc4a11	674.01	260.31	1.37
Fam167a	54.00	20.74	1.38
Eif6	9267.47	3572.70	1.38
Mcam	1568.84	603.58	1.38
Trim46	267.10	102.67	1.38
Psd3	836.01	321.49	1.38
Cd84	56.89	21.78	1.38
Espl1	70.39	26.97	1.38
Tagln2	12541.12	4804.74	1.38
Itgb2	379.91	145.19	1.39
Gm43460	32.78	12.45	1.39
Sdc1	1698.05	648.17	1.39
Mical2	663.41	253.05	1.39
Gpihbp1	509.12	193.93	1.39
Gm8116	204.42	77.78	1.39
Lyz2	890.97	339.12	1.39
Apbb1ip	98.35	37.34	1.39
Galnt6	35.67	13.48	1.40
Cdk6	2655.56	1008.03	1.40
Tnfrsf1a	6225.24	2362.44	1.40
Hgf	169.71	64.30	1.40
Tmem43	1979.62	750.84	1.40
Plek	257.45	97.49	1.40
-	1664.30	630.54	1.40
Uchl1	241.06	91.27	1.40
Gm47283	566.98	214.68	1.40
Mmp14	980.65	371.27	1.40
Peg10	38.57	14.52	1.40
Tnfrsf22	214.06	80.89	1.40
Map3k14	1187.00	448.02	1.41
Zfp850	96.42	36.30	1.41
Tspan4	4045.06	1525.53	1.41

Tlr13	115.71	43.56	1.41
F2rl3	41.46	15.56	1.41
Tubb4a	41.46	15.56	1.41
Pfkfb3	1840.76	692.76	1.41
Usp27x	223.70	84.01	1.41
Stx1b	44.35	16.60	1.41
-	44.35	16.60	1.41
Ggt7	105.10	39.41	1.41
Pgm1	1263.17	473.94	1.41
Ecscr	404.02	151.42	1.42
Cenph	47.25	17.63	1.42
Gpr153	357.74	133.78	1.42
Gpr176	94.49	35.26	1.42
Gm16184	50.14	18.67	1.42
H3f3c	27.96	10.37	1.42
Fblim1	1329.71	496.76	1.42
Fam111a	239.13	89.19	1.42
Lox	222.74	82.97	1.42
Rhoc	6333.24	2359.33	1.42
Vat1	5893.54	2195.48	1.42
Cdr2l	409.81	152.45	1.43
Pdk4	3153.12	1170.85	1.43
Mcm3	715.48	265.49	1.43
Calhm5	33.75	12.45	1.43
Flna	13824.55	5128.30	1.43
Gm44126	117.64	43.56	1.43
Sema6b	501.41	185.64	1.43
Efemp2	735.73	271.71	1.44
Nbl1	522.62	192.90	1.44
Cenps	73.28	26.97	1.44
Gm6477	79.07	29.04	1.44
Gm8186	235.28	86.08	1.45
Trpv5	1880.30	687.58	1.45
Ywhah	7399.71	2703.64	1.45
Gm9951	423.31	154.53	1.45
Tubb5	6879.01	2511.78	1.45
Tgfb1	1023.07	373.35	1.45
Fbn1	1384.67	505.06	1.45
Cxcr4	142.71	51.86	1.46
Prr5l	339.42	123.41	1.46
Fhl3	362.56	131.71	1.46
Ccr5	188.99	68.45	1.46
Aldh18a1	538.05	194.97	1.46
Fyb2	280.60	101.64	1.46
Chek1	126.31	45.63	1.47
Ighv14-2	26.03	9.34	1.47
Catip	26.03	9.34	1.47
Mapk6	3081.76	1113.81	1.47
Pik3r1	7464.31	2697.42	1.47
Mir155hg	28.92	10.37	1.47
Aff2	28.92	10.37	1.47
Scn5a	34.71	12.45	1.47
Gad1os	37.60	13.48	1.47
Sobp	40.50	14.52	1.47
Inka2	52.07	18.67	1.47
Ttc16	54.96	19.71	1.47
Aldh1a3	57.85	20.74	1.47
Ptpn7	63.64	22.82	1.47
Gm13597	377.99	135.86	1.48
Prc1	83.89	30.08	1.48
Sparc	11146.81	4004.12	1.48
Sptlc2	13612.41	4888.74	1.48

Golm1	866.86	311.12	1.48
Map3k1	4701.71	1685.24	1.48
Hck	168.74	60.15	1.49
Procr	151.39	53.93	1.49
Bhlhe40	8201.97	2922.46	1.49
Nfil3	582.41	207.42	1.49
Cdh3	824.44	293.49	1.49
Kif7	79.07	28.00	1.49
Cpne8	374.13	132.75	1.49
Cd300ld	73.28	25.93	1.49
Rap2b	231.42	81.93	1.50
Cd180	61.71	21.78	1.50
Hcn3	219.85	77.78	1.50
Lpcat2	55.92	19.71	1.50
Rhou	478.27	169.05	1.50
Klc3	123.42	43.56	1.50
Rras2	3610.18	1275.60	1.50
6330562C20Rik	50.14	17.63	1.50
Fbxo2	114.74	40.45	1.50
S100a14	47.25	16.60	1.50
Gpm6b	609.41	214.68	1.50
Ccnf	35.67	12.45	1.51
Sytl2	2121.36	743.58	1.51
Parp3	2979.55	1042.26	1.52
Fos	249.74	87.12	1.52
Gm12854	345.20	120.30	1.52
Akap6	27.00	9.34	1.52
Gm15601	59.78	20.74	1.52
Map3k6	742.47	258.23	1.52
Gm12981	24.10	8.30	1.52
Gm8234	24.10	8.30	1.52
Gm8893	24.10	8.30	1.52
Spsb1	519.73	180.45	1.53
Slc7a5	448.38	155.56	1.53
Slc28a2	21.21	7.26	1.53
Pold4	1564.99	541.35	1.53
Hmga1	1564.02	540.32	1.53
Asf1b	72.32	24.89	1.53
-	1394.31	481.20	1.53
Tgm7	45.32	15.56	1.54
Gbp7	1585.24	546.54	1.54
Ncapg2	241.06	82.97	1.54
Igf2bp2	1067.43	367.13	1.54
Foxs1	84.85	29.04	1.54
Stra6l	3759.64	1290.12	1.54
Nsl1	39.53	13.48	1.54
Sumo2	922.79	316.31	1.54
Adam19	299.88	102.67	1.55
Dctd	124.39	42.52	1.55
H2-DMb2	79.07	26.97	1.55
B4galnt1	1079.96	369.20	1.55
Clec4a1	36.64	12.45	1.55
Cdca8	76.17	25.93	1.55
Cenpu	54.96	18.67	1.55
Cftr	927.61	316.31	1.55
Clic1	7117.18	2426.74	1.55
Slc10a6	73.28	24.89	1.55
Maoa	1158.07	393.05	1.56
Lgals1	5236.88	1776.50	1.56
Ercc6l	67.49	22.82	1.56
Gadd45a	2966.05	1003.88	1.56
Csrnp1	922.79	312.16	1.56

Mall	98.35	33.19	1.56
Rasd1	331.70	112.01	1.57
Gsta1	479.23	161.79	1.57
Gbp3	836.01	282.09	1.57
Tuba1a	1839.80	620.17	1.57
Bmp1	985.47	331.86	1.57
2610028D06Rik	27.96	9.34	1.57
Lig1	693.30	233.34	1.57
Chaf1a	299.88	100.60	1.57
Tgtp1	242.03	80.89	1.58
Il7r	25.07	8.30	1.58
Adgrg3	722.23	240.60	1.59
Trim47	3595.71	1194.71	1.59
Plekho2	437.77	145.19	1.59
Rhob	7304.24	2421.56	1.59
Ckap2l	22.17	7.26	1.59
AC166110.3	34.71	11.41	1.59
Gm7265	47.25	15.56	1.60
Ulbp1	84.85	28.00	1.60
Rnd3	1075.14	355.72	1.60
BC055324	107.03	35.26	1.60
Arpc1b	4913.85	1619.90	1.60
Car13	365.45	120.30	1.60
Tes	1900.55	625.36	1.60
Gm38158	31.82	10.37	1.61
Lrrc32	1789.66	585.95	1.61
Cercam	41.46	13.48	1.61
Bbc3	250.70	81.93	1.61
Sox11	19.28	6.23	1.61
Cd6	19.28	6.23	1.61
2700029L08Rik	19.28	6.23	1.61
AL805899.1	19.28	6.23	1.61
Hivep3	260.35	85.04	1.61
Ajuba	5089.35	1659.31	1.62
Rab44	28.92	9.34	1.62
Trp53i11	459.95	149.34	1.62
Tnfaip8l1	38.57	12.45	1.62
Gm7665	383.77	124.45	1.62
S100a10	11962.57	3874.49	1.63
Runx3	96.42	31.12	1.63
Asns	974.86	315.27	1.63
Cldn3	782.97	253.05	1.63
Gm8990	54.96	17.63	1.63
Pdlim1	319.17	102.67	1.64
Asprv1	26.03	8.30	1.64
Tnfsf9	26.03	8.30	1.64
Pilrb2	26.03	8.30	1.64
Gm11963	26.03	8.30	1.64
Zfp57	167.78	53.93	1.64
Tlr2	270.95	87.12	1.64
-	184.17	59.12	1.64
Itga2	71.35	22.82	1.64
Tcf7	71.35	22.82	1.64
Sema4c	814.79	261.34	1.64
Gpr27	52.07	16.60	1.64
Arl4c	1128.18	360.90	1.64
Ptgs2	78.10	24.89	1.64
AC154232.2	23.14	7.26	1.66
Myh9	36584.87	11604.79	1.66
Kcnd1	55.92	17.63	1.66
Gm19144	55.92	17.63	1.66
Iglon5	55.92	17.63	1.66

Gbp2	717.40	227.12	1.66
Msln	88.71	28.00	1.66
Birc5	62.67	19.71	1.66
Rtn4	12217.13	3855.82	1.66
Gm15410	29.89	9.34	1.67
Slc13a1	42655.84	13433.14	1.67
Fam19a3	53.03	16.60	1.67
Eif4ebp1	2405.82	754.99	1.67
Apol9b	66.53	20.74	1.68
Adamts2	431.02	134.82	1.68
9830144P21Rik	308.56	96.45	1.68
Gm42928	43.39	13.48	1.68
Fscn1	757.90	236.45	1.68
Gm16091	86.78	26.97	1.68
Alox5	20.25	6.23	1.68
Foxa1	20.25	6.23	1.68
Ret	20.25	6.23	1.68
Gm15361	20.25	6.23	1.68
Slfn4	130.17	40.45	1.68
Stc1	584.34	181.49	1.69
Psd4	227.56	70.52	1.69
Tpbg	83.89	25.93	1.69
Card14	475.38	147.27	1.69
Gm26764	33.75	10.37	1.69
Tpm4	7536.63	2334.44	1.69
Sntg2	244.92	75.71	1.69
F2rl1	2397.14	741.51	1.69
Mthfd1l	325.92	100.60	1.69
Mirt1	67.49	20.74	1.70
Wisp1	350.02	107.86	1.70
Ppl	832.15	256.16	1.70
Phlda1	357.74	109.93	1.70
Nptxr	165.85	50.82	1.70
Nfkbie	351.95	107.86	1.71
Ppp1r13l	600.73	183.56	1.71
Izumo1	30.85	9.34	1.71
Casq1	30.85	9.34	1.71
Tubb2b	2437.64	743.58	1.71
Gm19951	24.10	7.26	1.71
Cks1b	242.03	73.63	1.72
Micall2	310.49	94.38	1.72
Efhd2	3595.71	1093.07	1.72
Ier5	1672.02	508.17	1.72
Rgs1	17.35	5.19	1.72
Gm11427	17.35	5.19	1.72
A430105J06Rik	17.35	5.19	1.72
Piwil2	17.35	5.19	1.72
Tnnt1	17.35	5.19	1.72
Pilra	41.46	12.45	1.73
Tmeff1	41.46	12.45	1.73
Mlkl	329.77	99.56	1.73
Mboat1	432.95	130.67	1.73
-	65.57	19.71	1.73
Nek6	2890.84	872.18	1.73
Rnf125	76.17	22.82	1.73
Mis18bp1	27.96	8.30	1.74
2310043M15Rik	27.96	8.30	1.74
Olfr56	27.96	8.30	1.74
Gm22107	27.96	8.30	1.74
Tmsb10	2200.43	657.50	1.74
Crlf2	545.77	162.82	1.74
-	90.64	26.97	1.74

Adgra1	55.92	16.60	1.75
Gm4898	66.53	19.71	1.75
2700099C18Rik	21.21	6.23	1.75
Gm1673	21.21	6.23	1.75
Pabpc1l	21.21	6.23	1.75
Cxcl9	21.21	6.23	1.75
Flot1	5573.40	1656.20	1.75
Olfml2b	49.17	14.52	1.75
Mcub	59.78	17.63	1.75
Cd24a	13596.02	4029.01	1.75
Pdgfb	3673.82	1080.63	1.77
Tnfrsf1b	519.73	152.45	1.77
Ms4a6b	109.92	32.15	1.77
Clec5a	67.49	19.71	1.77
AF357399	25.07	7.26	1.77
Gm44667	25.07	7.26	1.77
Oasl1	262.27	76.75	1.77
Pole	251.67	73.63	1.77
Hpse	14.46	4.15	1.77
Nr5a2	14.46	4.15	1.77
Dnmt3bos	14.46	4.15	1.77
Tmprss6	14.46	4.15	1.77
Krt87	14.46	4.15	1.77
Gm12479	14.46	4.15	1.77
Hdx	14.46	4.15	1.77
Kif14	14.46	4.15	1.77
Cpe	8948.30	2619.64	1.77
Lilrb4a	166.81	48.75	1.77
Efcab5	85.82	24.89	1.78
Chaf1b	228.53	66.38	1.78
Fcgr1	135.96	39.41	1.78
Fkbp10	485.98	141.04	1.78
Ccdc120	1177.35	341.20	1.79
Ptger4	348.09	100.60	1.79
Sbno2	5814.47	1680.05	1.79
Col6a3	2514.78	725.95	1.79
Cep85	17662.29	5094.08	1.79
Gm47806	18.32	5.19	1.80
Aunip	18.32	5.19	1.80
Gm26781	18.32	5.19	1.80
Cep170	754.05	216.75	1.80
Llcfc1	32.78	9.34	1.80
Sorcs1	47.25	13.48	1.80
Cd300lf	61.71	17.63	1.80
S100a11	10408.19	2983.65	1.80
Basp1	224.67	64.30	1.80
AI504432	65.57	18.67	1.81
Icam1	2568.78	734.25	1.81
E2f7	36.64	10.37	1.81
Knstrn	69.42	19.71	1.81
Sh2b2	120.53	34.23	1.81
Bcl2a1b	22.17	6.23	1.81
Gm26637	22.17	6.23	1.81
1810064F22Rik	22.17	6.23	1.81
Hells	306.63	87.12	1.81
Nav3	73.28	20.74	1.81
Trem2	40.50	11.41	1.82
Fosb	40.50	11.41	1.82
Ms4a6c	58.82	16.60	1.82
Rell1	1618.02	457.35	1.82
Rmi2	44.35	12.45	1.82
Samsn1	44.35	12.45	1.82

Gm49064	26.03	7.26	1.82
Loxl2	742.47	209.49	1.82
Cd276	48.21	13.48	1.83
Ncf4	74.24	20.74	1.83
Krt15	74.24	20.74	1.83
Psca	52.07	14.52	1.83
Siglec1	78.10	21.78	1.84
Isg20	222.74	62.23	1.84
Camk2n2	319.17	89.19	1.84
Tedc1	81.96	22.82	1.84
Masp1	260.35	72.60	1.84
Orc1	37.60	10.37	1.85
Pgbd5	37.60	10.37	1.85
Brca1	153.31	42.52	1.85
B4galt2	250.70	69.49	1.85
Krt8	15262.26	4218.80	1.86
Hspb8	495.63	136.90	1.86
Trim6	116.67	32.15	1.86
Cdca5	49.17	13.48	1.86
5-Sep	15.43	4.15	1.86
Nxpe5	15.43	4.15	1.86
Mrap	15.43	4.15	1.86
Snord123	15.43	4.15	1.86
Il12rb1	15.43	4.15	1.86
Gm43654	15.43	4.15	1.86
Ccr7	15.43	4.15	1.86
Sh3bp2	1886.08	516.46	1.87
Dtl	174.53	47.71	1.87
Msn	21440.25	5868.77	1.87
Il1f5	19.28	5.19	1.87
B230377A18Rik	19.28	5.19	1.87
Ccdc65	23.14	6.23	1.87
Ier2	932.43	254.08	1.88
Tgfbi	2086.65	568.32	1.88
Igkv9-120	27.00	7.26	1.88
Psrc1	30.85	8.30	1.88
Ncapg	30.85	8.30	1.88
AC238676.1	30.85	8.30	1.88
Slc6a14	30.85	8.30	1.88
Arsi	34.71	9.34	1.88
Slc20a1	5887.75	1597.09	1.88
Ckap2	38.57	10.37	1.88
Gm25835	38.57	10.37	1.88
Tmem154	42.42	11.41	1.88
Cenpm	46.28	12.45	1.88
F13a1	73.28	19.71	1.89
Hmox1	7203.00	1944.51	1.89
Pvr	1730.84	466.68	1.89
Il1b	119.56	32.15	1.89
Tnfrsf10b	300.85	80.89	1.89
Osmr	2043.26	549.65	1.89
Tinagl1	9175.87	2465.11	1.90
Rad51	143.67	38.37	1.90
Mt2	78.10	20.74	1.91
Slc2a6	54.96	14.52	1.91
Plekho1	1395.28	370.24	1.91
Gm6169	117.64	31.12	1.91
Rdh12	43.39	11.41	1.92
Lhfpl2	803.22	212.60	1.92
Rhbdl2	164.88	43.56	1.92
Cfi	654.73	173.19	1.92
Pclaf	157.17	41.49	1.92

Rras	2035.54	536.17	1.92
Tuft1	3718.17	979.00	1.93
Mtfr2	27.96	7.26	1.93
Rad51b	27.96	7.26	1.93
Rhbdf2	1594.88	418.98	1.93
Adora2b	391.49	102.67	1.93
Gins2	269.02	70.52	1.93
Sacs	174.53	45.63	1.93
Gm37805	24.10	6.23	1.93
Pgf	59.78	15.56	1.93
4930539E08Rik	95.46	24.89	1.93
Ptafr	52.07	13.48	1.94
5033403H07Rik	20.25	5.19	1.94
Ugt1a9	20.25	5.19	1.94
Knl1	20.25	5.19	1.94
Htra3	48.21	12.45	1.94
Fgr	200.56	51.86	1.95
Gm12185	16.39	4.15	1.95
Fpr2	16.39	4.15	1.95
Trank1	16.39	4.15	1.95
Gm13062	16.39	4.15	1.95
Sptssb	16.39	4.15	1.95
Gm38387	16.39	4.15	1.95
5730420D15Rik	16.39	4.15	1.95
Hbegf	1805.09	465.65	1.95
Ttc39a	60.75	15.56	1.96
Anln	101.24	25.93	1.96
Emilin2	121.49	31.12	1.96
Trip13	56.89	14.52	1.96
Spns2	8985.91	2306.44	1.96
Cdk1	73.28	18.67	1.97
Iqgap3	32.78	8.30	1.97
Bpifc	12.53	3.11	1.97
Adgre4	12.53	3.11	1.97
Gm16617	12.53	3.11	1.97
Krt28	12.53	3.11	1.97
Gm16144	12.53	3.11	1.97
Gm17416	12.53	3.11	1.97
Gm13684	12.53	3.11	1.97
Ankrd55	12.53	3.11	1.97
4633401B06Rik	12.53	3.11	1.97
Gm44567	12.53	3.11	1.97
Snai1	212.13	53.93	1.97
Vim	3580.28	908.47	1.98
Gm8126	94.49	23.86	1.98
Fgd3	2614.10	661.65	1.98
F630028O10Rik	41.46	10.37	1.99
Il1rl1	25.07	6.23	1.99
Sprr2a3	25.07	6.23	1.99
2010003K11Rik	742.47	186.68	1.99
Hk3	66.53	16.60	2.00
Pea15a	7491.31	1872.95	2.00
Bcat1	2869.63	716.62	2.00
Sh2d5	62.67	15.56	2.00
Trib1	1951.65	486.39	2.00
Gm4070	204.42	50.82	2.01
Acat3	3510.86	874.25	2.01
Ripk3	125.35	31.12	2.01
9130208D14Rik	21.21	5.19	2.01
1700105P06Rik	21.21	5.19	2.01
Gm43820	21.21	5.19	2.01
Spink12	21.21	5.19	2.01

Gm48478	21.21	5.19	2.01
Hmga1b	175.49	43.56	2.01
Scara3	46.28	11.41	2.01
Cfap157	155.24	38.37	2.01
Tubb6	1570.77	388.90	2.01
Cebpb	2745.24	678.25	2.02
Gadd45b	302.77	74.67	2.02
Birc3	1136.86	280.01	2.02
Plp2	1716.37	421.05	2.03
Fndc4	444.52	108.90	2.03
Lrrc25	72.32	17.63	2.03
Prrg4	1111.79	271.71	2.03
Ifitm6	17.35	4.15	2.03
Gm16096	17.35	4.15	2.03
A430110C17Rik	17.35	4.15	2.03
Gdnf	17.35	4.15	2.03
Snord73a	17.35	4.15	2.03
Tmem45a	246.85	60.15	2.03
Nfkb2	2249.61	546.54	2.04
Stx11	124.39	30.08	2.04
Peg12	34.71	8.30	2.05
AC165953.1	34.71	8.30	2.05
Anxa8	34.71	8.30	2.05
Il18r1	43.39	10.37	2.05
Jun	4757.64	1145.96	2.05
Mvp	8381.32	2018.14	2.05
Sox4	1387.56	331.86	2.06
Fignl1	143.67	34.23	2.07
Rasl10a	30.85	7.26	2.07
Atp1a3	22.17	5.19	2.07
Batf	22.17	5.19	2.07
Tacstd2	7376.56	1754.72	2.07
Samd4	637.37	151.42	2.07
Gm15726	13.50	3.11	2.07
Entpd3	13.50	3.11	2.07
Gm12932	13.50	3.11	2.07
Cass4	13.50	3.11	2.07
A530064N14Rik	13.50	3.11	2.07
Gm45548	13.50	3.11	2.07
Aoc1	703.90	166.97	2.07
Capg	3206.15	760.17	2.08
Gli3	70.39	16.60	2.08
Rin1	500.45	118.23	2.08
Gm26115	83.89	19.71	2.08
Gm15895	480.20	113.04	2.09
Panx1	106.07	24.89	2.09
Timd2	7324.49	1720.50	2.09
Col5a1	1810.87	425.20	2.09
Cdkn1a	2113.65	494.68	2.09
Cfap44	40.50	9.34	2.10
Mir142hg	18.32	4.15	2.11
Gm17210	18.32	4.15	2.11
Pkmyt1	161.99	37.34	2.11
Top2a	202.49	46.67	2.11
Cidec	85.82	19.71	2.12
Tmem158	315.31	72.60	2.12
Tmem173	613.26	141.04	2.12
Jazf1	95.46	21.78	2.13
Itga5	1401.06	320.46	2.13
Kif1a	41.46	9.34	2.14
Myof	6333.24	1439.45	2.14
Snora20	74.24	16.60	2.15

Aspm	32.78	7.26	2.16
Ctps	5057.53	1134.56	2.16
Zfp36l1	7784.44	1742.28	2.16
Eid3	37.60	8.30	2.16
Fbln2	321.09	71.56	2.16
Adamts1	11506.48	2565.71	2.16
Litaf	5332.34	1187.45	2.17
Apln	942.08	209.49	2.17
Eda2r	70.39	15.56	2.17
-	14.46	3.11	2.17
Dnase1l3	14.46	3.11	2.17
Gm4813	14.46	3.11	2.17
Zfp82	14.46	3.11	2.17
Tmcc3os	14.46	3.11	2.17
Gm43123	14.46	3.11	2.17
Mki67	192.85	42.52	2.18
Rad51ap1	56.89	12.45	2.18
Lrguk	56.89	12.45	2.18
Adam11	1312.35	288.31	2.19
Syt12	1052.97	231.27	2.19
Gm12751	562.16	123.41	2.19
Tgif1	1686.48	370.24	2.19
-	71.35	15.56	2.19
Ahnak2	28.92	6.23	2.19
Pglyrp2	28.92	6.23	2.19
Lilr4b	223.70	48.75	2.20
Plin2	20114.40	4368.13	2.20
Vcam1	872.65	188.75	2.21
Ngf	168.74	36.30	2.21
Gm3776	192.85	41.49	2.21
Pxdc1	644.12	137.93	2.22
Spred3	494.66	105.78	2.22
Arg2	1574.63	336.01	2.23
Actn1	4990.03	1063.00	2.23
Col5a3	453.20	96.45	2.23
Steap1	1099.25	233.34	2.24
Chil3	112.82	23.86	2.24
Lgals3	7586.77	1606.42	2.24
Dnaaf3	59.78	12.45	2.25
Col15a1	2016.26	422.09	2.26
Gprc5a	377.02	78.82	2.26
Sphk1	1177.35	245.79	2.26
Snx10	944.00	197.05	2.26
Gtse1	80.03	16.60	2.26
F3	3118.40	649.21	2.26
Gm13398	15.43	3.11	2.26
Gm26740	15.43	3.11	2.26
Snord71	15.43	3.11	2.26
Lrr1	15.43	3.11	2.26
Tifa	1057.79	219.86	2.27
Cdc6	125.35	25.93	2.27
Diaph3	40.50	8.30	2.27
Nos2	35.67	7.26	2.28
Gvin1	212.13	43.56	2.28
Gm10752	10.60	2.08	2.29
Gm43442	10.60	2.08	2.29
Gm2238	10.60	2.08	2.29
Gprin1	10.60	2.08	2.29
Twist2	10.60	2.08	2.29
Adam12	593.02	121.34	2.29
Ptp4a1	86.78	17.63	2.29
Bin2	97.39	19.71	2.30

Ttc9	97.39	19.71	2.30
Ndc80	26.03	5.19	2.30
Tnf	26.03	5.19	2.30
Gm26628	26.03	5.19	2.30
Ctxn1	276.74	56.00	2.30
3110039I08Rik	431.02	87.12	2.31
BC023105	56.89	11.41	2.31
Ptp4a1	246.85	49.78	2.31
Pdlim7	1884.16	380.61	2.31
Gm5417	103.17	20.74	2.31
Plac8	1429.99	288.31	2.31
Tsc22d1	48257.21	9731.85	2.31
Btg2	2696.06	543.43	2.31
Rtn4rl2	144.64	29.04	2.31
Kbtbd13	21.21	4.15	2.32
Ypel2	2684.49	537.20	2.32
Pf4	99.32	19.71	2.33
Sele	57.85	11.41	2.33
-	392.45	77.78	2.33
Ltbp2	68.46	13.48	2.33
Krt18	2333.50	461.50	2.34
Lrrc73	53.03	10.37	2.34
Trpv6	338.45	66.38	2.35
AI506816	2314.21	454.24	2.35
Gpr141	16.39	3.11	2.35
Ube2t	37.60	7.26	2.35
Zfp239	334.59	65.34	2.35
Il34	3353.68	655.43	2.36
Dpysl3	1121.43	218.82	2.36
Slc39a4	43.39	8.30	2.37
Fosl2	4158.84	803.73	2.37
Col1a1	4546.47	877.36	2.37
Edn1	382.81	73.63	2.38
Acsbg1	157.17	30.08	2.38
Nov	22.17	4.15	2.38
Gm26669	162.96	31.12	2.38
Slpi	108.96	20.74	2.39
Gm41144	38.57	7.26	2.39
Ptprn	131.14	24.89	2.39
Ms4a6d	147.53	28.00	2.39
Cxcl14	1039.47	197.05	2.40
E2f8	44.35	8.30	2.40
Cd300lb	27.96	5.19	2.40
Hpcal4	358.70	67.41	2.41
Syt15	50.14	9.34	2.41
Cd177	11.57	2.08	2.41
Gm29292	11.57	2.08	2.41
Gm10286	11.57	2.08	2.41
Gm48127	11.57	2.08	2.41
Anp32b-ps1	11.57	2.08	2.41
Mx1	72.32	13.48	2.41
Gm16174	61.71	11.41	2.42
Anks1b	39.53	7.26	2.43
Itgam	134.99	24.89	2.43
Mefv	17.35	3.11	2.43
Gm7774	17.35	3.11	2.43
Hcar2	17.35	3.11	2.43
Ms4a7	79.07	14.52	2.44
Cdsn	79.07	14.52	2.44
Ptrh1	729.94	134.82	2.44
Anxa2	9018.69	1666.57	2.44
Nrg1	2934.23	540.32	2.44

Uhrf1	760.80	140.01	2.44
9530034E10Rik	28.92	5.19	2.45
D430020J02Rik	34.71	6.23	2.46
Ankle1	34.71	6.23	2.46
Hmga2	34.71	6.23	2.46
Tub	34.71	6.23	2.46
Csf1	2354.71	428.31	2.46
Dusp10	171.63	31.12	2.46
Gch1	339.42	61.19	2.47
Gm21887	477.30	86.08	2.47
Crybg2	375.09	67.41	2.47
Mmp19	202.49	36.30	2.48
Chsy3	35.67	6.23	2.50
Adgrg2	106.07	18.67	2.50
Bcl2a1d	29.89	5.19	2.50
Tgm1	5498.19	970.70	2.50
Gprin2	18.32	3.11	2.51
Gm38248	18.32	3.11	2.51
-	1805.09	314.23	2.52
Tnfrsf9	12.53	2.08	2.53
1700110K17Rik	12.53	2.08	2.53
Vstm2b	12.53	2.08	2.53
Rnf225	12.53	2.08	2.53
Fndc8	12.53	2.08	2.53
Gm30301	12.53	2.08	2.53
C5ar1	317.24	54.97	2.53
Gpr35	150.42	25.93	2.53
Prr7	36.64	6.23	2.53
Anxa3	2826.23	487.42	2.54
Slfn9	162.96	28.00	2.54
Nfkbiz	2570.71	441.79	2.54
Kdelr3	236.24	40.45	2.54
Creb5	274.81	46.67	2.55
Nek5	80.03	13.48	2.56
Melk	25.07	4.15	2.56
Nuf2	37.60	6.23	2.57
Trib3	333.63	56.00	2.57
Epha2	1520.63	255.12	2.57
Postn	80.99	13.48	2.58
Arid5a	544.80	91.27	2.58
Asic2	50.14	8.30	2.58
Pilrb1	19.28	3.11	2.58
Gm7233	19.28	3.11	2.58
Fmn2	19.28	3.11	2.58
Vcan	87.74	14.52	2.59
Ubd	31.82	5.19	2.59
Oit3	31.82	5.19	2.59
AC169509.1	31.82	5.19	2.59
Chrnb1	1074.18	178.38	2.59
Serp2	69.42	11.41	2.59
Gm47430	51.10	8.30	2.60
Gm7327	51.10	8.30	2.60
Eomes	38.57	6.23	2.61
Ccl9	248.78	40.45	2.62
Fam83c	77.14	12.45	2.62
Bdkrb2	364.49	59.12	2.62
C3	19162.68	3097.72	2.63
Psors1c2	13.50	2.08	2.63
Gm5530	13.50	2.08	2.63
-	13.50	2.08	2.63
Sfn	1735.66	278.97	2.64
Krt5	161.99	25.93	2.64

Hspb1	2749.09	440.76	2.64
Areg	39.53	6.23	2.64
Tpx2	98.35	15.56	2.65
Fanci	46.28	7.26	2.65
Gm5431	20.25	3.11	2.65
Ms4a4a	20.25	3.11	2.65
Bnipl	20.25	3.11	2.65
Cebpd	1998.90	315.27	2.66
-	434.88	68.45	2.67
Gm8989	27.00	4.15	2.67
Capn13	27.00	4.15	2.67
Fcer2a	27.00	4.15	2.67
Gm26674	40.50	6.23	2.68
Klf6	7658.13	1164.63	2.72
Pcsk2	21.21	3.11	2.72
Fgf21	124.39	18.67	2.73
Tdrd1	14.46	2.08	2.73
9630050E16Rik	14.46	2.08	2.73
Gm4651	14.46	2.08	2.73
Ch25h	305.67	45.63	2.74
Ccne1	83.89	12.45	2.74
Gm20706	42.42	6.23	2.75
Hhipl1	126.31	18.67	2.75
Junb	4221.51	626.39	2.75
Dlgap5	28.92	4.15	2.77
Sgo1	28.92	4.15	2.77
S100a6	3048.98	446.98	2.77
Ccl7	64.60	9.34	2.77
Nxnl2	64.60	9.34	2.77
Naip1	57.85	8.30	2.78
B430306N03Rik	22.17	3.11	2.78
Ccr1	172.60	24.89	2.79
Nipal1	136.92	19.71	2.79
Ccl12	86.78	12.45	2.79
Cxcr2	73.28	10.37	2.81
Syt8	58.82	8.30	2.81
Cenpi	29.89	4.15	2.81
Adcyap1r1	205.38	29.04	2.82
Btbd17	15.43	2.08	2.82
Lypd3	15.43	2.08	2.82
Baalc	15.43	2.08	2.82
Prtn3	15.43	2.08	2.82
Gm19774	15.43	2.08	2.82
Gm43336	15.43	2.08	2.82
Akap12	4222.48	589.06	2.84
Neto2	30.85	4.15	2.86
Lrp8	709.69	97.49	2.86
Trim72	38.57	5.19	2.87
Relb	865.90	118.23	2.87
-	161.99	21.78	2.89
Lrg1	4770.18	644.02	2.89
Glipr2	524.55	70.52	2.89
Adamts4	1152.28	154.53	2.90
Pcdh8	47.25	6.23	2.90
Pbk	39.53	5.19	2.90
Klhl41	24.10	3.11	2.90
9330188P03Rik	24.10	3.11	2.90
8430423G03Rik	16.39	2.08	2.91
-	16.39	2.08	2.91
Aloxe3	16.39	2.08	2.91
F730043M19Rik	86.78	11.41	2.91
2200002D01Rik	1976.72	261.34	2.92

Eps8l3	275.77	36.30	2.92
Gm18935	8.68	1.04	2.92
Gm13776	8.68	1.04	2.92
AC133488.1	8.68	1.04	2.92
Csf3	8.68	1.04	2.92
Gm37735	8.68	1.04	2.92
9530053A07Rik	8.68	1.04	2.92
Gm33024	8.68	1.04	2.92
Gm30054	8.68	1.04	2.92
Gm25134	8.68	1.04	2.92
Gm17227	8.68	1.04	2.92
Tmem156	8.68	1.04	2.92
Gm17028	8.68	1.04	2.92
D5Ertd605e	8.68	1.04	2.92
CT030636.2	8.68	1.04	2.92
2610318N02Rik	8.68	1.04	2.92
Adipoq	8.68	1.04	2.92
Egr1	2107.86	277.94	2.92
Aurkb	55.92	7.26	2.92
Slc25a24	1348.03	176.30	2.93
Ccl2	192.85	24.89	2.95
Krt19	556.37	71.56	2.96
-	339.42	43.56	2.96
Gm13241	25.07	3.11	2.96
Sertad4	672.08	86.08	2.96
Tnfrsf23	237.20	30.08	2.97
Cep55	17.35	2.08	2.99
Foxj1	117.64	14.52	3.01
Cdca7	210.21	25.93	3.01
Col3a1	8250.18	1016.33	3.02
Klf5	346.17	42.52	3.02
Dusp5	506.23	62.23	3.02
Cyr61	8544.28	1050.55	3.02
Fut1	119.56	14.52	3.03
Saa1	214.06	25.93	3.04
Gm3788	52.07	6.23	3.04
Clcf1	2338.32	284.16	3.04
Ifitm10	208.28	24.89	3.06
Slc7a11	35.67	4.15	3.07
Sirpb1c	18.32	2.08	3.07
Gm29585	18.32	2.08	3.07
Kif18b	9.64	1.04	3.07
Dhrs9	9.64	1.04	3.07
Gm8783	9.64	1.04	3.07
Tsnaxip1	9.64	1.04	3.07
Gm5121	9.64	1.04	3.07
Dcdc2a	2998.84	354.68	3.08
Samd5	504.30	59.12	3.09
Kcnh1	297.95	34.23	3.12
Gxylt2	82.92	9.34	3.13
Esco2	37.60	4.15	3.14
Sec1	37.60	4.15	3.14
-	147.53	16.60	3.14
Clspn	83.89	9.34	3.15
Myc	2314.21	259.27	3.16
Rgs16	140.78	15.56	3.17
Gm49146	140.78	15.56	3.17
Cxcl10	619.05	68.45	3.17
Fhad1	38.57	4.15	3.18
Cxcl17	48.21	5.19	3.19
Msr1	153.31	16.60	3.20
Rnf165	10.60	1.04	3.21

AC107711.1	10.60	1.04	3.21
Gm48632	10.60	1.04	3.21
Moxd1	10.60	1.04	3.21
Serpinb9b	10.60	1.04	3.21
Gm16178	10.60	1.04	3.21
Gm36161	10.60	1.04	3.21
Gm48129	10.60	1.04	3.21
Pth2r	10.60	1.04	3.21
Gm28085	10.60	1.04	3.21
Gm42930	10.60	1.04	3.21
Gm34552	10.60	1.04	3.21
Gm3364	10.60	1.04	3.21
Krt17	10.60	1.04	3.21
Gm11714	20.25	2.08	3.21
Ccl11	39.53	4.15	3.21
Serpina3n	383.77	40.45	3.24
Hfm1	30.85	3.11	3.26
Gdf15	3922.59	404.46	3.28
Aard	21.21	2.08	3.28
Mdfi	111.85	11.41	3.28
Cldn4	5266.77	538.24	3.29
Gm14005	42.42	4.15	3.32
Gm6745	11.57	1.04	3.33
Gm47513	11.57	1.04	3.33
Ttc30a2	11.57	1.04	3.33
Ap3b2	11.57	1.04	3.33
Hesx1	220.81	21.78	3.33
Runx1	389.56	38.37	3.34
Dnmt3l	22.17	2.08	3.34
Cd44	464.77	45.63	3.34
Gjb3	32.78	3.11	3.35
Serpina7	54.00	5.19	3.35
Adam8	301.81	29.04	3.37
-	54.96	5.19	3.37
Olr1	44.35	4.15	3.38
Klrg2	559.27	52.89	3.40
Cd68	624.84	58.08	3.42
Selp	34.71	3.11	3.43
Gm44040	12.53	1.04	3.45
Prph	12.53	1.04	3.45
Casp14	12.53	1.04	3.45
Klrb1b	12.53	1.04	3.45
1700019L13Rik	12.53	1.04	3.45
Nrcam	160.06	14.52	3.45
Ppm1j	816.72	72.60	3.49
Akr1b8	958.47	85.04	3.49
Bcl3	2331.57	205.34	3.50
Clec4n	25.07	2.08	3.52
Slfn10-ps	25.07	2.08	3.52
Clu	88234.34	7694.01	3.52
Tnfrsf12a	11057.13	956.18	3.53
Gpnmb	74.24	6.23	3.55
Clca3a2	74.24	6.23	3.55
Dpf1	13.50	1.04	3.55
Nlrp1a	13.50	1.04	3.55
Mir1943	13.50	1.04	3.55
Gm14046	13.50	1.04	3.55
1700061E18Rik	13.50	1.04	3.55
Dclk1	152.35	12.45	3.60
Fgg	36295.60	2958.76	3.62
Cd14	1836.91	149.34	3.62
-	320.13	25.93	3.62

Togaram2	14.46	1.04	3.65
Cemip	225.63	17.63	3.67
Gm44284	27.96	2.08	3.68
Hk2	452.23	35.26	3.68
Inhbb	921.83	71.56	3.69
Pla2g2e	54.96	4.15	3.69
Arg1	68.46	5.19	3.69
-	163.92	12.45	3.71
Cdkn3	28.92	2.08	3.72
2810430I11Rik	15.43	1.04	3.74
Gm15819	15.43	1.04	3.74
Clec2l	15.43	1.04	3.74
Cldn14	43.39	3.11	3.75
Cxcl5	85.82	6.23	3.76
Il6	44.35	3.11	3.78
Dusp8	1541.84	112.01	3.78
Ccl5	247.81	17.63	3.80
Calml3	132.10	9.34	3.81
Rnf39	88.71	6.23	3.81
Fosl1	511.05	36.30	3.81
Col17a1	45.32	3.11	3.81
Clec4e	30.85	2.08	3.82
Rnf223	16.39	1.04	3.83
Ackr2	16.39	1.04	3.83
AC117241.2	16.39	1.04	3.83
Lgi2	341.34	23.86	3.83
Spp1	634319.15	44252.73	3.84
Gabrp	32.78	2.08	3.90
Ticrr	17.35	1.04	3.91
Begain	17.35	1.04	3.91
Gm35189	17.35	1.04	3.91
Mybpc2	64.60	4.15	3.92
Meltf	97.39	6.23	3.94
Socs3	2942.91	189.79	3.95
Mmp7	98.35	6.23	3.96
Vtcn1	582.41	37.34	3.96
Plaur	2175.36	137.93	3.98
Fga	49219.53	3117.43	3.98
Ankrd1	575.66	36.30	3.98
Has2	18.32	1.04	3.99
Cxcl3	18.32	1.04	3.99
Maff	1650.81	102.67	4.01
Aldh1a2	3183.01	196.01	4.02
Ifi27l2b	19.28	1.04	4.06
Krt4	19.28	1.04	4.06
Tmem95	19.28	1.04	4.06
-	36.64	2.08	4.06
Rnd1	394.38	22.82	4.10
Gm49123	20.25	1.04	4.13
AA467197	93.53	5.19	4.14
Mmp3	76.17	4.15	4.16
Tnc	1791.59	97.49	4.20
Gm42791	21.21	1.04	4.20
Clec4d	42.42	2.08	4.27
Smpdl3b	647.98	33.19	4.28
Atf3	853.36	43.56	4.29
Krt14	88.71	4.15	4.38
Slc34a2	2704.74	129.64	4.38
Egr3	24.10	1.04	4.38
Muc13	133.06	6.23	4.39
Gm9844	25.07	1.04	4.44
Gpbar1	26.03	1.04	4.49

G930009F23Rik	96.42	4.15	4.50
Tmem145	147.53	6.23	4.54
Tubb3	27.96	1.04	4.60
Gm42793	1105.04	44.60	4.63
Flnc	554.44	21.78	4.66
Medag	109.92	4.15	4.69
Trem1	29.89	1.04	4.69
-	140.78	5.19	4.73
Gm15894	31.82	1.04	4.78
Lcn2	14649.95	500.91	4.87
Mdga1	34.71	1.04	4.91
Egr2	343.27	10.37	5.03
Sox9	1406.85	39.41	5.15
Cxcl1	2462.71	66.38	5.21
Serpine1	1609.34	40.45	5.31
Sprr1a	1896.69	46.67	5.34
4930546K05Rik	5.78	0.00	5.56
Cfap97d1	5.78	0.00	5.56
Tex14	5.78	0.00	5.56
Tnip3	5.78	0.00	5.56
Apol7e	5.78	0.00	5.56
4833417C18Rik	5.78	0.00	5.56
Gm14376	5.78	0.00	5.56
Ankrd2	5.78	0.00	5.56
4930426L09Rik	5.78	0.00	5.56
Pla2g1b	5.78	0.00	5.56
S100a3	5.78	0.00	5.56
Cers1	5.78	0.00	5.56
Gpr31b	5.78	0.00	5.56
Garem2	5.78	0.00	5.56
Osm	5.78	0.00	5.56
Gm8229	5.78	0.00	5.56
Dspp	5.78	0.00	5.56
Gm16244	5.78	0.00	5.56
Gm15406	5.78	0.00	5.56
Gm19040	5.78	0.00	5.56
5330413D20Rik	5.78	0.00	5.56
Gm45745	5.78	0.00	5.56
Apol6	5.78	0.00	5.56
Gm47580	5.78	0.00	5.56
Prss46	5.78	0.00	5.56
Gm31805	5.78	0.00	5.56
-	5.78	0.00	5.56
Gm43162	5.78	0.00	5.56
Gm43489	5.78	0.00	5.56
Lypd1	5.78	0.00	5.56
Crabp2	5.78	0.00	5.56
Gm35715	5.78	0.00	5.56
Rps2-ps5	5.78	0.00	5.56
Gm14472	5.78	0.00	5.56
Gm12219	5.78	0.00	5.56
2810442N19Rik	5.78	0.00	5.56
Gm45757	5.78	0.00	5.56
Gm28818	5.78	0.00	5.56
Gm12540	5.78	0.00	5.56
Gm49141	5.78	0.00	5.56
Gm16599	5.78	0.00	5.56
Gm47118	5.78	0.00	5.56
Cyp21a1	5.78	0.00	5.56
Gm9476	5.78	0.00	5.56
Gm13074	5.78	0.00	5.56
Ascl2	5.78	0.00	5.56

Snord49b	5.78	0.00	5.56
Sapcd2	5.78	0.00	5.56
Snora43	5.78	0.00	5.56
Mageb3	5.78	0.00	5.56
Inhba	161.03	3.11	5.64
Fut2	163.92	3.11	5.66
Acod1	6.75	0.00	5.78
Fpr1	6.75	0.00	5.78
1700086P04Rik	6.75	0.00	5.78
Gm38244	6.75	0.00	5.78
Gm13836	6.75	0.00	5.78
Gm38375	6.75	0.00	5.78
Gm12168	6.75	0.00	5.78
Zdbf2	6.75	0.00	5.78
Gm43792	6.75	0.00	5.78
Serpina3i	6.75	0.00	5.78
Gm38365	6.75	0.00	5.78
Syt5	6.75	0.00	5.78
Spdef	6.75	0.00	5.78
Gm17222	6.75	0.00	5.78
Gm29596	6.75	0.00	5.78
Ap3m1-ps	6.75	0.00	5.78
Gm8960	6.75	0.00	5.78
Gm9830	6.75	0.00	5.78
Gm44833	6.75	0.00	5.78
Gm13193	6.75	0.00	5.78
AC133498.1	6.75	0.00	5.78
Gm43013	6.75	0.00	5.78
F930017D23Rik	6.75	0.00	5.78
Gm20778	6.75	0.00	5.78
Gm17097	6.75	0.00	5.78
Gm26860	6.75	0.00	5.78
Gm12630	6.75	0.00	5.78
1700016G22Rik	6.75	0.00	5.78
Gm5799	6.75	0.00	5.78
Pcdha12	6.75	0.00	5.78
4931440J10Rik	6.75	0.00	5.78
A530030E21Rik	6.75	0.00	5.78
Gm22816	6.75	0.00	5.78
Gm16546	6.75	0.00	5.78
Gm26386	6.75	0.00	5.78
Wfdc21	7.71	0.00	5.97
Cdh17	7.71	0.00	5.97
Cacng1	7.71	0.00	5.97
Gm10375	7.71	0.00	5.97
Gm28068	7.71	0.00	5.97
Gjb5	7.71	0.00	5.97
4930593A02Rik	7.71	0.00	5.97
Gm19590	7.71	0.00	5.97
Gm8883	7.71	0.00	5.97
Serpinb5	7.71	0.00	5.97
Gm44066	7.71	0.00	5.97
Gm36377	7.71	0.00	5.97
Gm22847	7.71	0.00	5.97
Mir6913	7.71	0.00	5.97
Gm11832	7.71	0.00	5.97
Gm48958	7.71	0.00	5.97
Olfr373	7.71	0.00	5.97
Gm3573	7.71	0.00	5.97
Cav3	8.68	0.00	6.14
Plb1	8.68	0.00	6.14
Gm5150	8.68	0.00	6.14

Gm7893	8.68	0.00	6.14
Il24	8.68	0.00	6.14
1700093K21Rik	8.68	0.00	6.14
4833407H14Rik	8.68	0.00	6.14
Zfp652os	8.68	0.00	6.14
4930503E14Rik	8.68	0.00	6.14
Mir6909	8.68	0.00	6.14
Dok5	8.68	0.00	6.14
Gm650	8.68	0.00	6.14
Gm45425	8.68	0.00	6.14
Gml2	8.68	0.00	6.14
3930402G23Rik	8.68	0.00	6.14
Abhd16b	8.68	0.00	6.14
Gm48314	8.68	0.00	6.14
Cdh26	8.68	0.00	6.14
Gm3294	8.68	0.00	6.14
Tnfaip6	8.68	0.00	6.14
-	8.68	0.00	6.14
Igkv1-99	8.68	0.00	6.14
Il1rn	386.66	5.19	6.19
Serpina10	2772.24	36.30	6.25
Ripply1	9.64	0.00	6.29
-	9.64	0.00	6.29
Mir7061	9.64	0.00	6.29
BC061237	9.64	0.00	6.29
Mmp20	9.64	0.00	6.29
Ntn5	9.64	0.00	6.29
Fgb	12239.31	151.42	6.34
Timp1	1382.74	16.60	6.37
Il11	439.70	5.19	6.37
Tlr5	10.60	0.00	6.42
Gad1-ps	10.60	0.00	6.42
-	10.60	0.00	6.42
Erfe	10.60	0.00	6.42
Defb36	10.60	0.00	6.42
Raet1e	10.60	0.00	6.42
Ereg	10.60	0.00	6.42
BC100530	10.60	0.00	6.42
Mmp24	10.60	0.00	6.42
Ccr4	10.60	0.00	6.42
Gjb4	100.28	1.04	6.43
D630011A20Rik	11.57	0.00	6.55
Tmem40	11.57	0.00	6.55
Gm32857	11.57	0.00	6.55
E230025N22Rik	11.57	0.00	6.55
Gm38563	11.57	0.00	6.55
Krt20	3268.83	34.23	6.57
A2m	117.64	1.04	6.66
Gm16998	12.53	0.00	6.66
St8sia2	12.53	0.00	6.66
Stfa2l1	12.53	0.00	6.66
Odaph	12.53	0.00	6.66
Gm10521	12.53	0.00	6.66
Gm807	12.53	0.00	6.66
Slc26a9	12.53	0.00	6.66
AU015791	12.53	0.00	6.66
Zfp575	13.50	0.00	6.77
Tmem59l	13.50	0.00	6.77
Fsd1	13.50	0.00	6.77
Gm42986	13.50	0.00	6.77
Serpina3m	13.50	0.00	6.77
Mup-ps23	14.46	0.00	6.87

Tnni2	14.46	0.00	6.87
Gml	14.46	0.00	6.87
Crlf1	639.30	5.19	6.91
Gm16098	15.43	0.00	6.96
Serpina6	15.43	0.00	6.96
1700016P03Rik	15.43	0.00	6.96
Gm5833	16.39	0.00	7.05
Uox	19.28	0.00	7.28
Grp	20.25	0.00	7.35
Unc5cl	21.21	0.00	7.42
Gfra2	21.21	0.00	7.42
Lif	2345.07	13.48	7.43
Gm43745	22.17	0.00	7.48
Muc4	210.21	1.04	7.50
Upk3bl	23.14	0.00	7.54
Gm45424	23.14	0.00	7.54
Cxcl2	226.60	1.04	7.61
Nppb	25.07	0.00	7.66
2900011O08Rik	25.07	0.00	7.66
Wnt7a	26.03	0.00	7.71
Spaca7	27.00	0.00	7.76
Nptx2	27.00	0.00	7.76
Gm15199	29.89	0.00	7.91
Sprr2g	794.54	3.11	7.94
Havcr1	40346.44	127.56	8.30
Ccl20	417.52	1.04	8.49
-	45.32	0.00	8.51
Crct1	46.28	0.00	8.54
Has1	52.07	0.00	8.71
Zfp469	59.78	0.00	8.91
Tnfsf18	59.78	0.00	8.91
Gm15867	62.67	0.00	8.97
Prss22	68.46	0.00	9.10
Vgf	382.81	0.00	11.58
Sprr2f	400.16	0.00	11.65
Il1f6	692.33	0.00	12.44

Table S4: Differential expressed genes of WT_IR3D vs WT

gene_name	WT_IR1 readcount	WT_IR2 readcount	WT1 readcount	WT2 readcount	log2FoldChange
Il1f6	210.11	200.08	0.00	0.00	10.00
Cxcl2	123.54	259.42	0.00	0.00	9.90
Arhgap36	63.23	142.43	0.00	0.00	9.00
Mmp7	74.90	86.47	0.00	0.00	8.65
Timp1	393.96	466.29	1.07	1.12	8.62
Vgf	74.90	80.54	0.00	0.00	8.60
Ccl2	341.43	479.85	2.14	0.00	8.56
Ccl7	71.98	67.82	0.00	0.00	8.44
Havcr1	13683.53	13729.13	33.11	49.12	8.39
Cxcl5	54.47	61.89	0.00	0.00	8.18
Prss22	59.34	49.17	0.00	0.00	8.08
Gjb5	39.88	43.24	0.00	0.00	7.69
Mmp24	41.83	40.69	0.00	0.00	7.68
Il1rn	256.80	155.15	2.14	0.00	7.56
Sprr2f	145.91	53.41	0.00	1.12	7.51
Igdcc4	33.07	33.91	0.00	0.00	7.38
Serpina3i	44.75	18.65	0.00	0.00	7.30
Cxcl17	70.04	90.71	0.00	1.12	7.20
Serpinb9b	31.13	27.98	0.00	0.00	7.20
Xirp2	50.58	7.63	0.00	0.00	7.17
Clec4d	34.05	22.04	0.00	0.00	7.12
Medag	31.13	22.04	0.00	0.00	7.05
Crlf1	253.89	571.41	2.14	4.47	6.98
Krt20	931.88	940.20	5.34	10.05	6.94
Saa1	184.82	205.17	2.14	1.12	6.90
Adcy8	21.40	23.74	0.00	0.00	6.81
Saa3	17.51	25.43	0.00	0.00	6.74
Trem2	7.78	34.76	0.00	0.00	6.73
Ccl12	78.79	36.46	1.07	0.00	6.72
Popdc3	4.86	37.30	0.00	0.00	6.72
Gm12963	22.37	18.65	0.00	0.00	6.67
Mcub	129.37	89.87	1.07	1.12	6.65
Ifi27l2b	21.40	17.80	0.00	0.00	6.61
Cd44	1997.03	1608.26	14.95	22.33	6.60
Ivl	29.18	8.48	0.00	0.00	6.55
Themis	19.45	17.80	0.00	0.00	6.53
Gm21188	37.94	58.50	1.07	0.00	6.47
Mmp27	15.56	19.50	0.00	0.00	6.45
Col17a1	159.53	121.23	1.07	2.23	6.42
Vcam1	8032.89	9327.40	85.44	125.04	6.37
Sult1e1	5.84	27.13	0.00	0.00	6.36
Wnt7a	18.48	14.41	0.00	0.00	6.35
-	14.59	17.80	0.00	0.00	6.33
Has2	15.56	15.26	0.00	0.00	6.26
Krt17	26.26	4.24	0.00	0.00	6.24
-	16.54	12.72	0.00	0.00	6.19
Dnmt3l	20.43	8.48	0.00	0.00	6.17
AC122397.1	123.54	111.06	1.07	2.23	6.16
Sftpd	15.56	12.72	0.00	0.00	6.14
H2-M2	18.48	9.33	0.00	0.00	6.11
Pcsk2	9.73	17.80	0.00	0.00	6.10
-	1.95	24.59	0.00	0.00	6.05
Cdh17	14.59	11.87	0.00	0.00	6.04
Samd3	6.81	19.50	0.00	0.00	6.04
Tdrd1	10.70	15.26	0.00	0.00	6.02
Defb36	26.26	44.09	0.00	1.12	6.01
Slco5a1	12.65	12.72	0.00	0.00	5.98

Htr2b	19.45	5.93	0.00	0.00	5.98
Cuzd1	19.45	5.93	0.00	0.00	5.98
Serpina10	1871.55	1698.97	20.29	36.84	5.97
Gal3st2	8.75	16.11	0.00	0.00	5.95
Ccl20	33.07	33.91	1.07	0.00	5.94
Lcn2	6547.51	6769.61	128.15	88.20	5.94
Tnip3	9.73	14.41	0.00	0.00	5.91
Lif	606.99	771.49	6.41	16.75	5.91
Mmp3	47.66	83.08	0.00	2.23	5.90
Sit1	12.65	11.02	0.00	0.00	5.88
Ubd	346.30	374.72	0.00	12.28	5.88
Mefv	36.96	27.13	1.07	0.00	5.88
Dnah8	13.62	9.33	0.00	0.00	5.83
Clec4n	72.96	50.87	2.14	0.00	5.83
Cxcl1	979.55	818.12	11.75	20.10	5.83
Padi4	11.67	11.02	0.00	0.00	5.82
Gad1-ps	13.62	8.48	0.00	0.00	5.78
Hcar2	10.70	11.02	0.00	0.00	5.76
Ms4a6d	218.87	194.14	3.20	4.47	5.75
Cfap221	8.75	12.72	0.00	0.00	5.74
Col3a1	14697.13	13234.02	234.95	294.73	5.72
Nnmt	111.87	118.69	1.07	3.35	5.72
AC140209.1	11.67	9.33	0.00	0.00	5.71
CR974586.3	9.73	11.02	0.00	0.00	5.69
Ifi205	26.26	29.67	1.07	0.00	5.68
Nlrp1c-ps	7.78	12.72	0.00	0.00	5.68
Slc1a6	7.78	12.72	0.00	0.00	5.68
Mmp10	14.59	5.93	0.00	0.00	5.67
E230025N22Rik	6.81	13.56	0.00	0.00	5.67
Ccdc33	12.65	7.63	0.00	0.00	5.66
Aldh3a1	18.48	1.70	0.00	0.00	5.64
Ndp	7.78	11.87	0.00	0.00	5.61
2210406H18Rik	7.78	11.87	0.00	0.00	5.61
Iapp	7.78	11.87	0.00	0.00	5.61
Gm8165	6.81	12.72	0.00	0.00	5.61
4933424M12Rik	6.81	12.72	0.00	0.00	5.61
Cxcr2	35.02	17.80	1.07	0.00	5.60
Fcrls	245.13	176.34	2.14	6.70	5.59
Sox11	114.78	94.10	4.27	0.00	5.58
Cpz	31.13	21.19	1.07	0.00	5.58
Gm48709	54.47	49.17	1.07	1.12	5.57
9530034E10Rik	9.73	9.33	0.00	0.00	5.57
Sprr2g	236.38	69.52	4.27	2.23	5.55
AC170810.2	5.84	12.72	0.00	0.00	5.53
C3	57529.78	51304.09	838.34	1533.95	5.52
Gm8206	11.67	6.78	0.00	0.00	5.52
Gjb4	140.07	105.97	5.34	0.00	5.51
Orm2	31.13	17.80	0.00	1.12	5.48
Pvrig	6.81	11.02	0.00	0.00	5.47
Gpr174	4.86	12.72	0.00	0.00	5.46
Siglec5	10.70	6.78	0.00	0.00	5.44
Postn	442.60	160.23	4.27	10.05	5.40
Cd300lb	59.34	33.06	0.00	2.23	5.40
Serpina3n	412.44	550.22	7.48	15.63	5.39
Tubb3	30.15	15.26	0.00	1.12	5.37
Pglyrp2	106.03	119.54	2.14	3.35	5.37
Ms4a8a	9.73	6.78	0.00	0.00	5.36
5830428M24Rik	8.75	7.63	0.00	0.00	5.35
Gm5150	28.21	16.11	1.07	0.00	5.34
B430306N03Rik	14.59	1.70	0.00	0.00	5.34
Gpr65	87.55	87.32	2.14	2.23	5.32
Gm47662	4.86	11.02	0.00	0.00	5.31

Gm9733	11.67	4.24	0.00	0.00	5.30
Aqp9	26.26	16.96	0.00	1.12	5.30
Efhb	3.89	11.87	0.00	0.00	5.30
Sox9	938.69	1161.47	18.16	36.84	5.26
Prss27	7.78	7.63	0.00	0.00	5.26
Gm12426	6.81	8.48	0.00	0.00	5.25
Tmprss5	6.81	8.48	0.00	0.00	5.25
Zfp469	63.23	61.04	2.14	1.12	5.25
Kcna3	13.62	1.70	0.00	0.00	5.25
Col1a1	11021.14	9264.66	241.36	294.73	5.24
Aldh1a2	1242.19	1292.03	35.24	32.38	5.23
Cxcl10	1119.62	2163.56	33.11	54.70	5.23
Saa2	3.89	11.02	0.00	0.00	5.22
Acod1	10.70	4.24	0.00	0.00	5.21
Nxpe5	61.28	19.50	2.14	0.00	5.21
Osm	8.75	5.93	0.00	0.00	5.19
Cxcl3	8.75	5.93	0.00	0.00	5.19
Fcna	7.78	6.78	0.00	0.00	5.18
Gm30301	7.78	6.78	0.00	0.00	5.18
Igkv8-24	0.00	14.41	0.00	0.00	5.17
Gm8979	6.81	7.63	0.00	0.00	5.17
Meltf	80.74	75.45	4.27	0.00	5.17
Art2b	17.51	21.19	1.07	0.00	5.15
Tmem156	3.89	10.17	0.00	0.00	5.13
Gm47567	3.89	10.17	0.00	0.00	5.13
Pcdhb15	10.70	3.39	0.00	0.00	5.13
Ighv9-3	1.95	11.87	0.00	0.00	5.11
Gm45425	8.75	5.09	0.00	0.00	5.10
Orm1	8.75	5.09	0.00	0.00	5.10
Rgs20	7.78	5.93	0.00	0.00	5.09
Gm1070	6.81	6.78	0.00	0.00	5.08
Msr1	180.93	147.52	5.34	4.47	5.06
Nipal4	11.67	1.70	0.00	0.00	5.05
-	189.68	204.32	4.27	7.81	5.03
Fut2	55.45	51.72	1.07	2.23	5.03
9930111J21Rik1	9.73	3.39	0.00	0.00	5.03
Ccl9	280.15	285.71	7.48	10.05	5.02
Lrrc55	8.75	4.24	0.00	0.00	5.01
Gpr141	15.56	19.50	0.00	1.12	5.00
Hsp25-ps1	6.81	5.93	0.00	0.00	4.99
Serpina3h	6.81	5.93	0.00	0.00	4.99
Olfr1417	6.81	5.93	0.00	0.00	4.99
Rgs16	172.17	237.38	4.27	8.93	4.96
AC147041.2	3.89	8.48	0.00	0.00	4.95
Madcam1	15.56	17.80	1.07	0.00	4.94
Gm16098	15.56	17.80	0.00	1.12	4.93
Gm36161	38.91	60.19	2.14	1.12	4.92
Begain	13.62	19.50	0.00	1.12	4.92
Smpdl3b	328.79	398.46	10.68	13.40	4.92
Kcna6	20.43	12.72	0.00	1.12	4.92
Klhl33	12.65	20.35	0.00	1.12	4.92
Barx2	0.97	11.02	0.00	0.00	4.91
Gm33091	0.97	11.02	0.00	0.00	4.91
Gm15867	16.54	16.11	1.07	0.00	4.91
Fxyd3	7.78	4.24	0.00	0.00	4.90
Htra3	192.60	100.04	3.20	6.70	4.89
Ccl8	6.81	5.09	0.00	0.00	4.89
Fpr2	6.81	5.09	0.00	0.00	4.89
Gm6133	6.81	5.09	0.00	0.00	4.89
Gm8773	6.81	5.09	0.00	0.00	4.89
Slc7a11	20.43	11.87	0.00	1.12	4.88
Vwa3b	5.84	5.93	0.00	0.00	4.87

Thy1	651.74	524.78	17.09	23.44	4.86
Apod	22.37	9.33	1.07	0.00	4.86
-	3.89	7.63	0.00	0.00	4.84
Gm13074	3.89	7.63	0.00	0.00	4.84
-	135.21	84.78	2.14	5.58	4.84
Gm13241	20.43	11.02	0.00	1.12	4.84
Shisa6	2.92	8.48	0.00	0.00	4.83
Sprn	2.92	8.48	0.00	0.00	4.83
Arg1	23.35	7.63	1.07	0.00	4.83
Gm42793	451.35	290.79	14.95	11.16	4.82
Tlr8	66.15	57.65	1.07	3.35	4.82
Aoc1	4177.92	3327.58	171.94	97.13	4.80
Cd7	4.86	25.43	0.00	1.12	4.79
Ccr1	222.76	164.47	9.61	4.47	4.77
Gabrp	15.56	13.56	1.07	0.00	4.74
AC156790.4	15.56	13.56	1.07	0.00	4.74
Serpine1	350.19	256.03	7.48	15.63	4.72
Spib	65.17	49.17	2.14	2.23	4.71
Tnfrsf9	76.85	92.41	2.14	4.47	4.69
A2m	85.60	81.39	3.20	3.35	4.67
Gm45424	25.29	30.52	0.00	2.23	4.67
Tbc1d10c	31.13	23.74	2.14	0.00	4.66
Clec4a2	161.47	194.14	6.41	7.81	4.65
Adgra1	125.48	120.39	5.34	4.47	4.65
C920009B18Rik	12.65	14.41	1.07	0.00	4.64
Il1rl1	39.88	14.41	1.07	1.12	4.63
Itgam	175.09	201.77	7.48	7.81	4.62
Nptx2	11.67	15.26	0.00	1.12	4.62
Hk2	254.86	200.93	10.68	7.81	4.62
5033403H07Rik	16.54	10.17	1.07	0.00	4.61
P2ry13	38.91	40.69	1.07	2.23	4.60
Muc4	151.75	113.60	2.14	8.93	4.60
Ighv14-2	20.43	5.93	1.07	0.00	4.59
H2-DMb2	454.27	514.61	16.02	24.56	4.58
Zfp853	24.32	27.98	0.00	2.23	4.57
Socs3	1863.77	1704.91	82.23	68.10	4.57
Stx11	361.86	362.01	17.09	13.40	4.57
Ltbp2	138.13	222.97	6.41	8.93	4.56
Srsf12	15.56	10.17	1.07	0.00	4.56
5730420D15Rik	11.67	13.56	1.07	0.00	4.54
Clec4a1	181.90	147.52	5.34	8.93	4.53
Gm21451	44.75	5.93	0.00	2.23	4.52
Atf3	512.63	703.67	26.70	27.91	4.48
Ceacam16	11.67	12.72	0.00	1.12	4.48
2810430I11Rik	9.73	14.41	1.07	0.00	4.47
Tmprss6	15.56	33.06	0.00	2.23	4.47
Gpnmb	86.57	58.50	2.14	4.47	4.46
Mir6358	15.56	8.48	1.07	0.00	4.46
Clec12a	240.27	215.34	12.82	7.81	4.46
Lgi2	375.48	267.90	11.75	17.86	4.44
Bcl2a1a	12.65	11.02	1.07	0.00	4.44
Tnfrsf13b	101.16	110.21	4.27	5.58	4.43
Ankrd1	321.00	289.94	7.48	21.21	4.42
Lhx2	13.62	33.06	1.07	1.12	4.42
Kdelr3	162.45	116.15	5.34	7.81	4.41
Serpina3g	308.36	286.55	12.82	15.63	4.39
Fasl	13.62	9.33	0.00	1.12	4.39
Sprr1a	949.39	406.09	43.79	21.21	4.38
Cd200r1	21.40	23.74	2.14	0.00	4.38
Ms4a4a	48.64	65.28	1.07	4.47	4.38
Fhad1	18.48	27.13	0.00	2.23	4.38
Ptger2	11.67	11.02	0.00	1.12	4.37

Rnd1	192.60	182.28	11.75	6.70	4.34
Trib3	173.15	201.77	7.48	11.16	4.34
Krt4	19.45	2.54	1.07	0.00	4.33
Gm11515	5.84	16.11	0.00	1.12	4.33
Adam8	501.93	281.47	20.29	18.98	4.32
AC139671.1	16.54	5.09	1.07	0.00	4.31
Glipr2	359.91	288.25	14.95	17.86	4.31
Pdcd1	15.56	5.93	1.07	0.00	4.30
Acsbg1	128.40	172.10	5.34	10.05	4.30
F13a1	239.29	142.43	14.95	4.47	4.29
Mybpc2	155.64	78.00	8.54	3.35	4.29
Kel	30.15	33.06	0.00	3.35	4.26
Togaram2	10.70	10.17	0.00	1.12	4.25
Fcgr1	383.26	357.77	18.16	21.21	4.24
Trbc2	116.73	169.56	5.34	10.05	4.23
Ypel4	59.34	82.24	4.27	3.35	4.21
Cd5	69.06	74.61	0.00	7.81	4.21
Gm17028	10.70	9.33	1.07	0.00	4.20
Pf4	190.66	148.36	8.54	10.05	4.19
Cerkl	25.29	14.41	1.07	1.12	4.18
Sprr2a3	15.56	4.24	1.07	0.00	4.18
Mc2r	15.56	4.24	0.00	1.12	4.17
Raet1d	6.81	12.72	1.07	0.00	4.17
Dclk1	110.89	64.43	5.34	4.47	4.16
Bank1	30.15	8.48	2.14	0.00	4.15
Slfn4	71.98	25.43	2.14	3.35	4.15
Tlr2	1067.10	1256.43	65.14	65.87	4.15
Fam171b	24.32	14.41	1.07	1.12	4.15
Kcnt2	88.52	85.63	4.27	5.58	4.15
Krt14	35.02	3.39	2.14	0.00	4.14
C4bp	42.80	33.91	3.20	1.12	4.14
Gm13398	69.06	140.73	5.34	6.70	4.13
Ifitm6	10.70	8.48	0.00	1.12	4.13
Cd300lf	72.96	60.19	2.14	5.58	4.12
Tmem59l	8.75	10.17	0.00	1.12	4.11
Gfra2	7.78	11.02	0.00	1.12	4.10
Lonrf2	7.78	11.02	0.00	1.12	4.10
Sirpb1b	21.40	16.11	1.07	1.12	4.10
Ms4a4c	111.87	93.26	6.41	5.58	4.09
Pilra	88.52	60.19	4.27	4.47	4.09
Gli1	83.66	101.73	5.34	5.58	4.09
Gpr176	193.58	159.38	6.41	14.51	4.08
Fbxo48	12.65	5.93	0.00	1.12	4.08
Olr1	19.45	16.96	2.14	0.00	4.07
Gdf6	3.89	14.41	0.00	1.12	4.07
6030408B16Rik	20.43	16.11	1.07	1.12	4.06
Creb5	262.64	209.40	11.75	16.75	4.05
Adam19	476.64	355.22	23.49	26.79	4.05
Kcnk13	30.15	23.74	2.14	1.12	4.04
Kif1a	78.79	154.30	5.34	8.93	4.04
C5ar1	316.14	204.32	13.88	17.86	4.04
Ikzf3	60.31	83.08	3.20	5.58	4.04
Irf4	70.04	108.52	6.41	4.47	4.04
Runx2	103.11	145.82	8.54	6.70	4.03
Gm20612	6.81	11.02	0.00	1.12	4.03
Lyz2	4254.76	3459.84	283.01	192.02	4.02
P2ry12	27.24	44.09	0.00	4.47	4.02
Hspb1	4542.70	4631.48	301.16	267.94	4.01
Prrx1	192.60	139.89	5.34	15.63	4.00
Clec4e	25.29	9.33	2.14	0.00	3.99
Bpifc	38.91	48.32	1.07	4.47	3.99
Btk	75.87	80.54	2.14	7.81	3.99

Sh2d2a	71.01	85.63	1.07	8.93	3.99
Fgb	1128.38	1140.28	35.24	108.29	3.98
Cd276	130.35	94.10	7.48	6.70	3.98
Gpr34	21.40	47.48	2.14	2.23	3.98
Hpse	68.09	69.52	2.14	6.70	3.97
Cd180	124.51	182.28	12.82	6.70	3.97
Fam83c	55.45	64.43	2.14	5.58	3.97
Ccr2	842.39	982.59	38.45	79.27	3.96
Zfp683	12.65	21.19	0.00	2.23	3.94
Pirb	335.60	234.84	18.16	18.98	3.94
Cldn14	36.96	46.63	3.20	2.23	3.94
Cd84	146.88	136.49	9.61	8.93	3.93
Hk3	142.99	137.34	11.75	6.70	3.92
Siglec1	463.02	359.46	16.02	39.07	3.91
Adamts2	727.61	730.80	38.45	59.17	3.90
Ptprn	138.13	57.65	5.34	7.81	3.90
Ccl5	191.63	328.10	14.95	20.10	3.89
Lrrc25	188.71	167.01	14.95	8.93	3.89
Cdh11	884.22	696.88	41.65	65.87	3.88
Selp	29.18	19.50	0.00	3.35	3.88
Gzmb	39.88	56.80	1.07	5.58	3.88
Sirpb1a	49.61	47.48	0.00	6.70	3.88
Col6a3	2272.32	1604.87	131.36	132.85	3.87
Cyp4f18	142.02	178.04	7.48	14.51	3.87
Slc2a6	98.25	172.10	11.75	6.70	3.87
Sult4a1	46.69	48.32	5.34	1.12	3.87
Adamts12	299.60	178.04	18.16	14.51	3.87
Ms4a6c	188.71	210.25	7.48	20.10	3.86
Serpina7	55.45	72.06	2.14	6.70	3.86
Gbp2	1598.21	2171.19	101.46	160.76	3.85
BE692007	65.17	60.19	5.34	3.35	3.85
Itga11	37.94	55.95	3.20	3.35	3.84
Adam12	617.69	355.22	26.70	41.31	3.84
Col1a2	10623.29	9764.86	655.72	768.09	3.84
Sorcs1	97.27	121.23	6.41	8.93	3.84
Fut1	31.13	16.11	0.00	3.35	3.83
Mfap4	178.01	210.25	9.61	17.86	3.83
Parvg	249.99	286.55	23.49	14.51	3.82
Chil3	150.77	105.13	17.09	1.12	3.81
Phf11a	27.24	18.65	1.07	2.23	3.80
Cldn23	43.77	33.06	0.00	5.58	3.80
Ncf4	157.58	147.52	7.48	14.51	3.80
Arl11	46.69	59.35	4.27	3.35	3.80
Col15a1	2253.84	1758.32	164.46	123.92	3.80
Itk	88.52	93.26	6.41	6.70	3.79
Clcf1	28.21	77.15	4.27	3.35	3.79
Cd68	483.45	468.83	30.97	37.96	3.79
Tmem154	77.82	88.17	4.27	7.81	3.79
Pcdh8	88.52	91.56	8.54	4.47	3.78
-	27.24	2.54	2.14	0.00	3.78
Il31ra	16.54	13.56	0.00	2.23	3.77
Cd2	33.07	41.54	2.14	3.35	3.77
Cd3d	43.77	89.02	6.41	3.35	3.76
Dock10	1021.38	871.53	73.69	65.87	3.76
Gm4841	28.21	16.11	1.07	2.23	3.75
Col11a1	17.51	11.87	1.07	1.12	3.75
F730043M19Rik	44.75	44.09	0.00	6.70	3.75
2610203C20Rik	89.49	70.37	9.61	2.23	3.74
Runx1	345.32	368.79	24.56	29.03	3.74
Pla2g2e	35.99	22.04	3.20	1.12	3.74
Mfap5	57.39	89.02	1.07	10.05	3.74
Nkd2	99.22	103.43	7.48	7.81	3.73

Trbc1	53.50	77.15	2.14	7.81	3.73
Rtn4rl2	125.48	120.39	6.41	12.28	3.72
Tlr13	201.36	186.51	20.29	8.93	3.72
Clca3a2	113.81	59.35	4.27	8.93	3.72
Dpysl3	1643.93	1717.63	103.59	155.18	3.70
Ms4a6b	407.58	473.07	28.83	39.07	3.70
Podnl1	47.66	37.30	2.14	4.47	3.69
Spp1	331830.75	344945.91	26419.97	25959.98	3.69
Lyve1	12.65	15.26	2.14	0.00	3.69
Cd53	296.69	310.29	12.82	34.61	3.69
C5ar2	27.24	28.82	2.14	2.23	3.68
Spic	27.24	15.26	0.00	3.35	3.68
Relt	69.06	69.52	7.48	3.35	3.67
Mmp2	446.49	481.55	34.17	39.07	3.66
Mrc1	812.24	626.52	61.94	51.36	3.66
Olfr1034	7.78	19.50	2.14	0.00	3.66
Cd6	75.87	103.43	4.27	10.05	3.66
C3ar1	410.50	412.87	28.83	36.84	3.65
Dock2	301.55	301.81	21.36	26.79	3.65
Igsf10	83.66	66.13	8.54	3.35	3.65
Pik3ap1	278.20	200.93	16.02	22.33	3.64
Gm16184	35.02	33.06	3.20	2.23	3.64
Tlr9	98.25	79.69	4.27	10.05	3.64
Abcg3	58.36	50.87	3.20	5.58	3.64
Oas3	141.05	130.56	9.61	12.28	3.64
Spink12	11.67	15.26	1.07	1.12	3.63
Cxcr3	76.85	98.34	6.41	7.81	3.63
Hp	82.68	92.41	6.41	7.81	3.62
Pik3r5	165.37	170.41	12.82	14.51	3.62
Mgp	7445.35	9571.56	514.75	876.39	3.61
Vcan	129.37	56.80	10.68	4.47	3.61
Gxylt2	185.79	228.90	8.54	25.68	3.61
Pdgfrl	54.47	25.43	3.20	3.35	3.61
Dnm3os	92.41	133.95	5.34	13.40	3.60
AI504432	34.05	44.93	5.34	1.12	3.60
Cd52	440.65	525.63	33.11	46.89	3.60
Cd14	1290.83	1422.59	130.29	93.78	3.60
Gimap7	31.13	34.76	3.20	2.23	3.60
Tmem45a	137.16	140.73	6.41	16.75	3.59
Gm15931	13.62	12.72	1.07	1.12	3.59
Tbx21	29.18	49.17	3.20	3.35	3.58
Frem1	344.35	295.88	20.29	33.49	3.58
Clec4b1	26.26	26.28	0.00	4.47	3.57
Cp	8760.50	8739.88	580.96	888.67	3.57
Tmem173	580.73	600.24	43.79	55.82	3.57
Prrg4	942.58	876.62	46.99	107.18	3.56
Btbd17	13.62	11.87	2.14	0.00	3.56
Slc34a2	1300.55	851.18	103.59	79.27	3.55
BC147527	52.53	62.74	4.27	5.58	3.55
AI115009	15.56	22.89	1.07	2.23	3.55
Psd4	292.79	331.49	35.24	17.86	3.55
Tmem145	86.57	92.41	7.48	7.81	3.55
Gm16144	8.75	16.96	0.00	2.23	3.55
Prtn3	30.15	20.35	4.27	0.00	3.54
Adgre1	693.56	540.04	48.06	58.05	3.54
Gm38244	12.65	12.72	1.07	1.12	3.54
Nat8l	11.67	13.56	1.07	1.12	3.53
Coro1a	1576.81	1955.01	166.60	139.55	3.53
Fn1	5768.35	4947.71	404.75	525.83	3.53
St8sia2	17.51	7.63	1.07	1.12	3.52
Clu	58539.49	71716.36	5774.40	5621.14	3.51
Lrmp	162.45	174.64	11.75	17.86	3.51

Akr1b8	465.94	517.15	46.99	39.07	3.51
Bcl3	1494.13	1307.29	128.15	117.22	3.51
Ccr5	418.28	444.24	24.56	51.36	3.51
Col5a3	573.92	495.96	49.13	44.66	3.51
Mmp19	99.22	100.04	5.34	12.28	3.51
Gpr171	12.65	24.59	1.07	2.23	3.51
Sbk2	12.65	11.87	2.14	0.00	3.50
Col5a1	1609.88	1203.02	128.15	120.57	3.50
Ptpn7	127.43	133.10	5.34	17.86	3.50
Ifi207	386.18	294.18	23.49	36.84	3.50
Plac8	1966.88	1588.76	176.21	138.44	3.50
Fosl1	118.67	198.38	22.43	5.58	3.49
Itga2	101.16	33.91	7.48	4.47	3.49
Olfr56	22.37	14.41	2.14	1.12	3.49
Vtcn1	294.74	277.23	26.70	24.56	3.48
P2ry10	16.54	7.63	2.14	0.00	3.48
Ly86	281.12	301.81	23.49	29.03	3.47
Fndc1	87.55	58.50	4.27	8.93	3.47
Hmcn1	110.89	95.80	5.34	13.40	3.47
Sla	302.52	300.97	32.04	22.33	3.47
Pdlim4	122.57	106.82	10.68	10.05	3.47
Gm5547	14.59	9.33	2.14	0.00	3.46
Fbn2	71.01	121.23	9.61	7.81	3.46
Was	177.04	172.95	12.82	18.98	3.46
C1qb	2466.87	2542.53	215.73	238.91	3.46
Glipr1	66.15	78.84	3.20	10.05	3.46
Tmsb10	2438.66	2720.56	246.70	222.17	3.46
Gm39323	15.56	8.48	1.07	1.12	3.46
Lax1	55.45	63.58	8.54	2.23	3.46
Rac2	602.13	651.95	64.08	50.24	3.45
1810011H11Rik	117.70	85.63	7.48	11.16	3.45
Casp4	183.85	149.21	16.02	14.51	3.45
Cdca7	96.30	94.10	7.48	10.05	3.44
Lair1	190.66	165.32	19.22	13.40	3.44
D630011A20Rik	19.45	16.11	2.14	1.12	3.44
Adra1b	103.11	172.10	4.27	21.21	3.44
Itgax	184.82	232.29	9.61	29.03	3.44
Actg2	31.13	16.11	3.20	1.12	3.44
Gzmk	23.35	48.32	0.00	6.70	3.44
F630028O10Rik	54.47	50.87	7.48	2.23	3.43
Prrx2	44.75	61.04	4.27	5.58	3.43
Gm19719	23.35	11.87	2.14	1.12	3.43
C1qc	1827.78	1834.62	173.01	167.46	3.43
Itgb2	522.36	568.02	51.26	50.24	3.43
Mmp9	138.13	94.95	13.88	7.81	3.42
Lox	725.66	1037.70	44.85	120.57	3.41
Lum	75.87	52.56	3.20	8.93	3.41
Rgs1	14.59	32.22	0.00	4.47	3.41
Arl5c	18.48	39.00	4.27	1.12	3.41
Gm1966	67.12	83.08	4.27	10.05	3.40
Osmr	1700.35	1325.95	95.05	192.02	3.40
Ifi204	445.51	384.90	30.97	48.01	3.40
Slc11a1	355.05	280.62	19.22	41.31	3.40
Ubash3a	20.43	37.30	1.07	4.47	3.40
Adamts17	13.62	9.33	1.07	1.12	3.39
Slc9a9	93.38	78.00	10.68	5.58	3.39
Hmga2	16.54	17.80	1.07	2.23	3.39
Xkr5	17.51	16.96	0.00	3.35	3.38
Nlrp3	74.90	96.65	3.20	13.40	3.38
Itgal	318.09	433.22	26.70	45.77	3.38
Tac4	16.54	17.80	0.00	3.35	3.38
Atp1a3	53.50	59.35	7.48	3.35	3.38

Alx1	147.86	156.84	10.68	18.98	3.37
Lilr4b	253.89	118.69	14.95	21.21	3.37
-	45.72	55.11	6.41	3.35	3.36
Fblim1	1470.78	1173.34	118.54	138.44	3.36
Cmtm3	1203.28	1223.36	107.86	128.39	3.36
Arhgap30	470.81	507.83	35.24	60.29	3.36
S100a6	1498.02	1272.53	154.85	114.99	3.36
Igkv6-15	10.70	11.87	0.00	2.23	3.36
Klra2	52.53	59.35	3.20	7.81	3.35
Il11	21.40	33.91	3.20	2.23	3.35
Gm5431	31.13	46.63	2.14	5.58	3.34
BC051142	9.73	23.74	0.00	3.35	3.34
Ltb	84.63	137.34	5.34	16.75	3.34
Sash3	169.26	227.21	20.29	18.98	3.34
Col8a2	33.07	44.09	3.20	4.47	3.33
Gm3788	118.67	100.89	13.88	7.81	3.33
Gm16617	16.54	16.11	3.20	0.00	3.33
Galnt17	58.36	74.61	1.07	12.28	3.33
Runx3	94.36	100.89	16.02	3.35	3.32
Cygb	772.36	967.33	70.48	103.83	3.32
Aox3	22.37	21.19	2.14	2.23	3.32
Loxl3	414.39	421.35	51.26	32.38	3.32
AC107711.3	17.51	15.26	1.07	2.23	3.32
Fcgr3	584.62	597.69	34.17	84.85	3.32
Scara3	72.96	90.71	5.34	11.16	3.32
Nav3	98.25	65.28	7.48	8.93	3.32
Prr11	115.76	124.63	6.41	17.86	3.32
Edn1	366.72	407.79	25.63	52.47	3.31
Rasl10a	47.66	83.08	3.20	10.05	3.31
Ms4a4b	126.46	156.84	7.48	21.21	3.31
Cd300ld	101.16	50.87	6.41	8.93	3.31
Bend6	19.45	12.72	3.20	0.00	3.31
Zbp1	244.16	263.66	26.70	24.56	3.31
Gm48898	38.91	36.46	5.34	2.23	3.31
Il27ra	97.27	122.08	2.14	20.10	3.31
Cldn11	16.54	5.09	1.07	1.12	3.30
Prkcb	122.57	104.28	8.54	14.51	3.30
Tgfbi	2123.49	1966.88	184.76	231.10	3.30
Clec5a	55.45	73.76	4.27	8.93	3.30
-	60.31	45.78	8.54	2.23	3.29
Mgl2	101.16	112.76	6.41	15.63	3.29
Myo1f	452.32	473.07	50.19	44.66	3.29
Myo1g	314.19	389.14	29.90	42.42	3.28
Mdga1	35.02	50.02	4.27	4.47	3.28
Tyrobp	516.52	558.69	49.13	61.40	3.28
Ly6c2	130.35	156.84	12.82	16.75	3.28
Basp1	192.60	167.01	24.56	12.28	3.28
Clec4a3	112.84	110.21	9.61	13.40	3.28
Cd3g	64.20	83.93	6.41	8.93	3.28
Wisp1	208.17	160.23	23.49	14.51	3.27
Shc2	56.42	27.98	4.27	4.47	3.27
Gvin1	121.59	67.82	10.68	8.93	3.27
Cx3cr1	813.21	952.07	82.23	101.59	3.27
Cd209a	14.59	16.96	1.07	2.23	3.26
Gm26510	10.70	10.17	1.07	1.12	3.26
Gm9844	71.01	54.26	6.41	6.70	3.26
Zap70	79.76	66.13	8.54	6.70	3.26
Fbn1	1648.79	1544.68	121.75	213.24	3.26
Gdnf	18.48	22.89	3.20	1.12	3.25
Dusp8	673.14	863.05	86.50	74.80	3.25
Fcer1g	505.82	649.41	51.26	70.33	3.25
Cpxm1	351.16	447.63	32.04	52.47	3.24

Alox5ap	213.03	222.97	16.02	30.14	3.24
Pcdh9	16.54	14.41	2.14	1.12	3.24
Vav1	266.53	272.14	22.43	34.61	3.24
Inhba	88.52	25.43	5.34	6.70	3.24
Egr1	886.17	932.57	105.73	87.08	3.24
Nckap1l	665.35	640.93	70.48	68.10	3.24
Fam26f	30.15	21.19	3.20	2.23	3.23
Sct	12.65	17.80	3.20	0.00	3.23
Tnfaip8l2	127.43	149.21	12.82	16.75	3.23
Batf	38.91	32.22	5.34	2.23	3.22
Aim2	50.58	51.72	3.20	7.81	3.22
Plxnc1	73.93	68.67	5.34	10.05	3.22
Rhou	681.89	1010.57	82.23	100.48	3.21
Agap2	190.66	283.16	19.22	32.38	3.20
Apobr	172.17	150.06	16.02	18.98	3.20
Mpeg1	1795.68	1774.43	145.24	243.38	3.20
Themis2	280.15	289.94	35.24	26.79	3.20
Ch25h	131.32	84.78	22.43	1.12	3.20
Pilrb2	32.10	27.98	2.14	4.47	3.19
Aldh1a3	147.86	30.52	13.88	5.58	3.19
Apbb1ip	165.37	193.30	14.95	24.56	3.19
Tnc	612.83	409.48	58.74	53.59	3.18
Ctss	1778.17	2124.56	194.37	235.56	3.18
Col6a2	1242.19	1264.90	143.11	132.85	3.18
Dio2	13.62	16.11	1.07	2.23	3.18
Adgre4	32.10	17.80	0.00	5.58	3.17
Hr	295.71	474.76	29.90	55.82	3.17
Gm33858	8.75	20.35	3.20	0.00	3.17
Srpx	101.16	105.97	6.41	16.75	3.17
Psmd10	28.21	118.69	7.48	8.93	3.17
Rnf223	13.62	16.11	0.00	3.35	3.17
Lilrb4	246.10	127.17	18.16	23.44	3.17
Gm3636	11.67	17.80	0.00	3.35	3.16
Mb21d1	55.45	71.21	4.27	10.05	3.15
Slc28a2	71.98	55.11	3.20	11.16	3.15
Kcnh1	166.34	164.47	11.75	25.68	3.15
Ciita	486.37	450.18	50.19	55.82	3.14
Rab7b	188.71	122.08	8.54	26.79	3.14
Gm4070	113.81	70.37	5.34	15.63	3.14
Selplg	357.00	410.33	52.33	34.61	3.14
Adamts1	7732.31	7814.94	854.36	910.99	3.14
Clec10a	65.17	50.87	2.14	11.16	3.13
Fosb	39.88	121.23	14.95	3.35	3.13
Ms4a14	15.56	22.89	1.07	3.35	3.13
Cd72	286.96	275.53	39.51	24.56	3.13
Flnc	226.65	201.77	24.56	24.56	3.12
Cd48	142.02	133.95	16.02	15.63	3.12
Abcc3	455.24	372.18	40.58	54.70	3.12
Mdfi	75.87	103.43	9.61	11.16	3.11
Dcdc2a	1650.74	1520.09	150.58	216.58	3.11
Iglon5	34.05	69.52	5.34	6.70	3.11
Hdac9	66.15	65.28	8.54	6.70	3.11
Cd8b1	49.61	72.91	4.27	10.05	3.11
Slitrk4	28.21	46.63	5.34	3.35	3.11
Pik3cd	571.00	574.80	59.81	73.68	3.10
Nkg7	83.66	113.60	5.34	17.86	3.10
C1qa	2017.46	2282.25	221.07	282.45	3.10
Fgr	175.09	162.78	9.61	30.14	3.09
Fam26e	49.61	71.21	6.41	7.81	3.09
Il1r2	26.26	11.02	2.14	2.23	3.09
Pik3cg	135.21	180.58	16.02	21.21	3.09
Itgb7	184.82	249.25	20.29	31.26	3.08

Dusp10	81.71	92.41	12.82	7.81	3.07
Bmper	20.43	16.11	3.20	1.12	3.07
Cst7	15.56	11.87	2.14	1.12	3.07
Adgrg2	173.15	267.90	14.95	37.96	3.07
Adamts4	394.93	319.62	29.90	55.82	3.06
Sema4f	6.81	20.35	2.14	1.12	3.06
Ros1	40.86	13.56	4.27	2.23	3.06
Ptafr	86.57	77.15	7.48	12.28	3.05
Oit1	68.09	84.78	14.95	3.35	3.05
Naip1	41.83	22.04	2.14	5.58	3.05
Cxcr6	52.53	94.95	1.07	16.75	3.05
Fut7	5.84	21.19	1.07	2.23	3.04
Cd248	600.18	426.44	65.14	59.17	3.04
Laptm5	1584.59	1832.08	179.42	235.56	3.04
Gm12250	58.36	77.15	4.27	12.28	3.04
Nfam1	221.78	355.22	25.63	44.66	3.04
Bcl2a1b	24.32	20.35	4.27	1.12	3.04
Hck	336.57	266.21	32.04	41.31	3.04
Snai1	151.75	252.64	19.22	30.14	3.04
Tnfrsf1b	619.64	623.13	41.65	110.52	3.03
Krt19	613.80	330.64	65.14	50.24	3.03
Mx1	110.89	155.99	13.88	18.98	3.03
Il34	2105.01	2440.79	262.72	295.85	3.03
Hsh2d	11.67	15.26	0.00	3.35	3.02
Pstpip1	172.17	255.19	19.22	33.49	3.02
D430020J02Rik	59.34	39.00	3.20	8.93	3.02
Traf3ip3	92.41	110.21	13.88	11.16	3.02
Gpr18	15.56	11.02	1.07	2.23	3.01
Gbgt1	38.91	31.37	5.34	3.35	3.01
Cyth4	994.14	1005.48	112.13	136.20	3.01
Ly9	105.06	89.02	9.61	14.51	3.01
Masp1	148.83	184.82	17.09	24.56	3.01
Gapt	9.73	25.43	1.07	3.35	3.01
Kcnn4	71.01	95.80	6.41	14.51	3.00
Gm11427	13.62	12.72	1.07	2.23	3.00
Cntn1	75.87	46.63	5.34	10.05	3.00
Plaur	517.50	517.15	67.28	62.52	2.99
Lbp	624.50	717.23	68.35	100.48	2.99
Slc25a24	729.55	639.23	88.64	83.73	2.99
Olfml2b	146.88	87.32	14.95	14.51	2.99
Evi2a	107.00	117.00	14.95	13.40	2.98
Pou2f2	116.73	133.95	10.68	21.21	2.98
Gbp3	1462.03	1420.05	153.78	212.12	2.98
Bin2	214.00	200.08	20.29	32.38	2.98
Sidt1	31.13	20.35	4.27	2.23	2.98
Inhbb	575.86	395.92	52.33	71.45	2.97
S1pr2	422.17	427.29	50.19	58.05	2.97
Igsf23	16.54	9.33	1.07	2.23	2.97
Ifi27l2a	1114.76	952.92	138.83	125.04	2.97
Ccdc184	9.73	16.11	0.00	3.35	2.97
Cfp	619.64	708.75	81.16	89.31	2.96
Xlr4b	8.75	16.96	0.00	3.35	2.96
Il21r	141.05	105.97	11.75	20.10	2.96
Raet1e	23.35	19.50	0.00	5.58	2.95
Itih4	13.62	20.35	1.07	3.35	2.95
Syt8	54.47	29.67	7.48	3.35	2.95
Mmp12	41.83	25.43	5.34	3.35	2.95
Nfkbiz	1633.23	1581.13	191.16	226.63	2.94
Lhfp	1078.77	1034.31	111.07	164.11	2.94
Il1rl2	155.64	113.60	14.95	20.10	2.94
Nrros	379.37	368.79	41.65	55.82	2.94
Grap2	99.22	128.02	9.61	20.10	2.94

Gm17334	11.67	13.56	1.07	2.23	2.94
Birc3	1308.34	1192.84	152.72	173.04	2.94
C1qtnf6	438.71	428.13	76.89	35.73	2.94
Cyp1b1	1658.52	1748.99	127.09	317.06	2.94
Lck	148.83	229.75	14.95	34.61	2.94
Ttc9	46.69	37.30	4.27	6.70	2.94
Adcy7	638.12	726.56	76.89	101.59	2.94
Meis3	121.59	161.08	23.49	13.40	2.93
Cd96	10.70	14.41	1.07	2.23	2.93
Fkbp10	418.28	413.72	66.21	42.42	2.93
Cpe	5233.34	6304.18	586.30	924.39	2.93
Spaca7	30.15	44.09	8.54	1.12	2.93
Cxcl9	24.32	67.82	1.07	11.16	2.93
Cysltr1	46.69	69.52	6.41	8.93	2.93
Casp12	114.78	117.00	14.95	15.63	2.92
Gucy1a2	21.40	19.50	5.34	0.00	2.92
Gm9780	23.35	26.28	3.20	3.35	2.92
Ptprc	939.67	891.88	123.88	118.34	2.92
Mlkl	186.77	168.71	20.29	26.79	2.92
Gfi1	13.62	19.50	1.07	3.35	2.92
Pla2g12b	36.96	37.30	4.27	5.58	2.92
Art4	280.15	287.40	39.51	35.73	2.91
Il18rap	54.47	51.72	9.61	4.47	2.91
Panx1	156.61	178.88	24.56	20.10	2.91
Lst1	116.73	72.06	11.75	13.40	2.91
Loxl1	1278.18	1334.42	139.90	208.77	2.91
Snx20	104.08	157.69	16.02	18.98	2.91
Ighg2c	101.16	95.80	9.61	16.75	2.91
Klrd1	23.35	33.91	3.20	4.47	2.90
1500009L16Rik	59.34	55.11	7.48	7.81	2.90
Mnda	137.16	116.15	13.88	20.10	2.90
Aif1	170.23	146.67	25.63	16.75	2.90
Gria4	62.26	59.35	9.61	6.70	2.90
Milr1	76.85	93.26	10.68	12.28	2.89
Samd5	340.46	328.10	54.47	35.73	2.89
Xlr4a	9.73	22.89	0.00	4.47	2.89
Myc	1019.43	1140.28	118.54	174.16	2.89
Nrip3	33.07	22.89	6.41	1.12	2.88
Junb	2595.27	2835.01	365.24	371.77	2.88
Ctnnd2	15.56	32.22	5.34	1.12	2.88
Gpr55	20.43	19.50	4.27	1.12	2.88
Gbp5	455.24	326.40	34.17	72.57	2.88
Card11	69.06	74.61	11.75	7.81	2.87
Ifitm10	163.42	75.45	20.29	12.28	2.87
Ikzf1	250.97	243.32	27.77	40.19	2.87
Slpi	13.62	10.17	2.14	1.12	2.86
Tifab	264.59	314.53	35.24	44.66	2.86
Scube1	107.97	122.08	13.88	17.86	2.86
Plxna4os1	15.56	23.74	4.27	1.12	2.86
Plcb2	154.67	155.15	16.02	26.79	2.86
Casp1	71.01	88.17	5.34	16.75	2.86
Rasal3	213.03	263.66	20.29	45.77	2.86
Tnfsf8	28.21	43.24	2.14	7.81	2.85
Gpr153	274.31	303.51	30.97	49.12	2.85
Klf6	3879.29	4641.66	566.01	612.91	2.85
Ccdc122	51.56	58.50	9.61	5.58	2.85
Gm2237	22.37	16.96	3.20	2.23	2.85
Pdgfb	3517.43	4931.60	599.12	573.84	2.85
Ncf1	295.71	323.01	52.33	33.49	2.85
Pilrb1	21.40	17.80	3.20	2.23	2.85
Pid1	195.52	251.79	29.90	32.38	2.85
Hgf	83.66	50.87	3.20	15.63	2.84

Anxa3	1460.08	1363.25	202.91	190.91	2.84
Relb	989.28	967.33	120.68	152.95	2.84
Tgtp1	363.80	254.34	33.11	53.59	2.84
Srpx2	318.09	267.05	37.38	44.66	2.83
Efemp2	881.30	920.70	106.79	146.25	2.83
Adora2b	317.11	522.24	60.87	56.94	2.83
Spn	222.76	243.32	34.17	31.26	2.83
Tgfb1i1	719.83	643.47	101.46	90.43	2.83
Arid5a	449.41	573.11	69.42	74.80	2.83
Mrc2	2147.81	2624.76	318.25	355.02	2.83
Naip6	41.83	35.61	5.34	5.58	2.83
Lacc1	295.71	261.97	32.04	46.89	2.82
Icam1	2673.09	2676.48	330.00	426.47	2.82
Wfdc17	77.82	45.78	9.61	7.81	2.82
Akap12	1707.16	1554.85	264.85	196.49	2.82
Loxl2	671.19	716.38	96.12	100.48	2.82
Piezo2	228.59	260.27	40.58	29.03	2.81
Pcdhgc5	23.35	22.89	1.07	5.58	2.81
Arhgap27os3	25.29	35.61	5.34	3.35	2.81
Cybb	708.15	802.86	112.13	103.83	2.81
S100a14	129.37	91.56	19.22	12.28	2.81
Cldn4	3373.46	3270.78	527.57	423.12	2.80
Col6a1	1869.61	1776.12	227.47	296.97	2.80
Synpo2	322.95	316.23	38.45	53.59	2.80
A530040E14Rik	15.56	14.41	4.27	0.00	2.80
Slc15a3	244.16	205.17	21.36	43.54	2.80
Gna15	67.12	77.15	7.48	13.40	2.79
Oas2	633.25	518.85	77.96	88.20	2.79
Sfn	1227.60	1305.60	193.30	171.93	2.79
AC113595.1	41.83	33.91	5.34	5.58	2.79
Wdfy4	221.78	247.56	30.97	36.84	2.79
Pot1b	20.43	10.17	0.00	4.47	2.79
Plek	343.38	250.10	42.72	43.54	2.78
Fyb	303.49	239.08	25.63	53.59	2.78
Tnfrsf23	113.81	133.10	19.22	16.75	2.78
BC035044	19.45	10.17	4.27	0.00	2.78
Gng8	29.18	53.41	3.20	8.93	2.78
Mag	13.62	16.11	3.20	1.12	2.78
Prr7	33.07	33.91	6.41	3.35	2.77
Tlr1	131.32	145.82	14.95	25.68	2.77
Slfn9	136.18	93.26	25.63	7.81	2.77
Dse	401.74	344.20	42.72	66.98	2.77
Samsn1	39.88	50.02	2.14	11.16	2.77
Gm17501	85.60	55.95	8.54	12.28	2.77
Il2rb	89.49	111.06	12.82	16.75	2.77
Ticrr	54.47	49.17	8.54	6.70	2.76
Dpt	181.90	218.73	25.63	33.49	2.76
Epb42	157.58	100.89	24.56	13.40	2.76
Lat	97.27	153.45	17.09	20.10	2.76
Syk	329.76	335.73	36.31	62.52	2.75
Cd83	169.26	171.25	13.88	36.84	2.75
Lrriq1	17.51	11.87	2.14	2.23	2.75
Sla2	13.62	22.89	3.20	2.23	2.75
Fam109b	33.07	32.22	7.48	2.23	2.74
Smpd3	30.15	42.39	7.48	3.35	2.74
Col12a1	1218.84	1154.69	169.80	186.44	2.74
Cd4	78.79	95.80	11.75	14.51	2.74
Kif21b	276.26	371.33	39.51	58.05	2.73
AB124611	110.89	106.82	14.95	17.86	2.73
Arsi	35.02	30.52	3.20	6.70	2.73
Pld4	18.48	17.80	2.14	3.35	2.73
Lpcat2	119.65	126.32	12.82	24.56	2.72

Cd247	49.61	65.28	9.61	7.81	2.72
Rad51b	27.24	22.89	5.34	2.23	2.72
Ngf	181.90	250.95	22.43	43.54	2.72
Ssc5d	97.27	124.63	19.22	14.51	2.72
Tnf	19.45	23.74	2.14	4.47	2.72
Eno2	158.56	242.47	30.97	30.14	2.72
Smoc2	516.52	478.15	68.35	83.73	2.71
Mcub	86.57	70.37	13.88	10.05	2.71
Nt5c1a	84.63	58.50	9.61	12.28	2.71
9330102E08Rik	10.70	17.80	2.14	2.23	2.71
Gm13889	682.86	590.06	81.16	113.87	2.71
Gm19951	19.45	16.11	3.20	2.23	2.71
Hcls1	589.48	551.91	75.82	99.36	2.71
Cav3	17.51	11.02	2.14	2.23	2.71
Lcp2	181.90	187.36	27.77	29.03	2.70
Tnfrsf12a	2243.14	2765.50	357.76	413.07	2.70
Snx10	620.61	612.11	92.91	97.13	2.70
Bdkrb2	172.17	182.28	24.56	30.14	2.70
Fgg	4978.48	6108.34	459.22	1251.50	2.70
Lsp1	488.32	521.39	81.16	74.80	2.69
D630003M21Rik	280.15	350.14	32.04	65.87	2.69
Marcks	1951.32	1861.75	250.97	340.51	2.69
Nkd1	330.73	272.99	50.19	43.54	2.69
Aplnr	336.57	296.73	39.51	59.17	2.68
Ccdc88b	308.36	249.25	49.13	37.96	2.68
Fanci	71.98	54.26	6.41	13.40	2.68
Car13	256.80	310.29	26.70	62.52	2.67
Tlr6	40.86	35.61	6.41	5.58	2.67
Nlrc5	557.38	501.89	61.94	104.94	2.67
Mmp14	1577.78	1294.58	183.69	269.06	2.67
Doc2a	18.48	16.11	3.20	2.23	2.67
Ms4a7	133.27	110.21	11.75	26.79	2.66
Ttk	63.23	53.41	14.95	3.35	2.66
Ticam2	23.35	18.65	0.00	6.70	2.66
Nfkbid	148.83	160.23	32.04	16.75	2.66
Igkv1-117	6.81	27.98	0.00	5.58	2.66
Cebpd	882.28	656.19	138.83	104.94	2.66
AC166344.1	43.77	18.65	2.14	7.81	2.65
Ccl6	283.07	272.99	40.58	48.01	2.65
Mir142hg	20.43	40.69	5.34	4.47	2.64
Pcdh18	312.25	241.62	34.17	54.70	2.64
Hpx	13.62	13.56	2.14	2.23	2.64
Gm34397	13.62	20.35	2.14	3.35	2.64
Emilin2	213.03	206.01	42.72	24.56	2.64
Zfp831	11.67	22.04	3.20	2.23	2.63
Hcst	42.80	38.15	8.54	4.47	2.63
Oas1g	183.85	209.40	24.56	39.07	2.63
Trac	90.46	137.34	27.77	8.93	2.63
H2-Ab1	5056.30	6009.99	751.84	1037.15	2.63
Sh2d5	21.40	25.43	6.41	1.12	2.63
Ifi209	149.80	183.12	12.82	41.31	2.63
Klrg2	261.67	307.75	28.83	63.64	2.63
Ighv1-26	17.51	16.11	3.20	2.23	2.63
Mtfr2	39.88	33.91	7.48	4.47	2.62
-	35.02	51.72	10.68	3.35	2.62
Lhfpl2	741.23	1103.82	150.58	149.60	2.62
Bmp1	1310.28	1347.99	212.52	221.05	2.62
Trpv2	214.98	222.12	22.43	49.12	2.62
Hpgds	150.77	150.06	25.63	23.44	2.62
Gata6	80.74	117.84	24.56	7.81	2.61
Vstm4	544.73	453.57	52.33	111.64	2.61
Il10ra	312.25	351.83	27.77	81.50	2.61

Cldn1	1719.80	1545.52	246.70	289.15	2.61
Chsy3	31.13	15.26	5.34	2.23	2.61
Gm26674	22.37	11.02	2.14	3.35	2.61
Sh2b2	123.54	101.73	20.29	16.75	2.60
Cd74	10454.04	13267.09	1621.15	2297.58	2.60
Ripk3	157.58	140.73	19.22	30.14	2.60
Soat2	36.96	28.82	7.48	3.35	2.60
Igsf6	111.87	132.26	17.09	23.44	2.59
Rnase4	540.84	579.89	83.30	102.71	2.59
Ppp1r36	29.18	29.67	7.48	2.23	2.59
-	55.45	102.58	9.61	16.75	2.59
Tnfaip3	625.47	842.70	102.52	141.78	2.59
Emilin1	3161.40	2827.38	517.95	477.83	2.59
Vgll3	194.55	180.58	22.43	40.19	2.59
Gm38158	10.70	22.04	2.14	3.35	2.58
Gm16174	35.02	24.59	1.07	8.93	2.58
Oasl2	1087.52	1048.72	165.53	190.91	2.58
Dhx58	253.89	260.27	56.60	29.03	2.58
Gm15895	222.76	189.06	35.24	33.49	2.58
Gpr35	84.63	66.13	8.54	16.75	2.58
Scn7a	131.32	175.49	18.16	33.49	2.58
Ptchd1	33.07	18.65	6.41	2.23	2.57
Rhoh	41.83	36.46	4.27	8.93	2.57
Radil	19.45	19.50	3.20	3.35	2.57
Maff	510.69	415.42	90.78	64.75	2.57
Clec18a	164.39	171.25	37.38	18.98	2.57
Gpbar1	13.62	18.65	3.20	2.23	2.57
Gimap3	225.68	330.64	44.85	49.12	2.57
-	26.26	32.22	2.14	7.81	2.56
Spi1	379.37	433.22	69.42	68.10	2.56
Pdgfrb	2831.65	3024.92	413.30	578.30	2.56
Mycl	140.07	254.34	22.43	44.66	2.56
Camk1d	242.21	174.64	42.72	27.91	2.56
F730311O21Rik	21.40	16.96	4.27	2.23	2.55
Acta2	4116.64	2356.86	584.17	518.02	2.55
Vstm2b	27.24	37.30	2.14	8.93	2.55
Gbp10	27.24	23.74	6.41	2.23	2.55
Foxs1	79.76	118.69	13.88	20.10	2.55
Ttc39a	92.41	81.39	7.48	22.33	2.55
Tlr7	114.78	109.37	16.02	22.33	2.55
Fkbp1b	48.64	78.84	9.61	12.28	2.55
Gucy1a1	1172.15	1005.48	123.88	250.08	2.54
Aunip	21.40	16.96	2.14	4.47	2.54
Gpc3	1805.41	1904.14	338.54	298.08	2.54
Oit3	24.32	33.06	3.20	6.70	2.54
Lat2	79.76	122.93	19.22	15.63	2.54
Ddr2	713.02	657.04	92.91	142.90	2.54
Cd244	25.29	32.22	2.14	7.81	2.54
Iigp1	553.49	571.41	55.53	138.44	2.54
Gjc2	51.56	43.24	10.68	5.58	2.54
Arhgap9	275.29	314.53	42.72	59.17	2.54
Fyb2	403.69	549.37	56.60	108.29	2.53
Clip3	144.94	140.73	17.09	32.38	2.53
9130208D14Rik	22.37	9.33	2.14	3.35	2.53
H2-DMa	513.61	536.65	98.25	83.73	2.53
Il18r1	93.38	64.43	11.75	15.63	2.53
Ercc6l	76.85	111.91	14.95	17.86	2.53
Lrg1	984.41	922.40	166.60	165.23	2.52
Naip5	82.68	99.19	14.95	16.75	2.52
Mfap2	203.30	172.95	32.04	33.49	2.52
Gbp6	172.17	211.95	20.29	46.89	2.52
Colec12	757.76	747.75	102.52	160.76	2.52

Gsdmd	408.55	446.79	76.89	72.57	2.52
Fgl2	262.64	239.08	29.90	58.05	2.52
Marcksl1	1106.01	954.61	176.21	184.21	2.52
Stab1	2916.27	2690.89	458.15	524.71	2.51
Gli3	61.28	25.43	11.75	3.35	2.51
Lyl1	142.99	168.71	21.36	33.49	2.51
Sytl2	2776.20	2314.47	498.73	395.21	2.51
Ptpn22	294.74	354.38	38.45	75.92	2.51
Ecscr	321.00	287.40	56.60	50.24	2.51
Tnfrsf14	86.57	87.32	13.88	16.75	2.51
Rerg	226.65	225.51	38.45	41.31	2.50
Gm8369	15.56	15.26	3.20	2.23	2.50
Cemip	109.92	87.32	20.29	14.51	2.50
Cd300a	98.25	80.54	17.09	14.51	2.50
Parp14	1846.26	1657.43	288.35	332.69	2.50
Eomes	15.56	33.91	2.14	6.70	2.50
Tnfsf14	37.94	23.74	5.34	5.58	2.50
Serpinf1	95.33	58.50	14.95	12.28	2.49
-	75.87	23.74	3.20	14.51	2.49
Itga4	232.48	308.60	43.79	52.47	2.49
Vim	4139.01	3754.87	759.31	643.05	2.49
Gpr183	36.96	55.11	7.48	8.93	2.49
Ifi206	93.38	108.52	21.36	14.51	2.49
Apobec1	506.80	574.80	81.16	111.64	2.49
Ccdc80	520.42	363.70	71.55	85.96	2.49
Pdlim1	260.69	228.90	37.38	50.24	2.48
Neil3	56.42	72.06	8.54	14.51	2.48
Nos2	20.43	22.04	5.34	2.23	2.48
Olfm2	73.93	84.78	12.82	15.63	2.48
Mical1	255.83	232.29	40.58	46.89	2.48
Prune2	76.85	51.72	8.54	14.51	2.48
Siglece	103.11	104.28	14.95	22.33	2.48
Nfkbie	479.56	539.20	73.69	109.41	2.48
Psmb8	800.56	866.44	132.43	167.46	2.48
C6	23.35	37.30	5.34	5.58	2.48
Isg20	108.95	166.17	11.75	37.96	2.48
Tmem119	408.55	623.97	81.16	104.94	2.47
Gm26669	67.12	66.98	7.48	16.75	2.47
Nlrp1b	26.26	22.04	5.34	3.35	2.47
Clec2i	28.21	26.28	4.27	5.58	2.47
Rhob	6696.34	7723.38	1317.85	1291.69	2.47
Dennd2a	251.94	195.84	33.11	48.01	2.47
Cd40	166.34	171.25	26.70	34.61	2.46
Catip	19.45	22.89	2.14	5.58	2.46
Tcf7	91.44	100.89	17.09	17.86	2.46
Abi3bp	177.04	143.28	16.02	42.42	2.46
Eps8l3	87.55	98.34	14.95	18.98	2.46
Gm16332	33.07	15.26	2.14	6.70	2.46
Trim30d	142.02	141.58	11.75	40.19	2.45
Fam111a	256.80	293.34	46.99	53.59	2.45
Gab3	22.37	31.37	4.27	5.58	2.45
Csf1	976.63	935.96	183.69	166.35	2.45
2200002D01Rik	521.39	510.37	117.47	71.45	2.45
Fam19a3	27.24	32.22	5.34	5.58	2.45
Peg10	34.05	37.30	5.34	7.81	2.44
Ctsk	233.46	276.38	42.72	51.36	2.44
Cnn2	1999.95	1411.57	326.79	303.66	2.44
Gm14005	28.21	24.59	7.48	2.23	2.44
Fam105a	236.38	325.55	39.51	64.75	2.43
Nbl1	789.86	891.03	180.48	130.62	2.43
Gng2	244.16	215.34	34.17	51.36	2.43
Dact3	292.79	411.18	66.21	64.75	2.43

Adam23	16.54	18.65	3.20	3.35	2.43
Exo1	40.86	29.67	5.34	7.81	2.42
Isg15	347.27	271.29	71.55	43.54	2.42
Fermt3	370.61	497.65	81.16	81.50	2.42
-	52.53	52.56	8.54	11.16	2.42
Bgn	8692.40	10686.41	1496.20	2134.58	2.42
Ctse	35.02	22.89	8.54	2.23	2.42
Gbp8	41.83	56.80	10.68	7.81	2.41
Aox1	66.15	44.09	12.82	7.81	2.41
Syn1	24.32	22.04	5.34	3.35	2.41
Sptbn5	75.87	51.72	12.82	11.16	2.41
Cd79b	54.47	44.09	8.54	10.05	2.41
Cdsn	40.86	39.85	8.54	6.70	2.40
Slamf9	183.85	202.62	36.31	36.84	2.40
Penk	69.06	46.63	9.61	12.28	2.40
Fcrlb	17.51	22.89	3.20	4.47	2.40
Kif14	74.90	93.26	7.48	24.56	2.40
Arhgdib	713.02	713.84	138.83	131.74	2.40
Bcl2a1d	20.43	19.50	6.41	1.12	2.40
Depdc1a	49.61	48.32	8.54	10.05	2.40
Sacs	89.49	77.15	13.88	17.86	2.39
Hspb8	539.87	555.30	68.35	140.67	2.39
Cd37	152.72	127.17	33.11	20.10	2.39
Islr	324.90	307.75	41.65	79.27	2.39
Gm14230	22.37	17.80	3.20	4.47	2.39
-	30.15	38.15	8.54	4.47	2.39
Rgs19	258.75	267.05	52.33	48.01	2.39
1110002J07Rik	33.07	34.76	11.75	1.12	2.39
Sparc	11675.80	11831.77	2156.19	2336.65	2.39
Ctsw	73.93	119.54	18.16	18.98	2.38
Trim72	14.59	36.46	5.34	4.47	2.38
Gzma	30.15	21.19	4.27	5.58	2.38
Mmp11	312.25	593.45	70.48	103.83	2.38
H2-Aa	2678.93	3436.95	468.83	706.69	2.38
Skap1	66.15	75.45	16.02	11.16	2.38
Fxyd5	722.75	778.27	166.60	121.69	2.38
Emp3	392.99	331.49	85.44	53.59	2.38
Gch1	159.53	187.36	26.70	40.19	2.38
Capg	1991.20	2148.30	444.27	352.79	2.38
Tgm7	94.36	116.15	10.68	30.14	2.37
Itih3	97.27	106.82	14.95	24.56	2.37
Tmem158	369.64	277.23	76.89	48.01	2.37
F2r	3602.06	4790.02	547.86	1079.57	2.37
Ang	77.82	74.61	11.75	17.86	2.37
Fscn1	594.34	504.44	93.98	119.46	2.36
Ebi3	27.24	34.76	4.27	7.81	2.36
Neurl3	579.75	552.76	90.78	129.50	2.36
Epsti1	203.30	257.73	37.38	52.47	2.36
Pdgfra	1492.18	1484.48	176.21	404.14	2.36
Tspan32	41.83	42.39	6.41	10.05	2.36
Abcd2	15.56	17.80	4.27	2.23	2.36
Ncf2	317.11	326.40	52.33	73.68	2.35
Tnfsf13b	102.14	130.56	32.04	13.40	2.35
Foxj1	91.44	87.32	14.95	20.10	2.35
5830408C22Rik	19.45	19.50	4.27	3.35	2.35
Adcyap1r1	109.92	61.89	24.56	8.93	2.35
Kcnk6	187.74	118.69	32.04	27.91	2.35
F2rl3	43.77	28.82	6.41	7.81	2.35
Gm26797	38.91	22.04	8.54	3.35	2.35
Sertad4	204.28	207.71	37.38	43.54	2.35
Clspn	120.62	73.76	21.36	16.75	2.35
Ptgs2	45.72	37.30	10.68	5.58	2.35

Gpr132	59.34	57.65	8.54	14.51	2.35
Lcp1	2066.10	1847.34	344.95	425.35	2.35
Gm43461	15.56	17.80	2.14	4.47	2.34
Rbp1	472.75	534.11	103.59	94.90	2.34
Rgs14	78.79	109.37	17.09	20.10	2.34
Sdc3	1848.21	1370.03	333.20	301.43	2.34
Galnt6	71.98	72.06	11.75	16.75	2.34
Cyr61	2606.94	1854.97	545.72	336.04	2.34
Trpv6	268.48	229.75	37.38	61.40	2.34
Msln	50.58	20.35	12.82	1.12	2.34
AW112010	144.94	218.73	32.04	40.19	2.33
Nipal1	77.82	54.26	14.95	11.16	2.33
Map7d3	24.32	25.43	3.20	6.70	2.33
Gpc2	25.29	18.65	5.34	3.35	2.33
Csf3r	230.54	192.45	48.06	35.73	2.33
Pkib	36.96	61.89	9.61	10.05	2.33
Fstl1	2136.14	1837.16	466.69	321.53	2.33
Sh2d1b1	44.75	53.41	12.82	6.70	2.33
-	43.77	44.09	6.41	11.16	2.33
Clic6	31.13	23.74	4.27	6.70	2.32
Fam167a	122.57	100.89	27.77	16.75	2.32
Svep1	736.36	885.94	120.68	204.30	2.32
Cxcl14	423.14	451.02	76.89	98.24	2.32
Spsb1	298.63	401.85	75.82	64.75	2.32
Rad54l	92.41	92.41	18.16	18.98	2.32
Adamtsl4	366.72	339.12	69.42	72.57	2.31
Kcnd1	43.77	59.35	8.54	12.28	2.31
Actn1	3311.21	2407.73	562.81	590.58	2.31
Rcn3	790.84	741.82	146.31	163.00	2.31
Pimreg	134.24	131.41	21.36	32.38	2.31
Trim34b	33.07	10.17	5.34	3.35	2.31
Flna	10635.94	7450.39	1902.02	1767.28	2.30
Gm19391	27.24	21.19	4.27	5.58	2.30
Tubb2b	1633.23	1720.17	362.03	320.41	2.30
Adora2a	245.13	210.25	48.06	44.66	2.30
Lsmem2	81.71	105.13	21.36	16.75	2.29
Dapp1	234.43	253.49	60.87	39.07	2.29
Ccr9	12.65	24.59	3.20	4.47	2.28
Cldn6	86.57	67.82	14.95	16.75	2.28
Klf5	121.59	89.87	29.90	13.40	2.28
AI662270	29.18	44.93	8.54	6.70	2.28
Sp110	181.90	210.25	42.72	37.96	2.28
Ednra	150.77	140.73	29.90	30.14	2.28
Ctxn1	256.80	319.62	57.67	61.40	2.28
Jaml	27.24	36.46	4.27	8.93	2.28
Nfe2l3	37.94	30.52	9.61	4.47	2.28
Lrp8	85.60	121.23	12.82	30.14	2.27
Axl	2103.06	2099.98	444.27	425.35	2.27
Prkg1	50.58	28.82	6.41	10.05	2.27
Micall2	319.06	254.34	67.28	51.36	2.27
B3gnt9	81.71	75.45	21.36	11.16	2.27
Cd300c2	140.07	153.45	35.24	25.68	2.27
Padi2	132.29	150.91	32.04	26.79	2.27
Rap2b	170.23	185.67	43.79	30.14	2.27
Fhl2	892.98	992.76	149.51	243.38	2.26
BC023105	33.07	61.04	10.68	8.93	2.26
Ncapg	72.96	125.47	24.56	16.75	2.26
Emp2	766.52	803.71	165.53	161.88	2.26
Jazf1	80.74	55.11	16.02	12.28	2.26
Tuba1a	1320.98	1169.95	262.72	257.89	2.26
Foxf1	16.54	20.35	1.07	6.70	2.26
Anxa1	797.65	558.69	143.11	140.67	2.26

Ms4a4d	34.05	23.74	3.20	8.93	2.26
Ddx60	119.65	99.19	23.49	22.33	2.25
Chac1	94.36	177.19	18.16	39.07	2.25
Procr	99.22	94.95	7.48	33.49	2.25
Pclaf	206.22	197.54	50.19	34.61	2.25
Dcn	1092.39	1135.19	143.11	325.99	2.25
Irf1	1148.81	1257.27	248.83	257.89	2.25
Rnase6	44.75	64.43	7.48	15.63	2.25
Pdlim7	1174.10	1215.73	254.17	250.08	2.24
Vsir	720.80	670.60	137.77	156.30	2.24
Aurkb	199.41	224.66	38.45	51.36	2.24
Rel	168.28	136.49	32.04	32.38	2.24
Sorcs2	46.69	45.78	12.82	6.70	2.24
Rtp4	547.65	515.46	108.93	116.11	2.24
C1ra	891.03	945.29	156.99	232.21	2.24
Pcdhb22	61.28	61.89	17.09	8.93	2.24
Adgrg5	19.45	42.39	5.34	7.81	2.24
Ppp1r18	721.77	673.15	123.88	171.93	2.24
Ptgir	131.32	143.28	17.09	41.31	2.24
Rftn2	60.31	73.76	11.75	16.75	2.24
Arhgef25	501.93	528.17	119.61	99.36	2.23
Adamtsl5	244.16	292.49	70.48	43.54	2.23
Cd226	13.62	22.04	5.34	2.23	2.23
Kcnmb1	73.93	60.19	8.54	20.10	2.23
-	3211.99	2892.66	778.53	525.83	2.23
Cd33	136.18	181.43	24.56	43.54	2.23
Pgf	27.24	18.65	5.34	4.47	2.22
Hpcal4	222.76	204.32	49.13	42.42	2.22
Fgd2	354.08	368.79	72.62	82.61	2.22
Ptch2	163.42	254.34	32.04	58.05	2.22
Trpm2	95.33	87.32	20.29	18.98	2.22
Anxa2	4265.46	3887.97	981.44	780.37	2.21
Il3ra	216.92	150.06	48.06	31.26	2.21
Atp8b4	91.44	80.54	12.82	24.56	2.21
Tgfb1	1034.02	1080.09	218.93	240.03	2.20
Sbno2	3718.78	4039.73	824.46	865.22	2.20
Stil	76.85	93.26	19.22	17.86	2.20
Cd8a	113.81	194.99	42.72	24.56	2.20
Akna	353.10	440.85	65.14	108.29	2.20
Mx2	90.46	125.47	16.02	31.26	2.20
Unc5a	106.03	113.60	23.49	24.56	2.19
A730049H05Rik	118.67	106.82	16.02	33.49	2.19
Tmem229b	196.49	182.28	35.24	48.01	2.19
Tgfb3	131.32	167.01	29.90	35.73	2.19
Pltp	323.92	306.05	74.76	63.64	2.19
Arhgap25	112.84	110.21	26.70	22.33	2.18
Lrp1	3642.91	3456.44	614.07	950.07	2.18
Slc16a3	120.62	87.32	25.63	20.10	2.18
Il33	469.83	371.33	102.52	82.61	2.18
Fcgr4	114.78	108.52	21.36	27.91	2.18
St6galnac5	59.34	39.85	10.68	11.16	2.18
Fosl2	2176.02	2105.91	388.73	555.97	2.18
Trim30a	467.89	434.92	87.57	111.64	2.18
Prph	12.65	27.13	2.14	6.70	2.18
Plekho2	480.53	470.52	95.05	114.99	2.18
Myof	3465.87	2223.76	720.87	534.76	2.18
Sp140	66.15	87.32	13.88	20.10	2.18
Sh3kbp1	373.53	306.05	90.78	59.17	2.18
Cpne7	82.68	174.64	21.36	35.73	2.18
Nid1	3097.20	2631.54	486.98	780.37	2.18
-	22.37	55.95	11.75	5.58	2.18
Zfp57	160.50	137.34	20.29	45.77	2.18

Cks1b	193.58	245.01	46.99	50.24	2.17
Tekt2	36.96	32.22	6.41	8.93	2.17
Cped1	224.70	265.36	37.38	71.45	2.17
E2f8	85.60	72.06	16.02	18.98	2.17
Knl1	111.87	94.10	26.70	18.98	2.17
St6galnac4	207.19	260.27	43.79	60.29	2.17
Cercam	94.36	111.91	21.36	24.56	2.17
Steap4	100.19	91.56	18.16	24.56	2.17
Elf4	445.51	473.07	88.64	116.11	2.17
Ighm	376.45	424.74	102.52	75.92	2.17
Oas1a	382.29	355.22	83.30	81.50	2.16
Zfp239	278.20	258.58	57.67	62.52	2.16
Rnf150	221.78	183.97	38.45	52.47	2.16
Arhgap45	803.48	1066.52	174.08	245.61	2.16
Mxra8	1523.31	1622.67	358.83	347.20	2.16
Gm43197	49.61	18.65	7.48	7.81	2.15
Hhipl1	42.80	34.76	8.54	8.93	2.15
Dusp2	46.69	78.84	14.95	13.40	2.15
Olfr1372-ps1	44.75	32.22	12.82	4.47	2.15
Tnfaip8l1	55.45	41.54	8.54	13.40	2.15
Ptrh1	290.85	304.36	83.30	51.36	2.14
Prf1	20.43	27.98	3.20	7.81	2.14
Map4k1	78.79	113.60	22.43	21.21	2.14
Paqr6	35.99	31.37	8.54	6.70	2.14
Ifi213	158.56	131.41	20.29	45.77	2.14
Unc13d	96.30	124.63	21.36	29.03	2.14
Selenon	486.37	409.48	106.79	97.13	2.13
Uhrf1	448.43	394.22	126.02	65.87	2.13
Sobp	38.91	56.80	9.61	12.28	2.13
Rgs10	197.47	185.67	39.51	48.01	2.13
Slc20a1	2496.05	3326.73	625.82	705.57	2.13
Irak3	119.65	105.13	22.43	29.03	2.13
H2-Eb1	2548.58	3135.98	558.54	743.53	2.13
Cfh	5274.20	5734.46	910.96	1610.99	2.13
Dnah1	45.72	34.76	13.88	4.47	2.13
Bbc3	371.59	473.07	82.23	111.64	2.13
AI839979	28.21	19.50	5.34	5.58	2.13
Pvr	879.36	1107.22	187.96	267.94	2.13
Gypc	58.36	32.22	10.68	10.05	2.12
Klrk1	17.51	25.43	3.20	6.70	2.12
Kif2c	165.37	200.93	32.04	52.47	2.12
Gm16091	97.27	50.87	9.61	24.56	2.12
Nlgn2	688.70	843.55	164.46	190.91	2.11
Sox4	1196.47	1236.08	284.07	280.22	2.11
Ifitm3	2515.51	2242.41	536.11	568.25	2.11
Rab31	1115.73	1257.27	238.15	313.71	2.11
Traf1	338.51	381.51	105.73	61.40	2.11
Kcnd3	128.40	73.76	23.49	23.44	2.10
Shc4	44.75	34.76	11.75	6.70	2.10
Psmb9	387.15	447.63	91.84	102.71	2.10
P2rx7	228.59	238.23	53.40	55.82	2.10
Rad51ap1	91.44	81.39	18.16	22.33	2.09
Ccnf	188.71	222.12	37.38	59.17	2.09
Rinl	223.73	270.45	49.13	66.98	2.09
Ak5	149.80	155.15	17.09	54.70	2.09
Card9	101.16	120.39	37.38	14.51	2.09
Ifitm1	371.59	260.27	80.10	68.10	2.09
Gm8126	35.99	38.15	9.61	7.81	2.09
Rasgrp4	56.42	55.11	10.68	15.63	2.09
Epha3	40.86	23.74	10.68	4.47	2.09
Tpm4	4761.56	3984.62	1026.30	1034.92	2.08
Pgbd5	45.72	61.04	9.61	15.63	2.08

-	12.65	125.47	19.22	13.40	2.08
Gpc6	88.52	50.87	13.88	18.98	2.08
Lbh	2310.26	2278.02	415.43	668.73	2.08
Psmb10	1015.54	991.07	257.38	216.58	2.08
Tnik	52.53	44.93	7.48	15.63	2.08
Ankrd6	522.36	445.09	115.34	113.87	2.08
Nfkb2	2262.59	2299.21	504.07	579.42	2.07
C1s1	878.38	877.46	191.16	226.63	2.07
Alox5	24.32	26.28	4.27	7.81	2.07
Zfp575	20.43	30.52	1.07	11.16	2.07
Inpp5d	541.82	642.63	112.13	170.81	2.07
Fam198b	147.86	113.60	26.70	35.73	2.07
Ier5	1166.32	1748.99	313.98	382.93	2.07
Gdf15	1133.24	1314.08	213.59	371.77	2.07
Lxn	197.47	276.38	57.67	55.82	2.06
Xaf1	469.83	407.79	93.98	116.11	2.06
Rftn1	338.51	356.07	72.62	93.78	2.06
Gm4951	88.52	89.87	14.95	27.91	2.06
Csf2rb	245.13	250.95	55.53	63.64	2.06
Fes	514.58	507.83	121.75	123.92	2.06
Lamb3	492.21	298.42	92.91	97.13	2.06
Krt5	74.90	10.17	18.16	2.23	2.05
Neurl1a	81.71	72.06	19.22	17.86	2.05
Plekho1	1092.39	1335.27	260.58	325.99	2.05
Lrrc17	22.37	22.89	4.27	6.70	2.05
Naip2	145.91	116.15	28.83	34.61	2.05
Havcr2	48.64	37.30	6.41	14.51	2.04
Hesx1	56.42	60.19	14.95	13.40	2.04
Gpx7	163.42	139.89	51.26	22.33	2.04
Ndc80	84.63	104.28	17.09	29.03	2.04
Cenpn	91.44	105.97	21.36	26.79	2.04
Lix1l	422.17	372.18	86.50	107.18	2.04
9930111J21Rik2	66.15	64.43	9.61	22.33	2.04
Zmynd15	978.58	1086.87	256.31	247.84	2.03
Tcf21	922.16	1322.55	245.63	303.66	2.03
Pcdhb20	29.18	33.06	8.54	6.70	2.03
Tgif1	979.55	1144.52	262.72	257.89	2.03
Efna2	25.29	27.98	7.48	5.58	2.03
Dock11	104.08	109.37	25.63	26.79	2.03
Gm867	33.07	24.59	8.54	5.58	2.03
Rasa3	517.50	508.67	123.88	128.39	2.02
Serping1	2545.66	2757.86	527.57	777.02	2.02
Hic1	411.47	395.07	74.76	123.92	2.02
1810064F22Rik	15.56	33.06	6.41	5.58	2.02
Oasl1	349.21	408.64	86.50	100.48	2.02
Cfap157	77.82	72.06	24.56	12.28	2.02
Smpd5	40.86	51.72	13.88	8.93	2.02
9330175E14Rik	48.64	53.41	9.61	15.63	2.02
Acap1	117.70	161.08	25.63	43.54	2.01
Lepr	316.14	256.88	72.62	69.22	2.01
Ppfia4	159.53	218.73	48.06	45.77	2.01
B4galt2	214.98	217.03	48.06	59.17	2.01
St8sia4	104.08	120.39	26.70	29.03	2.01
Wdr66	99.22	82.24	14.95	30.14	2.01
Ube2l6	450.38	443.39	100.39	121.69	2.01
Gm15418	21.40	44.09	10.68	5.58	2.01
Lgals9	660.49	628.21	149.51	170.81	2.01
Serpinb9	1684.79	1630.30	461.35	362.83	2.01
Rasa4	382.29	417.11	89.71	109.41	2.01
Atg4a	11.67	84.78	9.61	14.51	2.01
Rin1	297.66	206.86	63.01	62.52	2.01
Msi1	30.15	56.80	14.95	6.70	2.00

Gm17383	32.10	24.59	9.61	4.47	2.00
Calml3	103.11	85.63	20.29	26.79	2.00
Carmil2	40.86	55.11	13.88	10.05	2.00
Colec11	32.10	20.35	7.48	5.58	2.00
Tspyl3	37.94	71.21	12.82	14.51	2.00
Gm20559	457.19	529.02	144.17	102.71	2.00
Rasal1	87.55	60.19	20.29	16.75	1.99
Slc22a15	248.05	235.69	49.13	72.57	1.99
Gem	212.06	244.16	54.47	60.29	1.99
Cftr	261.67	282.31	55.53	81.50	1.99
Pip4k2a	594.34	612.11	130.29	174.16	1.99
Tlr4	141.05	105.97	30.97	31.26	1.99
Ppm1j	265.56	278.92	68.35	69.22	1.99
5-Sep	67.12	83.93	19.22	18.98	1.98
Alpk1	266.53	204.32	50.19	69.22	1.98
Negr1	47.66	16.96	7.48	8.93	1.97
Apobec3	761.66	700.28	220.00	151.83	1.97
Mical2	415.36	400.16	88.64	119.46	1.97
B3galt1	39.88	41.54	9.61	11.16	1.97
Stc2	249.02	228.06	46.99	74.80	1.97
Tfr2	90.46	77.15	16.02	26.79	1.97
Pea15a	3674.04	3097.83	877.85	852.94	1.97
Tmem71	39.88	37.30	6.41	13.40	1.97
1700058P15Rik	41.83	51.72	13.88	10.05	1.97
Katnal2	27.24	36.46	9.61	6.70	1.96
Slc2a3	71.01	86.47	20.29	20.10	1.96
Slc37a2	179.96	148.36	40.58	43.54	1.96
Ccdc8	66.15	70.37	13.88	21.21	1.96
Robo1	127.43	114.45	36.31	25.68	1.96
Cxcl11	41.83	39.85	3.20	17.86	1.96
Sphk1	585.59	651.95	175.14	142.90	1.96
Zfp599	31.13	28.82	3.20	12.28	1.96
Adcy3	124.51	122.08	26.70	36.84	1.96
Ptpre	180.93	182.28	25.63	68.10	1.96
Tap1	1086.55	1279.32	300.09	309.25	1.96
Twist1	51.56	50.02	13.88	12.28	1.96
Serpinb6b	626.44	645.17	179.42	148.48	1.95
Scn2b	43.77	27.98	11.75	6.70	1.95
Sgol2a	39.88	40.69	7.48	13.40	1.95
Ano5	58.36	72.91	12.82	21.21	1.95
Heyl	330.73	467.98	81.16	126.15	1.95
Slit3	351.16	398.46	92.91	101.59	1.95
Des	574.89	506.13	144.17	136.20	1.95
Nsl1	97.27	78.84	28.83	16.75	1.95
Dnaaf3	42.80	66.98	8.54	20.10	1.94
Samd4	321.98	306.05	60.87	102.71	1.94
Tifa	549.60	506.13	119.61	155.18	1.94
Ugcg	877.41	1021.59	218.93	275.75	1.94
Slfn2	112.84	82.24	9.61	41.31	1.94
Lpxn	133.27	147.52	36.31	36.84	1.94
Ccna2	377.42	392.53	106.79	93.78	1.94
A230050P20Rik	616.72	561.24	148.44	158.53	1.94
Gpr173	30.15	40.69	11.75	6.70	1.94
Top2a	860.87	905.44	252.04	208.77	1.94
Prelp	624.50	740.97	128.15	228.86	1.94
Filip1l	572.94	492.57	147.38	130.62	1.94
Ccne1	63.23	53.41	19.22	11.16	1.94
Cxcl16	2678.93	2797.71	757.18	674.31	1.94
Plekhg2	1456.19	1375.97	363.10	377.35	1.94
Ndn	146.88	116.15	35.24	33.49	1.93
Fndc4	146.88	211.10	49.13	44.66	1.93
Slc9a5	154.67	154.30	36.31	44.66	1.93

Epha2	595.32	615.50	158.06	159.65	1.93
4930503L19Rik	163.42	132.26	37.38	40.19	1.93
Hoxa11	63.23	73.76	19.22	16.75	1.93
Rubcnl	25.29	37.30	5.34	11.16	1.93
Dkk3	653.68	939.35	145.24	275.75	1.92
Pknox2	176.07	117.00	44.85	32.38	1.92
Tagap	61.28	78.84	19.22	17.86	1.92
Rem1	306.41	325.55	48.06	119.46	1.92
Frmd6	445.51	345.90	79.03	130.62	1.92
Ifrd1	638.12	895.27	193.30	213.24	1.92
Bst2	1556.38	1639.63	441.06	406.37	1.91
Gm13212	25.29	23.74	9.61	3.35	1.91
Ano1	239.29	254.34	63.01	68.10	1.91
Cd86	59.34	80.54	14.95	22.33	1.91
Cpt1c	151.75	163.62	45.92	37.96	1.91
Tmem98	449.41	594.30	120.68	157.41	1.91
Arl4c	1054.45	1036.00	259.51	296.97	1.91
Col8a1	929.94	689.25	121.75	309.25	1.91
Efhd2	2729.51	2958.79	787.08	726.79	1.91
Hoxa11os	49.61	40.69	9.61	14.51	1.91
Slc2a10	45.72	32.22	8.54	12.28	1.90
Apaf1	421.20	353.53	85.44	121.69	1.90
Sult5a1	119.65	145.82	34.17	36.84	1.90
Birc5	249.99	283.16	83.30	59.17	1.90
Traip	40.86	48.32	17.09	6.70	1.90
Adam11	598.24	618.89	187.96	137.32	1.90
A630001G21Rik	100.19	115.30	32.04	25.68	1.90
Nrcam	40.86	48.32	14.95	8.93	1.90
Ctsc	2334.58	2621.37	639.70	691.06	1.90
-	78.79	83.08	26.70	16.75	1.90
Chst12	126.46	137.34	36.31	34.61	1.90
Ggta1	247.08	315.38	54.47	97.13	1.89
Col4a1	19161.03	18635.30	4679.75	5487.17	1.89
-	53.50	64.43	12.82	18.98	1.89
Matk	27.24	57.65	10.68	12.28	1.89
Uba7	830.72	728.25	205.05	215.47	1.89
Disc1	42.80	22.04	8.54	8.93	1.89
Slc7a2	398.82	301.81	82.23	107.18	1.89
Csf2ra	828.77	874.92	261.65	198.72	1.89
Thbs3	205.25	184.82	27.77	78.15	1.88
Cfi	1541.79	1154.69	328.93	401.91	1.88
Dlgap5	179.96	191.60	38.45	62.52	1.88
Unc93b1	1378.37	1565.87	371.65	427.59	1.88
Mvp	3607.89	3677.72	1038.05	938.90	1.88
Espl1	233.46	264.51	71.55	63.64	1.88
Sema3a	28.21	16.11	5.34	6.70	1.88
Ly6e	13371.28	14475.19	4040.05	3533.45	1.88
Tes	1356.00	1375.97	368.44	375.12	1.88
Lama4	263.61	138.19	50.19	59.17	1.88
Map3k8	93.38	87.32	20.29	29.03	1.88
Anln	319.06	308.60	83.30	88.20	1.87
Fbln1	482.48	408.64	120.68	122.81	1.87
Tbc1d2b	990.25	861.36	234.95	271.29	1.87
Nfatc4	225.68	300.97	67.28	77.03	1.87
Pros1	1496.07	1537.89	393.00	437.63	1.87
Gm28727	35.99	44.09	7.48	14.51	1.87
H2-T10	88.52	102.58	27.77	24.56	1.87
Gm19221	48.64	31.37	8.54	13.40	1.87
Armcx6	40.86	34.76	10.68	10.05	1.87
Thbs2	491.23	569.72	121.75	169.69	1.87
Igfbp6	31.13	32.22	10.68	6.70	1.86
Prr33	34.05	49.17	11.75	11.16	1.86

Hs3st1	71.98	73.76	29.90	10.05	1.86
Irf7	1833.61	1969.42	538.25	507.97	1.86
Ifit2	872.55	607.87	213.59	194.26	1.86
Mxra7	526.25	561.24	158.06	141.78	1.86
Pir	60.31	71.21	9.61	26.79	1.86
Calhm2	93.38	148.36	26.70	40.19	1.86
Msl3l2	37.94	50.02	3.20	21.21	1.86
Smox	701.35	845.25	199.71	227.75	1.86
Hivep3	172.17	132.26	42.72	41.31	1.86
Rras	1414.36	1576.04	423.98	403.03	1.85
Rgs4	73.93	95.80	18.16	29.03	1.85
Esyt1	1386.15	1351.38	422.91	336.04	1.85
Scarf2	1005.81	1075.00	239.22	339.39	1.85
Cpne2	428.98	396.77	107.86	121.69	1.85
Scimp	54.47	32.22	10.68	13.40	1.85
Trim30b	24.32	27.13	3.20	11.16	1.85
Plp2	942.58	1067.37	296.89	262.36	1.85
Jchain	71.01	173.80	18.16	50.24	1.85
Lrrc8e	49.61	55.95	16.02	13.40	1.84
Pmaip1	836.56	1163.17	192.23	366.18	1.84
Lag3	35.99	45.78	11.75	11.16	1.84
Nrg1	759.71	582.43	181.55	194.26	1.84
1700047I17Rik2	97.27	70.37	24.56	22.33	1.84
Scn8a	39.88	14.41	10.68	4.47	1.83
Stra6	190.66	198.38	82.23	26.79	1.83
Psd3	547.65	359.46	142.04	112.76	1.83
Tmsb4x	8588.32	8662.73	2578.03	2277.48	1.83
-	171.20	148.36	64.08	25.68	1.83
Ncaph	184.82	210.25	55.53	55.82	1.83
Mki67	1134.21	978.35	307.57	288.03	1.83
6720489N17Rik	26.26	20.35	5.34	7.81	1.83
Tmeff1	31.13	42.39	9.61	11.16	1.83
Il20rb	73.93	72.91	22.43	18.98	1.83
Cklf	135.21	120.39	30.97	41.31	1.82
Cenpi	51.56	52.56	16.02	13.40	1.82
Lrrc71	23.35	22.89	7.48	5.58	1.82
Socs1	35.99	52.56	12.82	12.28	1.82
Krt18	1650.74	978.35	443.20	300.32	1.82
Spock2	119.65	195.84	38.45	51.36	1.82
Lgals3	2453.25	2218.67	684.55	641.94	1.82
Mmd	485.40	759.62	170.87	183.09	1.82
Trp53i11	607.96	579.89	133.49	204.30	1.82
Pxdc1	269.45	294.18	53.40	107.18	1.81
Adamts7	202.33	236.53	48.06	77.03	1.81
Dck	298.63	329.79	86.50	92.66	1.81
Chrnb1	454.27	521.39	137.77	140.67	1.81
Bub1b	274.31	307.75	75.82	90.43	1.81
Ptger4	222.76	228.06	58.74	70.33	1.81
Lfng	339.49	347.59	95.05	101.59	1.81
Jun	2712.00	3253.82	961.15	745.76	1.81
P3h3	542.79	502.74	149.51	149.60	1.81
Spon1	1118.65	1180.97	310.77	349.44	1.80
Il1b	31.13	37.30	9.61	10.05	1.80
Itga9	742.20	424.74	173.01	161.88	1.80
Arxes2	35.02	33.06	12.82	6.70	1.80
Zcchc24	481.51	381.51	121.75	126.15	1.80
Tagln	26.26	33.91	12.82	4.47	1.80
Chtf18	80.74	89.02	30.97	17.86	1.80
Cep55	110.89	120.39	30.97	35.73	1.80
Tpx2	374.50	416.27	106.79	121.69	1.79
Tm6sf1	157.58	190.75	41.65	59.17	1.79
Tenm3	180.93	111.06	35.24	49.12	1.79

Man1c1	439.68	434.92	104.66	148.48	1.79
Hrct1	44.75	49.17	17.09	10.05	1.79
Prrg1	109.92	78.00	33.11	21.21	1.79
Plk4	22.37	26.28	9.61	4.47	1.79
Cdkn1a	878.38	1636.24	240.29	488.99	1.79
Apol9b	66.15	57.65	22.43	13.40	1.79
Cenpa	378.40	467.13	96.12	149.60	1.79
Ccnb1	277.23	314.53	69.42	102.71	1.78
Fam49a	174.12	164.47	44.85	53.59	1.78
Vwa7	47.66	38.15	18.16	6.70	1.78
Igha	655.63	1041.09	110.00	384.05	1.78
Tpm2	882.28	626.52	214.66	224.40	1.78
Unc5b	738.31	852.03	220.00	243.38	1.78
AI506816	907.57	944.44	295.82	243.38	1.78
Il16	145.91	124.63	33.11	45.77	1.78
Arpc1b	3372.49	3386.08	1048.73	922.16	1.78
Il2rg	97.27	143.28	22.43	48.01	1.78
Sh3pxd2b	504.85	338.27	115.34	130.62	1.78
Zkscan7	36.96	22.89	9.61	7.81	1.78
Asns	400.77	407.79	102.52	133.97	1.77
H1fx	65.17	76.30	23.49	17.86	1.77
Herc6	236.38	217.03	69.42	63.64	1.77
Trip13	44.75	59.35	13.88	16.75	1.77
Rcn1	813.21	748.60	239.22	219.93	1.77
Dtx1	26.26	29.67	5.34	11.16	1.77
Gm5637	97.27	72.91	30.97	18.98	1.76
Plk1	257.78	259.42	59.81	92.66	1.76
Klhl23	86.57	87.32	28.83	22.33	1.76
Cdca2	137.16	133.95	35.24	44.66	1.76
Cenps	58.36	37.30	21.36	6.70	1.76
Gc	3444.47	3162.26	855.43	1091.85	1.76
Fignl1	99.22	71.21	26.70	23.44	1.76
Vash1	93.38	50.87	25.63	16.75	1.76
Col14a1	1596.27	1613.35	344.95	601.75	1.76
AA414768	41.83	39.00	18.16	5.58	1.76
Pcdhb17	60.31	69.52	16.02	22.33	1.76
Hoxd11	90.46	102.58	21.36	35.73	1.76
Tril	468.86	501.89	127.09	159.65	1.76
Nxnl2	38.91	39.00	8.54	14.51	1.76
Armcx2	516.52	467.13	137.77	152.95	1.76
Fbln2	404.66	328.94	110.00	107.18	1.76
Mcm3	367.70	276.38	104.66	85.96	1.75
Ube2c	325.87	360.31	89.71	113.87	1.75
Adamts16	250.97	284.86	57.67	101.59	1.75
Gpm6b	313.22	307.75	99.32	84.85	1.75
P2ry6	272.37	241.62	86.50	65.87	1.75
Cenph	44.75	43.24	16.02	10.05	1.75
Kif15	109.92	94.95	37.38	23.44	1.75
Slc17a9	38.91	52.56	14.95	12.28	1.75
Gm6169	118.67	119.54	37.38	33.49	1.75
Cdh3	708.15	591.76	214.66	171.93	1.75
Cep170	377.42	329.79	79.03	131.74	1.75
Hn1	1318.06	1515.00	394.07	449.91	1.75
Slamf7	47.66	59.35	8.54	23.44	1.75
Clec7a	132.29	146.67	30.97	52.47	1.74
Numbl	246.10	305.20	84.37	80.38	1.74
Mmp23	262.64	284.86	79.03	84.85	1.74
Gabra3	130.35	133.10	26.70	52.47	1.74
Celf2	321.98	302.66	73.69	113.87	1.74
Rasl11a	315.17	491.72	121.75	120.57	1.74
Tpm1	5147.74	4306.78	1453.48	1384.35	1.74
Smarca1	59.34	56.80	18.16	16.75	1.73

Ska3	71.98	72.91	24.56	18.98	1.73
Clca3a1	941.61	907.98	202.91	353.90	1.73
Dusp5	133.27	149.21	46.99	37.96	1.73
Cmklr1	209.14	196.69	67.28	54.70	1.73
Pif1	75.87	101.73	26.70	26.79	1.73
H2-Q4	1811.24	2275.47	530.77	701.11	1.73
Cep85	10692.36	14443.82	4001.60	3578.11	1.73
Melk	121.59	122.93	51.26	22.33	1.73
Zfp36l1	3782.99	3950.71	1163.00	1173.35	1.73
Arrb2	868.66	844.40	249.90	267.94	1.73
Egflam	329.76	337.42	74.76	127.27	1.73
Cd27	46.69	50.87	12.82	16.75	1.72
Nek5	48.64	52.56	12.82	17.86	1.72
Gadd45b	191.63	243.32	68.35	63.64	1.72
Rnf213	3359.84	2465.38	831.93	936.67	1.72
H2-K1	20090.00	24137.46	6673.61	6762.12	1.72
Clstn3	26.26	41.54	12.82	7.81	1.72
Fos	177.04	223.82	66.21	55.82	1.72
Litaf	3040.78	2986.77	916.30	919.93	1.71
Phlda3	357.00	423.90	115.34	122.81	1.71
Lpar1	145.91	167.01	34.17	61.40	1.71
1110032F04Rik	35.99	21.19	10.68	6.70	1.71
Arntl2	95.33	66.13	22.43	26.79	1.71
Prex1	703.29	629.91	185.82	221.05	1.71
2010016I18Rik	28.21	28.82	10.68	6.70	1.71
-	137.16	36.46	11.75	41.31	1.71
Sp100	756.79	866.44	226.41	270.17	1.71
Trim6	58.36	77.15	20.29	21.21	1.71
Ccdc74a	35.99	35.61	8.54	13.40	1.71
Myo1b	613.80	506.98	137.77	205.42	1.71
Igkc	406.61	558.69	89.71	206.54	1.71
Gm1673	35.02	33.06	6.41	14.51	1.71
Sntg2	89.49	102.58	26.70	32.38	1.70
Mcm5	416.33	403.55	161.26	90.43	1.70
I830077J02Rik	41.83	74.61	21.36	14.51	1.70
Arg2	403.69	562.93	121.75	176.39	1.70
Ankle1	176.07	202.62	60.87	55.82	1.70
Wnt4	147.86	92.41	45.92	27.91	1.70
Cd109	69.06	40.69	17.09	16.75	1.69
Slfn5	879.36	683.32	203.98	279.10	1.69
Dtx3l	782.08	727.40	216.79	250.08	1.69
Cdr2l	319.06	250.10	82.23	93.78	1.69
Fam212a	59.34	46.63	16.02	16.75	1.69
Cald1	2170.18	1603.17	553.20	616.26	1.69
Kirrel	700.37	712.99	176.21	262.36	1.69
Serp2	45.72	39.00	10.68	15.63	1.69
Abi3	48.64	84.78	20.29	21.21	1.69
Racgap1	401.74	410.33	127.09	125.04	1.69
Apol9a	62.26	26.28	9.61	17.86	1.69
Gm8995	231.51	228.90	70.48	72.57	1.69
Fcho1	110.89	150.91	49.13	32.38	1.68
Ighg2b	45.72	69.52	20.29	15.63	1.68
Mfng	193.58	213.64	54.47	72.57	1.68
Adgrg3	515.55	417.11	199.71	90.43	1.68
Mcam	1227.60	942.74	343.88	332.69	1.68
Cfap44	32.10	27.13	11.75	6.70	1.68
Ifngr2	1371.56	1376.81	412.23	446.57	1.68
Msn	7764.41	6090.53	2230.94	2101.09	1.68
Mex3a	258.75	196.69	89.71	52.47	1.68
Matn2	569.05	359.46	134.56	156.30	1.67
Itga5	659.52	646.86	199.71	209.89	1.67
Snai2	71.01	75.45	20.29	25.68	1.67

Dsel	144.94	133.95	39.51	48.01	1.67
Ddx58	1824.86	1764.25	568.15	558.21	1.67
Orai2	159.53	165.32	34.17	68.10	1.67
Rab8b	627.42	679.08	191.16	219.93	1.67
Ttc16	56.42	37.30	16.02	13.40	1.67
Tgfb2	142.99	69.52	26.70	40.19	1.67
Bace2	74.90	100.89	35.24	20.10	1.67
Mndal	363.80	412.03	85.44	159.65	1.66
Scn1b	601.15	807.95	227.47	217.70	1.66
Tead2	178.98	128.86	49.13	48.01	1.66
Ccnb2	360.89	378.96	112.13	121.69	1.66
Tap2	1043.75	1120.78	324.66	359.49	1.66
Gstm6	103.11	89.87	36.31	24.56	1.66
Nuf2	137.16	135.65	39.51	46.89	1.66
Fancd2	109.92	59.35	22.43	31.26	1.66
Fut10	51.56	69.52	10.68	27.91	1.66
Ggt7	93.38	89.87	17.09	41.31	1.65
Hacd4	287.93	198.38	92.91	61.40	1.65
Elmo1	144.94	118.69	46.99	36.84	1.65
Col4a2	12165.08	12364.19	3663.06	4150.83	1.65
Btg2	1129.35	1305.60	365.24	410.84	1.65
Sapcd2	41.83	54.26	12.82	17.86	1.65
Elf3	1296.66	1280.16	431.45	389.63	1.65
Crlf2	457.19	449.33	151.65	137.32	1.65
Tpst1	212.06	175.49	54.47	69.22	1.65
Abca1	2172.13	1537.89	657.86	525.83	1.65
Adamtsl1	144.94	135.65	41.65	48.01	1.65
Sync	34.05	27.13	12.82	6.70	1.64
Sdk2	99.22	44.09	24.56	21.21	1.64
-	184.82	143.28	39.51	65.87	1.64
Plbd1	278.20	284.86	91.84	89.31	1.64
Cdk5r1	21.40	49.17	16.02	6.70	1.64
Trim59	176.07	187.36	41.65	75.92	1.63
Olfml3	912.43	1008.02	261.65	360.60	1.63
Lrrc32	1118.65	930.87	268.06	396.33	1.63
Adamts10	1006.79	1216.58	352.42	368.42	1.63
Slc39a6	370.61	417.96	118.54	137.32	1.63
Kng1	80.74	80.54	28.83	23.44	1.62
Sash1	3186.70	2715.48	873.58	1041.61	1.62
Stk10	346.30	350.14	110.00	116.11	1.62
Nrp2	951.34	750.30	201.84	351.67	1.62
Cx3cl1	3808.28	3592.09	1143.77	1262.66	1.62
Cdk1	325.87	367.94	117.47	108.29	1.62
Iqgap3	343.38	317.07	112.13	102.71	1.62
Ppp1r3d	56.42	64.43	17.09	22.33	1.62
Zgrf1	45.72	55.11	12.82	20.10	1.62
Tvp23a	32.10	27.98	12.82	6.70	1.62
Col18a1	14379.04	14971.15	4820.72	4743.64	1.62
Rad18	90.46	98.34	17.09	44.66	1.62
Layn	83.66	78.00	16.02	36.84	1.62
Ngfr	97.27	94.10	22.43	40.19	1.61
Ecm2	108.95	122.08	32.04	43.54	1.61
Golm1	613.80	482.39	212.52	145.13	1.61
Anxa9	203.30	143.28	65.14	48.01	1.61
Map1a	142.02	117.84	45.92	39.07	1.61
Brca1	125.48	128.02	39.51	43.54	1.61
Fam114a1	251.94	183.97	82.23	60.29	1.61
Tubb5	4472.66	4234.72	1451.34	1401.10	1.61
Fbxw17	613.80	645.17	203.98	208.77	1.61
Stap2	710.10	769.79	280.87	204.30	1.61
Nexn	129.37	97.50	33.11	41.31	1.61
Mast1	65.17	44.93	16.02	20.10	1.61

Ppp1r14a	202.33	153.45	57.67	59.17	1.61
H2-M3	133.27	155.15	49.13	45.77	1.60
Il6ra	283.07	334.88	90.78	112.76	1.60
Stx1a	115.76	139.89	38.45	45.77	1.60
Igsf9	326.84	342.51	112.13	108.29	1.60
Pkmyt1	163.42	163.62	64.08	43.54	1.60
Cstb	2896.82	3503.07	1071.15	1041.61	1.60
Bub1	87.55	107.67	26.70	37.96	1.60
Aebp1	3522.29	3867.62	1296.49	1153.26	1.59
Cd1d1	85.60	111.91	30.97	34.61	1.59
Ncapg2	171.20	173.80	64.08	50.24	1.59
6030419C18Rik	94.36	112.76	32.04	36.84	1.59
Naalad2	116.73	94.10	28.83	41.31	1.59
C1ql3	72.96	84.78	22.43	30.14	1.59
Zwilch	114.78	124.63	38.45	41.31	1.59
Zc3hav1l	112.84	122.93	41.65	36.84	1.59
Mob3a	130.35	144.12	54.47	36.84	1.59
Serpinb6a	1224.68	1381.05	433.59	434.29	1.59
Parp3	1621.56	1543.83	567.08	487.87	1.58
Cnrip1	37.94	67.82	6.41	29.03	1.58
Shcbp1	87.55	89.02	29.90	29.03	1.58
Atoh8	142.02	269.60	66.21	71.45	1.58
Ifi203	431.90	459.50	124.95	173.04	1.58
Obsl1	346.30	311.14	105.73	113.87	1.58
F3	969.82	712.14	318.25	243.38	1.58
Sgce	178.01	204.32	59.81	68.10	1.58
Cacna1c	141.05	87.32	43.79	32.38	1.58
Cfap65	219.84	228.90	59.81	90.43	1.58
Cpne8	263.61	200.93	98.25	56.94	1.58
Abca8b	91.44	104.28	28.83	36.84	1.58
Cenpt	288.90	280.62	97.18	93.78	1.58
Psrc1	83.66	146.67	40.58	36.84	1.57
Zdhhc15	69.06	93.26	26.70	27.91	1.57
G530011O06Rik	21.40	63.58	6.41	22.33	1.57
Tpbg	80.74	88.17	26.70	30.14	1.57
Cacna2d1	169.26	142.43	49.13	55.82	1.57
2700099C18Rik	35.02	39.85	8.54	16.75	1.57
Aldh18a1	339.49	281.47	108.93	100.48	1.57
Zfand4	32.10	39.00	11.75	12.28	1.57
Rtn2	131.32	124.63	35.24	51.36	1.56
Mapk11	243.18	221.27	82.23	74.80	1.56
Phf11b	142.99	101.73	48.06	34.61	1.56
Eda2r	52.53	38.15	11.75	18.98	1.56
Kcnip3	71.01	79.69	33.11	17.86	1.56
Chst2	386.18	304.36	110.00	123.92	1.56
Uchl1	202.33	223.82	59.81	84.85	1.56
Rprm	57.39	20.35	10.68	15.63	1.56
BC028528	35.02	35.61	11.75	12.28	1.56
Lama2	425.09	379.81	84.37	189.79	1.56
Sipa1	1082.66	1188.60	414.36	358.37	1.56
Cenpw	35.02	44.93	14.95	12.28	1.55
Cldn3	852.12	794.38	263.78	296.97	1.55
Lmnb1	544.73	490.87	205.05	147.37	1.55
AU020206	369.64	607.87	155.92	177.51	1.55
AC166344.3	57.39	60.19	28.83	11.16	1.55
Parp9	1055.42	935.96	339.61	339.39	1.55
9830144P21Rik	131.32	150.06	46.99	49.12	1.55
Map3k6	333.65	253.49	85.44	114.99	1.55
B2m	12471.50	15133.92	4234.41	5191.33	1.55
Ckap2	170.23	198.38	50.19	75.92	1.55
Zeb2	1026.24	1027.52	332.13	369.53	1.55
Ccdc102a	220.81	213.64	72.62	75.92	1.55

D16Ertd472e	39.88	27.13	12.82	10.05	1.55
Knstrn	313.22	287.40	97.18	108.29	1.55
Map3k1	2798.57	2336.51	875.72	880.85	1.55
Zfp9	60.31	55.11	13.88	25.68	1.55
Nfkb1	1919.22	1917.70	594.85	720.09	1.55
Steap1	279.18	288.25	91.84	102.71	1.54
Prc1	323.92	342.51	103.59	125.04	1.54
Klhl6	178.01	210.25	65.14	68.10	1.54
Sdc1	1039.86	1097.04	389.80	343.86	1.54
Atp8b2	637.14	662.12	216.79	229.98	1.54
Dok1	260.69	250.95	81.16	94.90	1.54
Ube2t	61.28	55.95	22.43	17.86	1.54
B4galnt1	861.85	849.49	356.69	232.21	1.54
Dok3	232.48	295.88	91.84	90.43	1.54
Spred3	303.49	268.75	105.73	91.55	1.54
Mis18bp1	74.90	92.41	29.90	27.91	1.53
Ncam1	187.74	171.25	63.01	61.40	1.53
Emp1	1346.27	1351.38	501.94	433.17	1.53
Gbp7	827.80	706.21	231.74	300.32	1.53
Plxnd1	2290.80	2192.39	678.15	877.50	1.53
Ptgfrn	817.10	835.07	277.67	295.85	1.53
Plat	2298.58	2214.43	814.84	751.35	1.53
Nupr1	591.43	688.41	237.08	207.65	1.53
H2-K2	325.87	445.09	107.86	160.76	1.52
Bend4	38.91	39.85	8.54	18.98	1.52
Batf2	26.26	36.46	8.54	13.40	1.52
Timp2	2030.11	2122.02	697.37	751.35	1.52
Atp1b2	495.12	764.71	181.55	259.01	1.52
Ephb6	199.41	175.49	61.94	69.22	1.52
-	57.39	70.37	21.36	23.44	1.51
Fam57a	165.37	180.58	56.60	64.75	1.51
Lgals1	3968.78	3530.20	1399.01	1229.17	1.51
Fgf2	149.80	161.08	54.47	54.70	1.51
Vwf	195.52	105.97	57.67	48.01	1.51
Csdc2	130.35	211.10	53.40	66.98	1.51
Slc43a3	1445.49	1600.63	387.67	685.48	1.51
A530020G20Rik	78.79	81.39	38.45	17.86	1.51
Eme1	41.83	47.48	20.29	11.16	1.50
Ptk7	720.80	747.75	270.19	247.84	1.50
Ikbke	321.00	401.01	143.11	111.64	1.50
Usp18	200.38	233.99	58.74	94.90	1.50
Prr5l	220.81	257.73	86.50	82.61	1.50
Trim47	2595.27	3299.60	910.96	1173.35	1.50
Camkk1	105.06	77.15	34.17	30.14	1.50
BC052040	173.15	148.36	54.47	59.17	1.50
Sema6b	334.62	271.29	133.49	80.38	1.50
Cnp	516.52	545.13	187.96	187.56	1.50
Itpripl2	999.00	848.64	292.62	361.72	1.50
Zfp125	63.23	63.58	17.09	27.91	1.50
8-Sep	1249.97	1144.52	396.21	453.26	1.50
Foxm1	351.16	342.51	140.97	104.94	1.49
Gm6277	70.04	62.74	17.09	30.14	1.49
Kif18b	81.71	99.19	35.24	29.03	1.49
Syt13	63.23	44.93	13.88	24.56	1.49
Rad51	107.97	91.56	38.45	32.38	1.49
Il1r1	486.37	327.25	123.88	165.23	1.49
Pgm2l1	151.75	139.89	50.19	53.59	1.49
Wipf1	547.65	644.32	187.96	236.68	1.49
Zfp37	56.42	50.87	20.29	17.86	1.49
Baiap3	56.42	85.63	12.82	37.96	1.49
Sgo1	49.61	63.58	20.29	20.10	1.49
Efs	42.80	63.58	25.63	12.28	1.49

Gpr68	66.15	65.28	25.63	21.21	1.49
Adgrf1	77.82	72.06	27.77	25.68	1.49
St3gal1	964.96	1018.20	343.88	363.95	1.49
Zfp455	31.13	42.39	10.68	15.63	1.49
Synj2	187.74	176.34	63.01	66.98	1.49
Scx	162.45	142.43	64.08	44.66	1.49
Agbl2	35.02	56.80	12.82	20.10	1.48
Blm	61.28	61.04	19.22	24.56	1.48
Rhoj	407.58	295.88	137.77	113.87	1.48
Hip1	1014.57	999.55	354.56	367.30	1.48
Pxdn	1486.35	1672.69	437.86	695.53	1.48
Sh3bgrl3	1660.47	2016.90	772.13	547.04	1.48
Lsm5	20.43	77.15	11.75	23.44	1.48
Adamts5	375.48	444.24	119.61	175.28	1.48
H2-D1	16822.56	19283.86	6356.43	6620.34	1.48
Gpihbp1	424.11	408.64	112.13	187.56	1.48
Mamld1	60.31	49.17	17.09	22.33	1.47
Acat3	963.99	1167.41	374.85	392.98	1.47
Ndufa4l2	155.64	147.52	49.13	60.29	1.47
Vasp	2018.44	1865.14	747.56	654.22	1.47
S100a9	62.26	33.91	25.63	8.93	1.47
Stk17b	431.90	450.18	161.26	157.41	1.47
Sdk1	264.59	235.69	104.66	75.92	1.47
Arhgap19	233.46	250.10	80.10	94.90	1.47
Rad54b	45.72	44.93	14.95	17.86	1.47
Rasd1	246.10	362.85	107.86	112.76	1.47
Asf1b	125.48	129.71	57.67	34.61	1.47
Spdl1	99.22	70.37	23.49	37.96	1.46
Gins2	147.86	169.56	72.62	42.42	1.46
Larp6	60.31	39.00	19.22	16.75	1.46
Pqlc3	175.09	205.17	51.26	87.08	1.46
Ebf4	94.36	97.50	38.45	31.26	1.46
Fsd1l	97.27	113.60	27.77	49.12	1.46
Nemp2	128.40	108.52	44.85	41.31	1.46
Nlrc3	92.41	105.13	34.17	37.96	1.45
Aoc3	214.98	156.84	59.81	75.92	1.45
S1pr4	30.15	61.89	21.36	12.28	1.45
Rims3	26.26	48.32	12.82	14.51	1.45
Ska1	36.96	38.15	8.54	18.98	1.45
Pld2	652.71	675.69	210.39	275.75	1.45
Rell1	616.72	703.67	215.73	267.94	1.45
Klf10	523.33	810.49	191.16	298.08	1.45
Kif20a	240.27	211.95	59.81	106.06	1.45
Nfkbia	1310.28	1441.25	556.40	452.15	1.45
Sema4c	560.30	589.21	173.01	248.96	1.45
Olfr1033	107.00	75.45	23.49	43.54	1.45
Gtse1	122.57	189.91	49.13	65.87	1.45
Fhl3	229.57	190.75	93.98	60.29	1.44
Rhbdf2	1165.34	1249.64	447.47	442.10	1.44
Ifit1	376.45	277.23	98.25	142.90	1.44
Rnd3	515.55	555.30	168.74	226.63	1.44
Arhgef6	337.54	383.20	108.93	157.41	1.44
Rcsd1	516.52	516.30	202.91	178.63	1.44
C7	392.99	451.87	120.68	192.02	1.44
Tshz3	83.66	58.50	21.36	31.26	1.43
Clstn2	59.34	58.50	22.43	21.21	1.43
Cnr2	37.94	56.80	10.68	24.56	1.43
Usp35	151.75	165.32	68.35	49.12	1.43
Aurka	206.22	222.12	61.94	97.13	1.43
Incenp	392.01	442.55	155.92	154.07	1.43
Mrvi1	259.72	298.42	104.66	102.71	1.43
H2-DMb1	678.97	577.35	241.36	225.52	1.43

-	76.85	140.73	61.94	18.98	1.43
Aspm	157.58	125.47	36.31	69.22	1.42
Fam49b	550.57	507.83	236.02	158.53	1.42
Kif4	145.91	134.80	54.47	50.24	1.42
Trim5	74.90	66.13	19.22	33.49	1.42
Oas1b	212.06	154.30	61.94	74.80	1.42
Serpina1b	1343.35	1401.40	484.85	541.46	1.42
Galnt15	229.57	273.84	75.82	112.76	1.42
Slc7a5	330.73	227.21	132.43	75.92	1.42
Inf2	652.71	744.36	288.35	234.45	1.42
Gpsm3	174.12	172.10	68.35	61.40	1.42
Guca2a	53.50	77.15	26.70	22.33	1.42
Ripor3	358.94	366.25	95.05	177.51	1.41
Sema3c	1569.03	1337.81	575.62	515.78	1.41
Shisa4	562.24	643.47	253.10	199.84	1.41
Hmga1	1092.39	1169.10	441.06	409.72	1.41
Pcdhgc3	133.27	119.54	42.72	52.47	1.41
Cspg4	1427.01	1764.25	624.75	578.30	1.41
Nav1	755.82	596.00	293.69	216.58	1.40
Rasl12	497.07	602.78	195.43	221.05	1.40
Stambpl1	83.66	81.39	23.49	39.07	1.40
Klf4	452.32	617.19	183.69	222.17	1.40
Fam217b	130.35	83.93	29.90	51.36	1.40
Ehbp1l1	983.44	928.33	323.59	401.91	1.40
Itgb3	163.42	142.43	49.13	66.98	1.40
Notch3	1189.66	902.90	363.10	430.94	1.40
Sec14l2	181.90	200.93	64.08	81.50	1.40
Mxd3	79.76	89.87	28.83	35.73	1.40
Csrp1	2663.36	2212.74	946.20	908.76	1.39
Ckap4	777.22	696.04	268.06	292.50	1.39
Hap1	367.70	490.02	174.08	152.95	1.39
Eya2	65.17	92.41	26.70	33.49	1.39
Plscr1	1741.20	1845.64	705.91	662.03	1.39
Tapbpl	695.51	653.65	271.26	243.38	1.39
Gm31774	53.50	58.50	14.95	27.91	1.39
Rrad	439.68	614.65	216.79	186.44	1.39
Tnfrsf11a	215.95	222.97	89.71	78.15	1.39
Wdhd1	111.87	91.56	30.97	46.89	1.39
Phf11d	200.38	170.41	74.76	66.98	1.39
Dlg4	250.97	355.22	123.88	108.29	1.39
Asb2	97.27	107.67	42.72	35.73	1.39
Fbln5	2742.15	3572.59	971.83	1446.87	1.39
Trim14	80.74	101.73	32.04	37.96	1.38
Armcx3	393.96	415.42	151.65	158.53	1.38
Itgb6	2796.63	1787.99	1078.63	677.66	1.38
Cdkn3	81.71	89.02	34.17	31.26	1.38
CAAA01147332.1	53.50	82.24	33.11	18.98	1.38
Nek6	1077.80	891.03	294.75	461.08	1.38
Cenpe	195.52	183.12	69.42	75.92	1.38
Garnl3	53.50	82.24	30.97	21.21	1.38
Fut4	100.19	78.00	44.85	23.44	1.38
Spry1	706.21	760.47	205.05	359.49	1.38
Zc3hav1	1134.21	1016.50	398.34	428.70	1.38
Pla2r1	229.57	211.10	70.48	99.36	1.38
Tapbp	4331.61	4668.79	1847.55	1622.15	1.38
Tubb6	678.97	555.30	223.20	253.43	1.37
Arhgef40	1241.22	1294.58	477.37	502.39	1.37
Ap1s2	234.43	269.60	81.16	113.87	1.37
Gdf10	238.32	205.17	87.57	83.73	1.37
Klc3	101.16	140.73	54.47	39.07	1.37
Col16a1	685.78	679.93	252.04	276.87	1.37
Ctgf	1594.32	1142.82	460.29	599.51	1.37

Vat1	2716.86	2881.64	1127.75	1041.61	1.37
Cdc7	66.15	64.43	12.82	37.96	1.37
-	36.96	108.52	33.11	23.44	1.37
Timeless	310.30	287.40	134.56	97.13	1.37
Cd24a	6340.32	7553.82	2776.67	2625.81	1.36
Pde3a	315.17	356.07	122.81	138.44	1.36
Ttll7	97.27	91.56	24.56	49.12	1.36
Serpine2	742.20	891.03	315.04	322.64	1.36
Pi15	43.77	49.17	9.61	26.79	1.36
BC055324	46.69	45.78	14.95	21.21	1.36
Itih1	215.95	188.21	59.81	98.24	1.36
C2	674.11	726.56	268.06	280.22	1.35
Rtn4	5435.67	5024.01	2122.01	1973.82	1.35
Cdca8	179.96	234.84	75.82	87.08	1.35
Islr2	58.36	36.46	17.09	20.10	1.35
4833422C13Rik	40.86	59.35	14.95	24.56	1.35
Sult2b1	60.31	50.02	30.97	12.28	1.35
Tsc22d1	13312.92	12418.45	4555.87	5565.32	1.35
Cmtm7	286.96	329.79	111.07	131.74	1.35
Lyn	710.10	642.63	247.76	284.69	1.35
Cep192	448.43	401.85	182.62	151.83	1.35
S100a11	4395.81	4511.95	1790.95	1717.04	1.34
Plekha4	126.46	216.19	72.62	62.52	1.34
Tspan6	186.77	172.95	77.96	63.64	1.34
Myl9	1658.52	1240.32	595.92	545.93	1.34
Ifih1	581.70	509.52	246.70	183.09	1.34
Kcnab2	155.64	153.45	70.48	51.36	1.34
Hcn3	134.24	125.47	55.53	46.89	1.34
Mark1	136.18	132.26	51.26	54.70	1.34
Ets1	1300.55	1156.39	422.91	548.16	1.34
Adgrg6	147.86	91.56	60.87	33.49	1.34
Map7d1	1782.06	1966.03	717.66	764.74	1.34
Agrn	10515.32	8778.88	4115.87	3514.47	1.34
Mex3b	136.18	137.34	50.19	58.05	1.34
Chst1	136.18	247.56	88.64	63.64	1.33
Mab21l3	140.07	149.21	53.40	61.40	1.33
Loxl4	139.10	129.71	64.08	42.42	1.33
Stat2	1289.85	1169.10	462.42	513.55	1.33
Vcl	1823.89	1062.28	509.41	636.36	1.33
Slc1a2	249.99	233.14	99.32	92.66	1.33
Scarb1	835.58	935.11	348.15	356.14	1.33
Pola1	149.80	133.10	55.53	56.94	1.33
Cdh24	121.59	113.60	53.40	40.19	1.33
C330027C09Rik	137.16	122.93	51.26	52.47	1.33
Clic1	4012.55	4007.51	1726.87	1473.67	1.33
Gpsm1	316.14	344.20	113.20	150.72	1.32
Zfp429	104.08	108.52	49.13	35.73	1.32
Ubtd2	62.26	60.19	30.97	17.86	1.32
Rhbdl2	71.98	61.04	37.38	15.63	1.32
Jam2	429.95	336.57	131.36	175.28	1.32
Cttnbp2nl	1111.84	1028.37	449.61	406.37	1.32
Pald1	392.01	389.14	175.14	137.32	1.32
Fli1	285.01	264.51	95.05	125.04	1.32
Cavin3	298.63	265.36	114.27	111.64	1.32
Usp11	285.99	314.53	135.63	104.94	1.32
Bmf	480.53	740.12	201.84	288.03	1.32
Spns2	3327.74	2753.63	1031.64	1406.68	1.32
Btg1	2553.44	3252.98	1191.83	1136.51	1.32
Pcdhgb6	67.12	50.02	22.43	24.56	1.32
Clmp	115.76	96.65	38.45	46.89	1.32
Lca5l	45.72	38.15	21.36	12.28	1.31
Nnat	101.16	100.04	36.31	44.66	1.31

B3gnt3	213.03	209.40	89.71	80.38	1.31
Carhsp1	2377.38	2770.58	1080.76	995.84	1.31
Clec2d	1025.27	1191.15	416.50	478.94	1.31
Pla2g4a	338.51	297.57	140.97	116.11	1.31
-	1221.76	1434.46	349.22	725.67	1.31
Ypel2	1393.94	2087.26	588.44	821.68	1.30
Ckap2l	178.98	146.67	73.69	58.05	1.30
Raph1	678.97	601.08	256.31	262.36	1.30
Il18bp	214.00	247.56	110.00	77.03	1.30
-	341.43	591.76	155.92	223.28	1.30
Fkbp7	81.71	82.24	32.04	34.61	1.30
Smc2	269.45	267.90	96.12	122.81	1.30
Rbms3	296.69	197.54	124.95	75.92	1.30
Gimap8	485.40	445.09	186.89	192.02	1.30
S1pr3	473.72	369.64	155.92	187.56	1.30
Rela	2095.28	2280.56	852.22	931.09	1.30
Hmga1b	129.37	137.34	66.21	42.42	1.29
Tmem47	137.16	150.91	65.14	52.47	1.29
Bspry	3117.63	2593.39	1266.59	1067.29	1.29
Abca9	327.81	290.79	105.73	147.37	1.29
Eppk1	82.68	48.32	26.70	26.79	1.29
Gpr161	61.28	43.24	17.09	25.68	1.29
Gas2l3	113.81	103.43	30.97	58.05	1.29
Zfp991	50.58	59.35	13.88	31.26	1.29
Dapk2	169.26	178.04	57.67	84.85	1.29
Dchs1	327.81	298.42	115.34	141.78	1.28
Tcirg1	1570.97	1791.38	681.35	699.99	1.28
Mill2	158.56	113.60	43.79	68.10	1.28
Trim45	72.96	83.08	37.38	26.79	1.28
Trpv5	1497.05	791.84	473.10	468.89	1.28
Map4k4	2362.79	2213.58	898.14	986.91	1.28
Cd93	1227.60	909.68	426.11	454.38	1.28
Gbp9	399.80	363.70	114.27	200.95	1.28
Kif23	329.76	362.01	121.75	164.11	1.28
Tacc3	299.60	357.77	132.43	139.55	1.27
Eif4e3	315.17	300.97	146.31	108.29	1.27
Il17rb	547.65	486.63	191.16	236.68	1.27
Eif4ebp1	878.38	863.90	369.51	351.67	1.27
Mthfd2	338.51	338.27	150.58	129.50	1.27
Twf2	473.72	590.91	214.66	226.63	1.27
Dysf	955.23	775.73	363.10	353.90	1.27
Macc1	54.47	47.48	30.97	11.16	1.27
Ifngr1	1636.15	1704.91	608.73	777.02	1.27
Msrb3	408.55	339.96	143.11	167.46	1.27
Epb41l2	904.65	806.25	380.19	330.46	1.27
Tle6	72.96	107.67	39.51	35.73	1.27
AI467606	97.27	104.28	46.99	36.84	1.26
Myh9	16369.27	13043.27	6334.00	5910.30	1.26
Stard9	433.84	467.98	152.72	223.28	1.26
Paqr8	49.61	62.74	25.63	21.21	1.26
Pparg	68.09	50.02	22.43	26.79	1.26
Gjc1	153.69	152.60	57.67	70.33	1.26
Pde4a	274.31	402.70	148.44	135.09	1.26
Mfge8	3464.90	3816.76	1548.52	1504.93	1.25
Afap1l2	1088.50	1122.48	409.02	519.13	1.25
Mcm4	344.35	347.59	144.17	146.25	1.25
Sirpa	3220.74	3469.16	1286.88	1527.25	1.25
Cd34	1064.18	808.79	412.23	375.12	1.25
Csnk1e	670.22	678.23	291.55	275.75	1.25
Igf1	273.34	139.04	118.54	54.70	1.25
Apoe	10665.12	10970.42	4679.75	4435.51	1.25
Fam129a	1396.85	1171.65	519.02	563.79	1.25

Mamstr	50.58	50.02	25.63	16.75	1.25
Ifit3b	256.80	206.01	103.59	91.55	1.24
Tacstd2	2896.82	1638.78	1106.39	808.28	1.24
Fmnl2	508.74	454.42	189.03	217.70	1.24
Stom	424.11	406.94	156.99	194.26	1.24
H2-Q7	967.88	1219.97	426.11	499.04	1.24
Itpr3	692.59	643.47	307.57	256.78	1.24
Dtl	79.76	64.43	37.38	23.44	1.24
Paqr4	133.27	166.17	59.81	66.98	1.24
Enho	71.01	73.76	26.70	34.61	1.24
Mastl	98.25	89.87	35.24	44.66	1.24
Homer3	305.44	328.10	118.54	150.72	1.24
Pinx1	167.31	215.34	81.16	81.50	1.24
Slc7a1	706.21	500.20	279.80	232.21	1.23
Troap	82.68	89.87	29.90	43.54	1.23
Cck	77.82	64.43	49.13	11.16	1.23
Bcl6b	164.39	177.19	68.35	77.03	1.23
Prkar2b	81.71	51.72	30.97	25.68	1.23
Ggn	49.61	52.56	26.70	16.75	1.23
Evl	338.51	350.14	155.92	137.32	1.23
Antxr2	361.86	349.29	106.79	196.49	1.23
Gbp4	266.53	244.16	69.42	148.48	1.23
Helz2	1348.22	1203.86	526.50	561.56	1.23
Mthfd1l	190.66	165.32	105.73	45.77	1.23
Kif26b	341.43	367.09	147.38	155.18	1.23
Atp10a	90.46	78.00	39.51	32.38	1.23
Unc119	168.28	239.92	87.57	87.08	1.23
Dok4	656.60	676.54	283.01	286.92	1.23
Igtp	549.60	690.95	229.61	301.43	1.23
Kif22	210.11	245.86	74.76	120.57	1.23
Irgm1	672.16	712.99	289.41	303.66	1.22
9930012K11Rik	54.47	65.28	25.63	25.68	1.22
Slc43a1	50.58	41.54	16.02	23.44	1.22
Irgm2	949.39	987.68	346.02	484.52	1.22
Derl3	126.46	137.34	69.42	43.54	1.22
Gm10687	44.75	49.17	21.36	18.98	1.22
Itga8	296.69	285.71	120.68	129.50	1.22
Lig1	379.37	376.42	168.74	156.30	1.22
Cyp4v3	244.16	334.03	122.81	126.15	1.22
Ywhah	3409.45	3455.60	1472.70	1481.48	1.22
Ifit3	928.97	686.71	387.67	307.01	1.22
3110082I17Rik	127.43	105.97	50.19	50.24	1.22
Chst14	135.21	156.84	49.13	77.03	1.21
Csrnp1	376.45	377.27	169.80	155.18	1.21
Mylk	3901.66	2995.25	1337.07	1637.78	1.21
Ctps	1585.57	1069.06	545.72	601.75	1.21
Ppl	589.48	487.48	276.60	188.67	1.21
Samhd1	1282.07	1232.69	548.93	539.23	1.21
Agpat4	148.83	175.49	81.16	59.17	1.21
Ltk	57.39	61.04	26.70	24.56	1.21
2310022B05Rik	1929.92	1865.99	731.54	914.34	1.21
Aplp1	326.84	429.83	167.67	160.76	1.21
Timd2	2383.21	2609.50	1039.11	1132.04	1.20
Arhgap33	264.59	291.64	130.29	111.64	1.20
Ier2	532.09	620.58	262.72	238.91	1.20
Arhgap4	291.82	283.16	147.38	102.71	1.20
Tcaf2	165.37	135.65	61.94	69.22	1.20
Creb3l1	270.42	228.90	95.05	122.81	1.20
Atp8a1	587.53	406.09	250.97	181.98	1.20
Fjx1	338.51	445.09	155.92	186.44	1.20
Lilra5	81.71	75.45	40.58	27.91	1.20
Dnm1	159.53	144.12	54.47	78.15	1.20

Ccdc120	463.02	515.46	225.34	202.07	1.20
Entpd1	579.75	482.39	186.89	277.99	1.19
Meis1	175.09	162.78	57.67	90.43	1.19
Ogn	319.06	295.03	113.20	156.30	1.19
Nin	415.36	297.57	145.24	167.46	1.19
Dennd2c	66.15	55.95	21.36	32.38	1.18
Map3k14	686.75	742.67	278.73	350.55	1.18
B130024G19Rik	263.61	233.99	91.84	127.27	1.18
Mex3d	374.50	379.81	159.12	173.04	1.18
2310043M15Rik	52.53	44.09	22.43	20.10	1.18
Gmfg	169.26	189.06	86.50	71.45	1.18
Tcaf1	935.78	924.94	366.31	454.38	1.18
Flt4	483.45	414.57	179.42	216.58	1.18
Gm16008	62.26	78.84	27.77	34.61	1.18
Nrep	301.55	314.53	156.99	114.99	1.18
Rassf2	302.52	300.12	134.56	131.74	1.18
Pnrc1	1982.44	3336.91	1017.75	1334.11	1.18
Lrrc49	135.21	128.02	71.55	44.66	1.18
Slc12a4	1052.50	995.31	470.97	434.29	1.18
Dpysl2	242.21	327.25	127.09	125.04	1.18
Tgfbr2	3871.50	3267.39	1554.93	1603.17	1.18
Actb	18512.21	15287.37	8713.39	6240.75	1.18
Gpr4	134.24	149.21	61.94	63.64	1.18
Iffo2	292.79	291.64	117.47	141.78	1.17
Muc1	1071.96	1067.37	638.63	310.36	1.17
Arhgap11a	392.01	349.29	175.14	154.07	1.17
Gm7665	217.89	200.93	105.73	80.38	1.17
Gen1	73.93	76.30	24.56	42.42	1.17
Plscr3	1009.70	1073.30	464.56	463.31	1.17
Ets2	2210.06	2595.94	1008.14	1133.16	1.17
Maoa	405.63	424.74	187.96	181.98	1.17
Ehd2	1259.70	1082.63	574.56	468.89	1.17
Clec11a	106.03	105.13	39.51	54.70	1.17
Parp8	868.66	678.23	414.36	274.64	1.17
Plxna3	146.88	158.54	73.69	62.52	1.16
Serpinh1	4464.88	4365.28	1934.05	2006.20	1.16
Hmmr	191.63	200.08	80.10	94.90	1.16
Sh2d4b	93.38	76.30	25.63	50.24	1.16
Sema6a	738.31	863.90	317.18	399.68	1.16
Pced1b	107.97	79.69	49.13	34.61	1.16
Adamts14	97.27	37.30	30.97	29.03	1.16
Fabp4	1247.05	1127.56	446.40	616.26	1.16
Mapk7	646.87	592.61	307.57	246.73	1.16
Csf1r	1931.86	2166.11	818.05	1017.05	1.16
Tor3a	759.71	729.10	312.91	353.90	1.16
Mis18a	138.13	150.91	73.69	55.82	1.16
Arrdc4	680.92	941.90	310.77	417.54	1.16
Rhbdl3	169.26	239.08	92.91	90.43	1.16
Lgals3bp	6933.69	7330.00	3025.50	3373.80	1.16
2700081O15Rik	856.01	790.14	328.93	409.72	1.16
Fhod1	955.23	1194.54	461.35	504.62	1.15
Flt3l	134.24	178.88	67.28	73.68	1.15
Trim12a	370.61	318.77	160.19	149.60	1.15
Mapk6	1455.22	1425.98	611.93	684.36	1.15
Maged2	771.38	724.86	375.92	296.97	1.15
Zbtb7c	211.08	186.51	93.98	84.85	1.15
Ssbp4	1534.98	1737.97	731.54	743.53	1.15
Inha	66.15	78.84	32.04	33.49	1.15
Cyp4f16	411.47	395.07	189.03	175.28	1.15
Trim12c	403.69	316.23	197.57	127.27	1.15
Cep112	66.15	61.89	28.83	29.03	1.15
4-Sep	902.70	924.94	506.21	320.41	1.14

Kifc1	214.98	209.40	93.98	98.24	1.14
Sbsn	118.67	98.34	48.06	50.24	1.14
Tmem140	331.70	264.51	122.81	147.37	1.14
Klhl25	482.48	549.37	201.84	266.82	1.14
Nes	1516.50	1314.92	724.07	560.44	1.14
Crispld2	436.76	796.08	215.73	344.97	1.14
Mmp17	120.62	131.41	57.67	56.94	1.14
Rab13	332.68	345.90	162.33	146.25	1.14
11-Sep	2732.43	2510.31	1172.61	1213.54	1.14
Nptxr	110.89	89.87	53.40	37.96	1.13
Hnrnpu	180.93	448.48	135.63	151.83	1.13
Rhog	815.16	867.29	407.96	359.49	1.13
Fat4	182.88	124.63	57.67	82.61	1.13
Stbd1	479.56	448.48	213.59	209.89	1.13
Gucy1b1	247.08	209.40	79.03	129.50	1.13
Abcg1	391.04	289.94	194.37	116.11	1.13
3110043O21Rik	345.32	376.42	163.40	166.35	1.13
Peak1	927.99	777.42	346.02	433.17	1.13
Slc38a2	3321.91	2855.36	1371.25	1451.34	1.13
Rbm43	326.84	378.11	149.51	173.04	1.13
Syngap1	530.14	651.10	263.78	276.87	1.13
Rnf180	59.34	50.87	20.29	30.14	1.13
Ccdc71l	568.08	580.74	263.78	263.47	1.12
Mtss1l	389.10	514.61	199.71	215.47	1.12
Diaph3	92.41	62.74	30.97	40.19	1.12
Tnfrsf10b	163.42	199.23	64.08	102.71	1.12
Gm18194	49.61	52.56	23.49	23.44	1.12
Pla2g5	198.44	195.84	90.78	90.43	1.12
Coro2a	295.71	274.68	151.65	110.52	1.12
Kcns3	110.89	97.50	51.26	44.66	1.12
Tedc1	106.03	121.23	53.40	51.36	1.12
Zfp202	124.51	100.89	50.19	53.59	1.12
Thbs1	2890.98	2456.90	1171.54	1293.92	1.12
Dtx4	1339.46	1359.86	482.71	762.51	1.12
Satb1	106.03	92.41	52.33	39.07	1.12
Trib1	924.10	1048.72	427.18	483.41	1.12
Cntln	174.12	159.38	79.03	74.80	1.12
Limd2	834.61	910.53	371.65	434.29	1.12
Tmem43	1014.57	1186.91	492.32	524.71	1.11
Gm10052	371.59	288.25	152.72	151.83	1.11
Vopp1	369.64	344.20	164.46	165.23	1.11
Plk2	350.19	386.59	143.11	197.61	1.11
Smim3	232.48	289.10	122.81	118.34	1.11
Ldb2	315.17	337.42	117.47	185.32	1.11
Ksr1	277.23	317.07	123.88	151.83	1.11
Atf5	573.92	651.95	288.35	280.22	1.11
Dyrk3	87.55	77.15	39.51	36.84	1.11
Hivep2	705.24	485.78	271.26	281.34	1.11
Klf7	230.54	153.45	84.37	93.78	1.11
Trim34a	202.33	234.84	89.71	113.87	1.10
Esco2	64.20	83.93	26.70	42.42	1.10
1-Mar	132.29	102.58	51.26	58.05	1.10
Ehd4	1932.83	1483.63	807.37	783.72	1.10
Hpgd	206.22	450.18	134.56	171.93	1.10
Cxadr	1355.03	1391.23	619.41	663.15	1.10
Vwa5a	1643.93	1521.79	686.69	792.65	1.10
Sdf2l1	432.87	503.59	242.42	195.37	1.10
Irf9	1170.21	1328.49	519.02	649.75	1.10
Cenpf	232.48	223.82	90.78	122.81	1.10
Cpm	950.37	936.81	387.67	495.69	1.10
Gnao1	74.90	77.15	28.83	42.42	1.10
Ssh2	711.07	724.01	287.28	385.16	1.09

Fam83d	79.76	76.30	38.45	34.61	1.09
Tmtc2	64.20	52.56	24.56	30.14	1.09
Tmem176b	29601.45	31844.73	14397.01	14407.32	1.09
Col7a1	188.71	199.23	101.46	80.38	1.09
Ezh2	343.38	313.68	148.44	159.65	1.09
Chaf1a	151.75	136.49	72.62	62.52	1.09
Klf11	323.92	416.27	132.43	215.47	1.09
Snhg5	147.86	148.36	57.67	81.50	1.09
Znrf1	1232.46	1402.25	663.20	573.84	1.09
C1qtnf2	72.96	87.32	37.38	37.96	1.09
Myo9b	1731.48	1668.45	760.38	837.31	1.09
Sned1	651.74	463.74	266.99	256.78	1.09
Dbf4	191.63	188.21	86.50	92.66	1.08
Hid1	125.48	149.21	70.48	59.17	1.08
Fyn	416.33	430.68	164.46	235.56	1.08
Cdca5	78.79	84.78	46.99	30.14	1.08
2810474O19Rik	1050.56	902.05	452.81	468.89	1.08
Phlda1	107.97	136.49	60.87	54.70	1.08
Irf5	528.20	471.37	242.42	229.98	1.08
Fbxo2	62.26	122.08	38.45	49.12	1.08
5730559C18Rik	947.45	1172.50	468.83	537.00	1.08
Galnt12	124.51	130.56	66.21	54.70	1.08
Kcnh2	148.83	170.41	81.16	70.33	1.08
Pdgfa	588.51	752.84	327.86	309.25	1.07
H2-Q10	68.09	86.47	26.70	46.89	1.07
Fxyd1	134.24	144.12	58.74	73.68	1.07
Cebpb	1175.07	1429.38	558.54	683.25	1.07
Fchsd1	334.62	424.74	155.92	206.54	1.07
Aida	658.54	716.38	337.47	318.18	1.07
Eprn	507.77	498.50	247.76	232.21	1.07
Fmnl3	1099.20	1148.76	573.49	499.04	1.07
Cdk6	1272.34	947.83	456.01	603.98	1.07
Prickle2	199.41	164.47	83.30	90.43	1.07
Rnasel	304.47	269.60	123.88	150.72	1.06
Sh3bp2	796.67	795.23	336.40	425.35	1.06
Irf8	685.78	807.10	350.29	365.07	1.06
Elk3	629.36	587.52	263.78	319.29	1.06
Map1b	270.42	173.80	112.13	100.48	1.06
Fam131a	114.78	97.50	43.79	58.05	1.06
Car11	94.36	139.04	64.08	48.01	1.06
Taok3	824.88	913.92	427.18	407.49	1.06
Brip1	60.31	71.21	35.24	27.91	1.06
Pole	178.98	91.56	70.48	59.17	1.06
P2ry14	77.82	73.76	44.85	27.91	1.06
Hnrnpa1	4408.46	4192.33	2172.21	1962.66	1.06
Phldb1	2363.76	2197.47	1172.61	1021.52	1.06
Bcl6	561.27	558.69	306.50	232.21	1.06
Cdc20	398.82	403.55	165.53	221.05	1.05
Frzb	535.01	500.20	285.14	213.24	1.05
Wasf1	62.26	91.56	36.31	37.96	1.05
C1qtnf7	107.97	139.89	73.69	45.77	1.05
Gm9791	110.89	108.52	56.60	49.12	1.05
Gsap	385.20	335.73	189.03	158.53	1.05
Ifnar2	702.32	719.77	369.51	318.18	1.05
-	513.61	523.93	226.41	275.75	1.05
Ccnd2	479.56	385.75	171.94	246.73	1.05
Bora	123.54	106.82	54.47	56.94	1.05
Bhlhe40	3147.79	3727.74	1546.39	1782.91	1.05
Slc7a6	275.29	260.27	112.13	147.37	1.05
Chml	106.03	133.10	45.92	70.33	1.04
Ctsd	16527.82	16967.69	8378.05	7875.18	1.04
Gngt2	170.23	148.36	81.16	73.68	1.04

Ampd3	1223.71	1293.73	577.76	647.52	1.04
Zc3h12a	214.98	297.57	127.09	122.81	1.04
Rassf4	367.70	445.94	136.70	260.12	1.04
Hs6st2	163.42	109.37	48.06	84.85	1.04
Tmem184b	1335.57	1327.64	702.71	595.05	1.04
Irak4	456.22	385.75	203.98	206.54	1.04
Map3k12	298.63	406.94	184.76	159.65	1.04
Gm8186	167.31	161.08	82.23	78.15	1.03
Gpx8	288.90	267.05	142.04	129.50	1.03
Plekhh2	199.41	162.78	86.50	90.43	1.03
Ncapd2	582.67	651.10	308.64	294.73	1.03
Lmo2	544.73	373.03	255.24	193.14	1.03
Mms22l	111.87	108.52	60.87	46.89	1.03
Tbc1d9	325.87	309.44	161.26	149.60	1.03
Birc2	1321.95	1188.60	571.35	657.57	1.03
-	73.93	79.69	40.58	34.61	1.03
Cib2	90.46	109.37	57.67	40.19	1.03
Hells	86.57	55.95	39.51	30.14	1.03
Rhoc	2813.16	3225.85	1373.38	1588.66	1.03
Frk	629.36	558.69	304.37	277.99	1.03
1700017B05Rik	926.05	947.83	429.32	490.11	1.03
-	983.44	723.17	292.62	545.93	1.03
Pprc1	780.14	709.60	350.29	381.81	1.02
Il4ra	1174.10	1036.85	523.29	566.02	1.02
Chaf1b	113.81	153.45	72.62	59.17	1.02
A4galt	906.59	1157.24	525.43	492.34	1.02
Fat1	3394.86	2723.95	1562.41	1453.57	1.02
Cxcr4	103.11	118.69	46.99	62.52	1.02
Serinc2	946.48	997.00	531.84	426.47	1.02
Arid5b	1475.65	1316.62	618.34	759.16	1.02
Fam171a2	470.81	489.18	293.69	179.74	1.02
Tmem132a	418.28	445.94	212.52	214.35	1.02
Dmpk	524.31	635.00	305.43	267.94	1.02
Cyp4f17	432.87	548.52	257.38	228.86	1.01
Fbln7	208.17	234.84	133.49	85.96	1.01
Jund	191.63	217.88	100.39	102.71	1.01
Rsl1	130.35	113.60	69.42	51.36	1.01
Slc6a15	907.57	894.42	390.87	503.50	1.01
Trim46	228.59	261.12	159.12	83.73	1.01
Mad2l1	251.94	290.79	136.70	132.85	1.01
Rfx5	599.21	639.23	326.79	288.03	1.01
Tnfrsf1a	2785.93	2620.52	1312.51	1373.19	1.01
Tmbim1	1194.52	1299.66	578.83	662.03	1.01
Psd	312.25	355.22	159.12	173.04	1.01
Itm2c	3175.02	3104.61	1598.72	1527.25	1.01
Plec	4891.91	4514.49	2407.16	2276.37	1.01
Lmod1	153.69	107.67	57.67	72.57	1.00
Apold1	83.66	69.52	40.58	35.73	1.00
Tnfrsf18	67.12	57.65	30.97	31.26	1.00
Ahnak	4084.53	4015.14	1960.75	2083.23	1.00
Parp12	1032.08	990.22	472.03	538.11	1.00
Dzip3	202.33	163.62	77.96	104.94	1.00
Slc16a11	323.92	354.38	667.47	689.94	-1.00
AL589670.1	39.88	51.72	86.50	97.13	-1.00
Slc7a7	2250.92	1754.93	4453.34	3566.94	-1.00
Slc25a3	9916.11	9318.92	19997.33	18551.45	-1.00
Acad10	1089.47	1014.81	2052.60	2164.73	-1.00
Cyp2j13	1222.73	1247.95	2510.75	2442.71	-1.00
Synpo2l	40.86	42.39	82.23	84.85	-1.00
Gnaz	64.20	40.69	122.81	87.08	-1.01
Slc25a12	1056.40	1023.28	2092.11	2085.46	-1.01
Ndufa3	1290.83	1218.28	2601.52	2438.25	-1.01

Atp6v1a	5497.93	4861.24	10054.73	10761.11	-1.01
Fxn	210.11	183.12	413.30	376.23	-1.01
Pgam1	2406.56	2051.65	4530.24	4427.70	-1.01
Pde9a	359.91	384.90	755.04	744.65	-1.01
Slc19a3	252.91	286.55	476.30	610.68	-1.01
Gm5617	238.32	233.99	551.06	399.68	-1.01
Capn6	51.56	55.11	103.59	111.64	-1.01
Cmah	373.53	285.71	680.28	647.52	-1.01
Slco1a1	403.69	462.05	743.29	1007.01	-1.01
Igfbp1	46.69	111.91	140.97	180.86	-1.01
Wscd1	77.82	95.80	206.11	145.13	-1.02
Vnn1	1578.76	1425.98	2916.57	3162.80	-1.02
Bend5	302.52	222.97	603.39	458.85	-1.02
Rhov	106.03	128.02	218.93	255.66	-1.02
Cox10	236.38	244.16	497.66	475.59	-1.02
Prdx3	1796.65	1843.10	3684.42	3687.52	-1.02
Slit2	404.66	450.18	846.88	886.43	-1.02
Mpp3	33.07	47.48	83.30	80.38	-1.02
Gbe1	390.07	418.81	790.28	850.71	-1.02
Coq3	186.77	195.84	379.12	397.44	-1.02
Ftcd	245.13	194.14	458.15	432.05	-1.02
Fkbp5	1513.58	1752.38	3445.20	3184.01	-1.02
Gm10031	178.98	159.38	355.63	330.46	-1.02
Gpr137b-ps	697.45	761.32	1484.45	1477.02	-1.02
Mfn2	1794.71	1645.56	3664.13	3325.80	-1.02
Zfp385b	58.36	39.00	102.52	94.90	-1.02
Shmt1	871.57	990.22	2024.83	1762.82	-1.02
Ndufs4	881.30	887.64	1839.01	1759.47	-1.02
Mrps35	562.24	604.48	1190.76	1183.40	-1.02
Gatsl2	1180.91	1174.19	2503.27	2289.77	-1.03
Idi1	139.10	135.65	240.29	319.29	-1.03
Pcsk4	130.35	158.54	297.96	291.38	-1.03
Mturn	1497.05	1394.62	2925.11	2968.54	-1.03
Bdnf	102.14	98.34	196.50	212.12	-1.03
Gm5559	66.15	75.45	151.65	137.32	-1.03
Gm6768	220.81	165.32	357.76	428.70	-1.03
Mdk	624.50	670.60	1502.60	1139.86	-1.03
Stard8	1137.13	1097.04	2411.43	2146.86	-1.03
Syt17	177.04	172.10	363.10	349.44	-1.03
Ftl1-ps1	551.54	545.98	1312.51	927.74	-1.03
Acot13	815.16	757.08	1797.36	1412.26	-1.03
Tmem139	126.46	155.15	268.06	308.13	-1.03
Acp6	782.08	809.64	1637.16	1615.45	-1.03
Cyp4a31	4932.76	4537.38	8383.39	10966.53	-1.03
Mrpl4	1035.00	996.15	2214.92	1935.86	-1.03
6430571L13Rik	47.66	72.91	112.13	135.09	-1.03
Smdt1	1438.68	1323.40	3156.86	2490.72	-1.03
Afmid	196.49	165.32	389.80	349.44	-1.03
Mrpl16	350.19	415.42	736.88	830.61	-1.03
Acot6	227.62	241.62	432.52	528.06	-1.03
Hmgcs1	3350.12	2660.37	6826.33	5470.43	-1.03
Grsf1	1468.84	1536.20	2984.92	3166.15	-1.03
Sgk1	1534.98	1231.84	3375.78	2288.65	-1.03
Gm10250	435.79	398.46	915.23	792.65	-1.03
Mrps36	400.77	379.81	844.75	753.58	-1.03
Proz	53.50	43.24	117.47	80.38	-1.04
Slc7a9	1659.49	1330.18	3374.72	2750.84	-1.04
Frs3	153.69	174.64	360.97	312.60	-1.04
Adk	1136.16	1201.32	2103.86	2691.67	-1.04
Decr1	2324.85	2179.67	4549.46	4691.17	-1.04
Hagh	1950.34	1988.07	4184.22	3896.28	-1.04
Gstt3	410.50	583.28	1119.21	922.16	-1.04

Treh	919.24	967.33	1952.21	1922.46	-1.04
Zfp768	477.62	432.37	1007.08	860.76	-1.04
AI661453	385.20	332.33	831.93	640.82	-1.04
Ptges2	674.11	622.28	1388.33	1273.83	-1.04
Timm8a1	144.94	162.78	324.66	308.13	-1.04
Slc9a8	1230.52	1010.57	2742.49	1863.29	-1.04
Park2	69.06	68.67	127.09	156.30	-1.04
Lrrc31	56.42	35.61	97.18	91.55	-1.04
Vegfa	2725.62	2702.76	5907.89	5261.66	-1.04
Dnaja3	1213.98	1223.36	2573.76	2441.60	-1.04
Grpel1	982.47	1105.52	2161.53	2137.93	-1.04
Mtor	2558.31	2346.69	4675.48	5426.89	-1.04
Auh	815.16	858.81	1767.45	1681.32	-1.04
Pip5k1b	42.80	76.30	106.79	139.55	-1.04
Tpmt	632.28	697.73	1415.03	1326.30	-1.04
Clpx	1548.60	1800.71	2668.80	4234.56	-1.04
Tlcd1	527.23	541.74	1145.91	1057.24	-1.04
Slc5a10	2228.55	2548.46	4866.64	4983.67	-1.04
Aoah	642.01	577.35	1228.14	1284.99	-1.04
Slc2a13	68.09	61.04	140.97	125.04	-1.04
Igfbp4	12204.97	10972.11	26286.48	21503.25	-1.04
Ncoa4	1065.15	890.18	1855.03	2179.24	-1.05
Fhl1	1976.61	1905.83	3869.18	4148.59	-1.05
mt-Nd3	210.11	239.08	441.06	487.87	-1.05
Tmem120a	795.70	815.58	1680.95	1650.06	-1.05
Pdhb	1680.89	1756.62	3707.92	3402.83	-1.05
Aldh2	7378.23	6905.26	15112.54	14435.23	-1.05
Gsta4	265.56	232.29	569.22	459.96	-1.05
Pxmp4	2051.51	2356.01	4059.27	5062.94	-1.05
Ndufb2	170.23	134.80	304.37	325.99	-1.05
Hspa9	6824.74	6060.01	13451.88	13226.16	-1.05
Reep6	592.40	679.08	1113.87	1520.56	-1.05
Ggact	601.15	693.49	1360.57	1322.95	-1.05
Spc25	284.04	350.99	676.01	641.94	-1.05
Dhfr	392.01	414.57	823.39	849.59	-1.05
Shroom1	43.77	39.00	83.30	88.20	-1.05
Stk32b	192.60	183.12	374.85	404.14	-1.05
Por	3477.55	3146.15	7210.79	6532.14	-1.05
Mfsd4b1	1550.55	1526.02	3068.22	3316.87	-1.05
Pdzk1ip1	5339.37	4031.25	10581.23	8869.91	-1.05
Depdc7	256.80	213.64	461.35	514.67	-1.05
Grip2	66.15	103.43	160.19	193.14	-1.05
Cpt1a	4826.74	6385.56	9783.47	13529.82	-1.06
Gm11767	37.94	40.69	85.44	78.15	-1.06
Mto1	249.99	243.32	541.45	484.52	-1.06
Tmem86a	346.30	402.70	789.21	770.33	-1.06
Cpne4	36.96	30.52	85.44	54.70	-1.06
Pgk1	4937.63	4786.63	10401.82	9835.61	-1.06
Smpd2	833.64	935.11	1963.96	1718.16	-1.06
Utp20	817.10	950.37	1504.74	2175.89	-1.06
Chst7	322.95	258.58	676.01	533.65	-1.06
Gss	2676.01	2459.44	5734.88	4961.34	-1.06
Abcb8	746.09	729.10	1708.72	1364.26	-1.06
2310010J17Rik	54.47	33.91	112.13	71.45	-1.06
Ndufaf6	189.68	170.41	409.02	341.62	-1.06
Rhpn1	428.01	499.35	970.76	965.70	-1.06
BC022687	56.42	62.74	121.75	127.27	-1.06
Abcc2	4189.59	3142.76	7154.19	8162.10	-1.06
Zc2hc1c	100.19	132.26	242.42	244.49	-1.06
Tmem189	1894.90	2044.87	4172.47	4070.45	-1.06
Tkfc	4412.35	4106.70	8459.22	9363.36	-1.07
Bnip3	881.30	1024.98	1936.19	2054.20	-1.07

Asb9	271.39	278.08	555.33	595.05	-1.07
Cdo1	1604.05	1978.74	3544.52	3957.69	-1.07
Mfap3l	886.17	768.95	1696.97	1766.17	-1.07
Slc17a1	4774.21	4177.92	8813.78	9928.27	-1.07
Mogat1	704.26	707.06	1499.40	1455.80	-1.07
9430091E24Rik	71.01	57.65	121.75	147.37	-1.07
Etfdh	1792.76	2216.97	3922.57	4482.40	-1.07
Ak3	2150.73	2136.43	4566.55	4417.65	-1.07
Gfra1	41.83	64.43	70.48	152.95	-1.07
Efcab12	61.28	69.52	149.51	125.04	-1.07
Cntfr	72.96	99.19	187.96	174.16	-1.07
Tmem205	1015.54	1080.93	2268.32	2134.58	-1.07
Hpn	4956.11	4850.21	10728.61	9863.52	-1.07
Frg2f1	49.61	65.28	131.36	110.52	-1.07
Pdzk1	4530.05	3319.95	9592.31	6906.14	-1.07
Ndufa2	1547.63	1555.70	3600.05	2923.89	-1.07
Higd2a	1168.26	1241.17	2747.83	2319.91	-1.07
Slc25a11	1802.49	1717.63	4022.96	3382.73	-1.07
Mpc1	310.30	287.40	664.26	592.82	-1.07
-	120.62	79.69	175.14	245.61	-1.07
Poldip2	1032.08	1003.78	2276.87	2007.31	-1.07
Afg1l	275.29	315.38	731.54	512.43	-1.07
Atxn7l2	474.70	480.70	1116.01	895.36	-1.07
Gpm6a	856.98	836.77	1649.98	1918.00	-1.07
Larp1b	571.00	594.30	1223.87	1231.40	-1.07
Gm13910	601.15	530.72	1150.18	1233.64	-1.08
Sephs2	2442.55	2439.94	5696.44	4598.51	-1.08
Sowaha	40.86	44.93	95.05	85.96	-1.08
-	137.16	142.43	286.21	303.66	-1.08
1810021B22Rik	25.29	33.06	61.94	61.40	-1.08
Kcnj16	5871.46	4785.78	10557.74	11922.19	-1.08
Gm15417	41.83	78.00	142.04	111.64	-1.08
Paqr7	599.21	641.78	1283.67	1336.35	-1.08
Pnkd	1061.26	1134.34	2391.14	2243.99	-1.08
Folr1	4246.01	4135.53	9107.46	8591.92	-1.08
Ddc	753.87	1041.94	1487.65	2308.74	-1.08
Ptpdc1	393.96	430.68	902.42	840.66	-1.08
Pdss2	173.15	161.08	333.20	372.88	-1.08
Alkbh7	207.19	195.84	473.10	378.46	-1.08
Me3	471.78	402.70	961.15	886.43	-1.08
Trap1	2586.52	2472.16	5338.67	5368.84	-1.08
Pfkm	599.21	690.10	1581.63	1148.79	-1.08
Dlst	4271.30	4081.27	9197.17	8493.68	-1.08
Prps2	558.35	540.04	1277.27	1049.43	-1.08
Fpgs	926.05	953.77	1742.89	2241.76	-1.08
Mrpl28	683.84	831.68	1825.12	1388.82	-1.08
Tmem254c	26.26	26.28	52.33	59.17	-1.09
Gm33023	67.12	52.56	148.44	104.94	-1.09
Trappc9	706.21	786.75	1607.26	1561.86	-1.09
Slc25a15	1689.65	1972.81	3629.96	4147.48	-1.09
Ndufaf4	409.52	437.46	928.05	871.92	-1.09
Tmem106a	1374.48	1266.60	2984.92	2625.81	-1.09
Fads2	1133.24	939.35	2175.41	2228.36	-1.09
Entpd5	4688.61	4486.51	9443.87	10063.36	-1.09
Ankrd13c	2294.69	1929.57	4745.96	4241.26	-1.09
Smim1	716.91	726.56	1605.13	1469.20	-1.09
Bola3	520.42	484.94	1113.87	1027.10	-1.09
Eci1	1215.92	1153.00	2610.07	2436.02	-1.09
Gm10658	35.02	29.67	66.21	71.45	-1.09
Tmlhe	185.79	178.04	359.90	415.31	-1.09
Ctf1	85.60	93.26	237.08	144.02	-1.09
Fuca2	4308.26	4180.46	8815.91	9288.56	-1.09

Iah1	1429.93	1447.18	3210.25	2926.12	-1.09
Hadhb	1744.12	1877.01	3918.30	3806.97	-1.09
Pctp	392.01	434.92	941.93	822.80	-1.09
Iqgap2	827.80	689.25	1580.56	1656.76	-1.09
Rnf181	80.74	78.00	184.76	154.07	-1.09
Gm13075	66.15	73.76	178.35	120.57	-1.10
C730027H18Rik	99.22	77.15	200.77	175.28	-1.10
Pipox	3021.33	2903.69	6389.53	6267.55	-1.10
Gjb2	780.14	774.03	1406.49	1914.65	-1.10
Xylt1	241.24	164.47	479.51	386.28	-1.10
Ndufb8	1908.52	1763.41	4409.56	3441.90	-1.10
Apob	1893.92	1722.71	3332.00	4403.14	-1.10
Apoh	114.78	184.82	191.16	451.03	-1.10
Itpr2	1011.65	809.64	2040.85	1854.36	-1.10
Gm9922	36.96	45.78	103.59	73.68	-1.10
Mgat3	1422.15	1156.39	2978.51	2539.84	-1.10
Dusp28	155.64	162.78	373.78	308.13	-1.10
Polr3e	815.16	788.45	1615.81	1819.75	-1.10
Ppargc1a	737.34	567.17	1511.15	1283.88	-1.10
Nr1d1	294.74	329.79	442.13	898.71	-1.10
Flvcr2	277.23	280.62	596.98	600.63	-1.10
-	22.37	29.67	68.35	43.54	-1.10
Ndufb3	813.21	834.23	1910.56	1628.85	-1.10
Gas2	1913.38	1549.76	3695.10	3743.34	-1.10
Tsku	249.02	252.64	419.70	658.68	-1.10
Crocc	1017.49	1058.04	2468.03	1993.92	-1.10
Ppif	621.58	545.98	1293.29	1215.77	-1.10
Mrpl34	655.63	669.76	1584.84	1268.25	-1.11
Slc25a4	3929.87	3737.06	9352.02	7159.56	-1.11
Ttc38	657.57	601.93	1491.92	1220.24	-1.11
Sult1b1	35.99	37.30	84.37	73.68	-1.11
Chchd2	5412.32	5283.44	12004.81	11058.08	-1.11
Galm	1060.29	956.31	2267.25	2081.00	-1.11
Trpm6	202.33	157.69	349.22	426.47	-1.11
Enpp6	1106.98	913.07	2232.01	2123.42	-1.11
Phb	1960.07	1976.20	4524.90	3969.97	-1.11
Mcee	378.40	395.07	900.28	769.21	-1.11
Fuca1	2447.41	2461.99	5631.29	4964.69	-1.11
Slc25a17	977.60	1188.60	2299.29	2381.31	-1.11
Tprkb	1194.52	1241.17	2744.63	2517.51	-1.11
Spr	1530.12	1514.16	3704.71	2875.88	-1.11
Gm18588	119.65	83.08	229.61	207.65	-1.11
Cyp27a1	675.08	813.03	1632.89	1588.66	-1.11
Atp5j	2234.38	2130.50	5028.97	4417.65	-1.11
Mrps34	326.84	357.77	836.20	646.40	-1.11
Aamdc	276.26	298.42	664.26	580.54	-1.11
Pdk1	551.54	594.30	1377.65	1104.13	-1.11
Gcgr	400.77	507.83	971.83	998.07	-1.11
Erich4	479.56	448.48	1076.49	933.32	-1.12
Hnmt	142.02	107.67	271.26	269.06	-1.12
Sigirr	1151.72	1171.65	2796.96	2241.76	-1.12
D2hgdh	398.82	437.46	912.03	903.18	-1.12
L3hypdh	292.79	337.42	668.54	701.11	-1.12
Slc27a2	27789.23	24284.98	54256.04	58858.46	-1.12
Ugt1a7c	1689.65	1550.61	3437.73	3602.67	-1.12
Fam222a	72.96	117.00	195.43	218.82	-1.12
Me1	3344.28	3262.30	6562.54	7803.73	-1.12
Vdr	2488.27	2500.98	5583.23	5275.06	-1.12
Msra	2668.23	2609.50	6032.84	5454.80	-1.12
Kyat1	1053.48	1096.19	2513.95	2165.84	-1.12
Tysnd1	838.50	825.75	2063.28	1561.86	-1.12
Ccdc27	25.29	27.13	66.21	48.01	-1.12

Fmo1	4665.26	3759.95	9226.01	9133.38	-1.12
Idh3a	1615.72	1378.51	3442.00	3084.65	-1.12
Gm13655	35.02	37.30	87.57	70.33	-1.13
Gjb1	547.65	586.67	1248.43	1230.29	-1.13
Clpb	720.80	715.54	1581.63	1556.28	-1.13
Aifm3	198.44	198.38	480.58	386.28	-1.13
Fmod	61.28	68.67	155.92	128.39	-1.13
Napsa	11986.10	14499.77	28826.06	29121.66	-1.13
Igfbp5	3635.13	2119.48	7143.51	5445.87	-1.13
AI464131	470.81	728.25	1155.52	1471.43	-1.13
Tstd1	62.26	97.50	194.37	156.30	-1.13
H2afj	1306.39	1353.07	3053.26	2776.52	-1.13
Plekha7	1018.46	794.38	2136.96	1836.50	-1.13
Ndufb7	1170.21	1077.54	2799.09	2134.58	-1.13
Osbpl1a	770.41	748.60	1639.30	1698.07	-1.14
Tmem8	3172.10	2706.15	6764.38	6149.21	-1.14
-	47.66	51.72	102.52	116.11	-1.14
Dlec1	809.32	768.10	1949.01	1518.32	-1.14
Sdhd	2460.06	2449.27	5665.47	5131.04	-1.14
Acad8	728.58	751.99	1658.52	1598.70	-1.14
Ppil6	60.31	89.02	119.61	209.89	-1.14
Atp5h	2166.29	2177.13	5136.83	4421.00	-1.14
Nit2	784.03	697.73	1726.87	1532.84	-1.14
Ghitm	5419.13	5216.46	11835.00	11572.75	-1.14
Gm12338	253.89	280.62	622.61	555.97	-1.14
Tmem143	407.58	415.42	1014.55	799.35	-1.14
Snx29	682.86	462.89	1230.28	1292.81	-1.14
Got2	3341.36	3012.20	7487.39	6525.44	-1.14
Hoga1	1622.53	1644.72	3655.59	3556.90	-1.14
Gm17029	35.99	29.67	82.23	62.52	-1.14
Impa2	182.88	188.21	454.95	365.07	-1.14
Mfsd9	100.19	106.82	218.93	238.91	-1.14
Slc25a13	1174.10	939.35	2475.50	2197.10	-1.15
Pepd	2326.79	2245.80	5379.26	4735.83	-1.15
Slc5a9	412.44	290.79	839.41	714.50	-1.15
Cox7c	2868.61	2773.97	6529.44	5957.19	-1.15
Fdx1	623.53	612.95	1441.73	1298.39	-1.15
-	25.29	18.65	48.06	49.12	-1.15
Rtn4ip1	427.03	396.77	995.33	830.61	-1.15
Slc46a1	396.88	399.31	886.40	880.85	-1.15
Mpped1	36.96	50.87	96.12	99.36	-1.15
Scrn3	331.70	338.27	783.87	704.46	-1.15
Dpf3	26.26	27.98	45.92	74.80	-1.15
Fech	1469.81	1476.85	3012.68	3539.03	-1.15
Anapc13	645.90	640.08	1570.95	1288.34	-1.15
Oxsm	341.43	300.12	711.25	714.50	-1.15
Ndufs5	791.81	808.79	1951.14	1608.75	-1.15
Gm10736	78.79	66.13	215.73	106.06	-1.15
Tef	2908.49	1659.98	6503.80	3661.84	-1.15
6430573F11Rik	35.99	36.46	87.57	73.68	-1.15
Gm9755	26.26	33.06	59.81	72.57	-1.15
Iscu	1741.20	1776.97	4073.15	3760.08	-1.15
Kbtbd11	401.74	515.46	1045.52	999.19	-1.16
Fut9	1889.06	1175.04	3845.68	2976.36	-1.16
Alms1-ps2	29.18	24.59	71.55	48.01	-1.16
Amn	2362.79	2523.03	5935.66	4950.18	-1.16
Mgst3	1054.45	972.42	2452.01	2063.13	-1.16
Nsdhl	385.20	418.81	893.87	898.71	-1.16
-	166.34	152.60	362.03	348.32	-1.16
Isoc2b	195.52	150.06	461.35	308.13	-1.16
Rap1gap2	657.57	656.19	1663.86	1266.01	-1.16
Irs1	110.89	72.06	209.32	197.61	-1.16

Ndufa12	385.20	406.09	1034.84	731.25	-1.16
Nox4	2554.41	1885.49	5266.05	4648.75	-1.16
Aldh3b3	38.91	34.76	92.91	71.45	-1.16
Sycp3	44.75	32.22	82.23	89.31	-1.16
Cyp2u1	61.28	63.58	119.61	159.65	-1.16
-	344.35	301.81	745.43	697.76	-1.16
Lzts3	2600.13	1676.93	5839.54	3722.12	-1.16
mt-Rnr2	4258.66	5781.09	9035.91	13430.46	-1.16
Acadvl	1879.33	1861.75	4356.16	4017.97	-1.16
Aspg	901.73	1340.36	2258.71	2769.82	-1.16
Mif	2171.16	2145.76	5214.79	4461.19	-1.16
Rdh5	80.74	56.80	150.58	157.41	-1.17
Tufm	1455.22	1343.75	3490.05	2792.15	-1.17
4732463B04Rik	72.96	86.47	143.11	215.47	-1.17
Gm5560	33.07	27.98	84.37	52.47	-1.17
1500026H17Rik	31.13	34.76	82.23	65.87	-1.17
Glul	6878.24	7241.83	16689.90	15055.96	-1.17
Sostdc1	764.57	599.39	1792.02	1274.94	-1.17
Agpat3	4584.52	4790.02	10412.50	10685.20	-1.17
Mfsd2b	41.83	55.95	108.93	111.64	-1.17
Tmem37	1253.86	1084.33	2626.08	2638.09	-1.17
Ipcef1	136.18	122.93	306.50	276.87	-1.17
Acot1	1592.38	2679.02	3746.36	5882.39	-1.17
mt-Co1	224622.17	244263.07	508504.63	548831.37	-1.17
Ace	1790.81	1480.24	3547.72	3828.18	-1.17
Gm15962	70.04	60.19	150.58	142.90	-1.17
Apoa2	31.13	44.09	87.57	82.61	-1.17
Msrb1	2000.93	2170.35	4786.54	4630.89	-1.17
2410015M20Rik	916.32	892.72	2236.28	1847.67	-1.17
Shpk	214.00	265.36	517.95	566.02	-1.18
Gm37824	44.75	55.95	117.47	110.52	-1.18
Agmo	40.86	33.06	79.03	88.20	-1.18
Gys2	125.48	96.65	268.06	234.45	-1.18
Cited4	74.90	71.21	211.45	119.46	-1.18
Kegl	9735.18	8236.29	21490.32	19288.29	-1.18
Slc10a5	319.06	172.95	488.05	626.31	-1.18
Pdzd2	659.52	557.00	1385.13	1374.31	-1.18
Tcaim	367.70	256.03	723.00	691.06	-1.18
Bdh2	1593.35	1537.05	3628.89	3478.75	-1.18
Gm10732	34.05	22.89	60.87	68.10	-1.18
Apeh	2178.94	1941.44	4820.72	4539.34	-1.18
Efhd1	633.25	1108.91	2012.01	1950.38	-1.18
Tmem86b	112.84	118.69	309.70	216.58	-1.18
Tmem151a	103.11	128.86	268.06	260.12	-1.18
Calml4	1356.00	1273.38	3114.14	2863.60	-1.19
Immt	2751.88	2499.29	6137.50	5803.12	-1.19
Ndufs2	4254.76	4357.65	10509.68	9085.38	-1.19
Dnph1	202.33	217.03	492.32	462.20	-1.19
Gm16124	35.02	39.00	72.62	96.01	-1.19
Snrpn	52.53	72.91	138.83	147.37	-1.19
Hibch	403.69	401.01	886.40	945.60	-1.19
Cycs	2125.44	2180.52	4864.50	4945.71	-1.19
6820408C15Rik	31.13	16.96	56.60	52.47	-1.19
Tmem229a	2506.75	1978.74	5446.54	4773.79	-1.19
Stard7	1430.90	1388.68	3123.75	3303.47	-1.19
Ebp	615.74	686.71	1580.56	1392.17	-1.19
Uck1	781.11	896.12	1826.19	2002.85	-1.19
2810030D12Rik	57.39	50.87	120.68	126.15	-1.19
Mrps21	733.45	745.21	1897.74	1478.13	-1.19
Mcur1	1751.90	1481.94	3811.51	3571.41	-1.19
Glud1	6292.65	7034.12	13853.42	16597.73	-1.19
Syp	44.75	55.11	131.36	97.13	-1.19

Gm35281	26.26	22.04	49.13	61.40	-1.20
Spata2l	125.48	143.28	321.45	294.73	-1.20
Gm7730	170.23	183.97	468.83	342.74	-1.20
Gal3st1	358.94	299.27	891.74	615.14	-1.20
Mipep	373.53	384.05	905.62	830.61	-1.20
Sfxn2	457.19	476.46	1079.70	1061.71	-1.20
Atp1a1	66140.47	64054.87	159089.05	139511.56	-1.20
Uqcrb	990.25	1285.25	2372.98	2851.32	-1.20
2210408F21Rik	117.70	105.13	278.73	232.21	-1.20
Usmg5	1593.35	1414.12	3665.20	3236.48	-1.20
C030006K11Rik	475.67	451.87	1170.47	960.12	-1.20
Smim5	210.11	261.12	613.00	471.13	-1.20
-	35.99	32.22	64.08	92.66	-1.20
Chpt1	11383.97	11217.97	24095.05	27866.81	-1.20
Ankrd9	131.32	155.15	326.79	332.69	-1.20
Ndufs8	1228.57	1245.41	3120.54	2569.99	-1.20
L2hgdh	665.35	655.34	1453.48	1585.31	-1.20
Prkcz	899.78	868.14	2093.18	1976.05	-1.20
Tmem117	117.70	113.60	313.98	218.82	-1.20
Hint2	1064.18	989.37	2669.87	2064.25	-1.21
9630013D21Rik	25.29	47.48	101.46	66.98	-1.21
Dnajc19-ps	35.99	33.91	90.78	70.33	-1.21
Sfxn1	2300.53	2074.55	4984.11	5108.71	-1.21
Ndufb4	1168.26	1123.32	2841.81	2446.06	-1.21
-	61.28	105.97	281.94	104.94	-1.21
Kcnj12	35.02	22.89	63.01	70.33	-1.21
Mecr	424.11	410.33	1047.66	880.85	-1.21
Col4a4	2962.97	2375.51	6449.34	5898.02	-1.21
Ndufc2	753.87	721.47	1726.87	1685.79	-1.21
Tmem64	1164.37	1118.24	2836.47	2448.30	-1.21
Fitm2	430.92	596.00	1069.02	1311.79	-1.21
Paqr9	1332.65	916.46	2560.94	2645.90	-1.21
Gm7993	20.43	22.04	43.79	54.70	-1.21
Psat1	776.25	995.31	1621.15	2486.25	-1.21
Mpc2	2171.16	2429.77	5697.50	4971.39	-1.21
Gm5069	24.32	17.80	41.65	55.82	-1.21
Slc25a33	92.41	94.95	240.29	194.26	-1.21
Ugt2b34	211.08	118.69	379.12	384.05	-1.21
Gm15217	122.57	114.45	248.83	301.43	-1.22
Phyhipl	418.28	285.71	931.25	702.22	-1.22
Ndufs7	1592.38	1621.82	4151.11	3323.56	-1.22
Entpd8	135.21	148.36	337.47	322.64	-1.22
Reps2	335.60	269.60	732.61	674.31	-1.22
Ceacam1	224.70	230.60	450.67	609.56	-1.22
Slc25a21	118.67	91.56	281.94	206.54	-1.22
Shank2	616.72	373.03	1216.39	1085.15	-1.22
Ddah1	2470.76	2191.54	5293.82	5568.67	-1.22
Prodh2	902.70	690.95	2024.83	1688.02	-1.22
Tfrc	924.10	447.63	1910.56	1287.23	-1.22
Gm20743	23.35	21.19	50.19	53.59	-1.22
Esrra	1775.25	1739.67	4672.27	3525.64	-1.22
Slc25a16	1091.41	1052.96	2332.40	2672.70	-1.22
Vegfb	590.45	566.32	1602.99	1096.32	-1.22
Acsf3	679.95	607.02	1583.77	1418.96	-1.22
Slc26a10	35.99	51.72	156.99	48.01	-1.22
Tmem72	889.08	952.92	2274.73	2028.52	-1.22
Slc22a26	145.91	153.45	292.62	407.49	-1.22
Slc16a5	360.89	311.99	870.38	703.34	-1.23
Sms-ps	97.27	54.26	153.78	199.84	-1.23
Hgfac	118.67	143.28	347.08	266.82	-1.23
Gm15860	28.21	25.43	63.01	62.52	-1.23
Akr1c19	270.42	329.79	804.17	602.86	-1.23

Acss2	2089.45	1572.65	4418.10	4164.22	-1.23
Ephx2	2824.84	2831.62	6404.49	6858.13	-1.23
Pdha1	3326.77	3035.09	7281.27	7637.39	-1.23
Gm15920	604.07	643.47	1518.62	1407.80	-1.23
Mrpl12	921.18	896.96	2307.84	1955.96	-1.23
Slc7a8	2027.19	1821.06	4828.19	4196.60	-1.23
Rab17	473.72	541.74	1278.33	1105.25	-1.23
Cnnm1	164.39	233.14	461.35	473.36	-1.23
Rnf152	399.80	384.05	863.97	976.86	-1.23
Dlat	1468.84	1401.40	3516.75	3226.44	-1.23
Gabarapl1	5808.23	6846.76	13879.06	15867.59	-1.23
Ndufa11	974.69	875.77	2346.28	2005.08	-1.23
Prima1	32.10	42.39	89.71	85.96	-1.23
4930461G14Rik	26.26	25.43	76.89	44.66	-1.23
Ces2h	19.45	26.28	57.67	50.24	-1.24
C1qtnf12	699.40	584.13	1625.42	1395.52	-1.24
Ggct	228.59	256.03	589.51	552.62	-1.24
Fam151b	44.75	44.09	113.20	96.01	-1.24
Kcnj15	8517.31	7915.83	17764.25	20953.97	-1.24
Hepacam2	127.43	177.19	360.97	358.37	-1.24
Proc	1310.28	1208.95	3142.97	2793.27	-1.24
Tmem25	563.22	624.82	1564.54	1236.99	-1.24
Clcnkb	2126.41	2452.66	5755.17	5045.07	-1.24
Lyplal1	553.49	470.52	1256.98	1156.60	-1.24
Dglucy	1324.87	1180.97	3093.85	2818.95	-1.24
Gm47483	33.07	19.50	53.40	70.33	-1.24
A1cf	376.45	230.60	595.92	837.31	-1.24
2500002B13Rik	96.30	66.13	194.37	188.67	-1.24
AC131586.2	35.99	37.30	88.64	84.85	-1.24
Col4a3	2123.49	1509.92	4160.73	4434.40	-1.24
Gpam	760.68	657.89	1498.33	1858.83	-1.24
Tc2n	28.21	28.82	46.99	88.20	-1.24
Scd1	1803.46	2053.35	3530.64	5606.63	-1.24
Fam217a	60.31	44.93	124.95	123.92	-1.25
Pcyt2	1902.68	1810.03	4838.87	3964.39	-1.25
Tpk1	518.47	573.11	1224.94	1365.37	-1.25
Arhgef19	287.93	283.16	788.15	566.02	-1.25
Glud-ps	36.96	31.37	72.62	89.31	-1.25
A4gnt	89.49	95.80	245.63	194.26	-1.25
Odc1	3891.93	3306.39	8646.11	8436.74	-1.25
Sdhaf1	78.79	87.32	183.69	211.00	-1.25
Scp2	4726.54	4638.27	11261.51	10971.00	-1.25
Nat8f3	148.83	87.32	341.74	217.70	-1.25
Slc5a8	2038.86	1726.10	4061.41	4885.43	-1.25
Atp6v0a4	3717.81	3305.54	8248.83	8444.56	-1.25
Gpr137b	1253.86	1136.89	2921.91	2762.01	-1.25
Wnk1	3911.39	3289.43	8383.39	8742.64	-1.25
Gm45606	60.31	60.19	152.72	133.97	-1.25
Gm5425	88.52	83.93	228.54	181.98	-1.25
Cyb5a	3535.91	3596.33	8082.23	8908.98	-1.25
Mgam	757.76	562.93	1577.36	1567.45	-1.25
4833413G10Rik	61.28	65.28	134.56	167.46	-1.25
Gm11992	292.79	278.92	573.49	790.42	-1.25
Acsl3	223.73	166.17	544.65	384.05	-1.25
Coq8a	1275.26	1791.38	3535.98	3793.57	-1.26
Idh3g	2438.66	2244.95	5812.84	5381.12	-1.26
Mpst	917.29	829.99	2324.92	1852.13	-1.26
Ndufa5	977.60	969.03	2429.58	2228.36	-1.26
Atp2b2	43.77	49.17	136.70	85.96	-1.26
AC126028.1	24.32	19.50	52.33	52.47	-1.26
Gm16437	84.63	112.76	259.51	214.35	-1.26
Smim4	250.97	193.30	576.69	486.76	-1.26

Uqcrh	2171.16	2092.35	5577.89	4644.28	-1.26
Lrrc19	985.39	899.51	2014.15	2506.35	-1.26
Abcd3	3568.01	3605.66	7927.38	9286.33	-1.26
C8g	115.76	122.93	312.91	260.12	-1.26
Matn4	27.24	23.74	61.94	60.29	-1.26
Vwa8	2847.21	2588.31	6270.99	6782.22	-1.26
Acot12	1556.38	2044.02	3513.55	5141.09	-1.26
Ndufa8	1262.62	1286.95	3356.56	2774.29	-1.27
Ghr	5054.36	4287.28	11162.20	11342.77	-1.27
Dera	555.43	501.89	1314.64	1232.52	-1.27
Gm45495	34.05	18.65	66.21	60.29	-1.27
Prr5	495.12	552.76	1195.03	1334.11	-1.27
Ugt2b38	3408.48	2994.40	7201.18	8244.72	-1.27
Pink1	2651.69	2927.42	6851.96	6630.38	-1.27
Iyd	470.81	373.03	1069.02	970.16	-1.27
-	31.13	23.74	82.23	50.24	-1.28
Ndufab1	891.03	821.51	2164.73	1980.52	-1.28
Kynu	160.50	90.71	353.49	253.43	-1.28
Ndrg2	1097.25	1334.42	3134.43	2760.89	-1.28
Gm8566	114.78	105.97	255.24	280.22	-1.28
Ppara	425.09	278.92	811.64	894.25	-1.28
Tm7sf2	145.91	175.49	374.85	406.37	-1.28
AL589670.3	35.02	40.69	100.39	83.73	-1.28
Gm9905	21.40	16.96	40.58	52.47	-1.28
Cox5a	1675.06	1638.78	4397.81	3654.02	-1.28
Gm11789	26.26	43.24	75.82	93.78	-1.28
2010107E04Rik	1153.67	1243.71	3165.40	2667.11	-1.28
C2cd2l	1222.73	1089.41	2845.01	2778.75	-1.28
Ckmt1	696.48	683.32	2010.95	1348.63	-1.28
Cyp2c23	227.62	162.78	522.23	427.59	-1.29
Dao	3356.93	2827.38	7967.96	7126.07	-1.29
Rnf24	410.50	306.05	964.36	782.61	-1.29
Lypla1	971.77	1106.37	2485.12	2592.31	-1.29
Hnf4a	8242.02	7228.27	19151.52	18630.72	-1.29
Clybl	556.41	585.82	1485.52	1306.20	-1.29
Atp5c1	3941.54	3659.07	9512.22	9068.63	-1.29
mt-Nd5	57557.99	50831.02	120658.93	144670.51	-1.29
Gm2962	60.31	49.17	156.99	110.52	-1.29
Dclk3	430.92	445.09	1099.99	1047.20	-1.29
Ppa2	948.42	886.79	2265.12	2232.83	-1.29
Wdr91	785.97	852.03	1997.06	2020.71	-1.29
BC025446	478.59	424.74	1023.09	1191.21	-1.29
Rab11fip4	892.00	974.96	2251.23	2332.19	-1.30
Ngef	495.12	468.83	1472.70	893.13	-1.30
AC118733.1	12.65	26.28	45.92	50.24	-1.30
Rnf186	226.65	229.75	599.12	522.48	-1.30
Dmtn	1820.97	1791.38	4954.21	3924.20	-1.30
Fam120aos	277.23	261.12	690.96	631.89	-1.30
Mpzl2	72.96	47.48	159.12	136.20	-1.30
Kcne1	190.66	206.86	551.06	427.59	-1.30
Acad11	1715.91	1476.00	3806.17	4048.12	-1.30
Cyfip2	2043.73	2210.19	5826.73	4645.40	-1.30
Cox4i1	7739.12	7852.24	20879.46	17503.14	-1.30
Dnajc19	434.81	407.79	1102.12	972.40	-1.30
Ttc7b	356.02	271.29	752.90	790.42	-1.30
Pdp2	631.31	584.13	1469.50	1523.91	-1.30
Ndufa1	1002.89	1067.37	2639.97	2465.04	-1.30
Degs2	345.32	335.73	856.49	822.80	-1.30
Nos1	23.35	22.89	69.42	44.66	-1.30
Acnat1	276.26	222.97	585.24	647.52	-1.31
Ggnbp1	253.89	263.66	685.62	593.93	-1.31
Acadsb	1211.06	1114.00	2903.75	2844.62	-1.31

Plcd4	28.21	38.15	88.64	75.92	-1.31
Scp2-ps2	282.09	235.69	647.18	633.01	-1.31
Cbs	1462.03	1590.46	3699.37	3859.44	-1.31
Fndc9	15.56	37.30	75.82	55.82	-1.31
Slc39a8	447.46	492.57	996.40	1333.00	-1.31
Slc4a9	1340.44	1136.04	3126.95	3004.27	-1.31
Abhd14a	299.60	282.31	841.54	599.51	-1.31
Gfm1	966.90	903.75	2344.15	2290.88	-1.31
Irx1	514.58	578.19	1614.74	1094.09	-1.31
Ndufa4	3306.34	3516.64	8918.44	7997.99	-1.31
Tuba4a	1234.41	1401.40	3487.92	3053.39	-1.31
Mettl7a1	4316.05	3858.30	10374.05	9905.94	-1.31
Ndufb11	1779.14	1796.47	4850.62	4030.25	-1.31
Lrpap1	4241.15	3400.49	9811.24	9167.99	-1.31
Ppp2r2b	46.69	33.06	95.05	102.71	-1.31
Aadac	1004.84	911.38	2378.32	2384.66	-1.31
Gm43190	53.50	34.76	98.25	120.57	-1.31
Adrb1	25.29	35.61	68.35	83.73	-1.32
Dld	2361.81	2262.75	5780.80	5737.25	-1.32
Ndufv2	1474.67	1515.00	3869.18	3581.46	-1.32
Syt7	41.83	62.74	146.31	114.99	-1.32
Acsm3	1470.78	1597.24	3062.88	4591.81	-1.32
Cgref1	1347.24	1382.75	3546.66	3264.39	-1.32
Atp5j2	2069.99	1948.22	5449.74	4580.65	-1.32
Gamt	154.67	119.54	401.55	282.45	-1.32
Ccs	1095.31	1117.39	3047.92	2484.02	-1.32
-	54.47	46.63	135.63	117.22	-1.32
4430402I18Rik	47.66	39.85	117.47	101.59	-1.33
Cox6b1	2826.78	2715.48	7543.99	6355.75	-1.33
Sugct	722.75	744.36	1877.45	1806.36	-1.33
Plekhd1	158.56	210.25	472.03	455.50	-1.33
Slc9a2	501.93	553.61	1348.82	1303.97	-1.33
Glod5	224.70	171.25	526.50	466.66	-1.33
Slc16a9	1006.79	996.15	2403.95	2629.16	-1.33
Cndp1	334.62	319.62	830.86	813.87	-1.33
Ndufb5	920.21	942.74	2418.90	2266.32	-1.33
Hykk	1748.01	1372.57	4083.83	3762.32	-1.33
Kcnip2	46.69	39.00	102.52	112.76	-1.33
Ndufab1-ps	128.40	108.52	284.07	311.48	-1.33
-	51.56	85.63	162.33	184.21	-1.33
Gm2a	2871.53	2762.10	7341.08	6840.27	-1.33
Nat8	1424.09	1250.49	3653.45	3079.07	-1.33
Pccb	2799.55	2543.37	6851.96	6603.59	-1.33
Nudt6	52.53	59.35	155.92	126.15	-1.33
Dio1	353.10	292.49	868.24	758.05	-1.33
Adra2b	1580.70	1500.59	3762.38	4009.04	-1.33
Atp5o	2542.74	2451.81	6825.26	5771.86	-1.33
Nipsnap1	1193.55	1231.84	3299.96	2824.53	-1.34
Gm19950	1629.34	1450.57	3883.06	3894.05	-1.34
Uqcrc2	2785.93	2594.24	6916.03	6683.97	-1.34
Atp5f1	3597.19	3637.02	9326.39	8972.62	-1.34
Slc13a2	561.27	703.67	1659.59	1542.88	-1.34
Gatb	520.42	533.26	1318.92	1347.51	-1.34
Glyctk	566.13	724.01	1642.50	1626.62	-1.34
Tmem88b	36.96	61.89	93.98	157.41	-1.34
Fabp3	933.83	544.28	2254.44	1490.41	-1.34
Dhrs4	1684.79	1590.46	4330.53	3968.85	-1.34
1700018L02Rik	22.37	12.72	40.58	48.01	-1.34
Ogdh	6526.11	5364.82	16271.26	13897.12	-1.34
Sqor	956.20	919.01	2509.68	2249.57	-1.34
1810019D21Rik	300.58	284.86	760.38	725.67	-1.34
Oxld1	140.07	139.04	393.00	315.95	-1.35

Sucla2	1387.13	1478.55	3783.74	3503.31	-1.35
Fam81a	229.57	243.32	685.62	516.90	-1.35
4930512B01Rik	14.59	20.35	52.33	36.84	-1.35
Ndufs6	1026.24	1052.96	2989.19	2307.63	-1.35
Hspd1	3714.89	3562.42	9024.16	9524.13	-1.35
Fgf9	123.54	150.91	293.69	407.49	-1.35
Pdzd3	957.18	863.05	2550.26	2092.16	-1.35
2310039H08Rik	309.33	324.70	901.35	716.74	-1.35
Mettl26	1408.53	1357.31	3615.00	3443.02	-1.35
Rilp	173.15	235.69	602.32	443.22	-1.35
D630039A03Rik	345.32	422.20	867.17	1095.20	-1.35
Gm45769	45.72	64.43	124.95	157.41	-1.35
Gcat	91.44	91.56	246.70	221.05	-1.35
Pde4c	207.19	217.88	561.74	525.83	-1.35
Pyroxd2	1261.64	1238.62	3315.98	3080.19	-1.36
Myh14	737.34	679.93	1620.08	2007.31	-1.36
Sdhc	2235.36	2237.32	6119.34	5330.88	-1.36
Cavin2	253.89	270.45	621.55	722.32	-1.36
Ppp1r1b	373.53	611.26	1529.30	995.84	-1.36
AC160930.2	18.48	21.19	41.65	60.29	-1.36
Lrp2	13912.13	7944.65	27322.39	28788.97	-1.36
Macrod1	263.61	317.07	753.97	739.07	-1.36
Gm15879	32.10	25.43	65.14	82.61	-1.36
4932422M17Rik	66.15	52.56	193.30	111.64	-1.36
Sult1d1	5987.21	7094.32	14817.78	18881.91	-1.37
Gm28424	19.45	18.65	52.33	45.77	-1.37
F5	97.27	42.39	177.28	180.86	-1.37
Nol3	103.11	106.82	297.96	243.38	-1.37
-	62.26	22.89	131.36	87.08	-1.37
Gm11695	58.36	38.15	136.70	111.64	-1.37
Tmem174	2546.63	2558.63	6788.95	6413.80	-1.37
Echdc3	288.90	234.84	680.28	674.31	-1.37
Abcc6	151.75	130.56	386.60	343.86	-1.37
Gm13262	25.29	16.11	56.60	50.24	-1.37
Gsta3	553.49	534.96	1446.00	1376.54	-1.38
Hba-a2	112.84	150.91	335.34	350.55	-1.38
1810034E14Rik	91.44	56.80	234.95	148.48	-1.38
Cat	6105.89	6084.60	14813.51	16841.11	-1.38
Slc47a1	4464.88	4061.77	11155.79	11013.42	-1.38
Osgin1	769.44	880.86	1534.64	2758.66	-1.38
Cacnb4	77.82	83.93	243.49	177.51	-1.38
Glyat	3334.55	2631.54	8167.67	7367.22	-1.38
Cox7b	1739.26	1693.89	4694.70	4250.19	-1.38
Nrtn	145.91	183.12	431.45	427.59	-1.38
Prox1	109.92	77.15	302.23	184.21	-1.38
Gm10804	815.16	813.03	2018.42	2230.60	-1.38
Sgk2	974.69	1049.57	2877.05	2410.34	-1.38
Atp5d	4362.74	4216.07	12308.10	10102.43	-1.39
Ndufa13	1534.01	1507.37	4528.10	3438.56	-1.39
Insig1	329.76	245.86	900.28	606.21	-1.39
Acnat2	1452.30	1036.00	3207.05	3312.40	-1.39
-	109.92	67.82	259.51	205.42	-1.39
Tarm1	35.99	35.61	116.41	71.45	-1.39
Gm36028	21.40	16.11	49.13	49.12	-1.39
-	155.64	111.06	344.95	353.90	-1.39
Car12	2273.29	1839.71	5806.43	4992.60	-1.39
Aifm1	1278.18	1171.65	3444.13	2988.64	-1.39
Kyat3	410.50	428.98	1122.41	1082.92	-1.39
Fahd1	1088.50	938.50	2803.36	2524.21	-1.39
Atp5l	1401.72	1333.58	3639.57	3553.55	-1.40
Cpt2	1828.75	2206.80	4837.81	5785.26	-1.40
Hebp1	597.26	599.39	1623.28	1526.14	-1.40

Fxyd2	16659.14	17224.57	48506.21	40696.64	-1.40
Ech1	4291.73	4177.07	11045.79	11261.27	-1.40
Smpdl3a	2876.39	2796.02	7057.00	7891.93	-1.40
Slco4c1	175.09	255.19	457.08	679.90	-1.40
Slc22a1	3799.52	3426.77	10169.00	8897.82	-1.40
Fmc1	358.94	364.55	1031.64	883.08	-1.40
Akr1a1	13013.31	12287.04	35012.68	31959.58	-1.40
Uqcr10	2303.45	2113.54	6450.41	5241.56	-1.40
Mfsd4a	518.47	529.02	1489.79	1284.99	-1.41
Gm12350	45.72	42.39	126.02	107.18	-1.41
Slc2a9	207.19	227.21	607.66	545.93	-1.41
Gm5529	114.78	99.19	307.57	260.12	-1.41
Mrps23	63.23	28.82	171.94	71.45	-1.41
Nqo1	398.82	544.28	1078.63	1430.13	-1.41
Suclg1	2715.89	2565.42	7095.45	6945.21	-1.41
Inpp5j	401.74	640.93	1433.19	1345.28	-1.41
Spr-ps1	14.59	22.89	57.67	42.42	-1.41
Pim3	2095.28	1661.67	5302.36	4693.40	-1.41
-	65.17	61.89	148.44	189.79	-1.41
Chac2	171.20	178.88	543.59	388.51	-1.41
Mpc1-ps	682.86	718.08	1880.66	1851.01	-1.41
Cox5b	2500.91	2574.74	7599.52	5919.23	-1.41
Pmpcb	1354.05	1275.08	3519.96	3483.21	-1.41
0610043K17Rik	27.24	24.59	56.60	81.50	-1.41
mt-Nd6	15562.87	17795.99	42605.80	46340.11	-1.41
Idh3b	2411.42	2357.71	6649.05	6069.94	-1.42
Acsm1	875.47	680.78	2297.16	1851.01	-1.42
Slc2a12	101.16	63.58	237.08	200.95	-1.42
Lipg	30.15	12.72	77.96	35.73	-1.42
Slc13a4	33.07	44.93	126.02	82.61	-1.42
Cabcoco1	89.49	70.37	257.38	168.58	-1.42
Arl4d	176.07	113.60	443.20	329.34	-1.42
Aqp1	7858.77	5743.79	20166.07	16263.92	-1.42
Mlycd	606.02	759.62	1782.41	1878.92	-1.42
Pantr1	69.06	66.98	213.59	150.72	-1.42
Gm5475	20.43	13.56	40.58	50.24	-1.42
Tmem26	93.38	104.28	259.51	271.29	-1.42
Susd3	613.80	478.15	1730.08	1197.91	-1.42
Gm12251	72.96	94.95	234.95	216.58	-1.42
Ceacam2	128.40	211.10	433.59	480.06	-1.42
Atp6v1g3	319.06	387.44	1090.38	807.17	-1.42
Pank1	1980.50	2266.15	4766.25	6637.08	-1.42
Slc43a2	2281.08	2589.15	6163.13	6921.77	-1.43
Cox6a1	7093.22	6700.09	20551.60	16537.44	-1.43
Gm48135	44.75	61.89	132.43	155.18	-1.43
Atp5g3	5822.82	5664.09	16603.39	14296.80	-1.43
Ndufv1	3050.51	2913.86	8618.34	7430.85	-1.43
Letm1	1412.42	1366.64	3873.45	3607.13	-1.43
Rap1gap	1139.08	1294.58	3722.87	2839.04	-1.43
Dnajc6	154.67	132.26	386.60	386.28	-1.43
-	28.21	36.46	81.16	93.78	-1.43
Gm7652	24.32	21.19	49.13	73.68	-1.43
Mdh2	3694.47	3521.72	10623.95	8874.38	-1.43
Acot4	654.65	714.69	1752.50	1949.26	-1.43
Cdkl1	656.60	638.39	1962.89	1538.42	-1.44
Acot11	300.58	334.88	933.39	789.30	-1.44
Ndufv3	1239.27	1230.15	3516.75	3175.08	-1.44
Prdx5	10704.03	10418.51	30862.64	26381.98	-1.44
Lnx1	46.69	61.89	166.60	128.39	-1.44
Suclg2	2897.79	2800.25	7635.83	7814.90	-1.44
Selenbp2	80.74	61.89	253.10	132.85	-1.44
Ecsit	596.29	460.35	1504.74	1358.68	-1.44

Acox3	2372.51	2387.38	6398.08	6528.79	-1.44
Chrdl1	52.53	60.19	131.36	175.28	-1.44
Nudt19	3470.74	3106.31	9252.70	8609.78	-1.44
Cubn	2966.86	1927.03	6505.94	6784.45	-1.44
Esd-ps	13.62	10.17	29.90	34.61	-1.44
mt-Nd1	68763.95	65972.57	183002.55	183497.16	-1.44
Gm10053	234.43	311.99	740.09	750.23	-1.45
Slc22a2	2033.03	1861.75	5324.79	5281.75	-1.45
Dbt	817.10	647.71	1967.16	2025.18	-1.45
Nrap	22.37	15.26	57.67	44.66	-1.45
Acads	1579.73	1598.09	4606.06	4069.33	-1.45
Ndufa9	1599.18	1378.51	4373.25	3763.43	-1.45
Mccc2	641.04	595.15	1725.80	1652.29	-1.45
Anpep	1356.00	1136.89	3631.02	3180.66	-1.45
Ttc39c	303.49	338.27	821.25	934.44	-1.45
AC154227.1	26.26	12.72	50.19	55.82	-1.45
Slc8a1	328.79	174.64	686.69	687.71	-1.45
Tmem52b	1707.16	2534.90	5643.04	5981.75	-1.45
Cox7a2	1703.27	1658.28	4962.76	4247.96	-1.45
Gm47920	12.65	16.96	26.70	54.70	-1.45
Cox8a	5414.27	4891.76	15478.84	12801.92	-1.46
Hao2	1643.93	1149.60	4288.88	3382.73	-1.46
4931403E22Rik	10.70	16.11	42.72	31.26	-1.46
Dpep1	1074.88	1270.84	3040.45	3419.58	-1.46
Car9	101.16	94.10	263.78	273.52	-1.46
Hspb7	47.66	61.04	183.69	116.11	-1.46
Eif4ebp3	33.07	45.78	68.35	149.60	-1.46
Sirt3	575.86	541.74	1513.28	1567.45	-1.46
Gm45170	71.98	66.98	224.27	158.53	-1.46
Chadl	77.82	77.15	196.50	231.10	-1.46
Crat	1797.62	1793.08	4913.63	4995.95	-1.46
Usp2	1021.38	694.34	3210.25	1525.02	-1.46
Atp5e	1824.86	1781.21	5594.98	4359.60	-1.47
Bpnt1	2286.91	2050.81	6369.24	5637.89	-1.47
Egfl6	133.27	86.47	378.05	228.86	-1.47
mt-Tl1	18.48	34.76	53.40	94.90	-1.47
Cnnm4	195.52	173.80	481.64	541.46	-1.47
Gpr135	66.15	60.19	150.58	199.84	-1.47
Coq9	1506.77	1379.36	4284.61	3722.12	-1.47
Gm9115	29.18	30.52	87.57	78.15	-1.47
Acsm5	851.15	635.84	2157.26	1967.12	-1.47
Dgat2	1524.28	1573.50	4330.53	4271.40	-1.47
Cyp2b10	12.65	32.22	40.58	84.85	-1.47
Ybx2	305.44	315.38	1071.15	653.10	-1.47
Abhd14b	1043.75	941.90	3028.70	2485.14	-1.47
Gm39078	17.51	39.00	88.64	69.22	-1.47
Qdpr	1652.69	1469.22	4740.62	3936.48	-1.48
Defb29	266.53	164.47	663.20	532.53	-1.48
Xylb	3361.79	2374.66	8711.25	7237.71	-1.48
Cox6c	2796.63	2799.41	8241.36	7330.37	-1.48
Slc17a3	2686.71	2155.09	6320.12	7164.03	-1.48
Slc25a29	67.12	61.89	205.05	154.07	-1.48
Vwce	150.77	146.67	407.96	420.89	-1.48
Gm38319	20.43	7.63	34.17	43.54	-1.48
Gm13594	22.37	14.41	58.74	43.54	-1.48
Bcar3	266.53	217.03	682.42	666.50	-1.48
Mpv17l	2346.25	2426.38	6068.08	7312.51	-1.49
Tmie	153.69	112.76	397.28	348.32	-1.49
Gm45645	24.32	16.96	66.21	49.12	-1.49
-	62.26	63.58	194.37	158.53	-1.49
Bmp7	484.42	392.53	1425.71	1031.57	-1.49
Gm27343	7.78	16.11	40.58	26.79	-1.49

-	282.09	261.97	638.63	888.67	-1.49
Car4	1032.08	990.22	3388.60	2294.23	-1.49
Ddt	178.01	221.27	592.71	531.41	-1.49
Echs1	1772.33	1798.17	5326.93	4713.50	-1.49
Ndufb9	815.16	825.75	2446.67	2168.08	-1.49
Gm6415	10.70	20.35	57.67	30.14	-1.49
Slco1a6	1232.46	1080.93	2935.79	3581.46	-1.49
Bcat2	768.46	776.58	2538.51	1816.41	-1.50
Sdha	6383.12	6054.08	18201.04	16875.71	-1.50
Slc6a13	1208.14	1133.50	3493.26	3112.56	-1.50
Aldh7a1	2894.87	2707.00	8086.50	7725.59	-1.50
Etfa	2307.34	2374.66	6798.56	6431.66	-1.50
Ndufc1	671.19	626.52	2023.76	1644.48	-1.50
Uqcrc1	105.06	94.95	303.30	262.36	-1.50
Slc25a5	8413.23	8043.00	24784.94	21908.51	-1.50
Fmo2	2753.83	2588.31	7192.63	7973.43	-1.51
Magix	71.98	111.91	264.85	259.01	-1.51
Gm4459	124.51	89.02	336.40	269.06	-1.51
Atp5a1	523.33	471.37	1378.72	1451.34	-1.51
Stard10	3630.27	3769.28	10678.41	10390.47	-1.51
AC108858.2	16.54	17.80	57.67	40.19	-1.51
-	58.36	53.41	170.87	147.37	-1.51
Mgll	516.52	591.76	1688.43	1473.67	-1.51
Got2-ps1	19.45	16.11	57.67	43.54	-1.51
Gm31992	13.62	9.33	39.51	25.68	-1.51
Fbxo40	222.76	178.88	669.60	475.59	-1.51
Gm10221	475.67	493.41	1496.20	1272.71	-1.51
Slc46a3	202.33	135.65	552.13	411.96	-1.52
4732465J04Rik	22.37	26.28	76.89	62.52	-1.52
Gm11273	309.33	295.88	1011.35	720.09	-1.52
A630076J17Rik	14.59	12.72	52.33	25.68	-1.52
Prom2	222.76	225.51	692.03	591.70	-1.52
Ces2b	20.43	26.28	44.85	89.31	-1.52
Gpx3	224823.53	238286.99	672134.60	654396.13	-1.52
Csad	1902.68	1746.45	5182.75	5273.94	-1.52
Lhx1os	94.36	144.12	319.32	366.18	-1.52
Aldh8a1	2338.47	2375.51	6535.84	6982.05	-1.52
Sdhb	2422.12	2383.14	7270.59	6513.16	-1.52
Ptger3	367.70	345.05	1309.30	734.60	-1.52
Ehhadh	6494.98	8830.59	17295.42	26674.48	-1.52
Gm10039	630.34	552.76	1865.71	1530.60	-1.52
Tmem27	6440.51	6616.16	18446.67	19069.47	-1.52
mt-Cytb	108810.68	108493.54	304553.21	319993.28	-1.52
Aldh9a1	3671.12	3472.55	10161.53	10378.18	-1.52
Acox2	600.18	525.63	1669.20	1574.14	-1.53
Sod2	2168.24	2054.20	6521.96	5652.40	-1.53
Armc12	10.70	12.72	34.17	33.49	-1.53
Mep1a	9558.14	9364.70	25683.09	28911.77	-1.53
Nsg2	10.70	19.50	36.31	51.36	-1.53
Neu2	87.55	63.58	276.60	159.65	-1.53
Cisd3	342.40	359.46	1067.95	964.58	-1.53
Gm11615	21.40	20.35	68.35	52.47	-1.53
Ndufs1	1823.89	1531.96	5036.44	4700.10	-1.54
Mettl7b	1431.87	888.49	3650.25	3081.30	-1.54
mt-Nd4	47351.03	48077.39	132534.51	145187.41	-1.54
Ccdc151	41.83	34.76	140.97	81.50	-1.54
BC049987	91.44	86.47	279.80	237.80	-1.54
Bmp8a	15.56	14.41	45.92	41.31	-1.54
Gm11827	15.56	30.52	71.55	63.64	-1.54
Endog	290.85	265.36	897.08	727.90	-1.55
Mfsd4b5	2000.93	1608.26	5177.41	5372.18	-1.55
Nat8f7	32.10	25.43	112.13	55.82	-1.55

Gm14794	8.75	12.72	37.38	25.68	-1.55
C330002G04Rik	48.64	37.30	126.02	125.04	-1.55
Atp5b	24127.84	22205.35	72559.61	63143.26	-1.55
Bckdha	610.88	558.69	1766.39	1657.87	-1.55
Hmgcl	1045.70	1031.76	3014.82	3072.37	-1.55
Gm18009	13.62	14.41	29.90	52.47	-1.55
Fh1	1785.95	1693.04	4696.84	5518.43	-1.55
Tspan1	237.35	245.01	724.07	693.29	-1.55
Trim63	109.92	90.71	341.74	246.73	-1.55
Abhd3	551.54	481.55	1373.38	1664.57	-1.56
Uqcrq	2581.65	2480.64	8036.31	6861.48	-1.56
8430422M14Rik	47.66	60.19	203.98	113.87	-1.56
Ndufb10	1405.61	1298.82	4455.48	3506.66	-1.56
mt-Te	102.14	119.54	226.41	427.59	-1.56
Slc25a35	199.41	198.38	680.28	492.34	-1.56
Gm9826	100.19	50.02	218.93	222.17	-1.56
Hadh	3544.66	3421.69	10953.95	9606.74	-1.56
Ppat	1501.91	1440.40	4466.16	4221.16	-1.56
B230206L02Rik	14.59	16.11	41.65	49.12	-1.56
Etfb	450.38	376.42	1353.09	1088.50	-1.56
Aqp3	922.16	1160.63	3548.79	2610.18	-1.56
Hmcn2	96.30	118.69	369.51	267.94	-1.57
Asl	2191.58	2095.74	7154.19	5563.09	-1.57
Galnt11	4330.64	2826.54	11573.36	9660.33	-1.57
C230096K16Rik	12.65	10.17	36.31	31.26	-1.57
Pbld1	1412.42	1534.50	4367.91	4396.44	-1.57
Slc25a34	374.50	405.24	1110.67	1209.08	-1.57
Tln2	1551.52	984.29	4445.87	3104.75	-1.57
2610528J11Rik	446.49	370.48	1357.36	1075.11	-1.58
Cyc1	3555.36	3260.61	11098.12	9233.86	-1.58
Cyp4a32	13.62	25.43	49.13	68.10	-1.58
Pgk1-rs7	27.24	21.19	68.35	75.92	-1.58
Arsb	968.85	744.36	2543.85	2574.45	-1.58
Tmem52	30.15	44.93	128.15	97.13	-1.58
Gm37844	11.67	16.96	48.06	37.96	-1.58
Ccdc107	996.09	1038.54	3179.28	2921.66	-1.58
Aldob	42033.06	39999.64	115885.20	130298.91	-1.59
Phyh	2810.25	2607.81	8218.93	8068.32	-1.59
4833439L19Rik	6121.45	5255.46	18380.46	15830.75	-1.59
Shd	71.98	90.71	288.35	202.07	-1.59
Clcnka	825.86	886.79	3310.64	1856.60	-1.59
Lrat	37.94	36.46	90.78	133.97	-1.59
Sv2a	255.83	327.25	939.79	823.91	-1.60
Uroc1	420.22	518.85	1329.60	1510.51	-1.60
Gstz1	1651.71	1381.05	4656.25	4505.85	-1.60
Gm13855	85.60	105.97	270.19	310.36	-1.60
Rufy4	24.32	28.82	95.05	65.87	-1.60
Gpd1	3211.99	2854.51	9251.64	9103.24	-1.60
-	234.43	272.14	889.60	646.40	-1.60
Lpin1	524.31	632.45	1583.77	1924.70	-1.60
Atp6v1b1	1022.35	873.22	3467.63	2275.25	-1.60
Aco2	7098.08	6042.21	21066.35	18803.76	-1.60
Cisd1	1549.57	1515.85	4696.84	4615.26	-1.60
Lactb2	3227.55	2656.13	8643.97	9247.26	-1.60
-	51.56	61.04	163.40	179.74	-1.61
mt-Ty	18.48	35.61	63.01	102.71	-1.61
Acy1	714.96	697.73	2359.10	1949.26	-1.61
Colgalt2	120.62	123.78	422.91	322.64	-1.61
3110045C21Rik	33.07	40.69	124.95	100.48	-1.61
Uqcrfs1	3055.38	2810.43	9165.13	8731.47	-1.61
Sypl2	628.39	401.85	1833.67	1307.32	-1.61
Nlrp6	938.69	1147.06	3145.11	3226.44	-1.61

Gm14539	83.66	70.37	243.49	226.63	-1.61
Fn3k	184.82	164.47	560.67	506.85	-1.61
Hgd	2901.68	2411.97	8238.15	8033.72	-1.61
Notum	122.57	185.67	428.25	519.13	-1.62
Sptlc3	12.65	11.02	24.56	48.01	-1.62
Bphl	1390.05	1411.57	4309.17	4288.15	-1.62
Gm28438	36.96	41.54	120.68	120.57	-1.62
Slc28a1	127.43	138.19	462.42	353.90	-1.62
Gk	3667.23	2373.82	9581.63	9059.70	-1.63
Als2cr12	22.37	27.13	82.23	71.45	-1.63
Gm35549	7.78	11.87	26.70	34.61	-1.63
Gm5436	25.29	34.76	108.93	78.15	-1.64
Rhcg	1138.11	1268.30	4358.30	3128.19	-1.64
Rida	4280.06	4274.56	12810.04	13860.28	-1.64
Snx31	52.53	39.85	162.33	125.04	-1.64
Mdh1	8374.32	7329.16	25249.50	23739.43	-1.64
Gm16089	15.56	22.04	63.01	54.70	-1.64
Lpar3	230.54	200.93	743.29	601.75	-1.64
Wscd2	56.42	99.19	283.01	204.30	-1.64
Tlr12	246.10	189.06	854.36	502.39	-1.64
Gm13111	227.62	274.68	906.69	668.73	-1.65
Scnn1a	1430.90	1091.11	4391.40	3508.89	-1.65
Enpp2	3053.43	2563.72	8845.82	8769.43	-1.65
Sybu	8.75	7.63	24.56	26.79	-1.65
Svop	13.62	8.48	26.70	42.42	-1.65
Cryl1	2185.75	1903.29	6973.70	5876.80	-1.65
-	39.88	43.24	143.11	118.34	-1.65
Lrrc15	13.62	10.17	22.43	52.47	-1.66
4930481A15Rik	49.61	78.84	184.76	222.17	-1.66
Slc13a3	4802.42	3195.33	13535.18	11717.88	-1.66
Acacb	393.96	499.35	1613.67	1210.19	-1.66
Atp5g1	1770.39	1554.85	5872.65	4629.77	-1.66
Gm11127	19.45	16.96	77.96	36.84	-1.66
Grhpr	1650.74	1657.43	5539.45	4922.27	-1.66
Tmed6	86.57	72.91	259.51	244.49	-1.66
Kap	54482.19	62888.31	179231.62	193065.94	-1.67
Acsl1	2387.10	3034.24	7305.83	9899.24	-1.67
Gm4450	377.42	173.80	1121.35	627.42	-1.67
Slc5a11	450.38	430.68	1579.50	1218.01	-1.67
-	56.42	21.19	134.56	110.52	-1.67
Dnajc28	129.37	149.21	406.89	480.06	-1.67
Acox1	11218.61	11694.43	35112.00	37900.02	-1.67
Gm26684	25.29	18.65	72.62	66.98	-1.67
mt-Nd2	42885.18	42960.97	129393.68	144313.26	-1.67
Amacr	1075.85	923.24	3535.98	2843.51	-1.67
Cpt1b	6.81	13.56	32.04	33.49	-1.68
Cpeb3	362.83	269.60	1113.87	907.64	-1.68
Tldc2	41.83	31.37	137.77	96.01	-1.68
Ndufa10	1498.02	1471.77	5111.20	4409.84	-1.68
Sp5	133.27	170.41	544.65	430.94	-1.68
Ivd	3134.17	2997.79	10508.61	9167.99	-1.68
Slc39a5	436.76	388.29	1490.86	1155.49	-1.68
Agxt2	475.67	469.68	1530.37	1508.28	-1.68
Wnk4	1741.20	1798.17	6493.13	4892.13	-1.69
Slc38a3	637.14	1027.52	2000.27	3357.06	-1.69
Kcnj1	1563.19	1752.38	5692.16	4989.25	-1.69
D230017M19Rik	123.54	100.04	336.40	385.16	-1.69
Sectm1b	268.48	261.97	1047.66	667.62	-1.69
-	67.12	55.11	181.55	213.24	-1.69
Selenbp1	3242.14	3584.46	10993.46	11135.11	-1.70
Prok1	56.42	66.13	207.18	190.91	-1.70
Pdk2	2027.19	2385.68	7362.44	6963.07	-1.70

Fmo5	499.02	581.58	1687.36	1825.34	-1.70
Lhx1	244.16	244.16	674.94	913.23	-1.70
Fah	2906.55	2595.94	9372.31	8542.80	-1.70
Tcea3	358.94	295.88	1211.05	921.04	-1.70
2310016G11Rik	6.81	14.41	36.31	33.49	-1.71
Sall3	79.76	86.47	221.07	322.64	-1.71
Gm3716	64.20	53.41	231.74	151.83	-1.71
Slc6a20b	1240.24	779.97	3480.44	3119.26	-1.71
Uqcr11	1530.12	1469.22	5545.86	4290.38	-1.71
Uncx	12.65	8.48	54.47	14.51	-1.71
Prlr	150.77	166.17	482.71	558.21	-1.71
Klk1b9	5.84	13.56	38.45	25.68	-1.71
Hsd3b3	266.53	171.25	916.30	518.02	-1.71
Pcca	913.40	776.58	2816.18	2728.52	-1.71
Htr1b	13.62	20.35	68.35	43.54	-1.72
Slc2a5	612.83	579.89	1781.34	2136.82	-1.72
Slc26a7	13.62	10.17	54.47	23.44	-1.72
Mthfd1	1763.58	1635.39	5769.06	5409.03	-1.72
Gm27202	26.26	22.04	86.50	72.57	-1.72
Pcbd1	1184.80	1075.00	3919.37	3535.68	-1.72
Nat8f6	316.14	284.86	1316.78	666.50	-1.72
Aldh1l1	4430.83	4473.79	15065.55	14398.39	-1.73
Hibadh	2480.49	2328.88	8006.41	7905.33	-1.73
1700034P13Rik	9.73	6.78	30.97	23.44	-1.73
Shmt2	1552.49	1531.11	5520.22	4704.57	-1.73
Gpr179	6.81	10.17	33.11	23.44	-1.73
0610005C13Rik	661.46	600.24	2284.34	1902.37	-1.73
D10Jhu81e	1087.52	1068.22	4000.53	3165.03	-1.73
Aldoc	133.27	111.91	535.04	280.22	-1.74
Gcsh	629.36	564.63	2164.73	1817.52	-1.74
Klf1	25.29	22.04	79.03	79.27	-1.74
Ankrd65	9.73	25.43	60.87	58.05	-1.75
Cth	968.85	804.55	2985.98	2971.89	-1.75
Susd2	1494.13	1050.41	4611.40	3939.83	-1.75
Slc18a1	892.98	818.97	2901.62	2853.55	-1.75
-	28.21	23.74	79.03	96.01	-1.75
Slc25a10	3813.14	4493.29	13744.49	14326.94	-1.76
Gm31718	102.14	65.28	269.12	294.73	-1.76
Papss2	2044.70	1683.71	6970.50	5657.99	-1.76
Acaa1b	3178.91	3052.90	11644.91	9479.47	-1.76
Csrnp3	11.67	6.78	30.97	31.26	-1.76
Chchd10	1316.12	1233.54	4782.27	3866.14	-1.76
Atp5k	774.30	621.43	2717.93	2015.13	-1.76
Pbld2	164.39	145.82	601.25	452.15	-1.77
Slc12a3	10555.20	10092.11	35660.93	34613.30	-1.77
Vamp8	39.88	32.22	63.01	183.09	-1.77
Gm15348	28.21	22.04	60.87	110.52	-1.77
Ugt1a2	22.37	13.56	77.96	44.66	-1.78
Aass	2860.83	2428.92	9165.13	8993.83	-1.78
Ksr2	70.04	58.50	241.36	200.95	-1.79
Suox	652.71	681.62	2343.08	2262.97	-1.79
Etv1	133.27	79.69	362.03	371.77	-1.79
Vwa1	218.87	214.49	723.00	778.14	-1.79
Apoo	330.73	353.53	1230.28	1142.09	-1.79
Thnsl2	1429.93	1147.91	4698.97	4233.44	-1.79
Mtfp1	154.67	188.21	630.09	560.44	-1.79
Mfsd2a	101.16	161.93	421.84	493.45	-1.79
Tst	647.84	597.69	2434.92	1884.51	-1.79
Fam13a	436.76	554.46	1534.64	1910.18	-1.80
-	25.29	19.50	100.39	54.70	-1.80
Gm42067	24.32	21.19	83.30	74.80	-1.80
Adra1a	129.37	114.45	433.59	414.19	-1.80

Acss3	80.74	41.54	212.52	212.12	-1.80
Mut	1306.39	1152.15	4242.96	4337.27	-1.80
Xpnpep2	71.98	55.95	234.95	211.00	-1.80
Ugt3a2	5936.63	5915.89	18858.90	22560.49	-1.81
Lpl	1967.85	2251.73	7314.38	7439.78	-1.81
Hsd3b4	142.99	93.26	497.66	325.99	-1.81
Ak3l2-ps	66.15	26.28	170.87	150.72	-1.81
Gm5421	31.13	30.52	113.20	102.71	-1.81
-	15.56	16.96	71.55	42.42	-1.81
-	9.73	14.41	46.99	37.96	-1.81
Hnf4aos	37.94	55.95	200.77	129.50	-1.81
Sucnr1	103.11	78.84	404.75	232.21	-1.81
Idh2	3592.33	3358.10	13450.81	10995.56	-1.81
Smim24	768.46	701.97	2963.56	2213.85	-1.82
Tnfaip8	1343.35	1222.51	5101.59	3948.76	-1.82
Ntrk1	6.81	15.26	42.72	35.73	-1.82
Gm15889	164.39	109.37	530.77	433.17	-1.82
Gm4737	445.51	339.96	1419.30	1351.98	-1.82
Mccc1	891.03	764.71	2921.91	2936.17	-1.82
Etnppl	130.35	156.84	393.00	627.42	-1.83
Pxmp2	206.22	184.82	746.50	640.82	-1.83
Gm5512	10.70	10.17	40.58	33.49	-1.83
Shh	6.81	9.33	37.38	20.10	-1.83
Cyp4a10	3461.98	3524.27	10032.31	14798.07	-1.83
Rpl3l	12.65	22.89	53.40	73.68	-1.83
Khk	3421.13	3347.93	12528.10	11562.70	-1.83
Slc25a32	4.86	8.48	26.70	21.21	-1.84
Cuedc1	17.51	17.80	69.42	56.94	-1.84
Tmprss9	13.62	6.78	45.92	26.79	-1.84
Akr7a5	2152.67	2079.63	8308.64	6899.44	-1.85
Gm4956	27.24	27.98	101.46	97.13	-1.85
Cmbl	734.42	633.30	2695.50	2223.90	-1.85
Omd	4.86	21.19	25.63	69.22	-1.85
Gm29216	426.06	333.18	1283.67	1451.34	-1.85
Ppp1r1a	922.16	1306.45	4800.43	3258.81	-1.85
Kcnj10	642.01	569.72	2380.46	2007.31	-1.86
Adhfe1	1305.42	1150.45	4535.58	4359.60	-1.86
Ank2	94.36	71.21	338.54	260.12	-1.86
Bdh1	754.85	790.99	2925.11	2693.91	-1.86
Kl	4210.02	2643.41	12819.65	12118.67	-1.86
Ces2g	52.53	29.67	149.51	148.48	-1.87
D630024D03Rik	67.12	63.58	207.18	269.06	-1.87
Cwh43	143.97	196.69	577.76	667.62	-1.87
Ggt1	5682.75	5145.25	20112.67	19396.58	-1.87
Gpt	255.83	239.08	1029.50	779.26	-1.87
Casr	327.81	304.36	1182.22	1130.93	-1.87
Gcdh	2179.91	2076.24	7961.55	7635.16	-1.87
Tdrd12	10.70	5.09	37.38	20.10	-1.88
Slc22a19	283.07	398.46	1123.48	1391.05	-1.88
Aldh5a1	395.91	330.64	1316.78	1357.56	-1.88
Itih2	28.21	33.06	103.59	122.81	-1.88
Aldh4a1	2100.15	1759.17	8387.67	5860.06	-1.88
Syn2	49.61	55.11	225.34	161.88	-1.89
Slc25a25	880.33	1436.16	2502.20	6059.90	-1.89
Dhtkd1	279.18	215.34	985.72	842.89	-1.89
10-Mar	8.75	5.09	33.11	17.86	-1.89
Gm43667	36.96	29.67	122.81	123.92	-1.89
Acadm	4810.20	5131.68	17847.55	19128.64	-1.89
Bhmt2	762.63	611.26	2677.35	2438.25	-1.90
-	10.70	17.80	55.53	51.36	-1.90
Isoc2a	1217.87	1045.33	4769.46	3678.58	-1.90
Akr1c14	604.07	619.74	2205.31	2365.68	-1.90

Gm8947	28.21	11.87	95.05	53.59	-1.90
Emx1	336.57	343.36	1299.69	1243.69	-1.90
Aldh6a1	3889.99	3327.58	13134.70	13859.16	-1.90
Ttc36	549.60	475.61	2102.79	1738.26	-1.91
Gm34777	8.75	10.17	32.04	39.07	-1.91
Gm37336	12.65	12.72	41.65	53.59	-1.91
4833411C07Rik	5.84	6.78	10.68	36.84	-1.91
Azgp1	176.07	135.65	630.09	538.11	-1.91
Gm16755	21.40	27.13	80.10	102.71	-1.91
Rrh	10.70	11.02	43.79	37.96	-1.91
Bsnd	594.34	526.48	2251.23	1972.70	-1.91
Gm10222	74.90	85.63	340.68	265.71	-1.92
Macrod2	452.32	347.59	1456.68	1558.51	-1.92
Tmem163	9.73	20.35	61.94	52.47	-1.92
Aacs	1086.55	1246.25	4661.59	4163.11	-1.92
Acsm2	17200.96	14023.32	58175.41	59958.13	-1.92
Gm14320	9.73	9.33	33.11	39.07	-1.92
Gm13340	97.27	93.26	404.75	317.06	-1.92
Nap1l5	35.99	28.82	97.18	148.48	-1.92
Fa2h	70.04	88.17	376.99	224.40	-1.92
Tcn2	9837.32	8962.85	37159.26	34242.65	-1.93
Slc16a4	395.91	342.51	1289.01	1517.21	-1.93
Gm15638	97.27	67.82	309.70	317.06	-1.93
Gm28437	397.85	355.22	1626.49	1250.38	-1.93
Gm28661	1203.28	997.85	4654.12	3760.08	-1.94
Slc12a1	7401.58	7450.39	33227.07	23642.30	-1.94
Gm10639	16.54	24.59	100.39	58.05	-1.94
Acy3	6105.89	6323.67	25393.67	22368.47	-1.94
Fahd2a	404.66	366.25	1712.99	1250.38	-1.94
Gm12678	118.67	97.50	420.77	411.96	-1.95
Slc5a2	2753.83	2370.42	10380.46	9420.30	-1.95
Cyp2j11	658.54	590.06	2286.48	2540.96	-1.95
Aqp11	279.18	295.88	1144.84	1080.69	-1.95
Pcx	6138.96	4416.99	20267.52	20575.51	-1.95
Sardh	2724.64	1841.40	10626.09	7055.74	-1.95
Fancd2os	11.67	5.09	33.11	31.26	-1.95
Gm12231	23.35	19.50	84.37	81.50	-1.96
Ahcy	1949.37	1704.91	6995.06	7183.01	-1.96
Mfrp	14.59	12.72	74.76	31.26	-1.96
Bckdhb	213.03	228.90	1016.69	706.69	-1.96
Bco2	43.77	39.85	169.80	156.30	-1.96
-	25.29	15.26	70.48	87.08	-1.97
Slc22a18	2370.57	2198.32	9507.94	8353.01	-1.97
Pecr	1598.21	1186.91	5915.37	4970.28	-1.97
Gstk1	283.07	250.10	1069.02	1019.29	-1.97
Cyp2e1	9196.28	7221.49	35186.76	29225.49	-1.97
Abat	1888.09	1403.94	6462.15	6456.22	-1.97
Ranbp3l	77.82	48.32	317.18	176.39	-1.97
Sord	8775.09	6990.04	33661.73	28480.84	-1.98
Acaa2	1884.20	1729.49	7396.61	6855.90	-1.98
Slc37a4	1930.89	2147.46	7697.77	8406.60	-1.98
Gm6652	131.32	72.06	427.18	374.00	-1.98
Gm15318	71.98	66.13	272.33	274.64	-1.99
Ppp1r16b	423.14	385.75	1738.62	1465.85	-1.99
Akr1c21	3320.93	2884.19	12593.25	12065.09	-1.99
Comp	13.62	5.09	46.99	26.79	-1.99
1700101I11Rik	15.56	7.63	41.65	50.24	-2.00
-	26.26	27.13	119.61	93.78	-2.00
Gm15651	7.78	5.09	28.83	22.33	-2.00
Adgrg4	6.81	5.93	36.31	14.51	-2.00
Esrrb	285.01	202.62	1096.78	849.59	-2.00
Fam25c	20.43	11.02	70.48	54.70	-2.00

Halr1	31.13	30.52	122.81	125.04	-2.01
Cyp51	1171.18	887.64	4232.28	4045.88	-2.01
Mylk3	11.67	11.87	46.99	48.01	-2.01
Gm13341	24.32	24.59	99.32	98.24	-2.01
Cryzl2	1712.99	1483.63	6626.62	6295.46	-2.02
F13b	566.13	438.31	1969.30	2089.93	-2.02
Hsd3b2	503.88	342.51	1804.83	1638.90	-2.03
Gm16418	107.00	106.82	558.54	312.60	-2.03
Slc22a13	394.93	355.22	1506.87	1552.93	-2.03
Gm13339	12.65	11.87	56.60	43.54	-2.03
Gm47795	8.75	5.93	34.17	25.68	-2.03
Cyp2j7	10.70	7.63	49.13	25.68	-2.04
Slc16a14	412.44	250.10	1307.17	1404.45	-2.04
Tbx10	16.54	13.56	64.08	59.17	-2.04
Upb1	790.84	625.67	3239.09	2594.55	-2.04
Notumos	14.59	28.82	46.99	133.97	-2.05
Glt1d1	83.66	99.19	364.17	396.33	-2.05
Gm44421	11.67	12.72	53.40	48.01	-2.05
Gm28439	315.17	222.12	1133.09	1100.78	-2.06
Gm14257	18.48	15.26	74.76	65.87	-2.06
Acat1	3004.79	2954.55	12299.56	12689.16	-2.07
Mep1b	525.28	441.70	1959.69	2095.51	-2.07
Dnajc12	523.33	567.17	2237.35	2345.59	-2.07
Hist1h4h	14.59	25.43	86.50	82.61	-2.07
Cyp4a14	4557.29	4784.93	15518.36	23776.27	-2.07
Pm20d1	321.98	281.47	1299.69	1241.45	-2.08
Pex5l	5.84	6.78	26.70	26.79	-2.08
Lgr5	7.78	3.39	24.56	22.33	-2.08
-	5.84	3.39	27.77	11.16	-2.09
Col27a1	777.22	667.21	3170.74	2966.31	-2.09
Gm128	24.32	18.65	89.71	92.66	-2.09
Fbp1	5906.48	4590.79	21743.43	22952.36	-2.09
Zmynd10	130.35	133.10	639.70	485.64	-2.09
Hsd17b14	41.83	30.52	192.23	116.11	-2.10
Slc22a14	10.70	9.33	33.11	52.47	-2.10
Gm44386	66.15	50.87	284.07	215.47	-2.10
Ano3	44.75	21.19	138.83	141.78	-2.10
-	25.29	33.06	66.21	185.32	-2.10
Slc34a1	28039.23	18759.08	113017.76	88315.05	-2.11
Mymx	26.26	16.96	100.39	84.85	-2.11
Eci3	585.59	606.17	2556.67	2585.61	-2.11
Osr1	12.65	10.17	49.13	49.12	-2.11
4931406C07Rik	2910.44	2422.99	11619.28	11480.09	-2.12
Gm43618	5.84	5.93	27.77	23.44	-2.12
-	11.67	5.09	28.83	43.54	-2.12
Ggcx	7.78	5.09	49.13	6.70	-2.12
Nat8f1	1365.73	835.92	5961.29	3647.32	-2.13
Slc25a42	806.40	929.18	3420.64	4186.55	-2.13
Gm4952	15.56	4.24	54.47	31.26	-2.13
5830473C10Rik	8.75	5.09	42.72	17.86	-2.14
Fcamr	118.67	95.80	543.59	400.79	-2.14
Gm44120	201.36	284.01	1059.40	1097.43	-2.15
Gm31600	14.59	8.48	43.79	58.05	-2.15
Afm	235.40	163.62	977.17	794.89	-2.15
Gm15848	53.50	79.69	359.90	236.68	-2.16
Sec14l3	54.47	59.35	224.27	284.69	-2.16
Aspa	439.68	278.92	1653.18	1573.03	-2.17
Fam107a	401.74	469.68	2040.85	1883.39	-2.17
Rdh16	1061.26	976.66	4571.89	4596.28	-2.17
Phyhip	32.10	44.09	179.42	165.23	-2.17
Cyp7b1	535.98	846.10	2636.76	3604.90	-2.17
1700028J19Rik	57.39	78.84	350.29	269.06	-2.18

Serpinc1	5.84	5.09	16.02	33.49	-2.18
Acss1	1537.90	1543.83	7373.11	6604.71	-2.18
Mettl7a2	303.49	284.86	1208.92	1465.85	-2.19
Acmsd	322.95	188.21	1176.88	1143.21	-2.19
Gm48606	3.89	5.93	14.95	30.14	-2.19
Gm30784	9.73	2.54	32.04	23.44	-2.20
C1qtnf3	358.94	268.75	1284.74	1588.66	-2.20
Klf15	518.47	247.56	1970.36	1542.88	-2.20
Haao	805.43	683.32	3341.61	3501.07	-2.20
Slco1a4	6.81	22.89	46.99	91.55	-2.21
Kcnt1	138.13	150.06	811.64	519.13	-2.21
1700067K01Rik	25.29	27.98	154.85	91.55	-2.21
-	45.72	28.82	186.89	156.30	-2.21
Pah	3217.82	1881.25	13270.33	10335.76	-2.21
Akr1d1	112.84	80.54	452.81	439.87	-2.21
Gm10681	2.92	7.63	17.09	32.38	-2.21
Apom	220.81	225.51	1106.39	964.58	-2.21
Slc2a4	189.68	239.92	1188.63	807.17	-2.21
Kcne1l	21.40	8.48	89.71	48.01	-2.22
Hlf	511.66	331.49	2279.00	1643.36	-2.22
Retsat	1355.03	1081.78	6013.62	5338.69	-2.22
Stab2	47.66	20.35	170.87	144.02	-2.22
-	9.73	11.02	45.92	51.36	-2.23
Bglap3	6.81	4.24	44.85	6.70	-2.23
Fgfbp1	14.59	18.65	67.28	89.31	-2.23
Atp6v1c2	137.16	147.52	715.53	622.96	-2.23
Cela1	391.04	250.95	1762.11	1269.36	-2.24
Dcxr	940.64	818.97	4884.80	3449.72	-2.24
AI314278	418.28	289.10	1716.19	1641.13	-2.25
Fads6	213.03	252.64	1140.57	1077.34	-2.25
Aadat	752.90	841.01	3388.60	4203.30	-2.25
Oxgr1	153.69	145.82	703.78	723.44	-2.25
-	59.34	139.04	256.31	698.88	-2.27
Igfn1	6.81	1.70	18.16	22.33	-2.27
Klk1b24	1.95	7.63	23.49	23.44	-2.27
Gm6300	26.26	34.76	132.43	164.11	-2.28
Mogat2	79.76	71.21	435.72	294.73	-2.28
-	14.59	10.17	67.28	52.47	-2.28
Kmo	494.15	400.16	1942.60	2402.52	-2.28
-	85.60	43.24	373.78	250.08	-2.28
Malrd1	75.87	66.13	341.74	350.55	-2.29
-	49.61	16.96	185.82	138.44	-2.30
Asb11	19.45	29.67	118.54	125.04	-2.30
Gm44117	5.84	1.70	24.56	12.28	-2.31
Abca13	1057.37	636.69	4083.83	4319.41	-2.31
Fitm1	179.96	127.17	917.37	607.33	-2.32
Dusp9	60.31	64.43	293.69	328.23	-2.32
Mcrip2	577.81	438.31	2882.39	2199.34	-2.32
Chst9	7.78	4.24	37.38	22.33	-2.32
Slc22a30	338.51	248.40	1413.96	1539.53	-2.33
Grin3a	6.81	5.93	33.11	31.26	-2.34
D630023F18Rik	69.06	73.76	416.50	307.01	-2.34
Acot3	154.67	145.82	694.17	827.26	-2.34
-	48.64	44.09	243.49	226.63	-2.34
Upp2	154.67	232.29	598.05	1368.72	-2.35
Slc16a7	481.51	597.69	2837.54	2662.65	-2.35
Gchfr	130.35	130.56	681.35	650.87	-2.35
-	45.72	41.54	270.19	176.39	-2.36
Gm5424	1872.52	1400.55	9259.11	7554.77	-2.36
Zfp648	4.86	4.24	24.56	22.33	-2.37
Gm9442	3.89	5.09	29.90	16.75	-2.37
Gatm	92.41	85.63	482.71	439.87	-2.37

Slc6a19	1641.01	1194.54	7518.36	7200.87	-2.38
Gm2115	21.40	8.48	100.39	53.59	-2.38
AW822252	40.86	39.85	128.15	292.50	-2.38
Gm10925	622.55	552.76	2391.14	3742.22	-2.38
Bbox1	19.45	14.41	96.12	80.38	-2.39
Gm15883	52.53	35.61	249.90	214.35	-2.40
Perm1	44.75	16.96	218.93	106.06	-2.41
Dmgdh	464.97	399.31	2456.28	2132.35	-2.41
Pcsk6	161.47	178.04	1201.44	609.56	-2.41
Ugt3a1	820.99	802.01	3852.09	4818.44	-2.42
Nanp	6.81	3.39	9.61	44.66	-2.42
-	4.86	4.24	36.31	12.28	-2.42
C8a	79.76	74.61	404.75	422.00	-2.42
Mrap2	3.89	4.24	22.43	21.21	-2.42
Slc22a28	260.69	189.91	1155.52	1258.20	-2.42
Hist1h4c	2.92	5.93	25.63	22.33	-2.42
Tbc1d22bos	4.86	4.24	27.77	21.21	-2.43
0610006L08Rik	42.80	44.93	244.56	228.86	-2.43
Slc22a22	182.88	105.13	836.20	726.79	-2.45
Ass1	7295.55	5524.21	38451.48	31652.57	-2.45
Cyp2a4	1014.57	915.61	4829.26	5750.65	-2.45
Mmd2	2.92	8.48	32.04	31.26	-2.46
Gm12195	2.92	4.24	13.88	25.68	-2.46
Gm26685	3.89	5.93	29.90	24.56	-2.46
Gm48558	52.53	31.37	205.05	256.78	-2.47
Oxct1	4847.16	4188.94	25568.82	24775.46	-2.48
4930533I22Rik	5.84	4.24	20.29	35.73	-2.48
Slc26a4	357.00	328.10	1986.38	1848.78	-2.49
Gm15756	2.92	4.24	22.43	17.86	-2.49
Pik3c2g	28.21	11.02	122.81	96.01	-2.49
R3hdml	10.70	5.93	64.08	29.03	-2.50
Clec2h	312.25	149.21	1402.22	1206.84	-2.50
Rhbg	39.88	60.19	352.42	216.58	-2.50
Wfdc15b	366.72	464.59	2716.86	2005.08	-2.50
Klk1b5	9.73	14.41	89.71	48.01	-2.51
Kcns1	1.95	3.39	13.88	16.75	-2.51
Gm13528	8.75	13.56	56.60	71.45	-2.51
Cyp4f14	4.86	9.33	39.51	43.54	-2.54
BC024386	492.21	414.57	2883.46	2376.85	-2.54
-	6.81	8.48	60.87	29.03	-2.55
Cyp4b1	4077.73	4170.29	26285.41	22219.99	-2.56
Gm11788	7.78	19.50	66.21	96.01	-2.56
Angptl3	28.21	18.65	143.11	131.74	-2.56
Gm4208	18.48	5.93	102.52	40.19	-2.56
Lin7a	9.73	4.24	41.65	40.19	-2.57
Lrrc66	64.20	62.74	419.70	332.69	-2.57
Gm40787	2.92	4.24	18.16	24.56	-2.57
Gm26588	0.97	6.78	21.36	25.68	-2.57
Gata5	3.89	3.39	27.77	15.63	-2.58
Ak4	1370.59	944.44	8557.47	5306.32	-2.58
Serpina1d	425.09	665.52	2739.29	3847.16	-2.59
Ugt1a6b	0.97	5.93	17.09	25.68	-2.60
Slc22a6	4136.09	2493.35	21174.21	19102.96	-2.60
-	1.95	15.26	63.01	43.54	-2.61
Gm15441	9.73	35.61	108.93	171.93	-2.62
-	5.84	0.00	17.09	17.86	-2.62
-	16.54	18.65	129.22	87.08	-2.62
Gm33543	4.86	8.48	52.33	30.14	-2.62
Gjb6	11.67	2.54	57.67	29.03	-2.63
Gnmt	113.81	101.73	729.41	605.10	-2.63
Srp54	26.26	14.41	117.47	132.85	-2.63
Scn4b	210.11	375.57	1819.78	1820.87	-2.63

Fam151a	107.00	66.98	661.06	414.19	-2.63
Miox	5761.54	4366.97	31788.55	31093.25	-2.63
Vstm2a	21.40	22.89	145.24	131.74	-2.64
Serpina1e	1.95	2.54	13.88	14.51	-2.66
Aspdh	273.34	239.92	1766.39	1480.36	-2.66
Gm13412	2.92	4.24	26.70	18.98	-2.67
Slc22a29	25.29	4.24	108.93	75.92	-2.67
Serpina1c	3.89	2.54	13.88	26.79	-2.67
Rdh16f2	464.97	399.31	3120.54	2372.38	-2.67
Chrna4	234.43	222.12	1549.59	1358.68	-2.67
-	0.97	4.24	13.88	20.10	-2.68
Lrit3	7.78	0.85	29.90	24.56	-2.69
-	48.64	28.82	277.67	224.40	-2.71
Klhl3	288.90	362.85	2022.69	2260.74	-2.71
Tmem207	58.36	42.39	406.89	252.31	-2.72
Car5a	8.75	16.11	81.16	84.85	-2.73
-	15.56	12.72	92.91	94.90	-2.74
Pzp	102.14	105.13	587.37	800.47	-2.74
Stmn2	1.95	3.39	18.16	17.86	-2.74
-	2.92	1.70	11.75	18.98	-2.75
Pck1	13052.22	18852.33	91820.06	123791.34	-2.76
Gm10109	17.51	10.17	107.86	78.15	-2.76
Ces1f	1206.20	927.48	7600.59	6865.95	-2.76
4930563D23Rik	5.84	11.02	63.01	52.47	-2.76
Mreg	19.45	18.65	150.58	108.29	-2.77
Nkx3-1	4.86	7.63	29.90	55.82	-2.77
Hmx2	43.77	47.48	269.12	355.02	-2.77
Gm43031	9.73	2.54	49.13	33.49	-2.78
Gldc	1156.59	622.28	6177.01	6128.00	-2.79
Cox7a1	50.58	39.85	373.78	251.19	-2.79
Rnf212b	4.86	3.39	22.43	34.61	-2.80
Cyp2d9	658.54	813.88	4634.90	5604.40	-2.80
D630029K05Rik	380.34	267.05	2281.14	2249.57	-2.81
mt-Atp6	2067.07	513.76	10563.08	7563.70	-2.81
Cyp2c69	1.95	2.54	14.95	16.75	-2.81
Klk1b22	2.92	4.24	22.43	29.03	-2.84
Klkb1	13.62	5.09	64.08	68.10	-2.84
Ido2	21.40	28.82	165.53	196.49	-2.84
Cyp2f2	10.70	22.04	112.13	127.27	-2.86
Ces1g	4.86	3.39	30.97	29.03	-2.87
Pgam2	197.47	190.75	1525.03	1331.88	-2.88
Slc34a3	233.46	153.45	1528.23	1317.37	-2.88
Atp4a	180.93	239.92	1598.72	1530.60	-2.89
Car14	375.48	212.80	2621.81	1776.21	-2.90
Elavl3	0.97	4.24	22.43	17.86	-2.92
Tmem61	27.24	37.30	301.16	190.91	-2.93
Gm36864	4.86	5.09	48.06	27.91	-2.93
2410003L11Rik	5.84	3.39	65.14	5.58	-2.95
Gapdh	175.09	205.17	2641.04	299.20	-2.95
D630023O14Rik	2.92	9.33	42.72	53.59	-2.95
Cox6a2	2.92	5.09	48.06	14.51	-2.96
Ctcfl	1.95	1.70	11.75	16.75	-2.97
Gm17025	0.00	3.39	10.68	16.75	-2.97
Nr0b2	8.75	9.33	74.76	68.10	-2.98
-	4.86	2.54	49.13	8.93	-2.98
Gm15823	10.70	5.93	65.14	65.87	-2.99
Gm12315	1.95	0.85	8.54	13.40	-2.99
Abhd18	1.95	9.33	54.47	36.84	-2.99
G6pc	617.69	1245.41	6582.83	8403.25	-3.01
Gm5524	3.89	4.24	34.17	32.38	-3.03
Dpys	51.56	39.00	343.88	400.79	-3.05
-	9.73	9.33	72.62	85.96	-3.06

Gm17634	0.97	4.24	25.63	18.98	-3.07
Mecomos	2.92	3.39	34.17	18.98	-3.07
Gm12326	2.92	0.00	17.09	6.70	-3.07
-	3.89	5.09	71.55	4.47	-3.08
2200002J24Rik	0.97	1.70	11.75	11.16	-3.09
-	4.86	0.00	20.29	20.10	-3.10
Gm27239	1.95	3.39	25.63	21.21	-3.12
-	0.00	3.39	12.82	17.86	-3.13
Gpx6	131.32	115.30	1295.42	892.01	-3.15
Dpp6	1.95	0.85	19.22	5.58	-3.17
Ctnna2	2.92	3.39	35.24	22.33	-3.19
Nptx1	0.00	2.54	13.88	10.05	-3.19
1700003D09Rik	2.92	1.70	25.63	16.75	-3.21
Gm5131	1.95	0.85	21.36	4.47	-3.23
Gm45909	1.95	0.85	17.09	8.93	-3.24
Spp2	503.88	338.27	4441.60	3547.96	-3.25
4930434B07Rik	1.95	3.39	24.56	26.79	-3.25
Slc22a7	211.08	316.23	2218.13	2860.25	-3.26
Gm48851	0.97	2.54	17.09	17.86	-3.29
Nepn	45.72	22.04	394.07	263.47	-3.29
mt-Co3	785.97	568.87	7785.34	5593.23	-3.31
Cyp2d26	622.55	367.94	5317.31	4508.08	-3.31
Cyp4f15	1.95	0.85	11.75	15.63	-3.31
Slc7a13	412.44	497.65	4071.02	5013.82	-3.32
Gm48287	2.92	0.85	22.43	14.51	-3.32
Dnase1	772.36	528.17	8353.49	4774.90	-3.34
Gm29674	0.97	0.85	7.48	11.16	-3.36
mt-Atp8	28.21	11.02	257.38	151.83	-3.40
Klk1b27	2.92	2.54	32.04	25.68	-3.41
Slc6a12	17.51	13.56	252.04	77.03	-3.41
Lrrc52	3.89	1.70	28.83	30.14	-3.42
Dusp15	42.80	29.67	498.73	295.85	-3.46
1110019B22Rik	11.67	5.93	130.29	65.87	-3.49
Gm12962	1.95	4.24	34.17	36.84	-3.50
AY074887	3.89	0.85	21.36	31.26	-3.51
Sec14l4	2.92	5.09	35.24	56.94	-3.51
Gm47708	15.56	11.02	174.08	130.62	-3.53
4933417A18Rik	3.89	3.39	46.99	36.84	-3.53
Insrr	104.08	122.93	1475.90	1200.15	-3.56
-	5.84	1.70	40.58	46.89	-3.57
-	27.24	31.37	395.14	308.13	-3.58
Ces1d	654.65	391.68	7604.86	5028.33	-3.59
Pde6a	16.54	5.09	183.69	75.92	-3.61
Gpr37	1.95	0.85	16.02	17.86	-3.62
Klk1	3002.85	3263.15	39518.36	37863.18	-3.63
Gm29012	1.95	1.70	19.22	26.79	-3.66
Hrg	0.97	0.85	8.54	14.51	-3.67
B930092H01Rik	0.97	0.85	5.34	17.86	-3.67
Ctcflos	2.92	0.85	17.09	30.14	-3.68
-	1.95	0.85	25.63	10.05	-3.70
Ces1e	95.33	67.82	1177.95	1014.82	-3.76
Hhatl	22.37	24.59	342.81	296.97	-3.77
Hpd	149.80	402.70	2339.87	5352.09	-3.80
Cyp2d12	86.57	53.41	961.15	984.68	-3.81
Slc4a1	107.97	97.50	1712.99	1182.28	-3.82
Inmt	534.03	308.60	6963.02	4926.74	-3.82
Gm17597	1.95	0.00	21.36	5.58	-3.85
-	0.97	1.70	23.49	15.63	-3.86
C030005K06Rik	29.18	16.11	393.00	269.06	-3.88
Gm9973	4.86	11.87	146.31	111.64	-3.92
9030619P08Rik	20.43	11.02	256.31	218.82	-3.93
S100g	980.52	656.19	13395.28	12283.90	-3.97

Dnah11	4.86	1.70	75.82	26.79	-4.00
Bhmt	8.75	1.70	60.87	110.52	-4.07
-	3.89	1.70	55.53	37.96	-4.09
Ttr	182.88	228.06	3771.99	3572.52	-4.16
-	0.00	1.70	18.16	13.40	-4.16
Bfsp2	0.97	0.00	12.82	4.47	-4.23
Gssos1	0.97	0.00	11.75	5.58	-4.24
Stk32c	0.97	0.00	5.34	12.28	-4.26
Gm906	3.89	1.70	65.14	42.42	-4.29
Slc22a8	453.30	298.42	8117.47	6662.76	-4.30
Gm4756	1.95	0.00	10.68	26.79	-4.33
Calb1	534.03	496.81	10243.76	10723.16	-4.35
-	0.00	1.70	16.02	20.10	-4.35
Gm45727	1.95	0.00	21.36	16.75	-4.36
AC132878.3	1.95	0.85	34.17	27.91	-4.50
Odf4	1.95	0.85	39.51	23.44	-4.52
Gm45344	0.97	0.00	13.88	7.81	-4.56
Egf	1691.59	1088.56	33572.02	33736.92	-4.60
Grin2c	0.00	0.85	12.82	10.05	-4.67
Nccrp1	73.93	41.54	2323.86	745.76	-4.73
-	3.89	2.54	111.07	69.22	-4.82
Pappa2	2.92	0.00	33.11	45.77	-4.83
Rph3a	0.97	2.54	90.78	22.33	-4.98
6330410L21Rik	1.95	0.00	35.24	24.56	-5.02
Gm20684	0.00	0.00	6.41	4.47	-5.02
Gm8245	0.00	0.00	5.34	5.58	-5.03
Gm25545	0.00	0.00	4.27	6.70	-5.04
Gm18361	0.00	0.00	4.27	6.70	-5.04
Gm45623	0.00	0.00	10.68	1.12	-5.14
Gm11618	0.00	0.00	9.61	2.23	-5.15
Frmpd2	0.00	0.00	9.61	2.23	-5.15
-	0.00	0.00	8.54	3.35	-5.16
Gm48825	0.00	0.00	5.34	6.70	-5.17
2610028H24Rik	0.00	0.00	5.34	6.70	-5.17
Alb	4.86	1.70	110.00	122.81	-5.19
Sycn	0.97	0.00	23.49	10.05	-5.20
Gm11855	0.00	0.00	9.61	3.35	-5.28
-	0.00	0.00	8.54	5.58	-5.40
Gm4491	0.00	0.00	8.54	6.70	-5.51
Gm24515	0.00	0.00	5.34	10.05	-5.52
Epo	0.00	0.00	3.20	15.63	-5.82
Gm45650	0.00	0.00	11.75	7.81	-5.87
4930556H04Rik	0.00	0.00	8.54	12.28	-5.96
Gm5096	0.00	0.00	5.34	15.63	-5.97
Rtl4	0.00	0.00	10.68	15.63	-6.30
Hrh3	0.00	0.00	18.16	12.28	-6.51
Mlnr-ps	0.00	0.00	25.63	8.93	-6.69
-	0.00	0.00	60.87	36.84	-8.19
Pvalb	0.00	1.70	360.97	331.57	-8.58
